# Missing nurses cause missed care: is that it? Non-trivial configurations of reasons associated with missed care in Austrian hospitals – a qualitative comparative analysis

**DOI:** 10.1186/s12912-024-01923-y

**Published:** 2024-04-26

**Authors:** Ana Cartaxo, Hanna Mayer, Inge Eberl, Johannes M. Bergmann

**Affiliations:** 1 Vienna Doctoral School of Social Sciences, University of Vienna, Universitätsstraße 7, Vienna, Austria; 2 Austrian National Public Health Institute (Gesundheit Österreich GmbH, GÖG), Stubenring 6, Vienna, Austria; 3 Division Nursing Science With Focus On Person-Centred Care Research, Department of General Health Studies, Karl Landsteiner University of Health Sciences, Dr.-Karl-Dorrek-Straße 30, Krems, Austria; 4 Faculty of Social Work, Catholic University of Eichstätt-Ingolstadt, Ostenstraße 26, Eichstätt, Germany; 5 Münster Department of Health, FH Münster University of Applied Sciences, Johann-Krane-Weg 21, Münster, 48149 Germany

**Keywords:** Reasons for missed care, Unfinished care, Qualitative comparative analysis, QCA, Logical configurations, Acute care

## Abstract

**Background:**

Errors of omissions affect the quality of nursing care in hospitals. The Missed Nursing Care Model explains that the reasons for missed care are linked with 1) demand for patient care, 2) labor resource allocation, 3) material resource allocation, and 4) relationship and communication factors. Scientific evidence points to a lack of adequate nursing staffing as the most important factor triggering missed care. However, it remains unclear how the different theoretical reasons for missed care are interlinked with reports on missed care from the perspective of nurses in acute care settings. The aim of this study was to explore non-trivial configurations of reasons for missed care that are associated with missed care interventions from the perspective of nurses working in general units in Austrian hospitals.

**Methods:**

A cross-sectional study was conducted. Data collection was performed using the revised MISSCARE-Austria questionnaire. Our sample consisted of 401 nurses who provided complete data. Data were analyzed using qualitative comparative analysis. Configurational models of contextual factors, reasons for missed care, and missed nursing interventions were analyzed.

**Results:**

In our study contextual factors were not consistent precursors of the reasons for missed care. Missed care was consistently present when the demand for patient care was high. A lack of labor resources, in combination with the other known reasons for missed care, was consistently observed when missed care occurred. Different configurations of reasons were found to be non-trivially associated with different types and frequencies of missed care.

**Conclusions:**

To understand the complexity of the causal mechanisms of missed care, complexity theory may be necessary. Accordingly, a theoretical framework that acknowledges that complex systems, such as missed care, are composed of multiple interacting causal components must be further developed to guide new methodical approaches to enlighten its causal mechanisms.

**Supplementary Information:**

The online version contains supplementary material available at 10.1186/s12912-024-01923-y.

## Background

Missed nursing care (in short, *missed care*) is a concept, which was originally identified by Kalisch et al. [1] to describe implicit rationing of nursing care in general hospital units from the perspective of nurses in direct patient care. Missed care implies a failure to provide necessary aspects of nursing care and it poses a clinical error that is associated with negative patient outcomes, such as clinical complications and higher mortality, in combination with a higher financial burden for health care organizations [2, 3].

Research on missed care suggests that this problem is occurring worldwide; Jones et al. [4] found that between 55 and 98% of acute care nurses reported that they were unable to provide or complete the necessary care on their previous shift. Nurses in European countries have reported a particularly high incidence of omissions of care, with prevalence rates ranging from 75% (in England [5]) to 98% (in Switzerland [6, 7]).

Kalánková et al. [8] identified that omissions of care occur “across all categories of nursing care (…) such as documentation of care, emotional care and support, physical care, or coordination of care” (p. 1015). Furthermore, Griffiths et al. [6] found that care related to planning and communication, mobilization, oral and dental care, and participation in interdisciplinary meetings is omitted more often than clinical care. This suggests that nurses are failing to carry out important aspects of their autonomous professional practice, putting their status as nurses and the nursing profession itself at risk [9].

Because of the risks that missed care poses to quality of care and patient safety, there has been an increased focus on the causes of missed care [3, 10]. Nursing scientists are trying to understand the essential causal mechanisms of missed care to propose interventions to minimize it [11].

### Theoretical background

Kalisch et al. [12] developed a theory that emphasizes the multifactorial causal nature behind missed care occurrences. They defined missed care as “*any aspect of required patient care that is omitted (either in part or in whole) or delayed*” ([12], p. 1510), explaining that missed care pertains to “*repeated omissions of [nursing] care”* and not to* “the occasional occurrence or care that is missed in an emergency or in a noncrisis situation”* ([1], p 307).

**Fig. 1:**
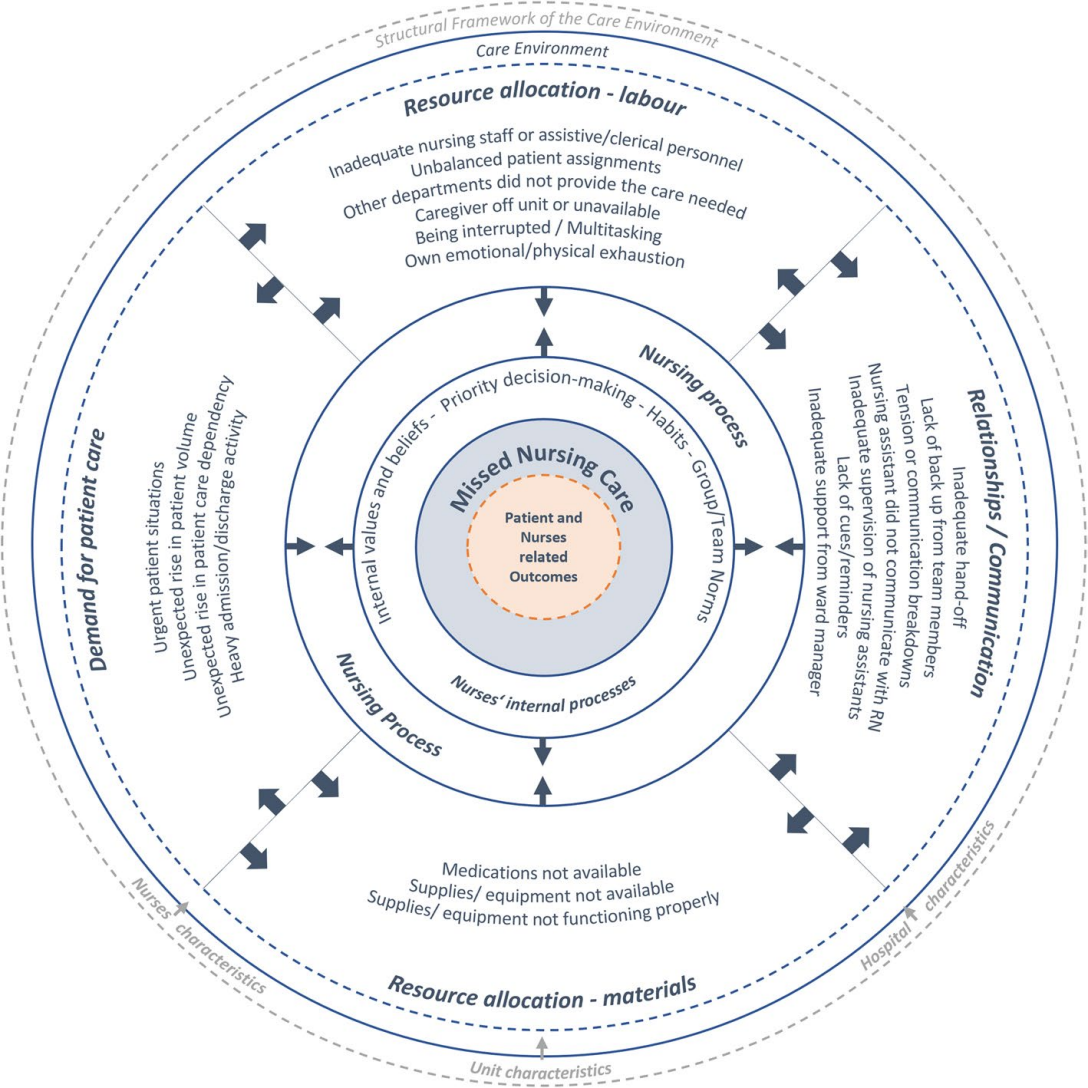
The middle-range explanatory theory of missed nursing care (Own interpretation and illustration; adapted from Kalisch et al. [12]) Content within image-derived Kalisch et al. [12], Kalisch and Williams [13], Dabney et al. [14]

The following are reasons for missed care: 1) high or complex demand for patient care, 2) poor labor resource allocation, 3) poor material resource allocation, and 4) unfavorable relationships and communication factors [12] (Fig. 1). These factors can be seen as theoretical prepositions for causal relationships in this model.

Alongside with the above explained reasons, contextual factors were identified as possibly important for missed care [2]. Hospital, unit, and staff attributes were postulated to influence the occurrence of missed care [2, 11]. Further research has demonstrated that the frequency of missed care can vary depending on factors such as hospital or unit size, as well as the qualifications and experience of nurses [11].

Kalisch et al. [12] further explained that the elements related to nurses’ internal processes need to be considered when understanding missed care. Kalisch [1] identified four internal processes that are relevant for the emergence of missed care: (1) group/team norms, (2) priority decision-making, (3) internal values and beliefs, and (4) habits. These elements seem deeply intricated with the individual nurses’ characteristics and may influence how nurses identify care priorities in the context of implicit rationing of nursing care. Kalisch et al. [12] clarified that if the presence of unfavorable reasons for missed care overwhelms the available internal resources of nurses and nursing teams, missed care will occur.

### Reasons associated with missed care in hospital settings

Considering the theoretical framework by Kalisch et al. [1, 12], we will now examine the reasons for missed care in more detail. A central reason for missed care is the poor allocation of labor resources. A large body of evidence links the lack of registered nurses in direct patient care to the occurrence of missed care as the most important reason for this phenomenon to occur [6]. If the number of nurses available is quantitatively lower, i.e., if their qualifications are qualitatively lower in relation to either the number of patients at the unit or the type, intensity or complexity of patients’ needs, then missed care is likely to occur because nurses do not have the time and/or expertise to carry out the necessary care [1, 6, 12].

Poor allocation of labor resources concerning other professionals and administrative staff also affects nursing care because nurses experience less support in clinical interventions and in administrative work. Here, nurses have to reorganize their priorities and sometimes forego nursing care that they would have been providing otherwise, to compensate for the lack of other professionals or administrative staff [1, 12].

The intensity and complexity of the demand for patient care poses a further important reason for missed care: when the urgency, dependency or complexity level of the patient’s situation is higher, the strain on the available labor resources is correspondingly greater [6, 12]. This also applies if the number of admissions and discharges is high because admitting patients and/or preparing them to leave means investing more resources in their transition to or from the hospital, as well as a high administrative workload [13]. In consequence omissions of nursing care are more likely to occur.

Factors related to labor resources seem to be intrinsically connected with factors related to relationships/communication in teams [15, 16]. Therefore, the third reason for missed care is concerned with the quality of relationships and the communication between patients and care team members of the unit, which involves nurses, physicians and other health care professionals and support staff [11, 12, 16]. This includes for example factors within the nursing care team, such as the communication of clinical information for the continuity of the nursing care process, the supervision of assistant nurses, or the provision of adequate support from nursing team members and nursing ward managers [13, 17].

Finally, poor allocation of material resources, including drugs, supplies and equipment, further hinders nurses’ ability to deliver necessary care interventions [13, 14]. If there is a lack of availability or functionality of material resources, it may not be possible to carry out instrumental and pharmacological interventions adequately, i.e., to carry out this type of intervention at all [12].

Empirical studies based on the middle-range explanatory theory of Kalisch and colleagues [1, 12] have consistently shown associations between the aforementioned reasons and the occurrence of missed care. Most of the evidence regarding these associations has been generated from the perspective of nurses in hospital settings using quantitative cross-sectional studies with surveys and statistical models based on inferential statistics [18]. Among these studies, multivariate analytical studies, which aim to bring into light the specific influence or impact of single reasons on the occurrence and frequency of missed care, have failed to identify high impact relationships in the data [19–21].

One reason for this may lie in the fact that these studies were conducted based on a reductionist approach, as explained by Albsoul et al. [18]. The authors made the case that this approach is inefficient and can lead to insufficient results because it does not consider the complexity of missed care and instead focuses on linear and symmetric relationships. In turn, they proposed that missed care should be regarded as a complex phenomenon [18].

To consider missed care as a complex phenomenon means that a more systemic approach is needed. In this sense, Albsoul et al. [18] proposed to consider missed care as occurring within a complex adaptive system, namely, *the hospital organization*. Holden [22] describes the key features of complex adaptive systems, stating that: 1) they consist of many elements interacting and sharing extensive information; 2) interactions between elements are diverse and nonlinear; 3) they operate within a specific context; and 4) that they are characterized by emerging patters such as organizational workplace cultures or (missed) care processes.

From a methodological perspective, this implies that the relationships between the identified reasons for missed care and its occurrence should be investigated considering nonsymmetric, nonlinear and interacting relationships and should be based on configurations and logical combinations of single cases, rather than solely on descriptions of single variables, their central tendency estimates and covariances, as in traditional statistical approaches; thus, a multiple configurational approach is needed. Such an approach makes it possible 1) to consider possible configurations of reasons that are particularly relevant for missed care to occur at a case level (*through sets of conditions, i.e., causal paths*); 2) to give qualitative meaning to measurements of reasons of missed care and of outcomes of missed care, for the purpose of interpreting the empirical data (*through calibration of data*); 3) to regard different possible theory-based models based on different sets of conditions that can lead to the same outcome (*according to the concept of equifinality)*; 4) or to consider the different roles of singular conditions, which can have different effects on a causal path to an outcome, depending on the causal configurations on which they are embedded (*according to the concept of causal complexity*) [23, 24].

### Missed care in Austrian hospitals

Between 2019 and 2023 the MISSCARE-Austria research project [21, 25–27] aimed to uncover missed care in general wards of Austrian hospitals and its reasons, based on the theoretical approach of Kalisch et al. [12] and on the revised MISSCARE Austria instrument [14]. In this project, 84% of nurses reported that fundamental patient care was regularly missed [25]. A complete list of publications related to the project can be found in Additional file 1.

The MISSCARE-Austria project investigated the reasons for missed care and their impact mainly using structural equation modeling and regression analysis. In these previous studies, it was found that singularly, the reason denoted as “allocation of labor resources” had a direct impact on missed care, which seems an incomplete finding according to the theoretical background by Kalisch et al. [12].

### Research gap

There is a large body of research that attempts to uncover the impact of contextual factors and of the known causes for missed care on the frequency and type of omissions of nursing care in hospital settings [11, 19, 28]. One finding has been consistent across the previously conducted research: missing nurses cause missed care [6, 29–32]. A lack of adequate nursing staffing plays a major role in missed care; this finding has been both logically and empirically consistent in nursing research and has developed into an obvious, almost trivial conclusion [33]. Still, a lack of adequate nursing staffing is not the only reason for omissions of care; reasons for missing care are various and possibly interact differently to generate emergent causal mechanisms [11, 18].

From this perspective, missed care is considered a complex phenomenon. Yet, the complexity of the associations between causal mechanisms that lead to missed care has not yet played a central role in previous quantitative studies [18], although this same complexity has been present in the theoretical underpinnings of missed care in nursing science [1, 12]. There is therefore a need to identify non-trivial relationships between known factors leading to missed care in hospital settings to further our comprehension of the emergence of this problem and develop preventive strategies. This is a gap that will be the subject of this study.

## Methods

### Aim

The purpose of this study is to explore 1) the multiple configurations of contextual aspects related to nurse, unit, and hospital characteristics that are associated with reasons for missed care and 2) the multiple configurations of reasons for missed care that are empirically associated with the occurrence of missed care using a qualitative comparative analysis approach. In doing so, we aim to explore the relationships between theoretical reasons for missed care and reports of missed care from the perspective of nurses in general wards of Austrian hospitals using an analytic method consistent with complexity theory.

### Design and sample

A cross-sectional study was conducted between August 2020 and February 2022. The research setting was general wards in Austrian hospitals. General wards were defined according to the Austrian Structural Health Plan [34] as conservative, surgical and both conservative and surgical units, where patients with clinically stable conditions, i.e., not requiring intensive medical care or continuous monitoring of vital signs, were admitted. The population was defined as professional nurses of all qualification levels [35]. Inclusion criteria for participation in the study were: 1) to be a registered nurse with at least a bachelor’s degree or a diploma in nursing; 2) alternatively, to be a nursing assistant with one to two years of training; 3) to have a minimum of one year of experience; 4) to work at the unit level in an Austrian hospital setting. Nurses working in intensive care units, pediatric units or ambulatory settings were excluded from the study.

Austria does not have a publicly available national register of nurses working in direct acute care that could be used for sampling purposes [36]. Furthermore, it is our perception that only a small proportion of nurses are inscribed in the national nursing association. Thus, a random sampling approach was not possible.

The recruitment period took place from 25^th^ May 2021 to 31^st^ July 2021. To ensure external validity, nurses were recruited using a semi-probabilistic approach based on snowball sampling [37] through both educational institutions and the national nursing association. 1) First, an online invitation to participate in the study was sent to all nursing educational institutions in Austria and to the national nursing association. These institutions redirected the invitation to participate in the survey to nurses working in direct patient care whose contact data were available in their internal newsletter registries. 2) Then, participating nurses were asked to redirect the study’s invitation to their colleagues working in the acute setting. The participants completed a written informed consent form before participating in the study. Data collection was anonymous, no personal information was collected. In this phase, inclusion and exclusion criteria were controlled using filter questions.

### Data collection

Data collection was conducted between May and July 2021 using the online version of the revised MISSCARE-Austria instrument [21, 38].

### Measurements

The revised MISSCARE-Austria includes 85 variables and was translated from the original American-English revised MISSCARE survey [14, 38]. The survey was developed and tested for validity and reliability [38] and consists of the following:

- ***Introductory section***: this section collects sociodemographic and work-related information on nurses, as well as data on ward and hospital characteristics (30 items);
- ***Section A***: this section surveys the frequency of omission of nursing interventions in the last two weeks from the perspective of nurses, which are considered theoretically representative for acute care nursing (30 items rated on a 6-point response scale with verbal endpoints ranging from 1 [“very rarely”] to 6 [“very often”] [14]. An option to identify nonrelevant interventions is included [-1 “not applicable”]);
- ***and Section B***: this section surveys the for missed care, operationalized according to the model of Kalisch et al. [12] model (25 items rated on a 6-point response scale with verbal endpoints ranging from 1 [“not decisive at all”] to 6 [“very decisive”]).

Psychometric testing of the original and of the Austrian versions has shown adequate statistics for construct validity and reliability [14, 21]. Considering the Austrian version of the instrument, a detailed explanation of the variables included in our study is presented in Table 1. Variables related to the care environment were defined as 2^nd^-level conditions, and 1^st^-level conditions were defined as the reasons for missed care.

**Table 1:** Variables: 1^st^- and 2^nd^-level conditions, missed care outcomes

	**Condition**	**Nr**	**Variable Name**	**ID**
**Outcomes (Section A)**	**Missed Nursing Care**	1	Ambulation/mobilization as frequently as necessary/as ordered	SA1
		2	Turning patient as frequently as necessary/as ordered	SA2
		3	Feeding patient when the food is still warm	SA3
		4	Setting up meals for patient who feeds themselves	SA4
		5	Administrating medications within the stipulated scheduled time	SA5
		6	Vital signs assessed as frequently as necessary/as ordered	SA6
		7	Monitoring intake/output	SA7
		8	Full documentation of all necessary nursing relevant data	SA8
		9.a	Patient teaching about illness and planed care	SA9a
		9.b	Informal caregiver teaching about illness and planed care	SA9b
		10.a	Emotional support to patient	SA10a
		10.b	Emotional support to informal caregivers	SA10b
		11	Assisting with body and skin care	SA11
		12	Assisting with oral and dental care	SA12
		13	Own hand hygiene	SA13
		14.a	Patient discharge planning	SA14a
		14.b	Counseling and training patients for discharge	SA14b
		14.c	Counseling and training informal caregivers for discharge	SA14c
		15	Bedside glucose monitoring as frequently as necessary/as ordered	SA15
		16	Performing comprehensive assessment of the patient’s condition	SA16
		17	Focused reassessments according to patient condition	SA17
		18.a	Peripheral venous catheter site care	SA18a
		18.b	Central line site care	SA18b
		19	Timely responding to patient call light	SA19
		20	Timely administrating PRN medication following patient requests	SA20
		21	Assess effectiveness of PRN medications	SA21
		22	Attend interdisciplinary care conferences	SA22
		23	Timely assisting with toileting needs following patient requests	SA23
		24	Skin damage and/or wound care	SA24
		25	Adequate surveillance of confused/impaired patients	SA25
**1**^**st**^**-level conditions (Section B)**	**Demand for patient care**	2	Urgent patient situations (e.g., a patient’s condition worsening)	**Demand** (mean score index)
		3.a	Unexpected rise in patient volume	
		3.b	Unexpected rise in patient care dependency on the unit	
		17	Heavy admission and discharge activity	
	**Relationship and communication factors**	7	Inadequate hand-off from previous shift or sending unit	**RelComm** (mean score index)
		11	Lack of back up support from team members	
		12	Tension/communication breakdowns w/SUPP.DEPARTMENTS	
		13	Tension/communication breakdowns within the NURSING TEAM	
		14	Tension/communication breakdowns w/INTERPROF. TEAM	
		15	Nursing assistant did not communicate that care was not provided	
		19	Inadequate supervision of nursing assistants	
		21	Lack of cues/reminders	
		22	Inadequate support from ward manager	
	**Labor resources allocation**	1	Inadequate number of nursing staff	**Labor** (mean score index)
		4	Inadequate number of assistive and/or clerical personnel	
		5	Unbalanced patient assignments	
		8	Other departments did not provide the care needed	
		16	Caregiver off unit or unavailable	
		18.a	Own emotional exhaustion	
		18.b	Own physical exhaustion	
		20.a	Frequently being interrupted during patient care	
		20.b	Multitasking	
	**Material resources allocation**	6	Medications were not available when needed	**Material** (mean score index)
		9	Supplies/equipment not available when needed	
		10	Supplies/equipment not functioning properly when needed	
**2**^**nd**^**-level conditions**	**Nurse Characteristics**	12	Nurse’s experience since first qualification in nursing	**NExp**
		6	Nurse’s highest nursing specific qualification	**NQual**
		7	Job Title/Role, on the unit she or he works on	**Role**
	**Unit Characteristics**	3	Type of the unit, on which she or he works on	**UT**
		13	How many beds are there in your unit?	**US**
		14	In the past 3 months, how often do you think nursing staffing was adequate on your unit?	**UNS**
	**Hospital Characteristics**	4.b	Type of hospital	**HT**
		4.c	Size of the hospital you work on (in bed-capacity)	**HS**
		4.a	Location of the hospital you work on	**HL**

ID – Variable Short Name in the DataSet

Nr. – Number of the item Section A, Sektion B and in the introductory section of the revised-MISSCARE Austria questionnaire, respectively

### Qualitative comparative analysis

Data analysis was conducted using qualitative comparative analysis (QCA) [39]. Qualitative comparative analysis can be used as an analytical tool when uncovering complex causal relationships in datasets as a complementary approach to regression analysis [39–41]. According to Parente and Federo [39], the procedure for conducting a qualitative comparative analysis, apart from data collection, consists of the following steps: 1) model specification, 2) data analysis and 3) presentation of results.

As QCA requires researchers to play an active role in defining thresholds and reference values for what can be considered the presence or absence of outcomes and manifestations of causal mechanisms, as well as in selecting the final solutions during data analysis, each step of the method needs to be clearly explained to ensure understanding of how and why a particular result is reached [41, 42]. With this in mind, we will now provide a detailed explanation of the implemented QCA approach in our study.

### Model specification

**Fig. 2:**
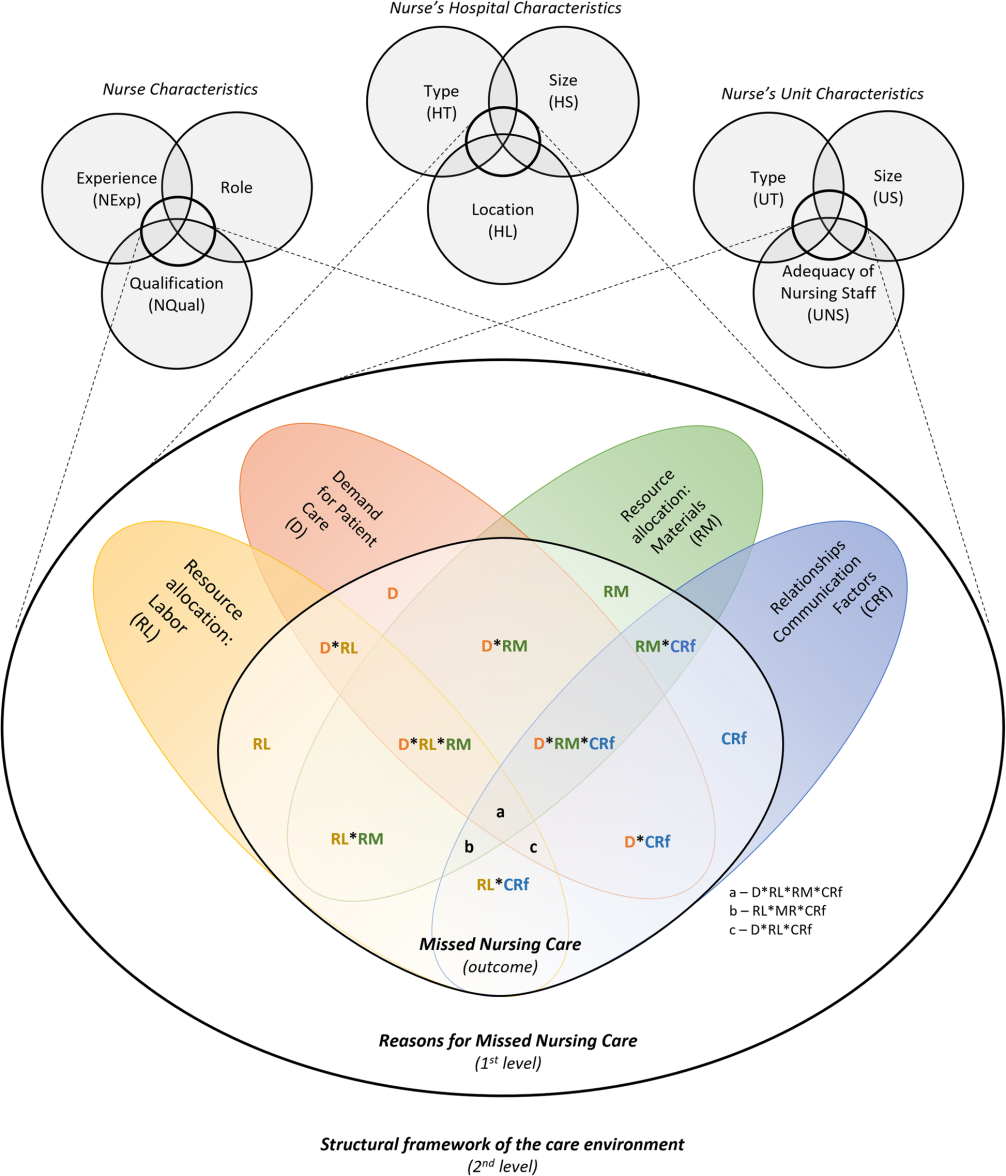
Venn diagram for reasons for missed care

After defining the participating nurses in our survey as the case level of our data analysis, we specified our configurational research model. This model consists of all possible configurations of reasons of missed care (1^st^- and 2^nd^-level conditions) according to Kalisch et al.’s [12] theoretical framework and including contextual factors. Reasons for missed care are represented in different sets of causal combinations that can lead to missed care (1^st^-level conditions). In addition, nurse, unit and hospital characteristics are considered contextual precursors of the reasons for missed care (2^nd^-level conditions) (Fig. 2).

Each of the 2^nd^-level conditions is represented by single variables, as depicted in Table 1. Each of the 1^st^-level conditions is represented by a *mean score index* consisting of the different quantitative variables, according to the operationalized theoretical constructs in the revised MISSCARE-Austria, which are also represented in Table 1. We constructed configurational models for each of the 30 outcomes presented in Table 1. We chose this approach because there is evidence of differences between missed care interventions in hospital settings, suggesting possible heterogeneity in the underlying causal mechanisms [43]. We included a maximum of 3 to 4 conditions per model to promote interpretability [44].

### Preparing data analysis: calibration

After excluding cases with missing values, the data were calibrated to define set membership. For 2^nd^-level conditions, we followed a crisp-set-based strategy. In crisp sets, conditions are binary, being considered as either present “1” or absent “0” in a particular causal path [24]. The calibration for 2^nd^-level conditions was conducted through the direct recoding of variables. For the calibration of conditions on unit and hospital characteristics, we followed a frequency distribution approach based on the data analysis of the previous MISSCARE-Austria studies [21, 25–27]. Here, the threshold was set at the mean values for the variables: unit type, hospital type and hospital size. The threshold for the remaining unit and hospital characteristics was based on the following central tendency statistics of the MISSCARE-Austria study’s sample [25]:

- ***UT)****“0”: surgical/medical-surgical units; “1”: medical units*
- ***US)****“0”: less than 30 patient beds; “1”: 30 patient beds or more*
- ***UNS)****“0”: nurse staffing adequate in the last 3 months; “1”: not adequate*
- ***HT)****“0”: private/nonprofit hospitals; “1”: public hospital*
- ***HS)****“0”: smaller than 500 patient beds; “1”: 500 patient beds or more*
- ***HL)****“0”: rural; “1”: urban*

The calibration of 2^nd^-level conditions regarding the nurses’ characteristics was carried out according to the following premises, which are based on empirical research evidence:

- ***NExp)****“0”: professional experience of 5 years or longer; “1” less than 5 years of professional experience.* Nurses with less experience perceive higher levels of missed care [20, 25, 29, 45]. The threshold was set at five years according to the literature on nursing expertise [46–48]
- ***NQual)****“0”: nursing assistants; “1” registered nurses.* Registered nurses perceive higher levels of missed care in comparison to assistant nurses [25, 49, 50]
- ***Role)****“0”: nurse managers; “1” nurses in nonmanagement positions in direct patient care.* Nurses in nonmanagement positions at the unit level report higher levels of missed care [25, 51]

When calibrating 1^st^-level conditions and the outcomes related to missed care, we followed a fuzzy set-based strategy. In fuzzy sets, conditions can vary between membership values of 0.0 (nonmembership) and 1.0 (full membership), indicating different degrees of belonging or not belonging. Here, crossover points indicate the qualitative anchors, where a change between belonging and not belonging occurs (0.5). To define the different degrees of membership, we followed a calibration by transformation approach based on the logit function according to Verkuilen [52] and Ragin [24] and implemented it as described in Duşa [53]. In addition, we varied set-membership values to explore differences in the obtained solutions [54], test the robustness of the obtained solutions [55, 56] and reflect on our assumptions regarding set-membership under consideration of theory-data fit.

Regarding 1^st^-level conditions, we defined set-membership values based on the symmetry of the mean index scales (which varied between 1 [“not decisive at all”] to 6 [“very decisive”]), setting the maximum ambiguity point in the middle of the scale [i.e., 3.5] and varying the extreme values, as follows:

- ***Low-calibration approach:***
  - full nonmembership = 1 │ crossover point = 3.5 │ full membership = 6
- ***Medium-calibration approach:***
  - full nonmembership = 1.5 │ crossover point = 3.5 │ full membership = 5.5
- ***High-calibration approach:***
  - full nonmembership = 2 │ crossover point = 3.5 │ full membership = 5

For the variables regarding the outcome missed care, the following thresholds were defined:

- ***Low-calibration approach:***
  - full nonmembership = 1 │ crossover point = 1.5 │ full membership = 6
- ***High-calibration approach:***
  - full nonmembership = 1 │ crossover point = 3.5 │ full membership = 6

Here, it seems important to clarify that although the outcomes regarding missed care were also scored on a 6-point Likert-scale (ranging from 1 [“very rarely”] to 6 [“very often”]), we decided to lower the maximum ambiguity point for set-membership values to 1.5 (low-calibration approach) because we were dealing with single variables and considered that in all the situations where the respondents entered a value greater than 1, nursing care was already being omitted to a degree and the outcome was already manifesting. Alternatively, we followed a less-sensitive calibration strategy with regard to the 2^nd^-level conditions, which was based on the logic of the scale (i.e., using set-membership values with a crossover-point of 3.5 [high-calibration approach]) to compare our results.

### Data analysis

The data analysis consisted of truth table construction, followed by necessity analysis and sufficiency analysis, and was concluded with an asymmetry analysis, according to the recommendations in Duşa [53, 57]. During the construction of truth tables, necessity analysis and sufficiency analysis we proceeded in three distinct phases:

1. **2**^**nd**^** level → 1**^**st**^** level:** In the first phase, we explored the configurations of 2^nd^-level conditions and their role in the emergence of 1^st^-level conditions.
2. **1**^**st**^** level → missed care:** In the second phase, we focused on configurations of 1^st^-level conditions associated with the presence of missed care outcomes, calculating one model for each variable related to the outcome.
3. **1**^**st**^** level → ~missed care:** In the third phase, the results of the second phase were tested against the absence of missed care according to De Morgan’s law [24] to discuss the asymmetry assumption (i.e., : “~missed care” means the negation/absence of the outcomes related to missed care).

Data analysis was performed in R using the package “QCA” [57] and the functions *calibrate()*, *truthTable()*, *pof()*, *superSubset() and minimize()*. The dataset and R code are available in the Zenodo repository [58].

#### Truth tables

After calibration, truth tables according to the defined configurational models were constructed. A truth table is a table with 2^ k^ lines (k refers to the number of conditions included), with all possible logical combinations between conditions and outcomes being studied [24]. By analyzing a a truth table, it is possible to determine how many cases with a particular logical combination of conditions (i.e., within a set) reported the presence of a missed care outcome. Based on these logical combinations, the principles of Boolean minimization and the joint method of agreement and disagreement were applied, which allowed us to uncover solutions in the next steps of data analysis [53, 57].

#### Necessity analysis

Following these principles, necessity analysis was conducted. Necessary conditions are those that are always present when a particular outcome occurs (i.e., they are a superset of the outcome: X ⇐Y). The outcome Y occurs in the presence of X; however, the condition X can also be present, without leading to the outcome Y [53, 57].

#### Boolean minimization and sufficiency analysis

Necessity analysis was followed by Boolean minimization and sufficiency analysis. Sufficient conditions are those that are able produce a particular outcome by themselves (i.e., they are a subset of the outcome: X ⇒Y) [24, 57]. If a particular condition X is present, it is expected that the outcome Y will also be present. By means of Boolean minimization, it is possible to reduce truth tables to find solutions that explain the set of conditions that are present and related to an outcome; as Duşa [57] explains: “The logical, or Boolean minimization process is the core of the QCA methodology, which seeks to find the simplest possible expression that is associated with the explained value of an output” (p. 159).

Due to the complexity of social phenomena, it is likely that multiple solutions emerge during a sufficiency analysis, because multiple combinations of conditions (i.e., X) are likely to be associated with the presence of a particular outcome (i.e., Y) [23]. Therefore, when conducting a QCA analysis, the research has to decide on the solution types that are most relevant to answer a particular research question. In our study, after initial Boolean minimization we focused on conservative solutions, followed by parsimonious and intermediate solutions because, according to Duşa [44], “a proper intermediate solution outperforms the parsimonious one in recovering a known causal structure and is positioned closest to the true, underlying causal model.” (p. 21). Conservative solutions only consider causal paths that are empirically present in truth tables, i.e., without including logical remainders. Logical remainders are “causal configurations that, due to the issue of limited diversity in social phenomena, have no empirical evidence” ([57] p. 178) and therefore are inconclusively connected to the outcome in the truth table (here, the outcome is represented with output = “?”). Parsimonious solutions are those that are generated after considering logical remainders during minimization, without contemplating whether these are theoretically or logically possible. Intermediate solutions offer more condensed configurations of conditions triggering the outcome than parsimonious solutions because—to a particular degree—they can deal with impossible, implausible or incoherent logical remainders and can resolve counterfactuals (e.g., conditions that are associated both with an outcome and with the absence of an outcome) if the researcher excludes problematic causal paths from the truth table and includes causal directional expectations [24, 44]. Because of the theoretical grounding of our study and of the extensive process of data analysis in our study, we focused on including directional expectations in the Boolean minimization process to generate intermediate solutions. Directional expectations can solve counterfactuals because they are theory-based expectations that hint about how causal conditions are present in a causal path where a particular outcome is also present [57], and in our case, filter out causal paths that are not in line with Kalisch et al. [12] theory.

#### Asymmetry analysis

According to Duşa [57], there are explanations for the occurrence of an outcome, and these may be different from the explanations for the non-occurrence of an outcome. To understand the complexity of the relationship between influencing conditions and the occurrence of an outcome it is therefore necessary to perform an asymmetry analysis as a final step of a QCA [24]. Here, researchers can analyze if a particular sufficient condition, that is always present when the outcome occurs, is also present when the outcome of interest does not occur. This could point to contradictory (theoretical) assumptions and guide researchers in redefining their model configurations.

#### Goodness-of-fit tests

When conducting QCA, it is possible that different possible solutions for an outcome emerge, revealing results that may not be empirically relevant or may be too complex to interpret. For this reason, different authors have recommended using different frequency thresholds (i.e., n.cut [minimum number of cases required in the causal paths] = 3, n.cut = 9 and n.cut = 15) and goodness-of-fit tests. This allows researchers to consider different possibilities of measurement error (i.e. to exclude causal paths with a low number of cases, considering them as logical remainders), i.e. to identify the most meaningful single solutions [40].

Goodness-of-fit indicators are normalized values between 0 and 1 and can be given as percentages; results that are close to 1 represent a better fulfillment of the different goodness-of-fit. We differentiate between goodness-of-fit indicators that are relevant for the interpretation of both necessary and sufficient solutions (i.e., consistency and coverage scores) and those that are specifically useful to interpret the results of necessity or sufficiency analysis (i.e., the proportional reduction in inconsistency and the relevance of necessity):

1. **Consistency scores for necessary **(inclN)** and sufficient** (inclS) **solutions** are defined as the proportion of cases that share the same causal path associated with an outcome [59]. Thresholds for inclS and inclN scores were set according to Ragin [24] at 0.8 and 0.9, respectively. When exploring consistency scores, we varied cutoff values for the number of cases in causal paths, beginning with an initial frequency threshold of *n*=3, which we changed to *n*=9 and *n*=15 for testing robustness during data analysis.
  - **Proportional reduction in inconsistency** (PRI): To interpret inclS scores, an indicator regarding the potential logical contradictions of sufficient solutions was developed. The PRI is a measure used to explore simultaneous subset relations in situations where a solution appears to be sufficient to trigger both an outcome and its negation [57]. During sufficiency analysis, we considered causal paths with (inclS)>0.8 and a proportional reduction in inconsistency (PRI)>0.75 as particularly sufficient, especially when the value of PRI was equal to or close to the inclS score, meaning that a causal path was specific to explaining an outcome and not relevant to explaining the emergence of the negation of the outcome.
2. **Coverage scores for necessary** (covN) **and sufficient** (covS) **solutions** are parameters that explore how much of the outcome is explained by the solutions being studied: “*Raw coverage indicates how much of the membership in the outcome is covered by the membership in a single path (…). The solution coverage expresses how much is covered by the entire solution* term” ([40], p. 139). Our threshold for coverage was (cov)>0.75.
  - **Relevance of necessity** (RoN): The RoN describes whether there are significantly more cases in which a particular necessary causal path is present, but the outcome is absent, than cases that share the same causal path but show that the outcome is present; a necessary causal path is deemed trivial if this is the case. The threshold for relevance of necessity (RoN) was set at >0.5. It is important to explain that both the CovN and RoN are goodness of fit indicators used to assess trivialness from different perspectives. The CovN checks if a particular causal path is not relevant because it is present mostly in cases where the outcome does not occur. The RoN checks if a particular causal path is not relevant because it is a universally expected condition (i.e., close to a constant) and therefore not particularly relevant to explain the presence of a particular outcome [27].

### Presenting results

Solutions were presented using an adapted configuration chart based on Fiss [60] and Rutten [54], focusing on configurations of emerging conditions found during Boolean minimization (as opposed to single solutions within every single model). Our article was written according to the recommendations of the COMPASSS (COMPArative Methods for Systematic cross-caSe analysis Network [42]) and according to best practice recommendations for applying QCA in health-related sciences [61, 62].

## Results

### Participants

From an initial sample of 1006 nurses, a total of 401 persons provided complete data for the variables of interest and were considered for further data analysis. Eight percent (*n* = 31) of the respondents had training as a nursing assistant as their highest qualification, while 92% were registered nurses, of whom 18% worked as ward managers (*n* = 67). The majority of the respondents had more than 5 years of experience since qualifying for professional practice (m = 12.9 years, sd = 9.6) and worked in medical wards (*n* = 210 vs. *n* = 181, who worked in surgical/medical-surgical wards).

The mean unit size of the participants was 30.6 patient beds (sd = 9.8). More than half worked in urban (*n* = 292, 72.8%) and/or public hospitals (*n* = 267, 66.6%) with more than 500 patient beds (*n* = 175, 43.6%). Overall, 71.6% of nurses (*n* = 287) reported experiencing inadequate nursing staffing in their teams in the last 3 months.

The most frequently reported reason for missed care was *high demand for patient care* (m = 4.6, sd = 1.1), followed by *poor labor resources* (m = 4.1, sd = 1.0), *difficulties in relationships and communication* (m = 3.0, sd = 1.1) and *poor material resources* (m = 2.7, sd = 1.3). The mean frequency of missed care across all outcomes was 3.0 (sd = 1.0). Descriptive statistics are presented in Table 2 and show that both the 2nd- and 1st-level conditions, as well as the outcome, were empirically diverse across cases.

**Table 2:**
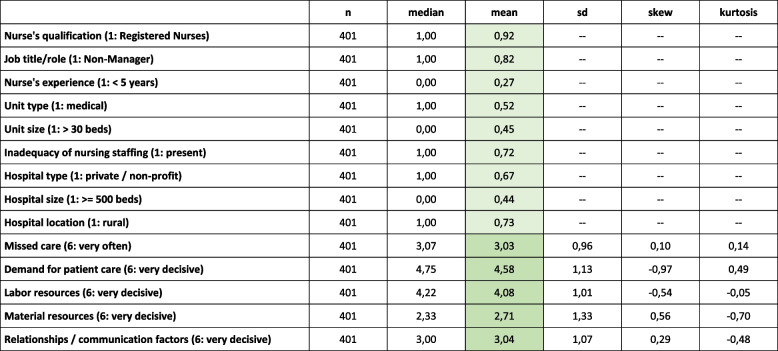
Sample characteristics

n: number of cases; sd: standard deviation; light green: mean values concerning binary contextual factors (2^nd^-level conditions) represent the proportion of cases with the value = 1; dark green: mean values concerning reasons for missed care (1^st^-level conditions) and the mean index of missed care

### Truth tables

In the ***first data analysis phase***, we focused on configurations of contextual factors leading to the presence of reasons for missed care. At this stage, we observed causal paths in the truth tables showing that nurses’ experience, role or qualification were important for triggering nurses’ perception of a *high demand for patient care* (inclS: 0.808–0.872; PRI: 0.783–0.853; *n* = number of cases in the causal paths between 9 and 96 cases [9–96]); the lack of adequate nurse staffing, a larger unit size or unit type were important for triggering a *high demand for patient care* (inclS: 0. 805–0.872; PRI: 0. 760–0.856; *n* = 66–73) and *poor labor resources* (inclS: 0.806; PRI: 0.769; *n* = 67)*;* and hospital characteristics were associated with *high demand for patient care* through different causal paths (inclS: 0.802–0.947, PRI: 0.768–0. 944; *n* = 7–54).

Apart from the abovementioned causal paths, we did not observe any configurations of contextual factors in truth tables that consistently led to the occurrence of reasons for missed care. Two causal paths were identified as logical remainders: a causal path concerning assistant nurses in management positions and more than five years of experience (~ NQual + ~ Role + ~ NExp) and a causal path concerning assistant nurses in management positions and five years of experience or less (~ NQual + ~ Role + NExp). Because these causal paths were logically improbable, given that leadership positions in nursing teams in hospitals typically require the expertise of registered nurses, and also because most of the causal paths that we observed in the truth tables regarding nurse characteristics were not associated with the presence of an outcome, we considered these remainders to be unrelated to the outcome.

In the ***second phase,*** we constructed truth tables for all of the possible configurations of 1^st^-level conditions leading to the 30 different outcomes regarding missed care. For the low-calibration approach, most causal paths that led to a missing care outcome consistently included the conditions of *poor labor resources* and/or *high demand for patient care,* whether alone or in combination with other 1^st^-level conditions. This means that we observed multiple configurations of conditions leading to missed care, where *poor labor resources* or *high demand for patient care* were especially important for triggering the outcomes of missed care. This also implies that the conditions of *difficulties in relationships and communication* and *poor material resources* were present only in some causal paths and mainly in combination with the abovementioned conditions. Some outcomes were consistently absent when 1^st^-level conditions were present.

Using the low-calibration approach (crossover point = 1.5), the following missed care outcomes were absent for any possible configurations of reasons for missed care:

- ***SA4:*** *Setting up meals for patients who feed themselves*
- ***SA13:*** *Own hand hygiene*
- ***SA15:*** *Bedside glucose monitoring as frequently as necessary or as ordered*
- ***SA18.a:**** Peripheral venous catheter site care*
- ***SA18.b:*** *Central line site care according to hospital standards*
- ***SA24:*** *Skin damage and/or wound care*

These results indicate that the depicted configurations may be insufficient to explain the occurrence of these particular outcomes in our dataset, even when considering a highly sensitive calibration approach. It is also important to note that we observed multiple logical remainders within all of the constructed truth tables relying on the low-calibration approach. None of these logical remainders seemed to pose logical impossibilities, since they can all be grounded on the theoretical propositions of the Missed Nursing Care Model [12].

Using the high-calibration approach (crossover point = 3.5), we found that a greater number of outcomes was absent in single causal paths. However, we found that the previously observed configurations of reasons for missed care remained consistently present, when the following missed care outcomes were present:

- *SA1: Ambulation/mobilization as frequently as necessary or as ordered*
- *SA8: Full documentation of all necessary nursing relevant data*
- ***SA9.a/SA9.b: Patient and Informal caregiver teaching about illness and planned care***
- ***SA10.a/SA10.b: Emotional support to patient and to informal caregivers***
- *SA14.b/****SA14.c: Counseling and training**** patients and ****informal caregivers for discharge***
- *SA17: Focused reassessments according to patient condition*
- *SA19: Timely responding to patient call lights*
- *SA21: Assess the effectiveness of PRN medications*
- ***SA22: Attend interdisciplinary care conferences***
- *SA25: Adequate surveillance of confused/impaired patients*

Adequate goodness-of-fit scores were observed for causal paths included in the outcomes SA9.a, SA9.b, SA10.a, SA10.b, SA14.c and SA22 (***in bold***). This may indicate that the considered reasons for missed care are especially meaningful to explain why nurses omit interventions relating to teaching and counseling, emotional support and attending care conferences concerning case and care management.

When using a high-calibration approach, a high number of deviant cases was observed for causal paths in almost every truth table. This may reflect on the one side, the complexity of missed care as a phenomenon per se. One the other hand, this may reflect the need to improve the theory-data fit within this analytical approach. Because of this discrepancy, in the further steps of data analysis, we focused solely on the configurations of reasons triggering the outcomes of missed care using the low-calibration approach.

### Necessity analysis

During necessity analysis we did not observe necessary 2^nd^-level conditions that fulfilled the previously defined goodness-of-fit cutoff values. In other words, for the cases in our dataset, there were no robust contextual factors that were always present, either singularly or in combination, when nurses reported that reasons for missed care were present in their care environment.

In contrast, and regarding configurations of 1st-level conditions triggering missed care outcomes, we found that a *high demand for patient care* was a necessary condition for the outcomes ***SA14.b****: Counseling and training patients for discharge* (inclN = 0.902; RoN = 0.650; covN = 0.831) and ***SA16:*** *Performing comprehensive assessment of the patient’s condition* (inclN = 0.901; RoN = 0.562; covN = 0.754). Multiple configurations of 1^st^-level conditions—mainly consisting of logical disjunctions (OR)—were also identified as necessary for 14 outcomes. They are presented in Table 3.

**Table 3:** Necessary causal paths: solutions for 1^st^-level conditions and missed care

ID	**Missed Care Outcomes**	SolNr	**Necessary Reasons for Missed Care**				***Goodness-of-fit***		
			***Demand***	***Labor***	***Material***	***RelComm***	***inclN***	***RoN***	***covN***
SA1	**Ambulation/mobilization as frequently as necessary/as ordered**	1	●	●			0.937	0.581	0.826
		2	●		●		0.903	0.635	0.831
		3	●			●	0.907	0.632	0.831
SA8	**Full documentation of all necessary nursing relevant data**	1	●	●			0.931	0.535	0.791
		2	●			●	0.901	0.585	0.795
SA9a	**Patient teaching about illness and planed care**	2	●	●			0.916	0.679	0.886
SA9b	**Informal caregiver teaching about illness and planed care**	2	●	●			0.918	0.682	0.888
SA10a	**Emotional support to patient**	2	●	●			0.913	0.669	0.881
SA10b	**Emotional support to informal caregivers**	2	●	●			0.907	0.756	0.922
SA14b	**Counseling and training patients for discharge**	1	●				0.902	0.650	0.831
SA14c	**Counseling and training informal caregivers for discharge**	2	●	●			0.933	0.627	0.856
		3	●			●	0.903	0.675	0.861
SA16	**Performing comprehensive assessment of the patient’s condition**	1	●				0.901	0.562	0.754
SA17	**Focused reassessments according to patient condition**	1	●	●			0.933	0.547	0.801
		2	●		●		0.900	0.602	0.806
		3	●			●	0.907	0.602	0.809
SA19	**Timely responding to patient call lights**	1	●	●			0.935	0.527	0.784
		2	●		●		0.902	0.581	0.788
		3	●			●	0.906	0.578	0.789
SA21	**Assess effectiveness of PRN medications**	1	●	●			0.937	0.546	0.800
		2	●		●		0.904	0.600	0.804
		3	●			●	0.910	0.600	0.807
SA22	**Attend interdisciplinary care conferences**	2	●	●			0.926	0.630	0.858
		4	●		●	●	0.901	0.663	0.859
SA25	**Adequate surveillance of confused/impaired patients**	2	●	●			0.934	0.604	0.842
		3	●		●		0.901	0.659	0.848
		4	●			●	0.906	0.658	0.849

ID: variable short name); SolNr: solution number in overall model of necessity analysis; Demand: high demand for patient care; Labor: poor allocation of labor resources; Material: poor allocation of material resources; RelComm: difficulties in relationships and communication; inclN: consistency score for necessary; RoN: relevance of necessity; covN: coverage score for necessary

● necessary condition (logical disjunctions are presented)

Necessary 1^st^-level conditions consistently included *poor labor resources*, *high demand for patient care,* or both. The conditions *difficulties in relationships and communication* and *poor material resources* were present in single necessary configurations; nonetheless, they occurred inconsistently, both in their present and negated form.

### Boolean minimization and sufficiency analysis

Regarding the association between contextual factors and the reasons for missed care, we observed that truth table minimization was only possible for the reasons of *high demand for patient care* and *poor labor resources*, generating in both cases higher-order solutions with high sufficiency consistency values (inclS) but low coverage values (covS). These results possibly mean that contextual factors are not consistently relevant as causal mechanisms for the emergence of reasons of missed care. Therefore we only considered conservative solutions and concluded data analysis with this step.

Regarding the association of the reasons for missed care on the outcomes of missed care, we considered conservative, intermediate and parsimonious solutions. Due to the presence of a significant number of logical remainders, parsimonious solutions seemed too broad for the aim of our study, given our strong theoretical background. Therefore, to consider theoretical expectations during data analysis and interpretation, we focused on intermediate solutions. Intermediate solutions considered the directional expectation that a high demand for patient care AND a poor allocation of labor resources, OR a poor allocation of resources, OR difficulties in relationships and communication lead to missed care (Demandm * Laborm + Materialm + RelCommm). Conservative solutions were considered in the absence of logical remainders. For a frequency threshold of 15 cases, we found solutions for 20 of the 30 defined missed care outcomes. Predefined goodness of fit values were met for 10 of the 20 missed care outcomes with solutions, providing evidence to defend the robustness of the results (Table 4).

**Table 4:**
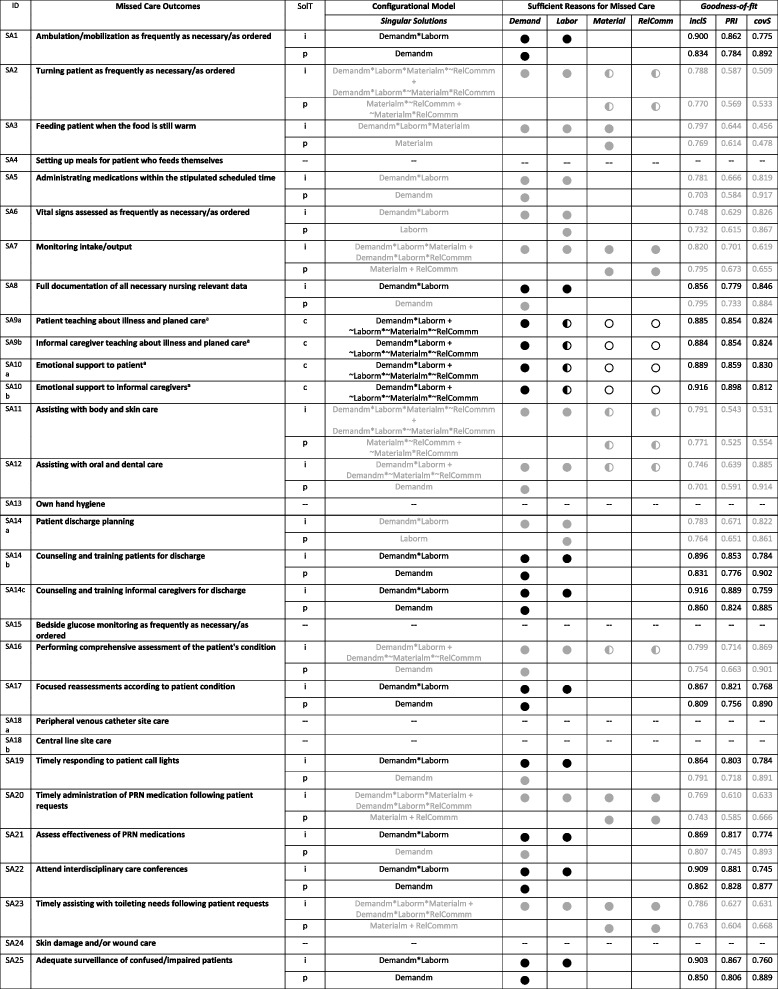
Sufficient causal paths: model solutions for 1^st^-level conditions and missed care (n.cut = 15)

ID: variable short name

SolT: solution type; i: intermediate solution; c: conservative solution; p: parsimonious solution

Conditions: Demand: high demand for patient care; Labor: poor allocation of labor resources; Material: poor allocation of material resources; RelComm: difficulties in relationships and communication

Logical operators: AND: "*" (i.e., logical conjunction between conditions); OR: “ + ” (i.e., logical disjunction between conditions); NOT: “ ~ ” (i.e., logical conjunction between conditions)

Solutions: ● sufficient condition; ○: negated sufficient condition ( ~). ◐: condition sufficient as both present and negated condition in different single solutions of the model

Goodness-of-fit: inclS: consistency score for sufficiency; PRI: proportional reduction in inconsistency; covS: coverage score for sufficiency; both more restrictive (in black [inclS > 0.8, PRI > 0.75, covS > 0.75]) and less restrictive results (in gray) are shown; symbols in gray carry the same meaning as symbols in black but do so for a model that did not fulfil goodness-of-fit thresholds

^a^Only conservative solutions were generated

### Understanding the results

For each of the 1^st^-level conditions, we conducted necessity analysis and calculated a total of 9 configurational models of contextual factors using different calibration approaches. For each of the missed care outcomes, we conducted necessity analysis and calculated a total of 18 configurational models, with different calibration and frequency threshold values. The complete results regarding necessity and sufficiency analysis, as well as minimization, can be found in Additional file 2. To summarize the findings according to the aim of our study, we elaborated the two configuration charts previously presented: the first chart presents the identified necessary causal paths (Table 3); the second chart presents the results of the Boolean minimization and the identified sufficient causal paths (Table 4). We did not present individual solutions within the single models in Table 4 but rather focused on the overview of configurations of conditions that emerged in our data. Our aim was to show if a particular condition was consistently present or absent in configurations associated with the occurrence of different missed care interventions.

In the following paragraphs, we will explain the procedure that led to our configuration charts, using an example focusing on the missed intervention *SA1 Ambulation/mobilization as frequently as necessary or as ordered*, to facilitate the understanding of the results. We used Venn diagrams to think about, discuss and interpret the configurations of conditions in our example. Nonetheless, one has to point out that this form of graphical representation is not reliable for representing fuzzy set-based configurations and can be misleading because the depicted proportions do not directly reflect coverage or inclusion quantities [57].

### Necessity analysis

**Fig. 3:**
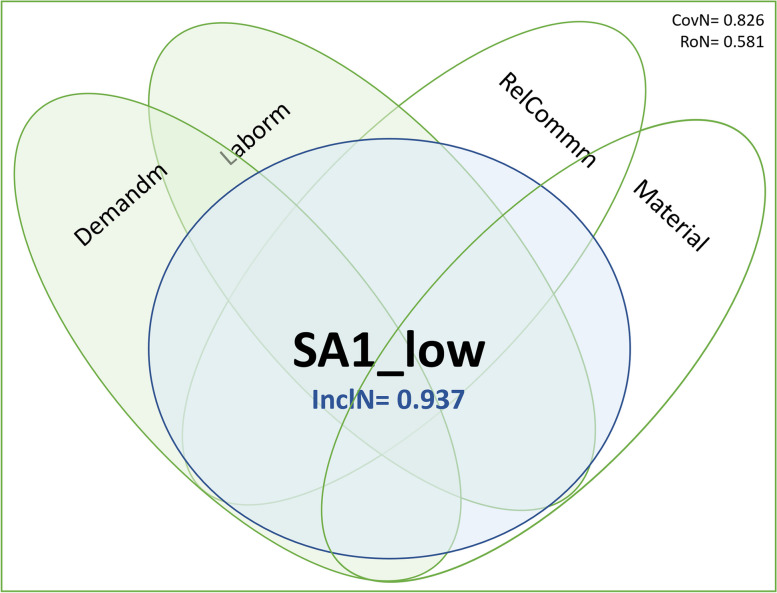
Necessity Analysis S1. Legend: Blue circle: Missed care outcome is present; Green Circle: Reasons for missed care are present

For the outcome ***SA1:****Ambulation/mobilization as frequently as necessary or as ordered* with a low-calibration approach (*SA1_low*) and considering the reasons for missed care with a medium-calibration approach (*Demandm, Laborm, Materialm* and *RelCommm*) as an example, a necessity analysis with the superSubset() function showed the following results:
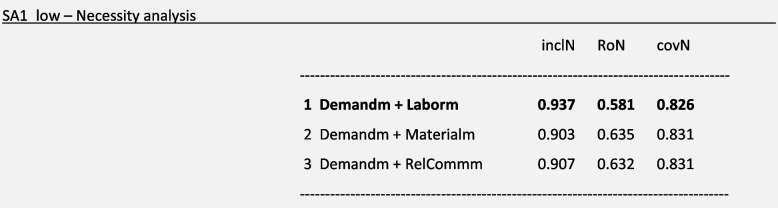


Considering these results, we will focus on solution 1 of the necessity analysis (**in bold**) to explain our example. We regarded *Demandm* and *Laborm* as present conditions in the disjunction necessary to trigger the outcome SA1. This solution means, from our perspective, that either the reason *Demandm* or the reason *Laborm* are necessary; i.e., one or the other are always present when the outcome SA1 occurs. Figure 3 presents a graphical depiction of solution 1 to facilitate interpretation:

In other words, when the intervention ***SA1****: Ambulation/mobilization as frequently as necessary or as ordered* was missed in direct patient care, in most of the cases (inclN = 0.94), either there was a poor allocation of labor resources or the demand for patient care was high, or both were true. This particular causal path covered 0.83 (covN) of the outcome. The necessity analysis of the negation of this expression with “ ~ Laborm* ~ Demandm ⇐ SA1_low” using the function pof() confirmed this interpretation (inclN = 0.199; RoN = 0.939; covN = 0.729). For a high-calibration approach, necessity analysis did not show any results fulfilling our thresholds (SA1_high).

As mentioned above, the necessity analysis revealed several disjunctions as being necessary for 14 missed care outcomes. Most of these involved two or three conditions and singular negations of reasons for missed care, which made the interpretation of the results more difficult, as acknowledged by Dușa [57]. Because of this complexity, we only focused on theoretically relevant results in our configurational chart [57]. Detailed results can be found individually in Additional file 2.

### Boolean minimization and sufficiency analysis

For the outcome ***SA1:*** *Ambulation/mobilization as frequently as necessary or as ordered* and considering a frequency threshold *n* = 15 for causal paths, we obtained the following truth table and the following solutions for a low-calibration approach using conservative, intermediate and parsimonious solutions:
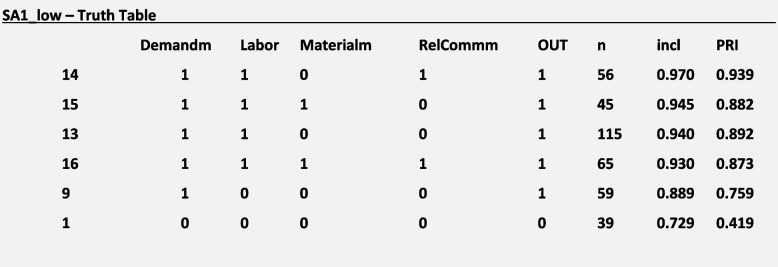



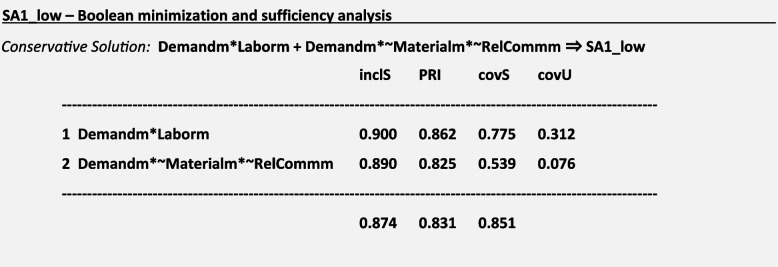



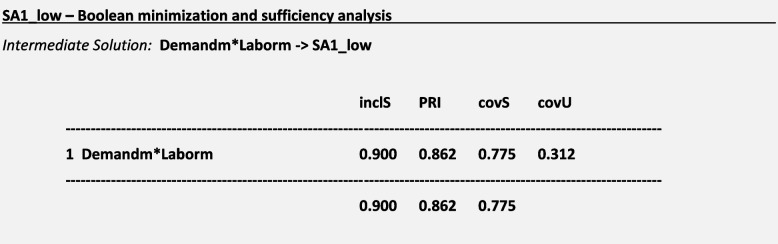



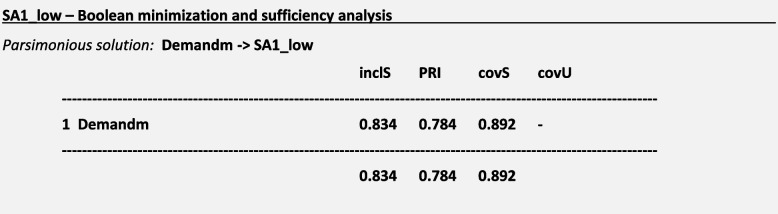


**Fig. 4:**
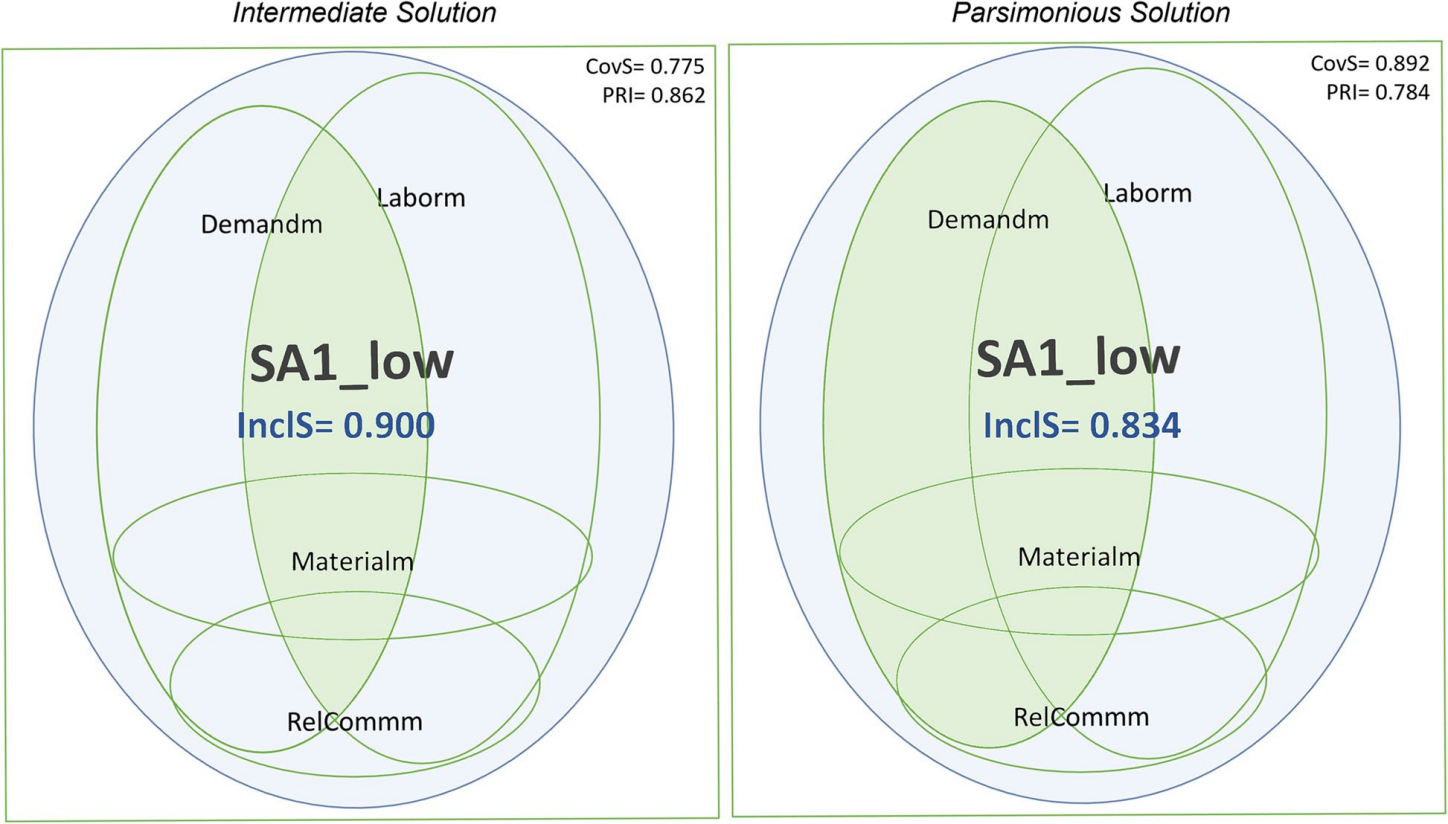
Sufficiency Analysis S1. Legend: Blue circle: Missed care outcome is present; Green Circle: Reasons for missed care are present

We interpreted the intermediate and parsimonious solutions regarding SA1_low graphically as follows (Fig. 4):

For a missed ***SA1:*** *Ambulation/mobilization as frequently as necessary or as ordered*, sufficient causal paths mostly included the presence of a high demand for patient care and a poor allocation of labor resources. According to the results of Boolean minimization, we considered Demandm and Laborm as sufficient conditions for triggering the outcome SA1 in the intermediate solution. Demandm was considered a sufficient condition for triggering the outcome SA1 in the parsimonious solution.

### Asymmetry analysis

Asymmetry analysis between reasons for missed care and missed care interventions showed that causal paths with higher sufficiency and consistency scores were those that reflected the absence of the reasons for missed care leading to the absence of missed care. Furthermore, upon conducting Boolean minimization, we could not observe any intermediate solutions (for n.cut = 15) with high goodness-of-fit values that contradicted our previous findings.

## Discussion

Our study aimed to reveal logical configurations of contextual factors and reasons for missed care associated with the presence of different missed care interventions in Austrian hospitals, following an comprehensive and theory-grounded qualitative comparative analysis approach. To achieve this aim, we proceeded according to the theoretical Missed Nursing Care Model by Kalisch et al. [12] and our understanding of what it means to observe a contextual factor, the reasons for missed care and the different missed care interventions as a *present condition or outcome.* This approach was made possible by applying different calibration strategies on a dataset consisting of quantitative data, which was generated with a validated instrument for assessing missed care [14, 21]. Here, we found that a more sensitive approach to calibrating outcomes relating to missed care is the most revealing strategy for uncovering configurations of reasons leading to missed care. We showed that the occurrence of missed care is associated with a high demand for patient care and with a lack of labor resources, frequently in combination with difficulties in relationships and communication and poor allocation of material resources. More importantly, we revealed that different missed care interventions may be associated with different causal configurations in the setting of our study.

We observed that certain reasons for missed care, namely, *poor allocation of labor resources* and/or *high demand for patient care*, were present in most logical causal paths leading to the different types of care that is missed (i.e., are necessary and possibly trivial conditions for missed care); the minimized configurational models rather showed that these reasons seemed sufficient for most missed care interventions to occur, either alone or in combination with factors related to *difficulties in relationships and communication* and *poor allocation of material resources*. These findings are in accordance with systematic reviews [6, 11, 28], which have pointed out that the most important reason why nursing care is currently being missed is the lack of adequate labor resources in the face of the high complexity and intensity levels of nursing care.

Interestingly, different missed care interventions were found to be associated with different causal paths of reasons. Considering these different configurations, it was clear that the type of missed care interventions, particularly those relating to ambulation, administrating scheduled medication and assessing vital signs, teaching about illness, emotional support, counseling and training, adequate surveillance of cognitive impaired patients, timely responding to patient call lights, and discharge planning or attending interdisciplinary care conferences, were clearly associated with the presence of *poor allocation of labor resources* and/or *high demand for patient care*. Other missed care interventions, e.g., those related to turning (i.e., repositioning patients in bed), body and oral care, feeding and monitoring intake/output, timely administration of PRN medication management or assisting with toileting needs, were associated with the presence of reasons for missed care in our data, including the *poor allocation of material resources* and *difficulties in relationships and communication*. Other omissions of nursing care, mainly those regarding medical interventions, such as bedside glucose monitoring, wound care or catheter site care, did not seem to occur in association with the presence of reasons for missed care, begging the question of whether the causal mechanisms underlying this type of error may differ from those depicted in Kalisch et al. [12], which otherwise seem to apply for most of the surveyed missed care interventions.

We observed that contextual factors, although consistently present in single logical causal paths associated with the presence of *high demand for patient care* and *poor labor resources*, were not relevant to explaining the regularities in the presence or absence of the reasons for missed care in the care environment in general units in Austrian hospitals. We expected to see a consistent association between contextual factors and reasons for missed care across the different cases in our sample, especially regarding a possible association between the adequacy of nursing staffing and *poor labor resources* and *difficulties in relationships and communication* or unit and hospital size and *high demand for patient care*. This was, however, not the case.

Kalisch et al. [63] have already shown in their studies regarding the variation of missed care according to nurse, hospital, and unit characteristics that the direct influence of these factors on the frequency of missed care seems weak (e.g., only 16% of the variance of missed care could be explained through these factors). In a further study, these authors identified that “unit size affects not only the team size but also the physical distance, communication, and diversity of the nursing team” (p. 221) [64]. Additionally, they pointed out that while “the actual number of nursing staff members as a whole was not significant to predicting nursing unit teamwork, the amount of unlicensed personnel was” (p. 222) [64]. Studies on the preventive measures of missed care have frequently pointed out that increasing the qualification and experience of nursing staff and redesigning hospitals and their units seems to play a role in missed care [6, 65, 66].

It is possible that the difference between our findings and those of Kalisch et al. [64] is due to our methodical approach – whereas Kalisch et al. [64] considered the direct impact of contextual factors on the frequency of missed nursing care interventions, our approach is an indirect one, focusing on the impact of contextual factors on the presence or absence of reasons for missed care using logical operators. In this sense, further studies investigating the impact of contextual factors on missed care should examine both a direct and indirect impact (i.e., mediated by reasons for missed care) to further the discussion of these different findings. Here, it seems relevant to rely on other measurement instruments focused on contextual factors, such as the adequacy of nursing staffing, and to test different calibration approaches.

Another possible explanation for the absence of configurations in our study regarding the association of contextual factors and reasons for missed care could be causal asymmetry within complex phenomena, as aspects previously associated with the occurrence of missed care do not seem to necessarily be associated with reasons for missed care. Further research regarding the role of contextual factors in the occurrence and prevention of missed care from the lens of complexity theory is, from this perspective, urgently needed.

Using a new analytical approach to explore configurations of contextual factors and reasons for missed care associated with different missed care interventions, our study clearly revealed the necessity of deepening the understanding of missed care as a complex phenomenon in nursing science. A further theorization of missed care seems necessary to clearly explain the context, the reasons and the mechanisms that lead to errors of omission in nursing care in more detail [1]. Furthermore, this theorization should offer researchers a clear orientation to decide, when pursuing a quantitative approach, if and when a particular reason for the outcome of missed care is present, guiding the attribution of meaning to numerical values in empirical quantitative data.

Most quantitative studies analyzing missed care have relied on central tendency measures to explore associations between influencing factors and the outcome of missed care as a whole, e.g., using mean score indices to depict the frequency of missed care across all types of care that is omitted [18]; our study confirms that this approach may conceal important differences in the manifestations of missed care and its reasons, which could be of paramount importance to understand this problem. Therefore, in further research on the reasons for missed care in nursing science in the future, the full scope of QCA as a valid approach to uncovering manifestations of complex causal mechanisms should be considered, expanding the possibilities of quantitative research, e.g., in the context of multiple case-studies or mixed-methods design studies. This approach would allow for analytical approaches that focus on individual case knowledge and detailed considerations regarding logical remainders and deviant cases, potentially producing new knowledge on the reasons for missed care and on the emergence of causal mechanisms regarding errors of omission in acute care nursing.

### Limitations

We conducted this study to deepen our understanding of the reasons for missed care in general units in Austrian hospitals and to expand the previous results of our study on exploring the theoretical antecedents of missed care using structural equation modeling based on linear regression analysis. In this sense, we implemented QCA as a complementary analytical approach to regression analysis. Regression analysis and QCA are based on very different epistemological considerations; nonetheless, both approaches can be used in the context of large-N studies to explore manifestations of possible causal mechanisms from complementary perspectives [24, 40]. Despite this recommendation, some authors are increasingly criticizing the utilization of QCA as an analytical tool for quantitative data generated in the context of cross-sectional survey research, defining this line of approach as “method stretching” and as an epistemological dissonance of sorts; this is because researchers who use this approach fail to go back to cases to refine data analysis, thus relaxing single-case knowledge and the dialog between analyzed data and empirical cases and consequently missing important aspects of the method, which would allow us to elaborate causal explanations [67]. This could be the case in our study, especially because we refrained from a detailed analysis of deviant cases and counterfactuals due to the great number of cases in our data. Rutten [54] recognized this problem and pleaded for the intersection of both empirical data and theoretical knowledge to allow for the elaboration of causal inference under scrutiny of the complexity and robustness of the results of QCA analysis. Furthermore, he stressed that researchers should be cautious when using QCA in large datasets because its results alone are not sufficient to elaborate causal explanations.

The main limitation of our paper lies in the fact that we implemented a QCA based on a quantitative reductionist approach to discuss the complexity of the reasons for missed care. Bearing this in mind, we refrained from elaborating causal explanations; instead, we focused on associations and configurations in our data. Approaching missed care as a complex nursing science phenomenon will require more from future researchers looking to QCA as a method; thus, another epistemological and methodological foundation for researching causality in missed care in nursing science will be needed.

## Conclusions

Missed care, as well as the various contextual factors and reasons that may contribute to its occurrence, are embedded in complex adaptive systems and need to be considered from the lens of complexity theory in nursing science. This was clear in our study, where e.g., contextual factors, although not showing consistent causal paths leading to reasons for missed care, provided important insights into the possible mechanisms that lead to missed care. In addition, poor labor resources and a high demand for patient care may be important reasons for missed care but were accompanied by configurational interactions with other reasons for missed care, reflecting possible cumulative and interacting causational mechanisms and making a case for equifinality regarding the reasons for missed care in Austrian general units in hospital settings.

Furthermore, not all missed care interventions seem to be triggered through the same configurations of reasons for missed care; e.g., missed interventions regarding emotional support to patients did not show the same causal paths as missing the assistance of patients with their oral and dental care in our study. This challenges the idea that the causal mechanisms of missed care can be researched using the outcome of missed care as a unidimensional construct based on central tendency measures. As stated before, to understand the complexity of missed care, it is necessary to turn to complexity theory. To do so, a theoretical framework that acknowledges that complex systems, such as missed care, are composed of multiple interacting components that can exhibit emergent behavior must be further developed. By examining missed care through the lens of complexity theory, researchers may be able to better understand the various factors that contribute to the occurrence of missed care and therefore deepen the knowledge on possible prevention strategies, thereby contributing to the mitigation of this challenge in health care systems worldwide.

### Supplementary Information

**Supplementary Material 1.**

**Supplementary Material 2.**

## Data Availability

The datasets generated and/or analyzed during the current study are available in the Zenodo repository: https://doi.org/10.5281/zenodo.10007599.
