# Supplementary material for: Missing nurses cause missed care: is that it? Non-trivial configurations of reasons associated with missed care in Austrian hospitals – a qualitative comparative analysis
Source: BMC Nurs. 2024 Apr 26;23:282. doi: 10.1186/s12912-024-01923-y (PMC11055368; doi:10.1186/s12912-024-01923-y)
Supplement: Supplementary file 1 — Supplementary Material 1. [file 12912_2024_1923_MOESM1_ESM.docx]

**Online-Supplement 1: *The MISSCARE-Austria Project***


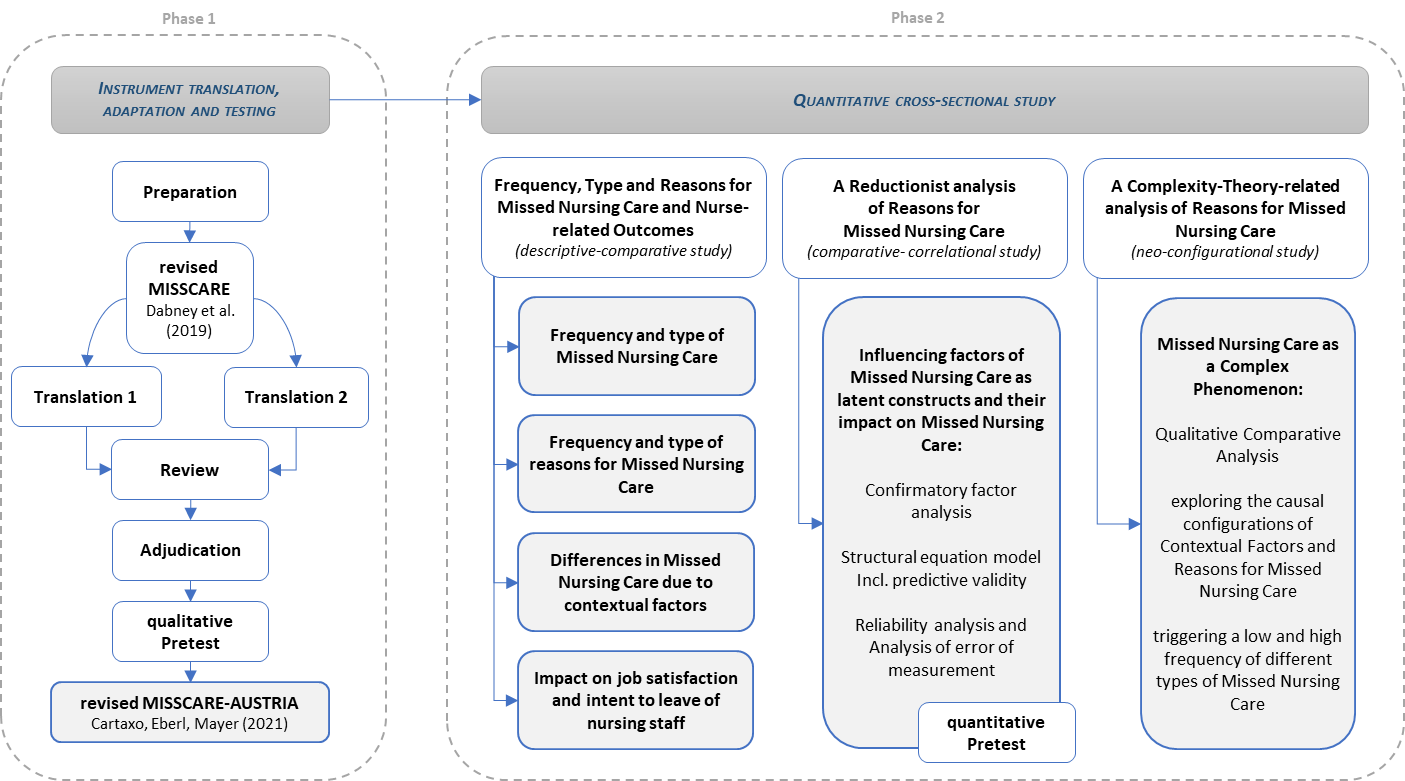


Cartaxo, A., Eberl, I., & Mayer, H. (2023). Übersetzung des revised MISSCARE Survey zum deutschsprachigen revised MISSCARE-Austria [Using the TRAPD method to translate the revised MISSCARE Survey from English into German: Revised MISSCARE-Austria]. Pflege, 10.1024/1012-5302/a000936. Advance online publication. <https://doi.org/10.1024/1012-5302/a000936>

Cartaxo, A., Eberl, I. & Mayer, H. (2022) Die MISSCARE-Austria-Studie – Teil I: Häufigkeit von Missed Nursing Care und assoziierten Einflussfaktoren auf Allgemeinstationen in österreichischen Krankenhäusern [Frequency of Missed Nursing Care and Associated Influencing Factors on General Wards in Austrian Hospitals]. HBScience 13 (Suppl 2), 30–42. <https://doi.org/10.1007/s16024-022-00387-x> *(in German)*

Cartaxo, A., Eberl, I., Mayer, H. (2022) Die MISSCARE Austria Studie – Teil II: Patient-to-Nurse Ratio, Angemessenheit der Pflegepersonalbesetzung und Einfluss auf Missed Nursing Care – eine quantitative Datenexploration auf Allgemeinstation in österreichischen Krankenhäusern [Patient-to-nurse ratio, adequacy of nurse staffing and influence on Missed Nursing Care - a quantitative data exploration on general wards in Austrian hospitals]. HBScience 13 (Suppl 2), 43–60 (2022). <https://doi.org/10.1007/s16024-022-00389-9> *(in German)*

Cartaxo, A., Eberl, I., Mayer, H. (2022) Die MISSCARE Austria Studie – Teil III: Missed Nursing Care auf Allgemeinstationen in österreichischen Krankenhäusern: Einfluss auf die Arbeitszufriedenheit und auf die Absicht, den Beruf zu verlassen [Missed Nursing Care on General Wards in Austrian Hospitals: Influence on job satisfaction and intention to leave]. HBScience 13 (Suppl 2), 61–78. <https://doi.org/10.1007/s16024-022-00390-2> *(in German)*

Cartaxo, A., Dabney, B. W., Mayer, H., Eberl, I., & Gonçalves, L. (2023). External influencing factors on missed care in Austrian hospitals: Testing the theoretical antecedents of missed care using structural equation modelling. Journal of Advanced Nursing, 00, 1– 16. <https://doi.org/10.1111/jan.15700>
