# Supplementary material for: Missing nurses cause missed care: is that it? Non-trivial configurations of reasons associated with missed care in Austrian hospitals – a qualitative comparative analysis
Source: BMC Nurs. 2024 Apr 26;23:282. doi: 10.1186/s12912-024-01923-y (PMC11055368; doi:10.1186/s12912-024-01923-y)
Supplement: Supplementary file 2 — Supplementary Material 2. [file 12912_2024_1923_MOESM2_ESM.docx]

Missing nurses cause missed care: is that it?

Non-Trivial Configurations of Reasons Associated with Missed Care in Austrian hospitals - A qualitative comparative analysis

**Online-Supplement 2:** R Script

(Output Documentation)

by

Ana Cartaxo, João Cartaxo, Johannes Bergmann

####################################################
### Data Processing 1/3 ###
####################################################

# Variables Description

# condsNurses: Nurses Characteristics (NExp [1=Nurse's experience < 5 years], NQual [1=registered nurse], Role [1=non-manager])
# condsU: Unit Characteristics (UT[1=medical units], US[1=30 patient-beds or more], UNS[1=Nurse Staffing adequate in the last 3 months not adequate])
# condsH: Hospital Characteristics (HT[1=public hospital], HS[1=500 patient-beds or more ], HL[1=urban])
# Demand: Demand for patient care (0: not decisive at all, 1: very decisive)
# Laborm: Labor resources allocation (0: not decisive at all, 1: very decisive)
# Materialm: Material resources allocation (0: not decisive at all, 1: very decisive)
# RelCommm: Relationship and communication factors (0: not decisive at all, 1: very decisive)
# _low: Low calibration approach ( 1 / 3.5 / 6 )
# m: Medium calibration approach ( 1.5 / 3.5 / 5.5 )
# _high: High calibration approach ( 2 / 3.5 / 5 )

#### Performing QCA: Truth Tables: 2nd Level -> Reasons MNC
# Setting Conditions: Defining the contextual factors regarding Nurses, Unit and Hospital Characteristics

# (According to Table 1)
condsNurses <- base::subset(c1QCA, select = c("NQual", "Role", "NExp")) # Nurses Characteristics
condsU <- base::subset(c1QCA, select = c("UT", "US", "UNS")) # Unit Characteristics
condsH <- base::subset(c1QCA, select = c("HT", "HS", "HL")) # Hospital Characteristics

# Setting Outcomes:create vector with reasons for MNC strings (According to Table 1)
outcome_reasons = c("Demandm_low", "Demandm", "Demandm_high",
 "Laborm_low", "Laborm", "Laborm_high",
 "Materialm_low", "Materialm", "Materialm_high",
 "RelCommm_low", "RelCommm", "RelCommm_high")

#### Main Results: print lists
# Result: Truth Table: Nurses Characteristics triggering Reasons for MNC (for n.cut = 3)

## $Demandm_low
##
## OUT: output value
## n: number of cases in configuration
## incl: sufficiency inclusion score
## PRI: proportional reduction in inconsistency
##
## NQual Role NExp OUT n incl PRI
## 4 0 1 1 0 9 0.798 0.747
## 8 1 1 1 0 96 0.762 0.709
## 3 0 1 0 0 22 0.756 0.699
## 7 1 1 0 0 200 0.724 0.653
## 5 1 0 0 0 71 0.714 0.653
## 6 1 0 1 0 3 0.660 0.550
##
## It seems that all output values have been coded to zero.
## Suggestion: lower the inclusion score for the presence of the outcome,
## the relevant argument is "incl.cut" which now has a value of 0.8.
##
##
## $Demandm
##
## OUT: output value
## n: number of cases in configuration
## incl: sufficiency inclusion score
## PRI: proportional reduction in inconsistency
##
## NQual Role NExp OUT n incl PRI
## 4 0 1 1 1 9 0.834 0.801
## 8 1 1 1 0 96 0.790 0.755
## 3 0 1 0 0 22 0.782 0.742
## 7 1 1 0 0 200 0.749 0.698
## 5 1 0 0 0 71 0.739 0.698
## 6 1 0 1 0 3 0.676 0.591
##
##
## $Demandm_high
##
## OUT: output value
## n: number of cases in configuration
## incl: sufficiency inclusion score
## PRI: proportional reduction in inconsistency
##
## NQual Role NExp OUT n incl PRI
## 4 0 1 1 1 9 0.872 0.853
## 8 1 1 1 1 96 0.819 0.798
## 3 0 1 0 1 22 0.808 0.783
## 7 1 1 0 0 200 0.776 0.743
## 5 1 0 0 0 71 0.767 0.743
## 6 1 0 1 0 3 0.690 0.631
##
##

## $Laborm_low
##
## OUT: output value
## n: number of cases in configuration
## incl: sufficiency inclusion score
## PRI: proportional reduction in inconsistency
##
## NQual Role NExp OUT n incl PRI
## 8 1 1 1 0 96 0.674 0.559
## 7 1 1 0 0 200 0.653 0.529
## 4 0 1 1 0 9 0.646 0.539
## 3 0 1 0 0 22 0.635 0.514
## 6 1 0 1 0 3 0.568 0.426
## 5 1 0 0 0 71 0.549 0.368
##
## It seems that all output values have been coded to zero.
## Suggestion: lower the inclusion score for the presence of the outcome,
## the relevant argument is "incl.cut" which now has a value of 0.8.
##
##
## $Laborm
##
## OUT: output value
## n: number of cases in configuration
## incl: sufficiency inclusion score
## PRI: proportional reduction in inconsistency
##
## NQual Role NExp OUT n incl PRI
## 8 1 1 1 0 96 0.697 0.611
## 7 1 1 0 0 200 0.674 0.578
## 4 0 1 1 0 9 0.670 0.593
## 3 0 1 0 0 22 0.656 0.565
## 6 1 0 1 0 3 0.579 0.471
## 5 1 0 0 0 71 0.559 0.409
##
## It seems that all output values have been coded to zero.
## Suggestion: lower the inclusion score for the presence of the outcome,
## the relevant argument is "incl.cut" which now has a value of 0.8.
##
## $Laborm_high
##
## OUT: output value
## n: number of cases in configuration
## incl: sufficiency inclusion score
## PRI: proportional reduction in inconsistency
##
## NQual Role NExp OUT n incl PRI
## 8 1 1 1 0 96 0.724 0.664
## 4 0 1 1 0 9 0.700 0.652
## 7 1 1 0 0 200 0.698 0.631
## 3 0 1 0 0 22 0.682 0.622
## 6 1 0 1 0 3 0.593 0.521
## 5 1 0 0 0 71 0.573 0.458
##
## It seems that all output values have been coded to zero.
## Suggestion: lower the inclusion score for the presence of the outcome,
## the relevant argument is "incl.cut" which now has a value of 0.8.
## $Materialm_low
##
## OUT: output value
## n: number of cases in configuration
## incl: sufficiency inclusion score
## PRI: proportional reduction in inconsistency
##
## NQual Role NExp OUT n incl PRI
## 6 1 0 1 0 3 0.484 0.227
## 8 1 1 1 0 96 0.408 0.230
## 4 0 1 1 0 9 0.405 0.276
## 3 0 1 0 0 22 0.404 0.225
## 7 1 1 0 0 200 0.316 0.154
## 5 1 0 0 0 71 0.266 0.099
##
## It seems that all output values have been coded to zero.
## Suggestion: lower the inclusion score for the presence of the outcome,
## the relevant argument is "incl.cut" which now has a value of 0.8.
##
## $Materialm
##
## OUT: output value
## n: number of cases in configuration
## incl: sufficiency inclusion score
## PRI: proportional reduction in inconsistency
##
## NQual Role NExp OUT n incl PRI
## 6 1 0 1 0 3 0.480 0.259
## 3 0 1 0 0 22 0.402 0.259
## 4 0 1 1 0 9 0.400 0.298
## 8 1 1 1 0 96 0.399 0.255
## 7 1 1 0 0 200 0.299 0.169
## 5 1 0 0 0 71 0.242 0.109
##
## It seems that all output values have been coded to zero.
## Suggestion: lower the inclusion score for the presence of the outcome,
## the relevant argument is "incl.cut" which now has a value of 0.8.
##
## $Materialm_high
##
## OUT: output value
## n: number of cases in configuration
## incl: sufficiency inclusion score
## PRI: proportional reduction in inconsistency
##
## NQual Role NExp OUT n incl PRI
## 6 1 0 1 0 3 0.473 0.299
## 3 0 1 0 0 22 0.407 0.304
## 4 0 1 1 0 9 0.395 0.320
## 8 1 1 1 0 96 0.391 0.284
## 7 1 1 0 0 200 0.283 0.188
## 5 1 0 0 0 71 0.217 0.121
##
## It seems that all output values have been coded to zero.
## Suggestion: lower the inclusion score for the presence of the outcome,
## the relevant argument is "incl.cut" which now has a value of 0.8.
##
## $RelCommm_low
##
## OUT: output value
## n: number of cases in configuration
## incl: sufficiency inclusion score
## PRI: proportional reduction in inconsistency
##
## NQual Role NExp OUT n incl PRI
## 6 1 0 1 0 3 0.514 0.318
## 8 1 1 1 0 96 0.413 0.190
## 7 1 1 0 0 200 0.410 0.195
## 3 0 1 0 0 22 0.389 0.155
## 5 1 0 0 0 71 0.334 0.109
## 4 0 1 1 0 9 0.240 0.000
##
## It seems that all output values have been coded to zero.
## Suggestion: lower the inclusion score for the presence of the outcome,
## the relevant argument is "incl.cut" which now has a value of 0.8.
##
## $RelCommm
##
## OUT: output value
## n: number of cases in configuration
## incl: sufficiency inclusion score
## PRI: proportional reduction in inconsistency
##
## NQual Role NExp OUT n incl PRI
## 6 1 0 1 0 3 0.522 0.365
## 8 1 1 1 0 96 0.401 0.214
## 7 1 1 0 0 200 0.397 0.216
## 3 0 1 0 0 22 0.378 0.177
## 5 1 0 0 0 71 0.313 0.122
## 4 0 1 1 0 9 0.203 0.000
##
## It seems that all output values have been coded to zero.
## Suggestion: lower the inclusion score for the presence of the outcome,
## the relevant argument is "incl.cut" which now has a value of 0.8.
##
## $RelCommm_high
##
## OUT: output value
## n: number of cases in configuration
## incl: sufficiency inclusion score
## PRI: proportional reduction in inconsistency
##
## NQual Role NExp OUT n incl PRI
## 6 1 0 1 0 3 0.537 0.428
## 8 1 1 1 0 96 0.388 0.242
## 7 1 1 0 0 200 0.381 0.241
## 3 0 1 0 0 22 0.366 0.204
## 5 1 0 0 0 71 0.289 0.139
## 4 0 1 1 0 9 0.157 0.000
##
## It seems that all output values have been coded to zero.
## Suggestion: lower the inclusion score for the presence of the outcome,
## the relevant argument is "incl.cut" which now has a value of 0.8.

# Result: Truth Table: Unit Characteristics triggering Reasons for MNC (for n.cut = 3)

## $Demandm_low
##
## OUT: output value
## n: number of cases in configuration
## incl: sufficiency inclusion score
## PRI: proportional reduction in inconsistency
##
## UNS US UT OUT n incl PRI
## 8 1 1 1 1 66 0.805 0.760
## 7 1 1 0 0 73 0.795 0.745
## 5 1 0 0 0 67 0.794 0.755
## 4 0 1 1 0 20 0.739 0.680
## 6 1 0 1 0 81 0.710 0.632
## 1 0 0 0 0 28 0.706 0.639
## 3 0 1 0 0 23 0.591 0.475
## 2 0 0 1 0 43 0.572 0.453
##
##
## $Demandm
##
## OUT: output value
## n: number of cases in configuration
## incl: sufficiency inclusion score
## PRI: proportional reduction in inconsistency
##
## UNS US UT OUT n incl PRI
## 8 1 1 1 1 66 0.838 0.809
## 7 1 1 0 1 73 0.830 0.797
## 5 1 0 0 1 67 0.824 0.799
## 4 0 1 1 0 20 0.761 0.720
## 6 1 0 1 0 81 0.735 0.679
## 1 0 0 0 0 28 0.730 0.684
## 3 0 1 0 0 23 0.600 0.510
## 2 0 0 1 0 43 0.580 0.486
##
##
## $Demandm_high
##
## OUT: output value
## n: number of cases in configuration
## incl: sufficiency inclusion score
## PRI: proportional reduction in inconsistency
##
## UNS US UT OUT n incl PRI
## 8 1 1 1 1 66 0.872 0.856
## 7 1 1 0 1 73 0.867 0.850
## 5 1 0 0 1 67 0.853 0.839
## 4 0 1 1 0 20 0.784 0.758
## 6 1 0 1 0 81 0.763 0.726
## 1 0 0 0 0 28 0.756 0.728
## 3 0 1 0 0 23 0.609 0.545
## 2 0 0 1 0 43 0.589 0.521
##
##
## $Laborm_low
## OUT: output value
## n: number of cases in configuration
## incl: sufficiency inclusion score
## PRI: proportional reduction in inconsistency
##
## UNS US UT OUT n incl PRI
## 5 1 0 0 0 67 0.738 0.655
## 7 1 1 0 0 73 0.716 0.621
## 8 1 1 1 0 66 0.680 0.564
## 6 1 0 1 0 81 0.672 0.546
## 4 0 1 1 0 20 0.514 0.272
## 1 0 0 0 0 28 0.494 0.298
## 3 0 1 0 0 23 0.469 0.259
## 2 0 0 1 0 43 0.459 0.279
##
##
## $Laborm
## OUT: output value
## n: number of cases in configuration
## incl: sufficiency inclusion score
## PRI: proportional reduction in inconsistency
##
## UNS US UT OUT n incl PRI
## 5 1 0 0 0 67 0.770 0.711
## 7 1 1 0 0 73 0.748 0.681
## 8 1 1 1 0 66 0.706 0.618
## 6 1 0 1 0 81 0.697 0.600
## 4 0 1 1 0 20 0.517 0.304
## 1 0 0 0 0 28 0.492 0.328
## 3 0 1 0 0 23 0.466 0.291
## 2 0 0 1 0 43 0.453 0.306
##
## It seems that all output values have been coded to zero.
## Suggestion: lower the inclusion score for the presence of the outcome,
## the relevant argument is "incl.cut" which now has a value of 0.8.
##
## $Laborm_high
## OUT: output value
## n: number of cases in configuration
## incl: sufficiency inclusion score
## PRI: proportional reduction in inconsistency
##
## UNS US UT OUT n incl PRI
## 5 1 0 0 1 67 0.806 0.769
## 7 1 1 0 0 73 0.787 0.745
## 8 1 1 1 0 66 0.736 0.677
## 6 1 0 1 0 81 0.726 0.658
## 4 0 1 1 0 20 0.522 0.346
## 1 0 0 0 0 28 0.489 0.362
## 3 0 1 0 0 23 0.464 0.331
## 2 0 0 1 0 43 0.447 0.337
##
##

## $Materialm_low
## OUT: output value
## n: number of cases in configuration
## incl: sufficiency inclusion score
## PRI: proportional reduction in inconsistency
##
## UNS US UT OUT n incl PRI
## 7 1 1 0 0 73 0.381 0.189
## 8 1 1 1 0 66 0.369 0.199
## 6 1 0 1 0 81 0.349 0.181
## 5 1 0 0 0 67 0.333 0.182
## 1 0 0 0 0 28 0.332 0.147
## 2 0 0 1 0 43 0.279 0.119
## 3 0 1 0 0 23 0.263 0.107
## 4 0 1 1 0 20 0.257 0.121
##
##
## $Materialm
## OUT: output value
## n: number of cases in configuration
## incl: sufficiency inclusion score
## PRI: proportional reduction in inconsistency
##
## UNS US UT OUT n incl PRI
## 7 1 1 0 0 73 0.370 0.211
## 8 1 1 1 0 66 0.357 0.218
## 6 1 0 1 0 81 0.336 0.200
## 5 1 0 0 0 67 0.318 0.201
## 1 0 0 0 0 28 0.312 0.163
## 2 0 0 1 0 43 0.259 0.134
## 3 0 1 0 0 23 0.240 0.115
## 4 0 1 1 0 20 0.230 0.131
##
##
## $Materialm_high
## OUT: output value
## n: number of cases in configuration
## incl: sufficiency inclusion score
## PRI: proportional reduction in inconsistency
##
## UNS US UT OUT n incl PRI
## 7 1 1 0 0 73 0.360 0.239
## 8 1 1 1 0 66 0.346 0.242
## 6 1 0 1 0 81 0.324 0.223
## 5 1 0 0 0 67 0.305 0.225
## 1 0 0 0 0 28 0.291 0.182
## 2 0 0 1 0 43 0.241 0.152
## 3 0 1 0 0 23 0.216 0.123
## 4 0 1 1 0 20 0.204 0.142
##
## It seems that all output values have been coded to zero.
## Suggestion: lower the inclusion score for the presence of the outcome,
## the relevant argument is "incl.cut" which now has a value of 0.8.
##
##

## $RelCommm_low
##
## OUT: output value
## n: number of cases in configuration
## incl: sufficiency inclusion score
## PRI: proportional reduction in inconsistency
##
## UNS US UT OUT n incl PRI
## 5 1 0 0 0 67 0.458 0.253
## 7 1 1 0 0 73 0.437 0.205
## 6 1 0 1 0 81 0.417 0.167
## 8 1 1 1 0 66 0.414 0.183
## 1 0 0 0 0 28 0.326 0.141
## 3 0 1 0 0 23 0.295 0.095
## 2 0 0 1 0 43 0.293 0.113
## 4 0 1 1 0 20 0.271 0.069
##
## $RelCommm
##
## OUT: output value
## n: number of cases in configuration
## incl: sufficiency inclusion score
## PRI: proportional reduction in inconsistency
##
## UNS US UT OUT n incl PRI
## 5 1 0 0 0 67 0.451 0.280
## 7 1 1 0 0 73 0.427 0.229
## 6 1 0 1 0 81 0.405 0.188
## 8 1 1 1 0 66 0.401 0.206
## 1 0 0 0 0 28 0.309 0.157
## 2 0 0 1 0 43 0.270 0.127
## 3 0 1 0 0 23 0.268 0.106
## 4 0 1 1 0 20 0.236 0.073
##
## $RelCommm_high
##
## OUT: output value
## n: number of cases in configuration
## incl: sufficiency inclusion score
## PRI: proportional reduction in inconsistency
##
## UNS US UT OUT n incl PRI
## 5 1 0 0 0 67 0.442 0.311
## 7 1 1 0 0 73 0.416 0.256
## 6 1 0 1 0 81 0.390 0.214
## 8 1 1 1 0 66 0.386 0.233
## 1 0 0 0 0 28 0.291 0.177
## 2 0 0 1 0 43 0.246 0.143
## 3 0 1 0 0 23 0.239 0.120
## 4 0 1 1 0 20 0.193 0.076
##
## It seems that all output values have been coded to zero.
## Suggestion: lower the inclusion score for the presence of the outcome,
## the relevant argument is "incl.cut" which now has a value of 0.8.

# Result: Truth Table: Hospital Characteristics triggering Reasons for MNC (for n.cut = 3)

## $Demandm_low
##
## OUT: output value
## n: number of cases in configuration
## incl: sufficiency inclusion score
## PRI: proportional reduction in inconsistency
##
## HL HS HT OUT n incl PRI
## 1 0 0 0 1 7 0.868 0.848
## 6 1 0 1 0 54 0.775 0.726
## 7 1 1 0 0 39 0.754 0.707
## 8 1 1 1 0 116 0.736 0.670
## 2 0 0 1 0 82 0.718 0.645
## 5 1 0 0 0 83 0.713 0.636
## 3 0 1 0 0 5 0.712 0.656
## 4 0 1 1 0 15 0.681 0.616
##
##
## $Demandm
##
## OUT: output value
## n: number of cases in configuration
## incl: sufficiency inclusion score
## PRI: proportional reduction in inconsistency
##
## HL HS HT OUT n incl PRI
## 1 0 0 0 1 7 0.907 0.897
## 6 1 0 1 1 54 0.802 0.768
## 7 1 1 0 0 39 0.781 0.751
## 8 1 1 1 0 116 0.763 0.717
## 2 0 0 1 0 82 0.742 0.689
## 3 0 1 0 0 5 0.740 0.706
## 5 1 0 0 0 83 0.739 0.684
## 4 0 1 1 0 15 0.702 0.656
##
##
## $Demandm_high
##
## OUT: output value
## n: number of cases in configuration
## incl: sufficiency inclusion score
## PRI: proportional reduction in inconsistency
##
## HL HS HT OUT n incl PRI
## 1 0 0 0 1 7 0.947 0.944
## 6 1 0 1 1 54 0.828 0.806
## 7 1 1 0 1 39 0.808 0.791
## 8 1 1 1 0 116 0.792 0.763
## 3 0 1 0 0 5 0.769 0.753
## 2 0 0 1 0 82 0.768 0.734
## 5 1 0 0 0 83 0.767 0.731
## 4 0 1 1 0 15 0.723 0.693
##
##
## $Laborm_low
##
## OUT: output value
## n: number of cases in configuration
## incl: sufficiency inclusion score
## PRI: proportional reduction in inconsistency
##
## HL HS HT OUT n incl PRI
## 1 0 0 0 0 7 0.718 0.627
## 6 1 0 1 0 54 0.672 0.571
## 4 0 1 1 0 15 0.666 0.559
## 3 0 1 0 0 5 0.665 0.590
## 2 0 0 1 0 82 0.646 0.524
## 8 1 1 1 0 116 0.638 0.496
## 7 1 1 0 0 39 0.619 0.488
## 5 1 0 0 0 83 0.602 0.444
##
##
## $Laborm
##
## OUT: output value
## n: number of cases in configuration
## incl: sufficiency inclusion score
## PRI: proportional reduction in inconsistency
##
## HL HS HT OUT n incl PRI
## 1 0 0 0 0 7 0.746 0.679
## 6 1 0 1 0 54 0.693 0.616
## 3 0 1 0 0 5 0.692 0.643
## 4 0 1 1 0 15 0.690 0.610
## 2 0 0 1 0 82 0.667 0.574
## 8 1 1 1 0 116 0.657 0.546
## 7 1 1 0 0 39 0.638 0.536
## 5 1 0 0 0 83 0.618 0.492
##
##
## $Laborm_high
##
## OUT: output value
## n: number of cases in configuration
## incl: sufficiency inclusion score
## PRI: proportional reduction in inconsistency
##
## HL HS HT OUT n incl PRI
## 1 0 0 0 0 7 0.775 0.730
## 3 0 1 0 0 5 0.728 0.702
## 4 0 1 1 0 15 0.719 0.664
## 6 1 0 1 0 54 0.714 0.661
## 2 0 0 1 0 82 0.691 0.627
## 8 1 1 1 0 116 0.680 0.600
## 7 1 1 0 0 39 0.664 0.592
## 5 1 0 0 0 83 0.639 0.545
##
## It seems that all output values have been coded to zero.
## Suggestion: lower the inclusion score for the presence of the outcome,
## the relevant argument is "incl.cut" which now has a value of 0.8.
##
## $Materialm_low
##
## OUT: output value
## n: number of cases in configuration
## incl: sufficiency inclusion score
## PRI: proportional reduction in inconsistency
##
## HL HS HT OUT n incl PRI
## 4 0 1 1 0 15 0.475 0.327
## 3 0 1 0 0 5 0.468 0.307
## 6 1 0 1 0 54 0.337 0.193
## 8 1 1 1 0 116 0.336 0.171
## 5 1 0 0 0 83 0.336 0.163
## 7 1 1 0 0 39 0.331 0.158
## 2 0 0 1 0 82 0.315 0.136
## 1 0 0 0 0 7 0.269 0.019
##
##
## $Materialm
##
## OUT: output value
## n: number of cases in configuration
## incl: sufficiency inclusion score
## PRI: proportional reduction in inconsistency
##
## HL HS HT OUT n incl PRI
## 4 0 1 1 0 15 0.480 0.359
## 3 0 1 0 0 5 0.467 0.336
## 8 1 1 1 0 116 0.322 0.190
## 5 1 0 0 0 83 0.321 0.182
## 6 1 0 1 0 54 0.319 0.208
## 7 1 1 0 0 39 0.314 0.172
## 2 0 0 1 0 82 0.297 0.152
## 1 0 0 0 0 7 0.242 0.022
##
##
## $Materialm_high
##
## OUT: output value
## n: number of cases in configuration
## incl: sufficiency inclusion score
## PRI: proportional reduction in inconsistency
##
## HL HS HT OUT n incl PRI
## 4 0 1 1 0 15 0.490 0.397
## 3 0 1 0 0 5 0.472 0.372
## 8 1 1 1 0 116 0.308 0.214
## 5 1 0 0 0 83 0.308 0.206
## 6 1 0 1 0 54 0.300 0.225
## 7 1 1 0 0 39 0.297 0.188
## 2 0 0 1 0 82 0.282 0.173
## 1 0 0 0 0 7 0.211 0.029
##
## It seems that all output values have been coded to zero.
## Suggestion: lower the inclusion score for the presence of the outcome,
## the relevant argument is "incl.cut" which now has a value of 0.8.
##
## $RelCommm_low
##
## OUT: output value
## n: number of cases in configuration
## incl: sufficiency inclusion score
## PRI: proportional reduction in inconsistency
##
## HL HS HT OUT n incl PRI
## 4 0 1 1 0 15 0.461 0.259
## 6 1 0 1 0 54 0.420 0.226
## 2 0 0 1 0 82 0.420 0.191
## 3 0 1 0 0 5 0.404 0.192
## 8 1 1 1 0 116 0.402 0.188
## 1 0 0 0 0 7 0.369 0.000
## 5 1 0 0 0 83 0.354 0.111
## 7 1 1 0 0 39 0.336 0.137
##
## $RelCommm
##
## OUT: output value
## n: number of cases in configuration
## incl: sufficiency inclusion score
## PRI: proportional reduction in inconsistency
##
## HL HS HT OUT n incl PRI
## 4 0 1 1 0 15 0.458 0.286
## 2 0 0 1 0 82 0.411 0.216
## 6 1 0 1 0 54 0.408 0.248
## 8 1 1 1 0 116 0.387 0.208
## 3 0 1 0 0 5 0.385 0.207
## 1 0 0 0 0 7 0.341 0.000
## 5 1 0 0 0 83 0.333 0.126
## 7 1 1 0 0 39 0.315 0.153
##
## $RelCommm_high
##
## OUT: output value
## n: number of cases in configuration
## incl: sufficiency inclusion score
## PRI: proportional reduction in inconsistency
##
## HL HS HT OUT n incl PRI
## 4 0 1 1 0 15 0.453 0.316
## 2 0 0 1 0 82 0.403 0.250
## 6 1 0 1 0 54 0.395 0.271
## 8 1 1 1 0 116 0.370 0.232
## 3 0 1 0 0 5 0.357 0.219
## 5 1 0 0 0 83 0.310 0.146
## 1 0 0 0 0 7 0.300 0.000
## 7 1 1 0 0 39 0.293 0.172
##
## It seems that all output values have been coded to zero.
## Suggestion: lower the inclusion score for the presence of the outcome,
## the relevant argument is "incl.cut" which now has a value of 0.8.

#### Performing QCA: Necessity analysis: 2nd Level -> Reasons MNC
# create processed lists based on QCA::pof function, which applies the following relations

# Result: Necessity Analysis: Nurses Characteristics necessary for Reasons for MNC

## $Demandm_low
##
## inclN RoN covN
## -----------------------------
## 1 NQual 0.919 0.238 0.732
## 2 Role 0.821 0.465 0.740
## 3 NExp 0.279 0.919 0.762
## -----------------------------
##
##
## $Demandm
##
## inclN RoN covN
## -----------------------------
## 1 NQual 0.919 0.257 0.758
## 2 Role 0.821 0.492 0.766
## 3 NExp 0.280 0.928 0.791
## -----------------------------
##
##
## $Demandm_high
##
## inclN RoN covN
## -----------------------------
## 1 NQual 0.919 0.280 0.785
## 2 Role 0.821 0.523 0.793
## 3 NExp 0.280 0.938 0.820
## -----------------------------
##
##

## $Laborm_low
##
## inclN RoN covN
## -----------------------------
## 1 NQual 0.923 0.188 0.638
## 2 Role 0.841 0.398 0.658
## 3 NExp 0.282 0.891 0.668
## -----------------------------
##
##
## $Laborm
##
## inclN RoN covN
## -----------------------------
## 1 NQual 0.922 0.196 0.657
## 2 Role 0.843 0.414 0.679
## 3 NExp 0.283 0.898 0.692
## -----------------------------
##
##
## $Laborm_high
##
## inclN RoN covN
## -----------------------------
## 1 NQual 0.922 0.207 0.680
## 2 Role 0.844 0.434 0.705
## 3 NExp 0.284 0.906 0.718
## -----------------------------
##
##

## $Materialm_low
##
## inclN RoN covN
## -----------------------------
## 1 NQual 0.907 0.111 0.331
## 2 Role 0.850 0.259 0.351
## 3 NExp 0.327 0.821 0.410
## -----------------------------
##
##
## $Materialm
##
## inclN RoN covN
## -----------------------------
## 1 NQual 0.904 0.109 0.315
## 2 Role 0.856 0.255 0.338
## 3 NExp 0.336 0.819 0.401
## -----------------------------
##
##
## $Materialm_high
##
## inclN RoN covN
## -----------------------------
## 1 NQual 0.899 0.107 0.300
## 2 Role 0.864 0.251 0.326
## 3 NExp 0.344 0.817 0.393
## -----------------------------
##
##

## $RelCommm_low
##
## inclN RoN covN
## -----------------------------
## 1 NQual 0.932 0.122 0.397
## 2 Role 0.840 0.275 0.405
## 3 NExp 0.275 0.819 0.401
## -----------------------------
##
##
## $RelCommm
##
## inclN RoN covN
## -----------------------------
## 1 NQual 0.933 0.120 0.383
## 2 Role 0.843 0.271 0.392
## 3 NExp 0.276 0.816 0.388
## -----------------------------
##
##
## $RelCommm_high
##
## inclN RoN covN
## -----------------------------
## 1 NQual 0.935 0.117 0.367
## 2 Role 0.847 0.266 0.376
## 3 NExp 0.278 0.812 0.373
## -----------------------------

# Result: Necessity Analysis: Unit Characteristics necessary for Reasons for MNC

## $Demandm_low
##
## inclN RoN covN
## ---------------------------
## 1 UT 0.509 0.761 0.714
## 2 US 0.474 0.837 0.766
## 3 UNS 0.753 0.636 0.773
## ---------------------------
##
##
## $Demandm
##
## inclN RoN covN
## ---------------------------
## 1 UT 0.508 0.776 0.738
## 2 US 0.475 0.855 0.796
## 3 UNS 0.756 0.669 0.804
## ---------------------------
##
##
## $Demandm_high
##
## inclN RoN covN
## ---------------------------
## 1 UT 0.507 0.794 0.764
## 2 US 0.477 0.874 0.827
## 3 UNS 0.759 0.707 0.836
## ---------------------------
##
##

## $Laborm_low
##
## inclN RoN covN
## ---------------------------
## 1 UT 0.506 0.703 0.616
## 2 US 0.462 0.775 0.650
## 3 UNS 0.786 0.570 0.700
## ---------------------------
##
##
## $Laborm
##
## inclN RoN covN
## ---------------------------
## 1 UT 0.504 0.712 0.633
## 2 US 0.464 0.786 0.672
## 3 UNS 0.794 0.595 0.729
## ---------------------------
##
##
## $Laborm_high
##
## inclN RoN covN
## ---------------------------
## 1 UT 0.502 0.724 0.653
## 2 US 0.466 0.800 0.699
## 3 UNS 0.802 0.626 0.763
## ---------------------------
##
##

## $Materialm_low
##
## inclN RoN covN
## ---------------------------
## 1 UT 0.516 0.577 0.332
## 2 US 0.468 0.648 0.348
## 3 UNS 0.760 0.382 0.358
## ---------------------------
##
##
## $Materialm
##
## inclN RoN covN
## ---------------------------
## 1 UT 0.515 0.571 0.317
## 2 US 0.470 0.643 0.333
## 3 UNS 0.768 0.378 0.345
## ---------------------------
##
##
## $Materialm_high
##
## inclN RoN covN
## ---------------------------
## 1 UT 0.515 0.566 0.303
## 2 US 0.471 0.639 0.320
## 3 UNS 0.776 0.374 0.334
## ---------------------------
##
##

## $RelCommm_low
##
## inclN RoN covN
## ---------------------------
## 1 UT 0.502 0.593 0.377
## 2 US 0.453 0.665 0.393
## 3 UNS 0.785 0.411 0.431
## ---------------------------
##
##
## $RelCommm
##
## inclN RoN covN
## ---------------------------
## 1 UT 0.498 0.587 0.360
## 2 US 0.452 0.659 0.377
## 3 UNS 0.795 0.407 0.420
## ---------------------------
##
##
## $RelCommm_high
##
## inclN RoN covN
## ---------------------------
## 1 UT 0.493 0.580 0.340
## 2 US 0.449 0.652 0.358
## 3 UNS 0.806 0.401 0.408
## ---------------------------

# Result: Necessity Analysis: Hospital Characteristics necessary for Reasons for MNC

## $Demandm_low
##
## inclN RoN covN
## --------------------------
## 1 HT 0.666 0.655 0.735
## 2 HS 0.436 0.829 0.734
## 3 HL 0.733 0.589 0.739
## --------------------------
##
##
## $Demandm
##
## inclN RoN covN
## --------------------------
## 1 HT 0.666 0.677 0.761
## 2 HS 0.437 0.844 0.761
## 3 HL 0.733 0.614 0.766
## --------------------------
##
##
## $Demandm_high
##
## inclN RoN covN
## --------------------------
## 1 HT 0.666 0.703 0.788
## 2 HS 0.437 0.859 0.789
## 3 HL 0.733 0.644 0.793
## --------------------------
##
##

## $Laborm_low
##
## inclN RoN covN
## --------------------------
## 1 HT 0.678 0.588 0.649
## 2 HS 0.436 0.780 0.637
## 3 HL 0.721 0.503 0.631
## --------------------------
##
##
## $Laborm
##
## inclN RoN covN
## --------------------------
## 1 HT 0.678 0.603 0.669
## 2 HS 0.436 0.790 0.657
## 3 HL 0.720 0.516 0.650
## --------------------------
##
##
## $Laborm_high
##
## inclN RoN covN
## --------------------------
## 1 HT 0.678 0.620 0.693
## 2 HS 0.437 0.802 0.681
## 3 HL 0.720 0.533 0.672
## --------------------------
##
##

## $Materialm_low
##
## inclN RoN covN
## --------------------------
## 1 HT 0.667 0.431 0.338
## 2 HS 0.454 0.665 0.351
## 3 HL 0.725 0.360 0.336
## --------------------------
##
##
## $Materialm
##
## inclN RoN covN
## --------------------------
## 1 HT 0.667 0.426 0.322
## 2 HS 0.458 0.661 0.338
## 3 HL 0.724 0.354 0.320
## --------------------------
##
##
## $Materialm_high
##
## inclN RoN covN
## --------------------------
## 1 HT 0.668 0.421 0.309
## 2 HS 0.462 0.657 0.326
## 3 HL 0.722 0.350 0.305
## --------------------------
##
##

## $RelCommm_low
##
## inclN RoN covN
## --------------------------
## 1 HT 0.701 0.461 0.414
## 2 HS 0.435 0.680 0.392
## 3 HL 0.709 0.377 0.383
## --------------------------
##
##
## $RelCommm
##
## inclN RoN covN
## --------------------------
## 1 HT 0.708 0.457 0.403
## 2 HS 0.435 0.675 0.377
## 3 HL 0.704 0.371 0.366
## --------------------------
##
##
## $RelCommm_high
##
## inclN RoN covN
## --------------------------
## 1 HT 0.717 0.451 0.390
## 2 HS 0.434 0.669 0.360
## 3 HL 0.699 0.364 0.347
## --------------------------

#### Performing QCA: Sufficiency analysis: 2nd Level -> Reasons MNC
# create processed lists based on QCA::pof function, which applies the following relations

# Result: Sufficiency Analysis: Nurses Characteristics sufficient for Reasons for MNC

## $Demandm_low
##
## inclS PRI covS covU
## ------------------------------------
## 1 NQual 0.732 0.667 0.919 0.172
## 2 Role 0.740 0.676 0.821 0.056
## 3 NExp 0.762 0.708 0.279 0.000
## ------------------------------------
##
##
## $Demandm
##
## inclS PRI covS covU
## ------------------------------------
## 1 NQual 0.758 0.712 0.919 0.172
## 2 Role 0.766 0.721 0.821 0.056
## 3 NExp 0.791 0.754 0.280 0.000
## ------------------------------------
##
##
## $Demandm_high
##
## inclS PRI covS covU
## ------------------------------------
## 1 NQual 0.785 0.756 0.919 0.172
## 2 Role 0.793 0.765 0.821 0.056
## 3 NExp 0.820 0.798 0.280 0.000
## ------------------------------------
##
##

## $Laborm_low
##
## inclS PRI covS covU
## ------------------------------------
## 1 NQual 0.638 0.506 0.923 0.152
## 2 Role 0.658 0.537 0.841 0.055
## 3 NExp 0.668 0.554 0.282 0.000
## ------------------------------------
##
##
## $Laborm
##
## inclS PRI covS covU
## ------------------------------------
## 1 NQual 0.657 0.554 0.922 0.151
## 2 Role 0.679 0.587 0.843 0.055
## 3 NExp 0.692 0.605 0.283 0.000
## ------------------------------------
##
##
## $Laborm_high
##
## inclS PRI covS covU
## ------------------------------------
## 1 NQual 0.680 0.607 0.922 0.149
## 2 Role 0.705 0.641 0.844 0.055
## 3 NExp 0.718 0.659 0.284 0.000
## ------------------------------------
##
##

## $Materialm_low
##
## inclS PRI covS covU
## ------------------------------------
## 1 NQual 0.331 0.163 0.907 0.140
## 2 Role 0.351 0.184 0.850 0.066
## 3 NExp 0.410 0.234 0.327 0.000
## ------------------------------------
##
##
## $Materialm
##
## inclS PRI covS covU
## ------------------------------------
## 1 NQual 0.315 0.180 0.904 0.133
## 2 Role 0.338 0.203 0.856 0.069
## 3 NExp 0.401 0.259 0.336 0.000
## ------------------------------------
##
##
## $Materialm_high
##
## inclS PRI covS covU
## ------------------------------------
## 1 NQual 0.300 0.200 0.899 0.125
## 2 Role 0.326 0.227 0.864 0.073
## 3 NExp 0.393 0.288 0.344 0.000
## ------------------------------------
##
##

## $RelCommm_low
##
## inclS PRI covS covU
## ------------------------------------
## 1 NQual 0.397 0.178 0.932 0.150
## 2 Role 0.405 0.186 0.840 0.054
## 3 NExp 0.401 0.177 0.275 0.000
## ------------------------------------
##
##
## $RelCommm
##
## inclS PRI covS covU
## ------------------------------------
## 1 NQual 0.383 0.198 0.933 0.146
## 2 Role 0.392 0.207 0.843 0.055
## 3 NExp 0.388 0.199 0.276 0.000
## ------------------------------------
##
##
## $RelCommm_high
##
## inclS PRI covS covU
## ------------------------------------
## 1 NQual 0.367 0.223 0.935 0.141
## 2 Role 0.376 0.232 0.847 0.056
## 3 NExp 0.373 0.226 0.278 0.000
## ------------------------------------

# Result: Sufficiency Analysis: Unit Characteristics sufficient for Reasons for MNC

## $Demandm_low
##
## inclS PRI covS covU
## ----------------------------------
## 1 UT 0.714 0.641 0.509 0.083
## 2 US 0.766 0.710 0.474 0.046
## 3 UNS 0.773 0.720 0.753 0.181
## ----------------------------------
##
##
## $Demandm
##
## inclS PRI covS covU
## ----------------------------------
## 1 UT 0.738 0.686 0.508 0.082
## 2 US 0.796 0.758 0.475 0.045
## 3 UNS 0.804 0.768 0.756 0.181
## ----------------------------------
##
##
## $Demandm_high
##
## inclS PRI covS covU
## ----------------------------------
## 1 UT 0.764 0.729 0.507 0.080
## 2 US 0.827 0.804 0.477 0.044
## 3 UNS 0.836 0.815 0.759 0.181
## ----------------------------------
##
##

## $Laborm_low
##
## inclS PRI covS covU
## ----------------------------------
## 1 UT 0.616 0.471 0.506 0.077
## 2 US 0.650 0.521 0.462 0.042
## 3 UNS 0.700 0.596 0.786 0.193
## ----------------------------------
##
##
## $Laborm
##
## inclS PRI covS covU
## ----------------------------------
## 1 UT 0.633 0.518 0.504 0.074
## 2 US 0.672 0.572 0.464 0.041
## 3 UNS 0.729 0.651 0.794 0.196
## ----------------------------------
##
##
## $Laborm_high
##
## inclS PRI covS covU
## ----------------------------------
## 1 UT 0.653 0.569 0.502 0.070
## 2 US 0.699 0.629 0.466 0.039
## 3 UNS 0.763 0.711 0.802 0.198
## ----------------------------------
##
##

## $Materialm_low
##
## inclS PRI covS covU
## ----------------------------------
## 1 UT 0.332 0.167 0.516 0.089
## 2 US 0.348 0.174 0.468 0.045
## 3 UNS 0.358 0.187 0.760 0.165
## ----------------------------------
##
##
## $Materialm
##
## inclS PRI covS covU
## ----------------------------------
## 1 UT 0.317 0.185 0.515 0.086
## 2 US 0.333 0.192 0.470 0.043
## 3 UNS 0.345 0.207 0.768 0.165
## ----------------------------------
##
##
## $Materialm_high
##
## inclS PRI covS covU
## ----------------------------------
## 1 UT 0.303 0.206 0.515 0.084
## 2 US 0.320 0.214 0.471 0.040
## 3 UNS 0.334 0.232 0.776 0.166
## ----------------------------------
##
##

## $RelCommm_low
##
## inclS PRI covS covU
## ----------------------------------
## 1 UT 0.377 0.150 0.502 0.080
## 2 US 0.393 0.166 0.453 0.043
## 3 UNS 0.431 0.201 0.785 0.195
## ----------------------------------
##
##
## $RelCommm
##
## inclS PRI covS covU
## ----------------------------------
## 1 UT 0.360 0.168 0.498 0.077
## 2 US 0.377 0.185 0.452 0.041
## 3 UNS 0.420 0.224 0.795 0.199
## ----------------------------------
##
##
## $RelCommm_high
##
## inclS PRI covS covU
## ----------------------------------
## 1 UT 0.340 0.190 0.493 0.073
## 2 US 0.358 0.208 0.449 0.038
## 3 UNS 0.408 0.252 0.806 0.204
## ----------------------------------

# Result: Sufficiency Analysis: Hospital Characteristics sufficient for Reasons for MNC

## $Demandm_low
##
## inclS PRI covS covU
## ---------------------------------
## 1 HT 0.735 0.671 0.666 0.200
## 2 HS 0.734 0.673 0.436 0.012
## 3 HL 0.739 0.676 0.733 0.201
## ---------------------------------
##
##
## $Demandm
##
## inclS PRI covS covU
## ---------------------------------
## 1 HT 0.761 0.716 0.666 0.199
## 2 HS 0.761 0.719 0.437 0.012
## 3 HL 0.766 0.722 0.733 0.201
## ---------------------------------
##
##
## $Demandm_high
##
## inclS PRI covS covU
## ---------------------------------
## 1 HT 0.788 0.759 0.666 0.199
## 2 HS 0.789 0.763 0.437 0.012
## 3 HL 0.793 0.766 0.733 0.201
## ---------------------------------
##
##

## $Laborm_low
##
## inclS PRI covS covU
## ---------------------------------
## 1 HT 0.649 0.524 0.678 0.207
## 2 HS 0.637 0.503 0.436 0.013
## 3 HL 0.631 0.495 0.721 0.195
## ---------------------------------
##
##
## $Laborm
##
## inclS PRI covS covU
## ---------------------------------
## 1 HT 0.669 0.573 0.678 0.207
## 2 HS 0.657 0.553 0.436 0.013
## 3 HL 0.650 0.543 0.720 0.195
## ---------------------------------
##
##
## $Laborm_high
##
## inclS PRI covS covU
## ---------------------------------
## 1 HT 0.693 0.625 0.678 0.208
## 2 HS 0.681 0.607 0.437 0.013
## 3 HL 0.672 0.596 0.720 0.194
## ---------------------------------
##
##

## $Materialm_low
##
## inclS PRI covS covU
## ---------------------------------
## 1 HT 0.338 0.173 0.667 0.191
## 2 HS 0.351 0.185 0.454 0.017
## 3 HL 0.336 0.171 0.725 0.206
## ---------------------------------
##
##
## $Materialm
##
## inclS PRI covS covU
## ---------------------------------
## 1 HT 0.322 0.191 0.667 0.189
## 2 HS 0.338 0.204 0.458 0.018
## 3 HL 0.320 0.189 0.724 0.207
## ---------------------------------
##
##
## $Materialm_high
##
## inclS PRI covS covU
## ---------------------------------
## 1 HT 0.309 0.214 0.668 0.187
## 2 HS 0.326 0.228 0.462 0.019
## 3 HL 0.305 0.210 0.722 0.207
## ---------------------------------
##
##

## $RelCommm_low
##
## inclS PRI covS covU
## ---------------------------------
## 1 HT 0.414 0.201 0.701 0.218
## 2 HS 0.392 0.182 0.435 0.013
## 3 HL 0.383 0.167 0.709 0.186
## ---------------------------------
##
##
## $RelCommm
##
## inclS PRI covS covU
## ---------------------------------
## 1 HT 0.403 0.223 0.708 0.222
## 2 HS 0.377 0.202 0.435 0.013
## 3 HL 0.366 0.185 0.704 0.182
## ---------------------------------
##
##
## $RelCommm_high
##
## inclS PRI covS covU
## ---------------------------------
## 1 HT 0.390 0.250 0.717 0.228
## 2 HS 0.360 0.225 0.434 0.012
## 3 HL 0.347 0.207 0.699 0.177
## ---------------------------------

#### Performing QCA: Minimizing tT: 2nd Level -> Reasons MNC

# Main Results: Configurational Models

# Result: Final QCA Solutions for Configurations of Nurses Characteristics triggering Reasons for MNC (n.cut=3)

## $Demandm_low
## [1] "Error: None of the values in OUT is explained. Please check the truth table."
##
## $Demandm
##
## M1: ~NQual*Role*NExp -> Demandm
##
## inclS PRI covS covU
## -----------------------------------------------
## 1 ~NQual*Role*NExp 0.834 0.801 0.025 -
## -----------------------------------------------
## M1 0.834 0.801 0.025
##
##
## $Demandm_high
##
## M1: ~NQual*Role + Role*NExp -> Demandm_high
##
## inclS PRI covS covU
## ------------------------------------------
## 1 ~NQual*Role 0.827 0.803 0.081 0.056
## 2 Role*NExp 0.824 0.803 0.274 0.249
## ------------------------------------------
## M1 0.821 0.799 0.330
##
##

## $Laborm_low
## [1] "Error: None of the values in OUT is explained. Please check the truth table."
##
## $Laborm
## [1] "Error: None of the values in OUT is explained. Please check the truth table."
##
## $Laborm_high
## [1] "Error: None of the values in OUT is explained. Please check the truth table."
##

## $Materialm_low
## [1] "Error: None of the values in OUT is explained. Please check the truth table."
##
## $Materialm
## [1] "Error: None of the values in OUT is explained. Please check the truth table."
##
## $Materialm_high
## [1] "Error: None of the values in OUT is explained. Please check the truth table."
##

## $RelCommm_low
## [1] "Error: None of the values in OUT is explained. Please check the truth table."
##
## $RelCommm
## [1] "Error: None of the values in OUT is explained. Please check the truth table."
##
## $RelCommm_high
## [1] "Error: None of the values in OUT is explained. Please check the truth table."

# Result: Final QCA Solutions for Configurations of Unit Characteristics triggering Reasons for MNC (n.cut=3)

## $Demandm_low
##
## M1: UNS*US*UT -> Demandm_low
##
## inclS PRI covS covU
## ----------------------------------------
## 1 UNS*US*UT 0.805 0.760 0.180 -
## ----------------------------------------
## M1 0.805 0.760 0.180
##
##
## $Demandm
##
## M1: UNS*US + UNS*~UT -> Demandm
##
## inclS PRI covS covU
## --------------------------------------
## 1 UNS*US 0.834 0.803 0.380 0.181
## 2 UNS*~UT 0.827 0.798 0.380 0.181
## --------------------------------------
## M1 0.830 0.802 0.561
##
##
## $Demandm_high
##
## M1: UNS*US + UNS*~UT -> Demandm_high
##
## inclS PRI covS covU
## --------------------------------------
## 1 UNS*US 0.870 0.853 0.383 0.182
## 2 UNS*~UT 0.860 0.845 0.381 0.181
## --------------------------------------
## M1 0.864 0.848 0.563
##
##

## $Laborm_low
## [1] "Error: None of the values in OUT is explained. Please check the truth table."
##
## $Laborm
## [1] "Error: None of the values in OUT is explained. Please check the truth table."
##
## $Laborm_high
##
## M1: UNS*~US*~UT -> Laborm_high
##
## inclS PRI covS covU
## ------------------------------------------
## 1 UNS*~US*~UT 0.806 0.769 0.198 -
## ------------------------------------------
## M1 0.806 0.769 0.198
##
##

## $Materialm_low
## [1] "Error: None of the values in OUT is explained. Please check the truth table."
##
## $Materialm
## [1] "Error: None of the values in OUT is explained. Please check the truth table."
##
## $Materialm_high
## [1] "Error: None of the values in OUT is explained. Please check the truth table."
##

## $RelCommm_low
## [1] "Error: None of the values in OUT is explained. Please check the truth table."
##
## $RelCommm
## [1] "Error: None of the values in OUT is explained. Please check the truth table."
##
## $RelCommm_high
## [1] "Error: None of the values in OUT is explained. Please check the truth table."

# Result: Final QCA Solutions for Configurations of Hospital Characteristics triggering Reasons for MNC (n.cut=3)

## $Demandm_low
##
## M1: ~HL*~HS*~HT -> Demandm_low
##
## inclS PRI covS covU
## ------------------------------------------
## 1 ~HL*~HS*~HT 0.868 0.848 0.021 -
## ------------------------------------------
## M1 0.868 0.848 0.021
##
##
## $Demandm
##
## M1: HL*~HS*HT + ~HL*~HS*~HT -> Demandm
##
## inclS PRI covS covU
## ------------------------------------------
## 1 HL*~HS*HT 0.802 0.768 0.142 0.142
## 2 ~HL*~HS*~HT 0.907 0.897 0.021 0.021
## ------------------------------------------
## M1 0.814 0.784 0.163
##
##
## $Demandm_high
##
## M1: HL*HS*~HT + HL*~HS*HT + ~HL*~HS*~HT -> Demandm_high
##
## inclS PRI covS covU
## ------------------------------------------
## 1 HL*HS*~HT 0.808 0.791 0.100 0.100
## 2 HL*~HS*HT 0.828 0.806 0.141 0.141
## 3 ~HL*~HS*~HT 0.947 0.944 0.021 0.021
## ------------------------------------------
## M1 0.828 0.810 0.262
##
##

## $Laborm_low
## [1] "Error: None of the values in OUT is explained. Please check the truth table."
##
## $Laborm
## [1] "Error: None of the values in OUT is explained. Please check the truth table."
##
## $Laborm_high
## [1] "Error: None of the values in OUT is explained. Please check the truth table."
##

## $Materialm_low
## [1] "Error: None of the values in OUT is explained. Please check the truth table."
##
## $Materialm
## [1] "Error: None of the values in OUT is explained. Please check the truth table."
##
## $Materialm_high
## [1] "Error: None of the values in OUT is explained. Please check the truth table."
##

## $RelCommm_low
## [1] "Error: None of the values in OUT is explained. Please check the truth table."
##
## $RelCommm
## [1] "Error: None of the values in OUT is explained. Please check the truth table."
##
## $RelCommm_high
## [1] "Error: None of the values in OUT is explained. Please check the truth table."

####################################################
### Data Processing 2/3 ###
####################################################

# Variables Description
# _low: Low calibration approach ( 1 / 1.5 / 6 )
# _high: High calibration approach (1 / 3.5 / 6)


#### Performing QCA: Truth Tables: Reasons MNC -> MNC

# Setting Conditions: Singular Reasons for MNC (According to Table 1)
condsReasons <- base::subset(c1QCA, select = c("Demandm", "Laborm", "Materialm", "RelCommm"))

# Setting Conditions:create vector with outcomes of MNC
# (According to Table 1)
outcome_MNC = c("SA1_low","SA1_high", # SA1: Ambulation/mobilization as frequently as necessary or as ordered
 "SA2_low","SA2_high", # SA2: Turning patient as frequently as necessary or as ordered
 "SA3_low","SA3_high", # SA3: Feeding patient when the food is still warm
 "SA4_low","SA4_high", # SA4: Setting up meals for patient who feeds themselves
 "SA5_low","SA5_high", # SA5: Administrating medications within the stipulated scheduled time
 "SA6_low","SA6_high", # SA6: Vital signs assessed as frequently as necessary or as ordered
 "SA7_low","SA7_high", # SA7: Monitoring intake/output
 "SA8_low","SA8_high", # SA8: Full documentation of all necessary nursing relevant data
 "SA9a_low","SA9a_high", # SA9a: Patient teaching about illness and planed care
 "SA9b_low","SA9b_high", # SA9b: Informal caregiver teaching about illness and planed care
 "SA10a_low","SA10a_high", # SA10a:Emotional support to patient
 "SA10b_low","SA10b_high", # SA10b: Emotional support to informal caregivers
 "SA11_low","SA11_high", # SA11: Assisting with body and skin care
 "SA12_low","SA12_high", # SA12: Assisting with oral and dental care
 "SA13_low","SA13_high", # SA13:Own hand hygiene (of nurses)
 "SA14a_low","SA14a_high", # SA14a: Patient discharge planning
 "SA14b_low","SA14b_high", # SA14b: Counselling and training patients for discharge
 "SA14c_low","SA14c_high", # SA14b: Counselling and training patients for discharge
 "SA15_low","SA15_high", # SA15: Bedside glucose monitoring as frequently as necessary or as ordered
 "SA16_low","SA16_high", # SA16: Performing comprehensive assessment of the patient's condition
 "SA17_low","SA17_high", # SA17: Focused reassessments according to patient condition
 "SA18a_low","SA18a_high", # SA18a: Peripheral venous catheter site care according to hospital standards
 "SA18b_low","SA18b_high", # SA18b: Central line site care according to hospital standards
 "SA19_low","SA19_high", # SA19: Timely responding to patient call light
 "SA20_low","SA20_high", # SA20: Timely administrating PRN medication following patient*s request
 "SA21_low","SA21_high", # SA21: Assess effectiveness of PRN medications
 "SA22_low","SA22_high", # SA22: Attend interdisciplinary care conferences
 "SA23_low","SA23_high", # SA23: Timely assisting with toileting needs following patient*s request
 "SA24_low","SA24_high", # SA24: Skin damage and/or wound care
 "SA25_low", "SA25_high") # SA25: Adequate surveillance of confused/impaired patients

#### Performing QCA: Truth Tables: 1st Level conditions -> MNC

# Result: Truth Table: Reasons for MNC triggering single Outcomes of MNC (for n.cut = 3)

## $SA1_low
##
## OUT: output value
## n: number of cases in configuration
## incl: sufficiency inclusion score
## PRI: proportional reduction in inconsistency
##
## Demandm Laborm Materialm RelCommm OUT n incl PRI
## 14 1 1 0 1 1 56 0.970 0.939
## 15 1 1 1 0 1 45 0.945 0.882
## 13 1 1 0 0 1 115 0.940 0.892
## 11 1 0 1 0 1 4 0.939 0.770
## 16 1 1 1 1 1 65 0.930 0.873
## 5 0 1 0 0 1 11 0.903 0.721
## 9 1 0 0 0 1 59 0.889 0.759
## 1 0 0 0 0 0 39 0.729 0.419
##
##
## $SA1_high
##
## OUT: output value
## n: number of cases in configuration
## incl: sufficiency inclusion score
## PRI: proportional reduction in inconsistency
##
## Demandm Laborm Materialm RelCommm OUT n incl PRI
## 11 1 0 1 0 1 4 0.884 0.476
## 14 1 1 0 1 1 56 0.878 0.704
## 15 1 1 1 0 1 45 0.849 0.612
## 5 0 1 0 0 1 11 0.826 0.448
## 16 1 1 1 1 1 65 0.820 0.602
## 13 1 1 0 0 0 115 0.786 0.570
## 9 1 0 0 0 0 59 0.747 0.397
## 1 0 0 0 0 0 39 0.611 0.155
##
##

## $SA2_low
##
## OUT: output value
## n: number of cases in configuration
## incl: sufficiency inclusion score
## PRI: proportional reduction in inconsistency
##
## Demandm Laborm Materialm RelCommm OUT n incl PRI
## 14 1 1 0 1 1 56 0.813 0.590
## 11 1 0 1 0 1 4 0.807 0.331
## 15 1 1 1 0 1 45 0.803 0.529
## 5 0 1 0 0 0 11 0.798 0.387
## 16 1 1 1 1 0 65 0.785 0.554
## 13 1 1 0 0 0 115 0.754 0.519
## 9 1 0 0 0 0 59 0.739 0.394
## 1 0 0 0 0 0 39 0.621 0.189
##
##
## $SA2_high
##
## OUT: output value
## n: number of cases in configuration
## incl: sufficiency inclusion score
## PRI: proportional reduction in inconsistency
##
## Demandm Laborm Materialm RelCommm OUT n incl PRI
## 11 1 0 1 0 0 4 0.741 0.187
## 5 0 1 0 0 0 11 0.669 0.117
## 14 1 1 0 1 0 56 0.646 0.252
## 15 1 1 1 0 0 45 0.622 0.175
## 16 1 1 1 1 0 65 0.593 0.212
## 9 1 0 0 0 0 59 0.535 0.087
## 13 1 1 0 0 0 115 0.515 0.148
## 1 0 0 0 0 0 39 0.492 0.031
##
## It seems that all output values have been coded to zero.
## Suggestion: lower the inclusion score for the presence of the outcome,
## the relevant argument is "incl.cut" which now has a value of 0.8.
##
##

## $SA3_low
##
## OUT: output value
## n: number of cases in configuration
## incl: sufficiency inclusion score
## PRI: proportional reduction in inconsistency
##
## Demandm Laborm Materialm RelCommm OUT n incl PRI
## 11 1 0 1 0 1 4 0.861 0.450
## 15 1 1 1 0 1 45 0.845 0.632
## 16 1 1 1 1 1 65 0.817 0.640
## 14 1 1 0 1 0 56 0.778 0.555
## 13 1 1 0 0 0 115 0.734 0.525
## 5 0 1 0 0 0 11 0.719 0.292
## 9 1 0 0 0 0 59 0.702 0.381
## 1 0 0 0 0 0 39 0.553 0.149
##
##
## $SA3_high
##
## OUT: output value
## n: number of cases in configuration
## incl: sufficiency inclusion score
## PRI: proportional reduction in inconsistency
##
## Demandm Laborm Materialm RelCommm OUT n incl PRI
## 11 1 0 1 0 0 4 0.777 0.203
## 15 1 1 1 0 0 45 0.684 0.261
## 14 1 1 0 1 0 56 0.655 0.296
## 16 1 1 1 1 0 65 0.654 0.324
## 5 0 1 0 0 0 11 0.638 0.120
## 13 1 1 0 0 0 115 0.547 0.207
## 9 1 0 0 0 0 59 0.538 0.115
## 1 0 0 0 0 0 39 0.466 0.040
##
## It seems that all output values have been coded to zero.
## Suggestion: lower the inclusion score for the presence of the outcome,
## the relevant argument is "incl.cut" which now has a value of 0.8.
##
##

## $SA4_low
##
## OUT: output value
## n: number of cases in configuration
## incl: sufficiency inclusion score
## PRI: proportional reduction in inconsistency
##
## Demandm Laborm Materialm RelCommm OUT n incl PRI
## 15 1 1 1 0 0 45 0.796 0.507
## 14 1 1 0 1 0 56 0.790 0.516
## 11 1 0 1 0 0 4 0.789 0.247
## 16 1 1 1 1 0 65 0.768 0.528
## 13 1 1 0 0 0 115 0.700 0.419
## 5 0 1 0 0 0 11 0.692 0.220
## 9 1 0 0 0 0 59 0.659 0.267
## 1 0 0 0 0 0 39 0.543 0.122
##
## It seems that all output values have been coded to zero.
## Suggestion: lower the inclusion score for the presence of the outcome,
## the relevant argument is "incl.cut" which now has a value of 0.8.
##
##
## $SA4_high
##
## OUT: output value
## n: number of cases in configuration
## incl: sufficiency inclusion score
## PRI: proportional reduction in inconsistency
##
## Demandm Laborm Materialm RelCommm OUT n incl PRI
## 11 1 0 1 0 0 4 0.706 0.088
## 15 1 1 1 0 0 45 0.609 0.157
## 5 0 1 0 0 0 11 0.595 0.084
## 16 1 1 1 1 0 65 0.590 0.222
## 14 1 1 0 1 0 56 0.580 0.146
## 9 1 0 0 0 0 59 0.478 0.059
## 13 1 1 0 0 0 115 0.460 0.095
## 1 0 0 0 0 0 39 0.438 0.032
##
## It seems that all output values have been coded to zero.
## Suggestion: lower the inclusion score for the presence of the outcome,
## the relevant argument is "incl.cut" which now has a value of 0.8.
##
##

## $SA5_low
##
## OUT: output value
## n: number of cases in configuration
## incl: sufficiency inclusion score
## PRI: proportional reduction in inconsistency
##
## Demandm Laborm Materialm RelCommm OUT n incl PRI
## 11 1 0 1 0 1 4 0.909 0.609
## 15 1 1 1 0 1 45 0.899 0.747
## 14 1 1 0 1 1 56 0.875 0.702
## 16 1 1 1 1 1 65 0.868 0.730
## 13 1 1 0 0 1 115 0.836 0.663
## 5 0 1 0 0 1 11 0.820 0.444
## 9 1 0 0 0 1 59 0.803 0.505
## 1 0 0 0 0 0 39 0.641 0.221
##
##
## $SA5_high
##
## OUT: output value
## n: number of cases in configuration
## incl: sufficiency inclusion score
## PRI: proportional reduction in inconsistency
##
## Demandm Laborm Materialm RelCommm OUT n incl PRI
## 11 1 0 1 0 1 4 0.810 0.181
## 15 1 1 1 0 0 45 0.739 0.319
## 5 0 1 0 0 0 11 0.715 0.161
## 16 1 1 1 1 0 65 0.696 0.354
## 14 1 1 0 1 0 56 0.691 0.279
## 13 1 1 0 0 0 115 0.600 0.233
## 9 1 0 0 0 0 59 0.593 0.127
## 1 0 0 0 0 0 39 0.516 0.046
##
##

## $SA6_low
##
## OUT: output value
## n: number of cases in configuration
## incl: sufficiency inclusion score
## PRI: proportional reduction in inconsistency
##
## Demandm Laborm Materialm RelCommm OUT n incl PRI
## 14 1 1 0 1 1 56 0.852 0.659
## 16 1 1 1 1 1 65 0.850 0.709
## 15 1 1 1 0 1 45 0.848 0.653
## 11 1 0 1 0 1 4 0.825 0.393
## 13 1 1 0 0 1 115 0.805 0.618
## 5 0 1 0 0 0 11 0.782 0.389
## 9 1 0 0 0 0 59 0.761 0.447
## 1 0 0 0 0 0 39 0.622 0.200
##
##
## $SA6_high
##
## OUT: output value
## n: number of cases in configuration
## incl: sufficiency inclusion score
## PRI: proportional reduction in inconsistency
##
## Demandm Laborm Materialm RelCommm OUT n incl PRI
## 11 1 0 1 0 0 4 0.756 0.168
## 15 1 1 1 0 0 45 0.712 0.333
## 5 0 1 0 0 0 11 0.699 0.176
## 16 1 1 1 1 0 65 0.694 0.386
## 14 1 1 0 1 0 56 0.664 0.265
## 13 1 1 0 0 0 115 0.570 0.226
## 9 1 0 0 0 0 59 0.562 0.120
## 1 0 0 0 0 0 39 0.496 0.046
##
## It seems that all output values have been coded to zero.
## Suggestion: lower the inclusion score for the presence of the outcome,
## the relevant argument is "incl.cut" which now has a value of 0.8.
##
##

## $SA7_low
##
## OUT: output value
## n: number of cases in configuration
## incl: sufficiency inclusion score
## PRI: proportional reduction in inconsistency
##
## Demandm Laborm Materialm RelCommm OUT n incl PRI
## 14 1 1 0 1 1 56 0.875 0.726
## 11 1 0 1 0 1 4 0.869 0.481
## 15 1 1 1 0 1 45 0.864 0.664
## 16 1 1 1 1 1 65 0.861 0.721
## 5 0 1 0 0 0 11 0.788 0.412
## 13 1 1 0 0 0 115 0.781 0.588
## 9 1 0 0 0 0 59 0.736 0.431
## 1 0 0 0 0 0 39 0.627 0.210
##
##
## $SA7_high
##
## OUT: output value
## n: number of cases in configuration
## incl: sufficiency inclusion score
## PRI: proportional reduction in inconsistency
##
## Demandm Laborm Materialm RelCommm OUT n incl PRI
## 11 1 0 1 0 0 4 0.772 0.193
## 14 1 1 0 1 0 56 0.715 0.349
## 15 1 1 1 0 0 45 0.691 0.280
## 16 1 1 1 1 0 65 0.689 0.379
## 5 0 1 0 0 0 11 0.681 0.162
## 13 1 1 0 0 0 115 0.575 0.227
## 9 1 0 0 0 0 59 0.571 0.153
## 1 0 0 0 0 0 39 0.487 0.047
##
## It seems that all output values have been coded to zero.
## Suggestion: lower the inclusion score for the presence of the outcome,
## the relevant argument is "incl.cut" which now has a value of 0.8.
##
##

## $SA8_low
##
## OUT: output value
## n: number of cases in configuration
## incl: sufficiency inclusion score
## PRI: proportional reduction in inconsistency
##
## Demandm Laborm Materialm RelCommm OUT n incl PRI
## 14 1 1 0 1 1 56 0.928 0.856
## 11 1 0 1 0 1 4 0.925 0.729
## 15 1 1 1 0 1 45 0.922 0.836
## 16 1 1 1 1 1 65 0.906 0.833
## 13 1 1 0 0 1 115 0.893 0.810
## 5 0 1 0 0 1 11 0.888 0.678
## 9 1 0 0 0 1 59 0.874 0.723
## 1 0 0 0 0 0 39 0.756 0.449
##
##
## $SA8_high
##
## OUT: output value
## n: number of cases in configuration
## incl: sufficiency inclusion score
## PRI: proportional reduction in inconsistency
##
## Demandm Laborm Materialm RelCommm OUT n incl PRI
## 11 1 0 1 0 1 4 0.856 0.435
## 15 1 1 1 0 1 45 0.832 0.587
## 14 1 1 0 1 1 56 0.829 0.599
## 16 1 1 1 1 1 65 0.816 0.610
## 5 0 1 0 0 1 11 0.804 0.420
## 13 1 1 0 0 0 115 0.737 0.484
## 9 1 0 0 0 0 59 0.702 0.318
## 1 0 0 0 0 0 39 0.600 0.172
##
##

## $SA9a_low
##
## OUT: output value
## n: number of cases in configuration
## incl: sufficiency inclusion score
## PRI: proportional reduction in inconsistency
##
## Demandm Laborm Materialm RelCommm OUT n incl PRI
## 11 1 0 1 0 1 4 0.981 0.941
## 14 1 1 0 1 1 56 0.977 0.961
## 15 1 1 1 0 1 45 0.977 0.956
## 16 1 1 1 1 1 65 0.958 0.933
## 13 1 1 0 0 1 115 0.957 0.932
## 5 0 1 0 0 1 11 0.946 0.864
## 9 1 0 0 0 1 59 0.940 0.887
## 1 0 0 0 0 1 39 0.803 0.595
##
##
## $SA9a_high
##
## OUT: output value
## n: number of cases in configuration
## incl: sufficiency inclusion score
## PRI: proportional reduction in inconsistency
##
## Demandm Laborm Materialm RelCommm OUT n incl PRI
## 11 1 0 1 0 1 4 0.941 0.771
## 14 1 1 0 1 1 56 0.926 0.851
## 15 1 1 1 0 1 45 0.915 0.811
## 16 1 1 1 1 1 65 0.908 0.829
## 5 0 1 0 0 1 11 0.894 0.669
## 13 1 1 0 0 1 115 0.863 0.749
## 9 1 0 0 0 1 59 0.839 0.630
## 1 0 0 0 0 0 39 0.692 0.309
##
##

## $SA9b_low
##
## OUT: output value
## n: number of cases in configuration
## incl: sufficiency inclusion score
## PRI: proportional reduction in inconsistency
##
## Demandm Laborm Materialm RelCommm OUT n incl PRI
## 11 1 0 1 0 1 4 0.977 0.932
## 14 1 1 0 1 1 56 0.973 0.954
## 15 1 1 1 0 1 45 0.967 0.938
## 13 1 1 0 0 1 115 0.957 0.933
## 5 0 1 0 0 1 11 0.956 0.890
## 16 1 1 1 1 1 65 0.952 0.922
## 9 1 0 0 0 1 59 0.952 0.908
## 1 0 0 0 0 1 39 0.802 0.594
##
##
## $SA9b_high
##
## OUT: output value
## n: number of cases in configuration
## incl: sufficiency inclusion score
## PRI: proportional reduction in inconsistency
##
## Demandm Laborm Materialm RelCommm OUT n incl PRI
## 11 1 0 1 0 1 4 0.939 0.769
## 14 1 1 0 1 1 56 0.925 0.844
## 15 1 1 1 0 1 45 0.915 0.811
## 5 0 1 0 0 1 11 0.899 0.680
## 16 1 1 1 1 1 65 0.895 0.801
## 13 1 1 0 0 1 115 0.879 0.775
## 9 1 0 0 0 1 59 0.846 0.645
## 1 0 0 0 0 0 39 0.693 0.324
##
##

## $SA10a_low
##
## OUT: output value
## n: number of cases in configuration
## incl: sufficiency inclusion score
## PRI: proportional reduction in inconsistency
##
## Demandm Laborm Materialm RelCommm OUT n incl PRI
## 15 1 1 1 0 1 45 0.981 0.964
## 11 1 0 1 0 1 4 0.979 0.934
## 14 1 1 0 1 1 56 0.974 0.955
## 16 1 1 1 1 1 65 0.960 0.934
## 13 1 1 0 0 1 115 0.954 0.928
## 5 0 1 0 0 1 11 0.948 0.869
## 9 1 0 0 0 1 59 0.942 0.892
## 1 0 0 0 0 1 39 0.820 0.631
##
##
## $SA10a_high
##
## OUT: output value
## n: number of cases in configuration
## incl: sufficiency inclusion score
## PRI: proportional reduction in inconsistency
##
## Demandm Laborm Materialm RelCommm OUT n incl PRI
## 11 1 0 1 0 1 4 0.926 0.735
## 14 1 1 0 1 1 56 0.921 0.838
## 15 1 1 1 0 1 45 0.918 0.822
## 5 0 1 0 0 1 11 0.894 0.684
## 16 1 1 1 1 1 65 0.887 0.790
## 13 1 1 0 0 1 115 0.867 0.758
## 9 1 0 0 0 1 59 0.840 0.639
## 1 0 0 0 0 0 39 0.701 0.354
##
##

## $SA10b_low
##
## OUT: output value
## n: number of cases in configuration
## incl: sufficiency inclusion score
## PRI: proportional reduction in inconsistency
##
## Demandm Laborm Materialm RelCommm OUT n incl PRI
## 11 1 0 1 0 1 4 0.992 0.979
## 14 1 1 0 1 1 56 0.986 0.979
## 15 1 1 1 0 1 45 0.984 0.972
## 16 1 1 1 1 1 65 0.981 0.971
## 13 1 1 0 0 1 115 0.971 0.958
## 5 0 1 0 0 1 11 0.961 0.913
## 9 1 0 0 0 1 59 0.959 0.929
## 1 0 0 0 0 1 39 0.830 0.675
##
##
## $SA10b_high
##
## OUT: output value
## n: number of cases in configuration
## incl: sufficiency inclusion score
## PRI: proportional reduction in inconsistency
##
## Demandm Laborm Materialm RelCommm OUT n incl PRI
## 11 1 0 1 0 1 4 0.961 0.875
## 14 1 1 0 1 1 56 0.958 0.923
## 15 1 1 1 0 1 45 0.948 0.897
## 16 1 1 1 1 1 65 0.947 0.908
## 5 0 1 0 0 1 11 0.937 0.822
## 13 1 1 0 0 1 115 0.920 0.865
## 9 1 0 0 0 1 59 0.876 0.743
## 1 0 0 0 0 0 39 0.731 0.428
##
##

## $SA11_low
##
## OUT: output value
## n: number of cases in configuration
## incl: sufficiency inclusion score
## PRI: proportional reduction in inconsistency
##
## Demandm Laborm Materialm RelCommm OUT n incl PRI
## 11 1 0 1 0 1 4 0.835 0.282
## 15 1 1 1 0 1 45 0.820 0.508
## 14 1 1 0 1 1 56 0.806 0.524
## 16 1 1 1 1 0 65 0.788 0.541
## 13 1 1 0 0 0 115 0.765 0.483
## 5 0 1 0 0 0 11 0.762 0.243
## 9 1 0 0 0 0 59 0.754 0.358
## 1 0 0 0 0 0 39 0.604 0.132
##
##
## $SA11_high
##
## OUT: output value
## n: number of cases in configuration
## incl: sufficiency inclusion score
## PRI: proportional reduction in inconsistency
##
## Demandm Laborm Materialm RelCommm OUT n incl PRI
## 11 1 0 1 0 0 4 0.722 0.083
## 5 0 1 0 0 0 11 0.612 0.084
## 15 1 1 1 0 0 45 0.608 0.118
## 14 1 1 0 1 0 56 0.599 0.153
## 16 1 1 1 1 0 65 0.578 0.168
## 9 1 0 0 0 0 59 0.496 0.041
## 13 1 1 0 0 0 115 0.462 0.069
## 1 0 0 0 0 0 39 0.457 0.033
##
## It seems that all output values have been coded to zero.
## Suggestion: lower the inclusion score for the presence of the outcome,
## the relevant argument is "incl.cut" which now has a value of 0.8.
##
##

## $SA12_low
##
## OUT: output value
## n: number of cases in configuration
## incl: sufficiency inclusion score
## PRI: proportional reduction in inconsistency
##
## Demandm Laborm Materialm RelCommm OUT n incl PRI
## 14 1 1 0 1 1 56 0.874 0.731
## 11 1 0 1 0 1 4 0.869 0.498
## 15 1 1 1 0 1 45 0.866 0.697
## 13 1 1 0 0 1 115 0.832 0.667
## 16 1 1 1 1 1 65 0.830 0.682
## 5 0 1 0 0 1 11 0.825 0.468
## 9 1 0 0 0 1 59 0.817 0.554
## 1 0 0 0 0 0 39 0.642 0.240
##
##
## $SA12_high
##
## OUT: output value
## n: number of cases in configuration
## incl: sufficiency inclusion score
## PRI: proportional reduction in inconsistency
##
## Demandm Laborm Materialm RelCommm OUT n incl PRI
## 11 1 0 1 0 0 4 0.782 0.229
## 15 1 1 1 0 0 45 0.728 0.386
## 14 1 1 0 1 0 56 0.724 0.395
## 5 0 1 0 0 0 11 0.697 0.221
## 16 1 1 1 1 0 65 0.695 0.405
## 13 1 1 0 0 0 115 0.596 0.266
## 9 1 0 0 0 0 59 0.594 0.174
## 1 0 0 0 0 0 39 0.514 0.104
##
## It seems that all output values have been coded to zero.
## Suggestion: lower the inclusion score for the presence of the outcome,
## the relevant argument is "incl.cut" which now has a value of 0.8.
##
##

## $SA13_low
##
## OUT: output value
## n: number of cases in configuration
## incl: sufficiency inclusion score
## PRI: proportional reduction in inconsistency
##
## Demandm Laborm Materialm RelCommm OUT n incl PRI
## 16 1 1 1 1 0 65 0.675 0.402
## 11 1 0 1 0 0 4 0.671 0.110
## 14 1 1 0 1 0 56 0.653 0.366
## 15 1 1 1 0 0 45 0.645 0.280
## 5 0 1 0 0 0 11 0.602 0.132
## 13 1 1 0 0 0 115 0.535 0.244
## 9 1 0 0 0 0 59 0.534 0.167
## 1 0 0 0 0 0 39 0.490 0.112
##
## It seems that all output values have been coded to zero.
## Suggestion: lower the inclusion score for the presence of the outcome,
## the relevant argument is "incl.cut" which now has a value of 0.8.
##
##
## $SA13_high
##
## OUT: output value
## n: number of cases in configuration
## incl: sufficiency inclusion score
## PRI: proportional reduction in inconsistency
##
## Demandm Laborm Materialm RelCommm OUT n incl PRI
## 11 1 0 1 0 0 4 0.605 0.045
## 5 0 1 0 0 0 11 0.525 0.062
## 14 1 1 0 1 0 56 0.522 0.181
## 16 1 1 1 1 0 65 0.504 0.171
## 15 1 1 1 0 0 45 0.490 0.079
## 1 0 0 0 0 0 39 0.399 0.032
## 9 1 0 0 0 0 59 0.398 0.049
## 13 1 1 0 0 0 115 0.372 0.078
##
## It seems that all output values have been coded to zero.
## Suggestion: lower the inclusion score for the presence of the outcome,
## the relevant argument is "incl.cut" which now has a value of 0.8.
##
##

## $SA14a_low
##
## OUT: output value
## n: number of cases in configuration
## incl: sufficiency inclusion score
## PRI: proportional reduction in inconsistency
##
## Demandm Laborm Materialm RelCommm OUT n incl PRI
## 15 1 1 1 0 1 45 0.887 0.708
## 16 1 1 1 1 1 65 0.883 0.753
## 11 1 0 1 0 1 4 0.879 0.435
## 14 1 1 0 1 1 56 0.872 0.712
## 13 1 1 0 0 1 115 0.827 0.650
## 5 0 1 0 0 1 11 0.823 0.408
## 9 1 0 0 0 0 59 0.795 0.488
## 1 0 0 0 0 0 39 0.670 0.272
##
##
## $SA14a_high
##
## OUT: output value
## n: number of cases in configuration
## incl: sufficiency inclusion score
## PRI: proportional reduction in inconsistency
##
## Demandm Laborm Materialm RelCommm OUT n incl PRI
## 11 1 0 1 0 0 4 0.765 0.148
## 14 1 1 0 1 0 56 0.726 0.355
## 16 1 1 1 1 0 65 0.706 0.376
## 15 1 1 1 0 0 45 0.703 0.277
## 5 0 1 0 0 0 11 0.673 0.129
## 13 1 1 0 0 0 115 0.590 0.248
## 9 1 0 0 0 0 59 0.567 0.124
## 1 0 0 0 0 0 39 0.524 0.090
##
## It seems that all output values have been coded to zero.
## Suggestion: lower the inclusion score for the presence of the outcome,
## the relevant argument is "incl.cut" which now has a value of 0.8.
##
##

## $SA14b_low
##
## OUT: output value
## n: number of cases in configuration
## incl: sufficiency inclusion score
## PRI: proportional reduction in inconsistency
##
## Demandm Laborm Materialm RelCommm OUT n incl PRI
## 14 1 1 0 1 1 56 0.963 0.927
## 11 1 0 1 0 1 4 0.963 0.860
## 15 1 1 1 0 1 45 0.953 0.898
## 16 1 1 1 1 1 65 0.952 0.913
## 13 1 1 0 0 1 115 0.926 0.863
## 5 0 1 0 0 1 11 0.903 0.692
## 9 1 0 0 0 1 59 0.895 0.758
## 1 0 0 0 0 0 39 0.717 0.380
##
##
## $SA14b_high
##
## OUT: output value
## n: number of cases in configuration
## incl: sufficiency inclusion score
## PRI: proportional reduction in inconsistency
##
## Demandm Laborm Materialm RelCommm OUT n incl PRI
## 11 1 0 1 0 1 4 0.907 0.581
## 14 1 1 0 1 1 56 0.863 0.699
## 15 1 1 1 0 1 45 0.843 0.614
## 16 1 1 1 1 1 65 0.840 0.677
## 5 0 1 0 0 0 11 0.798 0.348
## 13 1 1 0 0 0 115 0.736 0.509
## 9 1 0 0 0 0 59 0.702 0.346
## 1 0 0 0 0 0 39 0.593 0.138
##
##

## $SA14c_low
##
## OUT: output value
## n: number of cases in configuration
## incl: sufficiency inclusion score
## PRI: proportional reduction in inconsistency
##
## Demandm Laborm Materialm RelCommm OUT n incl PRI
## 14 1 1 0 1 1 56 0.968 0.945
## 11 1 0 1 0 1 4 0.964 0.886
## 15 1 1 1 0 1 45 0.959 0.920
## 16 1 1 1 1 1 65 0.959 0.931
## 13 1 1 0 0 1 115 0.940 0.901
## 5 0 1 0 0 1 11 0.916 0.780
## 9 1 0 0 0 1 59 0.903 0.808
## 1 0 0 0 0 0 39 0.735 0.461
##
##
## $SA14c_high
##
## OUT: output value
## n: number of cases in configuration
## incl: sufficiency inclusion score
## PRI: proportional reduction in inconsistency
##
## Demandm Laborm Materialm RelCommm OUT n incl PRI
## 11 1 0 1 0 1 4 0.915 0.682
## 14 1 1 0 1 1 56 0.907 0.817
## 16 1 1 1 1 1 65 0.882 0.781
## 15 1 1 1 0 1 45 0.859 0.686
## 5 0 1 0 0 1 11 0.844 0.557
## 13 1 1 0 0 1 115 0.815 0.674
## 9 1 0 0 0 0 59 0.760 0.501
## 1 0 0 0 0 0 39 0.614 0.214
##
##

## $SA15_low
##
## OUT: output value
## n: number of cases in configuration
## incl: sufficiency inclusion score
## PRI: proportional reduction in inconsistency
##
## Demandm Laborm Materialm RelCommm OUT n incl PRI
## 11 1 0 1 0 0 4 0.672 0.084
## 15 1 1 1 0 0 45 0.630 0.241
## 16 1 1 1 1 0 65 0.618 0.307
## 14 1 1 0 1 0 56 0.610 0.263
## 5 0 1 0 0 0 11 0.581 0.090
## 13 1 1 0 0 0 115 0.539 0.223
## 9 1 0 0 0 0 59 0.518 0.136
## 1 0 0 0 0 0 39 0.451 0.064
##
## It seems that all output values have been coded to zero.
## Suggestion: lower the inclusion score for the presence of the outcome,
## the relevant argument is "incl.cut" which now has a value of 0.8.
##
##
## $SA15_high
##
## OUT: output value
## n: number of cases in configuration
## incl: sufficiency inclusion score
## PRI: proportional reduction in inconsistency
##
## Demandm Laborm Materialm RelCommm OUT n incl PRI
## 11 1 0 1 0 0 4 0.590 0.028
## 5 0 1 0 0 0 11 0.505 0.045
## 15 1 1 1 0 0 45 0.470 0.065
## 16 1 1 1 1 0 65 0.454 0.097
## 14 1 1 0 1 0 56 0.453 0.080
## 1 0 0 0 0 0 39 0.380 0.015
## 9 1 0 0 0 0 59 0.372 0.023
## 13 1 1 0 0 0 115 0.338 0.043
##
## It seems that all output values have been coded to zero.
## Suggestion: lower the inclusion score for the presence of the outcome,
## the relevant argument is "incl.cut" which now has a value of 0.8.
##
##

## $SA16_low
##
## OUT: output value
## n: number of cases in configuration
## incl: sufficiency inclusion score
## PRI: proportional reduction in inconsistency
##
## Demandm Laborm Materialm RelCommm OUT n incl PRI
## 14 1 1 0 1 1 56 0.913 0.811
## 11 1 0 1 0 1 4 0.913 0.618
## 16 1 1 1 1 1 65 0.912 0.823
## 15 1 1 1 0 1 45 0.912 0.776
## 13 1 1 0 0 1 115 0.871 0.748
## 5 0 1 0 0 1 11 0.867 0.579
## 9 1 0 0 0 1 59 0.853 0.655
## 1 0 0 0 0 0 39 0.703 0.314
##
##
## $SA16_high
##
## OUT: output value
## n: number of cases in configuration
## incl: sufficiency inclusion score
## PRI: proportional reduction in inconsistency
##
## Demandm Laborm Materialm RelCommm OUT n incl PRI
## 11 1 0 1 0 1 4 0.816 0.238
## 14 1 1 0 1 0 56 0.786 0.477
## 5 0 1 0 0 0 11 0.755 0.285
## 16 1 1 1 1 0 65 0.754 0.465
## 15 1 1 1 0 0 45 0.752 0.336
## 13 1 1 0 0 0 115 0.676 0.353
## 9 1 0 0 0 0 59 0.654 0.220
## 1 0 0 0 0 0 39 0.546 0.086
##
##

## $SA17_low
##
## OUT: output value
## n: number of cases in configuration
## incl: sufficiency inclusion score
## PRI: proportional reduction in inconsistency
##
## Demandm Laborm Materialm RelCommm OUT n incl PRI
## 11 1 0 1 0 1 4 0.948 0.842
## 15 1 1 1 0 1 45 0.937 0.877
## 14 1 1 0 1 1 56 0.934 0.878
## 16 1 1 1 1 1 65 0.924 0.871
## 13 1 1 0 0 1 115 0.896 0.823
## 9 1 0 0 0 1 59 0.878 0.750
## 5 0 1 0 0 1 11 0.868 0.665
## 1 0 0 0 0 0 39 0.701 0.409
##
##
## $SA17_high
##
## OUT: output value
## n: number of cases in configuration
## incl: sufficiency inclusion score
## PRI: proportional reduction in inconsistency
##
## Demandm Laborm Materialm RelCommm OUT n incl PRI
## 11 1 0 1 0 1 4 0.889 0.621
## 15 1 1 1 0 1 45 0.850 0.668
## 14 1 1 0 1 1 56 0.845 0.677
## 16 1 1 1 1 1 65 0.830 0.671
## 5 0 1 0 0 0 11 0.792 0.468
## 13 1 1 0 0 0 115 0.756 0.566
## 9 1 0 0 0 0 59 0.710 0.405
## 1 0 0 0 0 0 39 0.589 0.217
##
##

## $SA18a_low
##
## OUT: output value
## n: number of cases in configuration
## incl: sufficiency inclusion score
## PRI: proportional reduction in inconsistency
##
## Demandm Laborm Materialm RelCommm OUT n incl PRI
## 11 1 0 1 0 1 4 0.809 0.300
## 16 1 1 1 1 0 65 0.785 0.564
## 14 1 1 0 1 0 56 0.773 0.515
## 15 1 1 1 0 0 45 0.769 0.460
## 5 0 1 0 0 0 11 0.759 0.375
## 13 1 1 0 0 0 115 0.721 0.484
## 9 1 0 0 0 0 59 0.718 0.397
## 1 0 0 0 0 0 39 0.591 0.207
##
##
## $SA18a_high
##
## OUT: output value
## n: number of cases in configuration
## incl: sufficiency inclusion score
## PRI: proportional reduction in inconsistency
##
## Demandm Laborm Materialm RelCommm OUT n incl PRI
## 11 1 0 1 0 0 4 0.710 0.135
## 5 0 1 0 0 0 11 0.657 0.182
## 14 1 1 0 1 0 56 0.610 0.229
## 15 1 1 1 0 0 45 0.587 0.143
## 16 1 1 1 1 0 65 0.582 0.214
## 9 1 0 0 0 0 59 0.539 0.129
## 13 1 1 0 0 0 115 0.507 0.179
## 1 0 0 0 0 0 39 0.498 0.095
##
## It seems that all output values have been coded to zero.
## Suggestion: lower the inclusion score for the presence of the outcome,
## the relevant argument is "incl.cut" which now has a value of 0.8.
##
##

## $SA18b_low
##
## OUT: output value
## n: number of cases in configuration
## incl: sufficiency inclusion score
## PRI: proportional reduction in inconsistency
##
## Demandm Laborm Materialm RelCommm OUT n incl PRI
## 11 1 0 1 0 0 4 0.732 0.147
## 16 1 1 1 1 0 65 0.696 0.407
## 14 1 1 0 1 0 56 0.687 0.370
## 5 0 1 0 0 0 11 0.674 0.203
## 15 1 1 1 0 0 45 0.662 0.262
## 9 1 0 0 0 0 59 0.643 0.265
## 13 1 1 0 0 0 115 0.629 0.336
## 1 0 0 0 0 0 39 0.533 0.131
##
## It seems that all output values have been coded to zero.
## Suggestion: lower the inclusion score for the presence of the outcome,
## the relevant argument is "incl.cut" which now has a value of 0.8.
##
##
## $SA18b_high
##
## OUT: output value
## n: number of cases in configuration
## incl: sufficiency inclusion score
## PRI: proportional reduction in inconsistency
##
## Demandm Laborm Materialm RelCommm OUT n incl PRI
## 11 1 0 1 0 0 4 0.635 0.065
## 5 0 1 0 0 0 11 0.579 0.079
## 14 1 1 0 1 0 56 0.522 0.137
## 15 1 1 1 0 0 45 0.494 0.075
## 16 1 1 1 1 0 65 0.492 0.133
## 9 1 0 0 0 0 59 0.462 0.050
## 1 0 0 0 0 0 39 0.442 0.038
## 13 1 1 0 0 0 115 0.410 0.080
##
## It seems that all output values have been coded to zero.
## Suggestion: lower the inclusion score for the presence of the outcome,
## the relevant argument is "incl.cut" which now has a value of 0.8.
##
##

## $SA19_low
##
## OUT: output value
## n: number of cases in configuration
## incl: sufficiency inclusion score
## PRI: proportional reduction in inconsistency
##
## Demandm Laborm Materialm RelCommm OUT n incl PRI
## 15 1 1 1 0 1 45 0.941 0.863
## 14 1 1 0 1 1 56 0.935 0.861
## 11 1 0 1 0 1 4 0.928 0.721
## 13 1 1 0 0 1 115 0.911 0.832
## 16 1 1 1 1 1 65 0.910 0.827
## 5 0 1 0 0 1 11 0.885 0.656
## 9 1 0 0 0 1 59 0.871 0.698
## 1 0 0 0 0 0 39 0.743 0.414
##
##
## $SA19_high
##
## OUT: output value
## n: number of cases in configuration
## incl: sufficiency inclusion score
## PRI: proportional reduction in inconsistency
##
## Demandm Laborm Materialm RelCommm OUT n incl PRI
## 11 1 0 1 0 1 4 0.864 0.394
## 15 1 1 1 0 1 45 0.800 0.494
## 14 1 1 0 1 0 56 0.798 0.521
## 5 0 1 0 0 0 11 0.796 0.349
## 16 1 1 1 1 0 65 0.775 0.544
## 13 1 1 0 0 0 115 0.721 0.444
## 9 1 0 0 0 0 59 0.700 0.293
## 1 0 0 0 0 0 39 0.584 0.122
##
##

## $SA20_low
##
## OUT: output value
## n: number of cases in configuration
## incl: sufficiency inclusion score
## PRI: proportional reduction in inconsistency
##
## Demandm Laborm Materialm RelCommm OUT n incl PRI
## 16 1 1 1 1 1 65 0.830 0.650
## 15 1 1 1 0 1 45 0.827 0.576
## 14 1 1 0 1 1 56 0.825 0.586
## 11 1 0 1 0 0 4 0.799 0.296
## 5 0 1 0 0 0 11 0.775 0.306
## 13 1 1 0 0 0 115 0.767 0.522
## 9 1 0 0 0 0 59 0.714 0.326
## 1 0 0 0 0 0 39 0.613 0.170
##
##
## $SA20_high
##
## OUT: output value
## n: number of cases in configuration
## incl: sufficiency inclusion score
## PRI: proportional reduction in inconsistency
##
## Demandm Laborm Materialm RelCommm OUT n incl PRI
## 11 1 0 1 0 0 4 0.748 0.092
## 15 1 1 1 0 0 45 0.667 0.181
## 16 1 1 1 1 0 65 0.661 0.291
## 5 0 1 0 0 0 11 0.645 0.088
## 14 1 1 0 1 0 56 0.641 0.192
## 9 1 0 0 0 0 59 0.514 0.048
## 13 1 1 0 0 0 115 0.512 0.116
## 1 0 0 0 0 0 39 0.472 0.022
##
## It seems that all output values have been coded to zero.
## Suggestion: lower the inclusion score for the presence of the outcome,
## the relevant argument is "incl.cut" which now has a value of 0.8.
##
##

## $SA21_low
##
## OUT: output value
## n: number of cases in configuration
## incl: sufficiency inclusion score
## PRI: proportional reduction in inconsistency
##
## Demandm Laborm Materialm RelCommm OUT n incl PRI
## 15 1 1 1 0 1 45 0.944 0.878
## 11 1 0 1 0 1 4 0.940 0.784
## 14 1 1 0 1 1 56 0.933 0.867
## 16 1 1 1 1 1 65 0.929 0.872
## 13 1 1 0 0 1 115 0.908 0.835
## 5 0 1 0 0 1 11 0.891 0.685
## 9 1 0 0 0 1 59 0.884 0.744
## 1 0 0 0 0 0 39 0.729 0.412
##
##
## $SA21_high
##
## OUT: output value
## n: number of cases in configuration
## incl: sufficiency inclusion score
## PRI: proportional reduction in inconsistency
##
## Demandm Laborm Materialm RelCommm OUT n incl PRI
## 11 1 0 1 0 1 4 0.866 0.497
## 14 1 1 0 1 1 56 0.839 0.618
## 15 1 1 1 0 1 45 0.825 0.586
## 16 1 1 1 1 1 65 0.823 0.641
## 5 0 1 0 0 1 11 0.803 0.387
## 13 1 1 0 0 0 115 0.753 0.518
## 9 1 0 0 0 0 59 0.722 0.368
## 1 0 0 0 0 0 39 0.604 0.163
##
##

## $SA22_low
##
## OUT: output value
## n: number of cases in configuration
## incl: sufficiency inclusion score
## PRI: proportional reduction in inconsistency
##
## Demandm Laborm Materialm RelCommm OUT n incl PRI
## 14 1 1 0 1 1 56 0.958 0.928
## 15 1 1 1 0 1 45 0.948 0.910
## 13 1 1 0 0 1 115 0.944 0.911
## 11 1 0 1 0 1 4 0.941 0.853
## 16 1 1 1 1 1 65 0.938 0.901
## 9 1 0 0 0 1 59 0.909 0.831
## 5 0 1 0 0 1 11 0.908 0.779
## 1 0 0 0 0 0 39 0.742 0.494
##
##
## $SA22_high
##
## OUT: output value
## n: number of cases in configuration
## incl: sufficiency inclusion score
## PRI: proportional reduction in inconsistency
##
## Demandm Laborm Materialm RelCommm OUT n incl PRI
## 11 1 0 1 0 1 4 0.913 0.752
## 15 1 1 1 0 1 45 0.908 0.824
## 16 1 1 1 1 1 65 0.870 0.781
## 14 1 1 0 1 1 56 0.866 0.757
## 5 0 1 0 0 1 11 0.824 0.577
## 13 1 1 0 0 1 115 0.811 0.689
## 9 1 0 0 0 0 59 0.770 0.564
## 1 0 0 0 0 0 39 0.620 0.277
##
##

## $SA23_low
##
## OUT: output value
## n: number of cases in configuration
## incl: sufficiency inclusion score
## PRI: proportional reduction in inconsistency
##
## Demandm Laborm Materialm RelCommm OUT n incl PRI
## 11 1 0 1 0 1 4 0.850 0.339
## 16 1 1 1 1 1 65 0.845 0.674
## 15 1 1 1 0 1 45 0.839 0.575
## 14 1 1 0 1 1 56 0.836 0.601
## 13 1 1 0 0 0 115 0.796 0.557
## 5 0 1 0 0 0 11 0.778 0.268
## 9 1 0 0 0 0 59 0.775 0.406
## 1 0 0 0 0 0 39 0.599 0.123
##
##
## $SA23_high
##
## OUT: output value
## n: number of cases in configuration
## incl: sufficiency inclusion score
## PRI: proportional reduction in inconsistency
##
## Demandm Laborm Materialm RelCommm OUT n incl PRI
## 11 1 0 1 0 0 4 0.742 0.060
## 16 1 1 1 1 0 65 0.658 0.264
## 14 1 1 0 1 0 56 0.647 0.195
## 15 1 1 1 0 0 45 0.642 0.137
## 5 0 1 0 0 0 11 0.639 0.064
## 9 1 0 0 0 0 59 0.529 0.053
## 13 1 1 0 0 0 115 0.519 0.118
## 1 0 0 0 0 0 39 0.475 0.012
##
## It seems that all output values have been coded to zero.
## Suggestion: lower the inclusion score for the presence of the outcome,
## the relevant argument is "incl.cut" which now has a value of 0.8.
##
##

## $SA24_low
##
## OUT: output value
## n: number of cases in configuration
## incl: sufficiency inclusion score
## PRI: proportional reduction in inconsistency
##
## Demandm Laborm Materialm RelCommm OUT n incl PRI
## 11 1 0 1 0 1 4 0.802 0.212
## 15 1 1 1 0 0 45 0.773 0.442
## 16 1 1 1 1 0 65 0.773 0.534
## 5 0 1 0 0 0 11 0.761 0.245
## 14 1 1 0 1 0 56 0.755 0.437
## 9 1 0 0 0 0 59 0.706 0.331
## 13 1 1 0 0 0 115 0.700 0.399
## 1 0 0 0 0 0 39 0.596 0.125
##
##
## $SA24_high
##
## OUT: output value
## n: number of cases in configuration
## incl: sufficiency inclusion score
## PRI: proportional reduction in inconsistency
##
## Demandm Laborm Materialm RelCommm OUT n incl PRI
## 11 1 0 1 0 0 4 0.697 0.038
## 5 0 1 0 0 0 11 0.627 0.044
## 16 1 1 1 1 0 65 0.592 0.184
## 15 1 1 1 0 0 45 0.590 0.080
## 14 1 1 0 1 0 56 0.557 0.100
## 9 1 0 0 0 0 59 0.504 0.063
## 1 0 0 0 0 0 39 0.465 0.014
## 13 1 1 0 0 0 115 0.456 0.073
##
## It seems that all output values have been coded to zero.
## Suggestion: lower the inclusion score for the presence of the outcome,
## the relevant argument is "incl.cut" which now has a value of 0.8.
##
##

## $SA25_low
##
## OUT: output value
## n: number of cases in configuration
## incl: sufficiency inclusion score
## PRI: proportional reduction in inconsistency
##
## Demandm Laborm Materialm RelCommm OUT n incl PRI
## 11 1 0 1 0 1 4 0.962 0.851
## 14 1 1 0 1 1 56 0.958 0.920
## 15 1 1 1 0 1 45 0.948 0.889
## 13 1 1 0 0 1 115 0.942 0.901
## 16 1 1 1 1 1 65 0.940 0.893
## 9 1 0 0 0 1 59 0.923 0.834
## 5 0 1 0 0 1 11 0.911 0.749
## 1 0 0 0 0 0 39 0.754 0.465
##
##
## $SA25_high
##
## OUT: output value
## n: number of cases in configuration
## incl: sufficiency inclusion score
## PRI: proportional reduction in inconsistency
##
## Demandm Laborm Materialm RelCommm OUT n incl PRI
## 11 1 0 1 0 1 4 0.893 0.507
## 14 1 1 0 1 1 56 0.885 0.736
## 15 1 1 1 0 1 45 0.853 0.627
## 16 1 1 1 1 1 65 0.852 0.700
## 5 0 1 0 0 1 11 0.834 0.477
## 13 1 1 0 0 1 115 0.805 0.608
## 9 1 0 0 0 0 59 0.775 0.448
## 1 0 0 0 0 0 39 0.627 0.190

# Result: Truth Table: Reasons for MNC triggering single Outcomes of MNC (for n.cut = 9)

## $SA1_low
##
## OUT: output value
## n: number of cases in configuration
## incl: sufficiency inclusion score
## PRI: proportional reduction in inconsistency
##
## Demandm Laborm Materialm RelCommm OUT n incl PRI
## 14 1 1 0 1 1 56 0.970 0.939
## 15 1 1 1 0 1 45 0.945 0.882
## 13 1 1 0 0 1 115 0.940 0.892
## 16 1 1 1 1 1 65 0.930 0.873
## 5 0 1 0 0 1 11 0.903 0.721
## 9 1 0 0 0 1 59 0.889 0.759
## 1 0 0 0 0 0 39 0.729 0.419
##
##
## $SA1_high
##
## OUT: output value
## n: number of cases in configuration
## incl: sufficiency inclusion score
## PRI: proportional reduction in inconsistency
##
## Demandm Laborm Materialm RelCommm OUT n incl PRI
## 14 1 1 0 1 1 56 0.878 0.704
## 15 1 1 1 0 1 45 0.849 0.612
## 5 0 1 0 0 1 11 0.826 0.448
## 16 1 1 1 1 1 65 0.820 0.602
## 13 1 1 0 0 0 115 0.786 0.570
## 9 1 0 0 0 0 59 0.747 0.397
## 1 0 0 0 0 0 39 0.611 0.155
##
##

## $SA2_low
##
## OUT: output value
## n: number of cases in configuration
## incl: sufficiency inclusion score
## PRI: proportional reduction in inconsistency
##
## Demandm Laborm Materialm RelCommm OUT n incl PRI
## 14 1 1 0 1 1 56 0.813 0.590
## 15 1 1 1 0 1 45 0.803 0.529
## 5 0 1 0 0 0 11 0.798 0.387
## 16 1 1 1 1 0 65 0.785 0.554
## 13 1 1 0 0 0 115 0.754 0.519
## 9 1 0 0 0 0 59 0.739 0.394
## 1 0 0 0 0 0 39 0.621 0.189
##
##
## $SA2_high
##
## OUT: output value
## n: number of cases in configuration
## incl: sufficiency inclusion score
## PRI: proportional reduction in inconsistency
##
## Demandm Laborm Materialm RelCommm OUT n incl PRI
## 5 0 1 0 0 0 11 0.669 0.117
## 14 1 1 0 1 0 56 0.646 0.252
## 15 1 1 1 0 0 45 0.622 0.175
## 16 1 1 1 1 0 65 0.593 0.212
## 9 1 0 0 0 0 59 0.535 0.087
## 13 1 1 0 0 0 115 0.515 0.148
## 1 0 0 0 0 0 39 0.492 0.031
##
## It seems that all output values have been coded to zero.
## Suggestion: lower the inclusion score for the presence of the outcome,
## the relevant argument is "incl.cut" which now has a value of 0.8.
##
##

## $SA3_low
##
## OUT: output value
## n: number of cases in configuration
## incl: sufficiency inclusion score
## PRI: proportional reduction in inconsistency
##
## Demandm Laborm Materialm RelCommm OUT n incl PRI
## 15 1 1 1 0 1 45 0.845 0.632
## 16 1 1 1 1 1 65 0.817 0.640
## 14 1 1 0 1 0 56 0.778 0.555
## 13 1 1 0 0 0 115 0.734 0.525
## 5 0 1 0 0 0 11 0.719 0.292
## 9 1 0 0 0 0 59 0.702 0.381
## 1 0 0 0 0 0 39 0.553 0.149
##
##
## $SA3_high
##
## OUT: output value
## n: number of cases in configuration
## incl: sufficiency inclusion score
## PRI: proportional reduction in inconsistency
##
## Demandm Laborm Materialm RelCommm OUT n incl PRI
## 15 1 1 1 0 0 45 0.684 0.261
## 14 1 1 0 1 0 56 0.655 0.296
## 16 1 1 1 1 0 65 0.654 0.324
## 5 0 1 0 0 0 11 0.638 0.120
## 13 1 1 0 0 0 115 0.547 0.207
## 9 1 0 0 0 0 59 0.538 0.115
## 1 0 0 0 0 0 39 0.466 0.040
##
## It seems that all output values have been coded to zero.
## Suggestion: lower the inclusion score for the presence of the outcome,
## the relevant argument is "incl.cut" which now has a value of 0.8.
##
##

## $SA4_low
##
## OUT: output value
## n: number of cases in configuration
## incl: sufficiency inclusion score
## PRI: proportional reduction in inconsistency
##
## Demandm Laborm Materialm RelCommm OUT n incl PRI
## 15 1 1 1 0 0 45 0.796 0.507
## 14 1 1 0 1 0 56 0.790 0.516
## 16 1 1 1 1 0 65 0.768 0.528
## 13 1 1 0 0 0 115 0.700 0.419
## 5 0 1 0 0 0 11 0.692 0.220
## 9 1 0 0 0 0 59 0.659 0.267
## 1 0 0 0 0 0 39 0.543 0.122
##
## It seems that all output values have been coded to zero.
## Suggestion: lower the inclusion score for the presence of the outcome,
## the relevant argument is "incl.cut" which now has a value of 0.8.
##
##
## $SA4_high
##
## OUT: output value
## n: number of cases in configuration
## incl: sufficiency inclusion score
## PRI: proportional reduction in inconsistency
##
## Demandm Laborm Materialm RelCommm OUT n incl PRI
## 15 1 1 1 0 0 45 0.609 0.157
## 5 0 1 0 0 0 11 0.595 0.084
## 16 1 1 1 1 0 65 0.590 0.222
## 14 1 1 0 1 0 56 0.580 0.146
## 9 1 0 0 0 0 59 0.478 0.059
## 13 1 1 0 0 0 115 0.460 0.095
## 1 0 0 0 0 0 39 0.438 0.032
##
## It seems that all output values have been coded to zero.
## Suggestion: lower the inclusion score for the presence of the outcome,
## the relevant argument is "incl.cut" which now has a value of 0.8.
##
##

## $SA5_low
##
## OUT: output value
## n: number of cases in configuration
## incl: sufficiency inclusion score
## PRI: proportional reduction in inconsistency
##
## Demandm Laborm Materialm RelCommm OUT n incl PRI
## 15 1 1 1 0 1 45 0.899 0.747
## 14 1 1 0 1 1 56 0.875 0.702
## 16 1 1 1 1 1 65 0.868 0.730
## 13 1 1 0 0 1 115 0.836 0.663
## 5 0 1 0 0 1 11 0.820 0.444
## 9 1 0 0 0 1 59 0.803 0.505
## 1 0 0 0 0 0 39 0.641 0.221
##
##
## $SA5_high
##
## OUT: output value
## n: number of cases in configuration
## incl: sufficiency inclusion score
## PRI: proportional reduction in inconsistency
##
## Demandm Laborm Materialm RelCommm OUT n incl PRI
## 15 1 1 1 0 0 45 0.739 0.319
## 5 0 1 0 0 0 11 0.715 0.161
## 16 1 1 1 1 0 65 0.696 0.354
## 14 1 1 0 1 0 56 0.691 0.279
## 13 1 1 0 0 0 115 0.600 0.233
## 9 1 0 0 0 0 59 0.593 0.127
## 1 0 0 0 0 0 39 0.516 0.046
##
## It seems that all output values have been coded to zero.
## Suggestion: lower the inclusion score for the presence of the outcome,
## the relevant argument is "incl.cut" which now has a value of 0.8.
##
##

## $SA6_low
##
## OUT: output value
## n: number of cases in configuration
## incl: sufficiency inclusion score
## PRI: proportional reduction in inconsistency
##
## Demandm Laborm Materialm RelCommm OUT n incl PRI
## 14 1 1 0 1 1 56 0.852 0.659
## 16 1 1 1 1 1 65 0.850 0.709
## 15 1 1 1 0 1 45 0.848 0.653
## 13 1 1 0 0 1 115 0.805 0.618
## 5 0 1 0 0 0 11 0.782 0.389
## 9 1 0 0 0 0 59 0.761 0.447
## 1 0 0 0 0 0 39 0.622 0.200
##
##
## $SA6_high
##
## OUT: output value
## n: number of cases in configuration
## incl: sufficiency inclusion score
## PRI: proportional reduction in inconsistency
##
## Demandm Laborm Materialm RelCommm OUT n incl PRI
## 15 1 1 1 0 0 45 0.712 0.333
## 5 0 1 0 0 0 11 0.699 0.176
## 16 1 1 1 1 0 65 0.694 0.386
## 14 1 1 0 1 0 56 0.664 0.265
## 13 1 1 0 0 0 115 0.570 0.226
## 9 1 0 0 0 0 59 0.562 0.120
## 1 0 0 0 0 0 39 0.496 0.046
##
## It seems that all output values have been coded to zero.
## Suggestion: lower the inclusion score for the presence of the outcome,
## the relevant argument is "incl.cut" which now has a value of 0.8.
##
##

## $SA7_low
##
## OUT: output value
## n: number of cases in configuration
## incl: sufficiency inclusion score
## PRI: proportional reduction in inconsistency
##
## Demandm Laborm Materialm RelCommm OUT n incl PRI
## 14 1 1 0 1 1 56 0.875 0.726
## 15 1 1 1 0 1 45 0.864 0.664
## 16 1 1 1 1 1 65 0.861 0.721
## 5 0 1 0 0 0 11 0.788 0.412
## 13 1 1 0 0 0 115 0.781 0.588
## 9 1 0 0 0 0 59 0.736 0.431
## 1 0 0 0 0 0 39 0.627 0.210
##
##
## $SA7_high
##
## OUT: output value
## n: number of cases in configuration
## incl: sufficiency inclusion score
## PRI: proportional reduction in inconsistency
##
## Demandm Laborm Materialm RelCommm OUT n incl PRI
## 14 1 1 0 1 0 56 0.715 0.349
## 15 1 1 1 0 0 45 0.691 0.280
## 16 1 1 1 1 0 65 0.689 0.379
## 5 0 1 0 0 0 11 0.681 0.162
## 13 1 1 0 0 0 115 0.575 0.227
## 9 1 0 0 0 0 59 0.571 0.153
## 1 0 0 0 0 0 39 0.487 0.047
##
## It seems that all output values have been coded to zero.
## Suggestion: lower the inclusion score for the presence of the outcome,
## the relevant argument is "incl.cut" which now has a value of 0.8.
##
##

## $SA8_low
##
## OUT: output value
## n: number of cases in configuration
## incl: sufficiency inclusion score
## PRI: proportional reduction in inconsistency
##
## Demandm Laborm Materialm RelCommm OUT n incl PRI
## 14 1 1 0 1 1 56 0.928 0.856
## 15 1 1 1 0 1 45 0.922 0.836
## 16 1 1 1 1 1 65 0.906 0.833
## 13 1 1 0 0 1 115 0.893 0.810
## 5 0 1 0 0 1 11 0.888 0.678
## 9 1 0 0 0 1 59 0.874 0.723
## 1 0 0 0 0 0 39 0.756 0.449
##
##
## $SA8_high
##
## OUT: output value
## n: number of cases in configuration
## incl: sufficiency inclusion score
## PRI: proportional reduction in inconsistency
##
## Demandm Laborm Materialm RelCommm OUT n incl PRI
## 15 1 1 1 0 1 45 0.832 0.587
## 14 1 1 0 1 1 56 0.829 0.599
## 16 1 1 1 1 1 65 0.816 0.610
## 5 0 1 0 0 1 11 0.804 0.420
## 13 1 1 0 0 0 115 0.737 0.484
## 9 1 0 0 0 0 59 0.702 0.318
## 1 0 0 0 0 0 39 0.600 0.172
##
##

## $SA9a_low
##
## OUT: output value
## n: number of cases in configuration
## incl: sufficiency inclusion score
## PRI: proportional reduction in inconsistency
##
## Demandm Laborm Materialm RelCommm OUT n incl PRI
## 14 1 1 0 1 1 56 0.977 0.961
## 15 1 1 1 0 1 45 0.977 0.956
## 16 1 1 1 1 1 65 0.958 0.933
## 13 1 1 0 0 1 115 0.957 0.932
## 5 0 1 0 0 1 11 0.946 0.864
## 9 1 0 0 0 1 59 0.940 0.887
## 1 0 0 0 0 1 39 0.803 0.595
##
##
## $SA9a_high
##
## OUT: output value
## n: number of cases in configuration
## incl: sufficiency inclusion score
## PRI: proportional reduction in inconsistency
##
## Demandm Laborm Materialm RelCommm OUT n incl PRI
## 14 1 1 0 1 1 56 0.926 0.851
## 15 1 1 1 0 1 45 0.915 0.811
## 16 1 1 1 1 1 65 0.908 0.829
## 5 0 1 0 0 1 11 0.894 0.669
## 13 1 1 0 0 1 115 0.863 0.749
## 9 1 0 0 0 1 59 0.839 0.630
## 1 0 0 0 0 0 39 0.692 0.309
##
##

## $SA9b_low
##
## OUT: output value
## n: number of cases in configuration
## incl: sufficiency inclusion score
## PRI: proportional reduction in inconsistency
##
## Demandm Laborm Materialm RelCommm OUT n incl PRI
## 14 1 1 0 1 1 56 0.973 0.954
## 15 1 1 1 0 1 45 0.967 0.938
## 13 1 1 0 0 1 115 0.957 0.933
## 5 0 1 0 0 1 11 0.956 0.890
## 16 1 1 1 1 1 65 0.952 0.922
## 9 1 0 0 0 1 59 0.952 0.908
## 1 0 0 0 0 1 39 0.802 0.594
##
##
## $SA9b_high
##
## OUT: output value
## n: number of cases in configuration
## incl: sufficiency inclusion score
## PRI: proportional reduction in inconsistency
##
## Demandm Laborm Materialm RelCommm OUT n incl PRI
## 14 1 1 0 1 1 56 0.925 0.844
## 15 1 1 1 0 1 45 0.915 0.811
## 5 0 1 0 0 1 11 0.899 0.680
## 16 1 1 1 1 1 65 0.895 0.801
## 13 1 1 0 0 1 115 0.879 0.775
## 9 1 0 0 0 1 59 0.846 0.645
## 1 0 0 0 0 0 39 0.693 0.324
##
##

## $SA10a_low
##
## OUT: output value
## n: number of cases in configuration
## incl: sufficiency inclusion score
## PRI: proportional reduction in inconsistency
##
## Demandm Laborm Materialm RelCommm OUT n incl PRI
## 15 1 1 1 0 1 45 0.981 0.964
## 14 1 1 0 1 1 56 0.974 0.955
## 16 1 1 1 1 1 65 0.960 0.934
## 13 1 1 0 0 1 115 0.954 0.928
## 5 0 1 0 0 1 11 0.948 0.869
## 9 1 0 0 0 1 59 0.942 0.892
## 1 0 0 0 0 1 39 0.820 0.631
##
##
## $SA10a_high
##
## OUT: output value
## n: number of cases in configuration
## incl: sufficiency inclusion score
## PRI: proportional reduction in inconsistency
##
## Demandm Laborm Materialm RelCommm OUT n incl PRI
## 14 1 1 0 1 1 56 0.921 0.838
## 15 1 1 1 0 1 45 0.918 0.822
## 5 0 1 0 0 1 11 0.894 0.684
## 16 1 1 1 1 1 65 0.887 0.790
## 13 1 1 0 0 1 115 0.867 0.758
## 9 1 0 0 0 1 59 0.840 0.639
## 1 0 0 0 0 0 39 0.701 0.354
##
##

## $SA10b_low
##
## OUT: output value
## n: number of cases in configuration
## incl: sufficiency inclusion score
## PRI: proportional reduction in inconsistency
##
## Demandm Laborm Materialm RelCommm OUT n incl PRI
## 14 1 1 0 1 1 56 0.986 0.979
## 15 1 1 1 0 1 45 0.984 0.972
## 16 1 1 1 1 1 65 0.981 0.971
## 13 1 1 0 0 1 115 0.971 0.958
## 5 0 1 0 0 1 11 0.961 0.913
## 9 1 0 0 0 1 59 0.959 0.929
## 1 0 0 0 0 1 39 0.830 0.675
##
##
## $SA10b_high
##
## OUT: output value
## n: number of cases in configuration
## incl: sufficiency inclusion score
## PRI: proportional reduction in inconsistency
##
## Demandm Laborm Materialm RelCommm OUT n incl PRI
## 14 1 1 0 1 1 56 0.958 0.923
## 15 1 1 1 0 1 45 0.948 0.897
## 16 1 1 1 1 1 65 0.947 0.908
## 5 0 1 0 0 1 11 0.937 0.822
## 13 1 1 0 0 1 115 0.920 0.865
## 9 1 0 0 0 1 59 0.876 0.743
## 1 0 0 0 0 0 39 0.731 0.428
##
##

## $SA11_low
##
## OUT: output value
## n: number of cases in configuration
## incl: sufficiency inclusion score
## PRI: proportional reduction in inconsistency
##
## Demandm Laborm Materialm RelCommm OUT n incl PRI
## 15 1 1 1 0 1 45 0.820 0.508
## 14 1 1 0 1 1 56 0.806 0.524
## 16 1 1 1 1 0 65 0.788 0.541
## 13 1 1 0 0 0 115 0.765 0.483
## 5 0 1 0 0 0 11 0.762 0.243
## 9 1 0 0 0 0 59 0.754 0.358
## 1 0 0 0 0 0 39 0.604 0.132
##
##
## $SA11_high
##
## OUT: output value
## n: number of cases in configuration
## incl: sufficiency inclusion score
## PRI: proportional reduction in inconsistency
##
## Demandm Laborm Materialm RelCommm OUT n incl PRI
## 5 0 1 0 0 0 11 0.612 0.084
## 15 1 1 1 0 0 45 0.608 0.118
## 14 1 1 0 1 0 56 0.599 0.153
## 16 1 1 1 1 0 65 0.578 0.168
## 9 1 0 0 0 0 59 0.496 0.041
## 13 1 1 0 0 0 115 0.462 0.069
## 1 0 0 0 0 0 39 0.457 0.033
##
## It seems that all output values have been coded to zero.
## Suggestion: lower the inclusion score for the presence of the outcome,
## the relevant argument is "incl.cut" which now has a value of 0.8.
##
##

## $SA12_low
##
## OUT: output value
## n: number of cases in configuration
## incl: sufficiency inclusion score
## PRI: proportional reduction in inconsistency
##
## Demandm Laborm Materialm RelCommm OUT n incl PRI
## 14 1 1 0 1 1 56 0.874 0.731
## 15 1 1 1 0 1 45 0.866 0.697
## 13 1 1 0 0 1 115 0.832 0.667
## 16 1 1 1 1 1 65 0.830 0.682
## 5 0 1 0 0 1 11 0.825 0.468
## 9 1 0 0 0 1 59 0.817 0.554
## 1 0 0 0 0 0 39 0.642 0.240
##
##
## $SA12_high
##
## OUT: output value
## n: number of cases in configuration
## incl: sufficiency inclusion score
## PRI: proportional reduction in inconsistency
##
## Demandm Laborm Materialm RelCommm OUT n incl PRI
## 15 1 1 1 0 0 45 0.728 0.386
## 14 1 1 0 1 0 56 0.724 0.395
## 5 0 1 0 0 0 11 0.697 0.221
## 16 1 1 1 1 0 65 0.695 0.405
## 13 1 1 0 0 0 115 0.596 0.266
## 9 1 0 0 0 0 59 0.594 0.174
## 1 0 0 0 0 0 39 0.514 0.104
##
## It seems that all output values have been coded to zero.
## Suggestion: lower the inclusion score for the presence of the outcome,
## the relevant argument is "incl.cut" which now has a value of 0.8.
##
##

## $SA13_low
##
## OUT: output value
## n: number of cases in configuration
## incl: sufficiency inclusion score
## PRI: proportional reduction in inconsistency
##
## Demandm Laborm Materialm RelCommm OUT n incl PRI
## 16 1 1 1 1 0 65 0.675 0.402
## 14 1 1 0 1 0 56 0.653 0.366
## 15 1 1 1 0 0 45 0.645 0.280
## 5 0 1 0 0 0 11 0.602 0.132
## 13 1 1 0 0 0 115 0.535 0.244
## 9 1 0 0 0 0 59 0.534 0.167
## 1 0 0 0 0 0 39 0.490 0.112
##
## It seems that all output values have been coded to zero.
## Suggestion: lower the inclusion score for the presence of the outcome,
## the relevant argument is "incl.cut" which now has a value of 0.8.
##
##
## $SA13_high
##
## OUT: output value
## n: number of cases in configuration
## incl: sufficiency inclusion score
## PRI: proportional reduction in inconsistency
##
## Demandm Laborm Materialm RelCommm OUT n incl PRI
## 5 0 1 0 0 0 11 0.525 0.062
## 14 1 1 0 1 0 56 0.522 0.181
## 16 1 1 1 1 0 65 0.504 0.171
## 15 1 1 1 0 0 45 0.490 0.079
## 1 0 0 0 0 0 39 0.399 0.032
## 9 1 0 0 0 0 59 0.398 0.049
## 13 1 1 0 0 0 115 0.372 0.078
##
## It seems that all output values have been coded to zero.
## Suggestion: lower the inclusion score for the presence of the outcome,
## the relevant argument is "incl.cut" which now has a value of 0.8.
##
##

## $SA14a_low
##
## OUT: output value
## n: number of cases in configuration
## incl: sufficiency inclusion score
## PRI: proportional reduction in inconsistency
##
## Demandm Laborm Materialm RelCommm OUT n incl PRI
## 15 1 1 1 0 1 45 0.887 0.708
## 16 1 1 1 1 1 65 0.883 0.753
## 14 1 1 0 1 1 56 0.872 0.712
## 13 1 1 0 0 1 115 0.827 0.650
## 5 0 1 0 0 1 11 0.823 0.408
## 9 1 0 0 0 0 59 0.795 0.488
## 1 0 0 0 0 0 39 0.670 0.272
##
##
## $SA14a_high
##
## OUT: output value
## n: number of cases in configuration
## incl: sufficiency inclusion score
## PRI: proportional reduction in inconsistency
##
## Demandm Laborm Materialm RelCommm OUT n incl PRI
## 14 1 1 0 1 0 56 0.726 0.355
## 16 1 1 1 1 0 65 0.706 0.376
## 15 1 1 1 0 0 45 0.703 0.277
## 5 0 1 0 0 0 11 0.673 0.129
## 13 1 1 0 0 0 115 0.590 0.248
## 9 1 0 0 0 0 59 0.567 0.124
## 1 0 0 0 0 0 39 0.524 0.090
##
## It seems that all output values have been coded to zero.
## Suggestion: lower the inclusion score for the presence of the outcome,
## the relevant argument is "incl.cut" which now has a value of 0.8.
##
##

## $SA14b_low
##
## OUT: output value
## n: number of cases in configuration
## incl: sufficiency inclusion score
## PRI: proportional reduction in inconsistency
##
## Demandm Laborm Materialm RelCommm OUT n incl PRI
## 14 1 1 0 1 1 56 0.963 0.927
## 15 1 1 1 0 1 45 0.953 0.898
## 16 1 1 1 1 1 65 0.952 0.913
## 13 1 1 0 0 1 115 0.926 0.863
## 5 0 1 0 0 1 11 0.903 0.692
## 9 1 0 0 0 1 59 0.895 0.758
## 1 0 0 0 0 0 39 0.717 0.380
##
##
## $SA14b_high
##
## OUT: output value
## n: number of cases in configuration
## incl: sufficiency inclusion score
## PRI: proportional reduction in inconsistency
##
## Demandm Laborm Materialm RelCommm OUT n incl PRI
## 14 1 1 0 1 1 56 0.863 0.699
## 15 1 1 1 0 1 45 0.843 0.614
## 16 1 1 1 1 1 65 0.840 0.677
## 5 0 1 0 0 0 11 0.798 0.348
## 13 1 1 0 0 0 115 0.736 0.509
## 9 1 0 0 0 0 59 0.702 0.346
## 1 0 0 0 0 0 39 0.593 0.138
##
##

## $SA14c_low
##
## OUT: output value
## n: number of cases in configuration
## incl: sufficiency inclusion score
## PRI: proportional reduction in inconsistency
##
## Demandm Laborm Materialm RelCommm OUT n incl PRI
## 14 1 1 0 1 1 56 0.968 0.945
## 15 1 1 1 0 1 45 0.959 0.920
## 16 1 1 1 1 1 65 0.959 0.931
## 13 1 1 0 0 1 115 0.940 0.901
## 5 0 1 0 0 1 11 0.916 0.780
## 9 1 0 0 0 1 59 0.903 0.808
## 1 0 0 0 0 0 39 0.735 0.461
##
##
## $SA14c_high
##
## OUT: output value
## n: number of cases in configuration
## incl: sufficiency inclusion score
## PRI: proportional reduction in inconsistency
##
## Demandm Laborm Materialm RelCommm OUT n incl PRI
## 14 1 1 0 1 1 56 0.907 0.817
## 16 1 1 1 1 1 65 0.882 0.781
## 15 1 1 1 0 1 45 0.859 0.686
## 5 0 1 0 0 1 11 0.844 0.557
## 13 1 1 0 0 1 115 0.815 0.674
## 9 1 0 0 0 0 59 0.760 0.501
## 1 0 0 0 0 0 39 0.614 0.214
##
##

## $SA15_low
##
## OUT: output value
## n: number of cases in configuration
## incl: sufficiency inclusion score
## PRI: proportional reduction in inconsistency
##
## Demandm Laborm Materialm RelCommm OUT n incl PRI
## 15 1 1 1 0 0 45 0.630 0.241
## 16 1 1 1 1 0 65 0.618 0.307
## 14 1 1 0 1 0 56 0.610 0.263
## 5 0 1 0 0 0 11 0.581 0.090
## 13 1 1 0 0 0 115 0.539 0.223
## 9 1 0 0 0 0 59 0.518 0.136
## 1 0 0 0 0 0 39 0.451 0.064
##
## It seems that all output values have been coded to zero.
## Suggestion: lower the inclusion score for the presence of the outcome,
## the relevant argument is "incl.cut" which now has a value of 0.8.
##
##
## $SA15_high
##
## OUT: output value
## n: number of cases in configuration
## incl: sufficiency inclusion score
## PRI: proportional reduction in inconsistency
##
## Demandm Laborm Materialm RelCommm OUT n incl PRI
## 5 0 1 0 0 0 11 0.505 0.045
## 15 1 1 1 0 0 45 0.470 0.065
## 16 1 1 1 1 0 65 0.454 0.097
## 14 1 1 0 1 0 56 0.453 0.080
## 1 0 0 0 0 0 39 0.380 0.015
## 9 1 0 0 0 0 59 0.372 0.023
## 13 1 1 0 0 0 115 0.338 0.043
##
## It seems that all output values have been coded to zero.
## Suggestion: lower the inclusion score for the presence of the outcome,
## the relevant argument is "incl.cut" which now has a value of 0.8.
##
##

## $SA16_low
##
## OUT: output value
## n: number of cases in configuration
## incl: sufficiency inclusion score
## PRI: proportional reduction in inconsistency
##
## Demandm Laborm Materialm RelCommm OUT n incl PRI
## 14 1 1 0 1 1 56 0.913 0.811
## 16 1 1 1 1 1 65 0.912 0.823
## 15 1 1 1 0 1 45 0.912 0.776
## 13 1 1 0 0 1 115 0.871 0.748
## 5 0 1 0 0 1 11 0.867 0.579
## 9 1 0 0 0 1 59 0.853 0.655
## 1 0 0 0 0 0 39 0.703 0.314
##
##
## $SA16_high
##
## OUT: output value
## n: number of cases in configuration
## incl: sufficiency inclusion score
## PRI: proportional reduction in inconsistency
##
## Demandm Laborm Materialm RelCommm OUT n incl PRI
## 14 1 1 0 1 0 56 0.786 0.477
## 5 0 1 0 0 0 11 0.755 0.285
## 16 1 1 1 1 0 65 0.754 0.465
## 15 1 1 1 0 0 45 0.752 0.336
## 13 1 1 0 0 0 115 0.676 0.353
## 9 1 0 0 0 0 59 0.654 0.220
## 1 0 0 0 0 0 39 0.546 0.086
##
## It seems that all output values have been coded to zero.
## Suggestion: lower the inclusion score for the presence of the outcome,
## the relevant argument is "incl.cut" which now has a value of 0.8.
##
##

## $SA17_low
##
## OUT: output value
## n: number of cases in configuration
## incl: sufficiency inclusion score
## PRI: proportional reduction in inconsistency
##
## Demandm Laborm Materialm RelCommm OUT n incl PRI
## 15 1 1 1 0 1 45 0.937 0.877
## 14 1 1 0 1 1 56 0.934 0.878
## 16 1 1 1 1 1 65 0.924 0.871
## 13 1 1 0 0 1 115 0.896 0.823
## 9 1 0 0 0 1 59 0.878 0.750
## 5 0 1 0 0 1 11 0.868 0.665
## 1 0 0 0 0 0 39 0.701 0.409
##
##
## $SA17_high
##
## OUT: output value
## n: number of cases in configuration
## incl: sufficiency inclusion score
## PRI: proportional reduction in inconsistency
##
## Demandm Laborm Materialm RelCommm OUT n incl PRI
## 15 1 1 1 0 1 45 0.850 0.668
## 14 1 1 0 1 1 56 0.845 0.677
## 16 1 1 1 1 1 65 0.830 0.671
## 5 0 1 0 0 0 11 0.792 0.468
## 13 1 1 0 0 0 115 0.756 0.566
## 9 1 0 0 0 0 59 0.710 0.405
## 1 0 0 0 0 0 39 0.589 0.217
##
##

## $SA18a_low
##
## OUT: output value
## n: number of cases in configuration
## incl: sufficiency inclusion score
## PRI: proportional reduction in inconsistency
##
## Demandm Laborm Materialm RelCommm OUT n incl PRI
## 16 1 1 1 1 0 65 0.785 0.564
## 14 1 1 0 1 0 56 0.773 0.515
## 15 1 1 1 0 0 45 0.769 0.460
## 5 0 1 0 0 0 11 0.759 0.375
## 13 1 1 0 0 0 115 0.721 0.484
## 9 1 0 0 0 0 59 0.718 0.397
## 1 0 0 0 0 0 39 0.591 0.207
##
## It seems that all output values have been coded to zero.
## Suggestion: lower the inclusion score for the presence of the outcome,
## the relevant argument is "incl.cut" which now has a value of 0.8.
##
##
## $SA18a_high
##
## OUT: output value
## n: number of cases in configuration
## incl: sufficiency inclusion score
## PRI: proportional reduction in inconsistency
##
## Demandm Laborm Materialm RelCommm OUT n incl PRI
## 5 0 1 0 0 0 11 0.657 0.182
## 14 1 1 0 1 0 56 0.610 0.229
## 15 1 1 1 0 0 45 0.587 0.143
## 16 1 1 1 1 0 65 0.582 0.214
## 9 1 0 0 0 0 59 0.539 0.129
## 13 1 1 0 0 0 115 0.507 0.179
## 1 0 0 0 0 0 39 0.498 0.095
##
## It seems that all output values have been coded to zero.
## Suggestion: lower the inclusion score for the presence of the outcome,
## the relevant argument is "incl.cut" which now has a value of 0.8.
##
##

## $SA18b_low
##
## OUT: output value
## n: number of cases in configuration
## incl: sufficiency inclusion score
## PRI: proportional reduction in inconsistency
##
## Demandm Laborm Materialm RelCommm OUT n incl PRI
## 16 1 1 1 1 0 65 0.696 0.407
## 14 1 1 0 1 0 56 0.687 0.370
## 5 0 1 0 0 0 11 0.674 0.203
## 15 1 1 1 0 0 45 0.662 0.262
## 9 1 0 0 0 0 59 0.643 0.265
## 13 1 1 0 0 0 115 0.629 0.336
## 1 0 0 0 0 0 39 0.533 0.131
##
## It seems that all output values have been coded to zero.
## Suggestion: lower the inclusion score for the presence of the outcome,
## the relevant argument is "incl.cut" which now has a value of 0.8.
##
##
## $SA18b_high
##
## OUT: output value
## n: number of cases in configuration
## incl: sufficiency inclusion score
## PRI: proportional reduction in inconsistency
##
## Demandm Laborm Materialm RelCommm OUT n incl PRI
## 5 0 1 0 0 0 11 0.579 0.079
## 14 1 1 0 1 0 56 0.522 0.137
## 15 1 1 1 0 0 45 0.494 0.075
## 16 1 1 1 1 0 65 0.492 0.133
## 9 1 0 0 0 0 59 0.462 0.050
## 1 0 0 0 0 0 39 0.442 0.038
## 13 1 1 0 0 0 115 0.410 0.080
##
## It seems that all output values have been coded to zero.
## Suggestion: lower the inclusion score for the presence of the outcome,
## the relevant argument is "incl.cut" which now has a value of 0.8.
##
##

## $SA19_low
##
## OUT: output value
## n: number of cases in configuration
## incl: sufficiency inclusion score
## PRI: proportional reduction in inconsistency
##
## Demandm Laborm Materialm RelCommm OUT n incl PRI
## 15 1 1 1 0 1 45 0.941 0.863
## 14 1 1 0 1 1 56 0.935 0.861
## 13 1 1 0 0 1 115 0.911 0.832
## 16 1 1 1 1 1 65 0.910 0.827
## 5 0 1 0 0 1 11 0.885 0.656
## 9 1 0 0 0 1 59 0.871 0.698
## 1 0 0 0 0 0 39 0.743 0.414
##
##
## $SA19_high
##
## OUT: output value
## n: number of cases in configuration
## incl: sufficiency inclusion score
## PRI: proportional reduction in inconsistency
##
## Demandm Laborm Materialm RelCommm OUT n incl PRI
## 15 1 1 1 0 1 45 0.800 0.494
## 14 1 1 0 1 0 56 0.798 0.521
## 5 0 1 0 0 0 11 0.796 0.349
## 16 1 1 1 1 0 65 0.775 0.544
## 13 1 1 0 0 0 115 0.721 0.444
## 9 1 0 0 0 0 59 0.700 0.293
## 1 0 0 0 0 0 39 0.584 0.122
##
##

## $SA20_low
##
## OUT: output value
## n: number of cases in configuration
## incl: sufficiency inclusion score
## PRI: proportional reduction in inconsistency
##
## Demandm Laborm Materialm RelCommm OUT n incl PRI
## 16 1 1 1 1 1 65 0.830 0.650
## 15 1 1 1 0 1 45 0.827 0.576
## 14 1 1 0 1 1 56 0.825 0.586
## 5 0 1 0 0 0 11 0.775 0.306
## 13 1 1 0 0 0 115 0.767 0.522
## 9 1 0 0 0 0 59 0.714 0.326
## 1 0 0 0 0 0 39 0.613 0.170
##
##
## $SA20_high
##
## OUT: output value
## n: number of cases in configuration
## incl: sufficiency inclusion score
## PRI: proportional reduction in inconsistency
##
## Demandm Laborm Materialm RelCommm OUT n incl PRI
## 15 1 1 1 0 0 45 0.667 0.181
## 16 1 1 1 1 0 65 0.661 0.291
## 5 0 1 0 0 0 11 0.645 0.088
## 14 1 1 0 1 0 56 0.641 0.192
## 9 1 0 0 0 0 59 0.514 0.048
## 13 1 1 0 0 0 115 0.512 0.116
## 1 0 0 0 0 0 39 0.472 0.022
##
## It seems that all output values have been coded to zero.
## Suggestion: lower the inclusion score for the presence of the outcome,
## the relevant argument is "incl.cut" which now has a value of 0.8.
##
##

## $SA21_low
##
## OUT: output value
## n: number of cases in configuration
## incl: sufficiency inclusion score
## PRI: proportional reduction in inconsistency
##
## Demandm Laborm Materialm RelCommm OUT n incl PRI
## 15 1 1 1 0 1 45 0.944 0.878
## 14 1 1 0 1 1 56 0.933 0.867
## 16 1 1 1 1 1 65 0.929 0.872
## 13 1 1 0 0 1 115 0.908 0.835
## 5 0 1 0 0 1 11 0.891 0.685
## 9 1 0 0 0 1 59 0.884 0.744
## 1 0 0 0 0 0 39 0.729 0.412
##
##
## $SA21_high
##
## OUT: output value
## n: number of cases in configuration
## incl: sufficiency inclusion score
## PRI: proportional reduction in inconsistency
##
## Demandm Laborm Materialm RelCommm OUT n incl PRI
## 14 1 1 0 1 1 56 0.839 0.618
## 15 1 1 1 0 1 45 0.825 0.586
## 16 1 1 1 1 1 65 0.823 0.641
## 5 0 1 0 0 1 11 0.803 0.387
## 13 1 1 0 0 0 115 0.753 0.518
## 9 1 0 0 0 0 59 0.722 0.368
## 1 0 0 0 0 0 39 0.604 0.163
##
##

## $SA22_low
##
## OUT: output value
## n: number of cases in configuration
## incl: sufficiency inclusion score
## PRI: proportional reduction in inconsistency
##
## Demandm Laborm Materialm RelCommm OUT n incl PRI
## 14 1 1 0 1 1 56 0.958 0.928
## 15 1 1 1 0 1 45 0.948 0.910
## 13 1 1 0 0 1 115 0.944 0.911
## 16 1 1 1 1 1 65 0.938 0.901
## 9 1 0 0 0 1 59 0.909 0.831
## 5 0 1 0 0 1 11 0.908 0.779
## 1 0 0 0 0 0 39 0.742 0.494
##
##
## $SA22_high
##
## OUT: output value
## n: number of cases in configuration
## incl: sufficiency inclusion score
## PRI: proportional reduction in inconsistency
##
## Demandm Laborm Materialm RelCommm OUT n incl PRI
## 15 1 1 1 0 1 45 0.908 0.824
## 16 1 1 1 1 1 65 0.870 0.781
## 14 1 1 0 1 1 56 0.866 0.757
## 5 0 1 0 0 1 11 0.824 0.577
## 13 1 1 0 0 1 115 0.811 0.689
## 9 1 0 0 0 0 59 0.770 0.564
## 1 0 0 0 0 0 39 0.620 0.277
##
##

## $SA23_low
##
## OUT: output value
## n: number of cases in configuration
## incl: sufficiency inclusion score
## PRI: proportional reduction in inconsistency
##
## Demandm Laborm Materialm RelCommm OUT n incl PRI
## 16 1 1 1 1 1 65 0.845 0.674
## 15 1 1 1 0 1 45 0.839 0.575
## 14 1 1 0 1 1 56 0.836 0.601
## 13 1 1 0 0 0 115 0.796 0.557
## 5 0 1 0 0 0 11 0.778 0.268
## 9 1 0 0 0 0 59 0.775 0.406
## 1 0 0 0 0 0 39 0.599 0.123
##
##
## $SA23_high
##
## OUT: output value
## n: number of cases in configuration
## incl: sufficiency inclusion score
## PRI: proportional reduction in inconsistency
##
## Demandm Laborm Materialm RelCommm OUT n incl PRI
## 16 1 1 1 1 0 65 0.658 0.264
## 14 1 1 0 1 0 56 0.647 0.195
## 15 1 1 1 0 0 45 0.642 0.137
## 5 0 1 0 0 0 11 0.639 0.064
## 9 1 0 0 0 0 59 0.529 0.053
## 13 1 1 0 0 0 115 0.519 0.118
## 1 0 0 0 0 0 39 0.475 0.012
##
## It seems that all output values have been coded to zero.
## Suggestion: lower the inclusion score for the presence of the outcome,
## the relevant argument is "incl.cut" which now has a value of 0.8.
##
##

## $SA24_low
##
## OUT: output value
## n: number of cases in configuration
## incl: sufficiency inclusion score
## PRI: proportional reduction in inconsistency
##
## Demandm Laborm Materialm RelCommm OUT n incl PRI
## 15 1 1 1 0 0 45 0.773 0.442
## 16 1 1 1 1 0 65 0.773 0.534
## 5 0 1 0 0 0 11 0.761 0.245
## 14 1 1 0 1 0 56 0.755 0.437
## 9 1 0 0 0 0 59 0.706 0.331
## 13 1 1 0 0 0 115 0.700 0.399
## 1 0 0 0 0 0 39 0.596 0.125
##
## It seems that all output values have been coded to zero.
## Suggestion: lower the inclusion score for the presence of the outcome,
## the relevant argument is "incl.cut" which now has a value of 0.8.
##
##
## $SA24_high
##
## OUT: output value
## n: number of cases in configuration
## incl: sufficiency inclusion score
## PRI: proportional reduction in inconsistency
##
## Demandm Laborm Materialm RelCommm OUT n incl PRI
## 5 0 1 0 0 0 11 0.627 0.044
## 16 1 1 1 1 0 65 0.592 0.184
## 15 1 1 1 0 0 45 0.590 0.080
## 14 1 1 0 1 0 56 0.557 0.100
## 9 1 0 0 0 0 59 0.504 0.063
## 1 0 0 0 0 0 39 0.465 0.014
## 13 1 1 0 0 0 115 0.456 0.073
##
## It seems that all output values have been coded to zero.
## Suggestion: lower the inclusion score for the presence of the outcome,
## the relevant argument is "incl.cut" which now has a value of 0.8.
##
##

## $SA25_low
##
## OUT: output value
## n: number of cases in configuration
## incl: sufficiency inclusion score
## PRI: proportional reduction in inconsistency
##
## Demandm Laborm Materialm RelCommm OUT n incl PRI
## 14 1 1 0 1 1 56 0.958 0.920
## 15 1 1 1 0 1 45 0.948 0.889
## 13 1 1 0 0 1 115 0.942 0.901
## 16 1 1 1 1 1 65 0.940 0.893
## 9 1 0 0 0 1 59 0.923 0.834
## 5 0 1 0 0 1 11 0.911 0.749
## 1 0 0 0 0 0 39 0.754 0.465
##
##
## $SA25_high
##
## OUT: output value
## n: number of cases in configuration
## incl: sufficiency inclusion score
## PRI: proportional reduction in inconsistency
##
## Demandm Laborm Materialm RelCommm OUT n incl PRI
## 14 1 1 0 1 1 56 0.885 0.736
## 15 1 1 1 0 1 45 0.853 0.627
## 16 1 1 1 1 1 65 0.852 0.700
## 5 0 1 0 0 1 11 0.834 0.477
## 13 1 1 0 0 1 115 0.805 0.608
## 9 1 0 0 0 0 59 0.775 0.448
## 1 0 0 0 0 0 39 0.627 0.190

# Result: Truth Table: Reasons for MNC triggering single Outcomes of MNC (for n.cut = 15)

## $SA1_low
##
## OUT: output value
## n: number of cases in configuration
## incl: sufficiency inclusion score
## PRI: proportional reduction in inconsistency
##
## Demandm Laborm Materialm RelCommm OUT n incl PRI
## 14 1 1 0 1 1 56 0.970 0.939
## 15 1 1 1 0 1 45 0.945 0.882
## 13 1 1 0 0 1 115 0.940 0.892
## 16 1 1 1 1 1 65 0.930 0.873
## 9 1 0 0 0 1 59 0.889 0.759
## 1 0 0 0 0 0 39 0.729 0.419
##
##
## $SA1_high
##
## OUT: output value
## n: number of cases in configuration
## incl: sufficiency inclusion score
## PRI: proportional reduction in inconsistency
##
## Demandm Laborm Materialm RelCommm OUT n incl PRI
## 14 1 1 0 1 1 56 0.878 0.704
## 15 1 1 1 0 1 45 0.849 0.612
## 16 1 1 1 1 1 65 0.820 0.602
## 13 1 1 0 0 0 115 0.786 0.570
## 9 1 0 0 0 0 59 0.747 0.397
## 1 0 0 0 0 0 39 0.611 0.155
##
##

## $SA2_low
##
## OUT: output value
## n: number of cases in configuration
## incl: sufficiency inclusion score
## PRI: proportional reduction in inconsistency
##
## Demandm Laborm Materialm RelCommm OUT n incl PRI
## 14 1 1 0 1 1 56 0.813 0.590
## 15 1 1 1 0 1 45 0.803 0.529
## 16 1 1 1 1 0 65 0.785 0.554
## 13 1 1 0 0 0 115 0.754 0.519
## 9 1 0 0 0 0 59 0.739 0.394
## 1 0 0 0 0 0 39 0.621 0.189
##
##
## $SA2_high
##
## OUT: output value
## n: number of cases in configuration
## incl: sufficiency inclusion score
## PRI: proportional reduction in inconsistency
##
## Demandm Laborm Materialm RelCommm OUT n incl PRI
## 14 1 1 0 1 0 56 0.646 0.252
## 15 1 1 1 0 0 45 0.622 0.175
## 16 1 1 1 1 0 65 0.593 0.212
## 9 1 0 0 0 0 59 0.535 0.087
## 13 1 1 0 0 0 115 0.515 0.148
## 1 0 0 0 0 0 39 0.492 0.031
##
## It seems that all output values have been coded to zero.
## Suggestion: lower the inclusion score for the presence of the outcome,
## the relevant argument is "incl.cut" which now has a value of 0.8.
##
##

## $SA3_low
##
## OUT: output value
## n: number of cases in configuration
## incl: sufficiency inclusion score
## PRI: proportional reduction in inconsistency
##
## Demandm Laborm Materialm RelCommm OUT n incl PRI
## 15 1 1 1 0 1 45 0.845 0.632
## 16 1 1 1 1 1 65 0.817 0.640
## 14 1 1 0 1 0 56 0.778 0.555
## 13 1 1 0 0 0 115 0.734 0.525
## 9 1 0 0 0 0 59 0.702 0.381
## 1 0 0 0 0 0 39 0.553 0.149
##
##
## $SA3_high
##
## OUT: output value
## n: number of cases in configuration
## incl: sufficiency inclusion score
## PRI: proportional reduction in inconsistency
##
## Demandm Laborm Materialm RelCommm OUT n incl PRI
## 15 1 1 1 0 0 45 0.684 0.261
## 14 1 1 0 1 0 56 0.655 0.296
## 16 1 1 1 1 0 65 0.654 0.324
## 13 1 1 0 0 0 115 0.547 0.207
## 9 1 0 0 0 0 59 0.538 0.115
## 1 0 0 0 0 0 39 0.466 0.040
##
## It seems that all output values have been coded to zero.
## Suggestion: lower the inclusion score for the presence of the outcome,
## the relevant argument is "incl.cut" which now has a value of 0.8.
##
##

## $SA4_low
##
## OUT: output value
## n: number of cases in configuration
## incl: sufficiency inclusion score
## PRI: proportional reduction in inconsistency
##
## Demandm Laborm Materialm RelCommm OUT n incl PRI
## 15 1 1 1 0 0 45 0.796 0.507
## 14 1 1 0 1 0 56 0.790 0.516
## 16 1 1 1 1 0 65 0.768 0.528
## 13 1 1 0 0 0 115 0.700 0.419
## 9 1 0 0 0 0 59 0.659 0.267
## 1 0 0 0 0 0 39 0.543 0.122
##
## It seems that all output values have been coded to zero.
## Suggestion: lower the inclusion score for the presence of the outcome,
## the relevant argument is "incl.cut" which now has a value of 0.8.
##
##
## $SA4_high
##
## OUT: output value
## n: number of cases in configuration
## incl: sufficiency inclusion score
## PRI: proportional reduction in inconsistency
##
## Demandm Laborm Materialm RelCommm OUT n incl PRI
## 15 1 1 1 0 0 45 0.609 0.157
## 16 1 1 1 1 0 65 0.590 0.222
## 14 1 1 0 1 0 56 0.580 0.146
## 9 1 0 0 0 0 59 0.478 0.059
## 13 1 1 0 0 0 115 0.460 0.095
## 1 0 0 0 0 0 39 0.438 0.032
##
## It seems that all output values have been coded to zero.
## Suggestion: lower the inclusion score for the presence of the outcome,
## the relevant argument is "incl.cut" which now has a value of 0.8.
##
##

## $SA5_low
##
## OUT: output value
## n: number of cases in configuration
## incl: sufficiency inclusion score
## PRI: proportional reduction in inconsistency
##
## Demandm Laborm Materialm RelCommm OUT n incl PRI
## 15 1 1 1 0 1 45 0.899 0.747
## 14 1 1 0 1 1 56 0.875 0.702
## 16 1 1 1 1 1 65 0.868 0.730
## 13 1 1 0 0 1 115 0.836 0.663
## 9 1 0 0 0 1 59 0.803 0.505
## 1 0 0 0 0 0 39 0.641 0.221
##
##
## $SA5_high
##
## OUT: output value
## n: number of cases in configuration
## incl: sufficiency inclusion score
## PRI: proportional reduction in inconsistency
##
## Demandm Laborm Materialm RelCommm OUT n incl PRI
## 15 1 1 1 0 0 45 0.739 0.319
## 16 1 1 1 1 0 65 0.696 0.354
## 14 1 1 0 1 0 56 0.691 0.279
## 13 1 1 0 0 0 115 0.600 0.233
## 9 1 0 0 0 0 59 0.593 0.127
## 1 0 0 0 0 0 39 0.516 0.046
##
## It seems that all output values have been coded to zero.
## Suggestion: lower the inclusion score for the presence of the outcome,
## the relevant argument is "incl.cut" which now has a value of 0.8.
##
##

## $SA6_low
##
## OUT: output value
## n: number of cases in configuration
## incl: sufficiency inclusion score
## PRI: proportional reduction in inconsistency
##
## Demandm Laborm Materialm RelCommm OUT n incl PRI
## 14 1 1 0 1 1 56 0.852 0.659
## 16 1 1 1 1 1 65 0.850 0.709
## 15 1 1 1 0 1 45 0.848 0.653
## 13 1 1 0 0 1 115 0.805 0.618
## 9 1 0 0 0 0 59 0.761 0.447
## 1 0 0 0 0 0 39 0.622 0.200
##
##
## $SA6_high
##
## OUT: output value
## n: number of cases in configuration
## incl: sufficiency inclusion score
## PRI: proportional reduction in inconsistency
##
## Demandm Laborm Materialm RelCommm OUT n incl PRI
## 15 1 1 1 0 0 45 0.712 0.333
## 16 1 1 1 1 0 65 0.694 0.386
## 14 1 1 0 1 0 56 0.664 0.265
## 13 1 1 0 0 0 115 0.570 0.226
## 9 1 0 0 0 0 59 0.562 0.120
## 1 0 0 0 0 0 39 0.496 0.046
##
## It seems that all output values have been coded to zero.
## Suggestion: lower the inclusion score for the presence of the outcome,
## the relevant argument is "incl.cut" which now has a value of 0.8.
##
##

## $SA7_low
##
## OUT: output value
## n: number of cases in configuration
## incl: sufficiency inclusion score
## PRI: proportional reduction in inconsistency
##
## Demandm Laborm Materialm RelCommm OUT n incl PRI
## 14 1 1 0 1 1 56 0.875 0.726
## 15 1 1 1 0 1 45 0.864 0.664
## 16 1 1 1 1 1 65 0.861 0.721
## 13 1 1 0 0 0 115 0.781 0.588
## 9 1 0 0 0 0 59 0.736 0.431
## 1 0 0 0 0 0 39 0.627 0.210
##
##
## $SA7_high
##
## OUT: output value
## n: number of cases in configuration
## incl: sufficiency inclusion score
## PRI: proportional reduction in inconsistency
##
## Demandm Laborm Materialm RelCommm OUT n incl PRI
## 14 1 1 0 1 0 56 0.715 0.349
## 15 1 1 1 0 0 45 0.691 0.280
## 16 1 1 1 1 0 65 0.689 0.379
## 13 1 1 0 0 0 115 0.575 0.227
## 9 1 0 0 0 0 59 0.571 0.153
## 1 0 0 0 0 0 39 0.487 0.047
##
## It seems that all output values have been coded to zero.
## Suggestion: lower the inclusion score for the presence of the outcome,
## the relevant argument is "incl.cut" which now has a value of 0.8.
##
##

## $SA8_low
##
## OUT: output value
## n: number of cases in configuration
## incl: sufficiency inclusion score
## PRI: proportional reduction in inconsistency
##
## Demandm Laborm Materialm RelCommm OUT n incl PRI
## 14 1 1 0 1 1 56 0.928 0.856
## 15 1 1 1 0 1 45 0.922 0.836
## 16 1 1 1 1 1 65 0.906 0.833
## 13 1 1 0 0 1 115 0.893 0.810
## 9 1 0 0 0 1 59 0.874 0.723
## 1 0 0 0 0 0 39 0.756 0.449
##
##
## $SA8_high
##
## OUT: output value
## n: number of cases in configuration
## incl: sufficiency inclusion score
## PRI: proportional reduction in inconsistency
##
## Demandm Laborm Materialm RelCommm OUT n incl PRI
## 15 1 1 1 0 1 45 0.832 0.587
## 14 1 1 0 1 1 56 0.829 0.599
## 16 1 1 1 1 1 65 0.816 0.610
## 13 1 1 0 0 0 115 0.737 0.484
## 9 1 0 0 0 0 59 0.702 0.318
## 1 0 0 0 0 0 39 0.600 0.172
##
##

## $SA9a_low
##
## OUT: output value
## n: number of cases in configuration
## incl: sufficiency inclusion score
## PRI: proportional reduction in inconsistency
##
## Demandm Laborm Materialm RelCommm OUT n incl PRI
## 14 1 1 0 1 1 56 0.977 0.961
## 15 1 1 1 0 1 45 0.977 0.956
## 16 1 1 1 1 1 65 0.958 0.933
## 13 1 1 0 0 1 115 0.957 0.932
## 9 1 0 0 0 1 59 0.940 0.887
## 1 0 0 0 0 1 39 0.803 0.595
##
##
## $SA9a_high
##
## OUT: output value
## n: number of cases in configuration
## incl: sufficiency inclusion score
## PRI: proportional reduction in inconsistency
##
## Demandm Laborm Materialm RelCommm OUT n incl PRI
## 14 1 1 0 1 1 56 0.926 0.851
## 15 1 1 1 0 1 45 0.915 0.811
## 16 1 1 1 1 1 65 0.908 0.829
## 13 1 1 0 0 1 115 0.863 0.749
## 9 1 0 0 0 1 59 0.839 0.630
## 1 0 0 0 0 0 39 0.692 0.309
##
##

## $SA9b_low
##
## OUT: output value
## n: number of cases in configuration
## incl: sufficiency inclusion score
## PRI: proportional reduction in inconsistency
##
## Demandm Laborm Materialm RelCommm OUT n incl PRI
## 14 1 1 0 1 1 56 0.973 0.954
## 15 1 1 1 0 1 45 0.967 0.938
## 13 1 1 0 0 1 115 0.957 0.933
## 16 1 1 1 1 1 65 0.952 0.922
## 9 1 0 0 0 1 59 0.952 0.908
## 1 0 0 0 0 1 39 0.802 0.594
##
##
## $SA9b_high
##
## OUT: output value
## n: number of cases in configuration
## incl: sufficiency inclusion score
## PRI: proportional reduction in inconsistency
##
## Demandm Laborm Materialm RelCommm OUT n incl PRI
## 14 1 1 0 1 1 56 0.925 0.844
## 15 1 1 1 0 1 45 0.915 0.811
## 16 1 1 1 1 1 65 0.895 0.801
## 13 1 1 0 0 1 115 0.879 0.775
## 9 1 0 0 0 1 59 0.846 0.645
## 1 0 0 0 0 0 39 0.693 0.324
##
##

## $SA10a_low
##
## OUT: output value
## n: number of cases in configuration
## incl: sufficiency inclusion score
## PRI: proportional reduction in inconsistency
##
## Demandm Laborm Materialm RelCommm OUT n incl PRI
## 15 1 1 1 0 1 45 0.981 0.964
## 14 1 1 0 1 1 56 0.974 0.955
## 16 1 1 1 1 1 65 0.960 0.934
## 13 1 1 0 0 1 115 0.954 0.928
## 9 1 0 0 0 1 59 0.942 0.892
## 1 0 0 0 0 1 39 0.820 0.631
##
##
## $SA10a_high
##
## OUT: output value
## n: number of cases in configuration
## incl: sufficiency inclusion score
## PRI: proportional reduction in inconsistency
##
## Demandm Laborm Materialm RelCommm OUT n incl PRI
## 14 1 1 0 1 1 56 0.921 0.838
## 15 1 1 1 0 1 45 0.918 0.822
## 16 1 1 1 1 1 65 0.887 0.790
## 13 1 1 0 0 1 115 0.867 0.758
## 9 1 0 0 0 1 59 0.840 0.639
## 1 0 0 0 0 0 39 0.701 0.354
##
##

## $SA10b_low
##
## OUT: output value
## n: number of cases in configuration
## incl: sufficiency inclusion score
## PRI: proportional reduction in inconsistency
##
## Demandm Laborm Materialm RelCommm OUT n incl PRI
## 14 1 1 0 1 1 56 0.986 0.979
## 15 1 1 1 0 1 45 0.984 0.972
## 16 1 1 1 1 1 65 0.981 0.971
## 13 1 1 0 0 1 115 0.971 0.958
## 9 1 0 0 0 1 59 0.959 0.929
## 1 0 0 0 0 1 39 0.830 0.675
##
##
## $SA10b_high
##
## OUT: output value
## n: number of cases in configuration
## incl: sufficiency inclusion score
## PRI: proportional reduction in inconsistency
##
## Demandm Laborm Materialm RelCommm OUT n incl PRI
## 14 1 1 0 1 1 56 0.958 0.923
## 15 1 1 1 0 1 45 0.948 0.897
## 16 1 1 1 1 1 65 0.947 0.908
## 13 1 1 0 0 1 115 0.920 0.865
## 9 1 0 0 0 1 59 0.876 0.743
## 1 0 0 0 0 0 39 0.731 0.428
##
##

## $SA11_low
##
## OUT: output value
## n: number of cases in configuration
## incl: sufficiency inclusion score
## PRI: proportional reduction in inconsistency
##
## Demandm Laborm Materialm RelCommm OUT n incl PRI
## 15 1 1 1 0 1 45 0.820 0.508
## 14 1 1 0 1 1 56 0.806 0.524
## 16 1 1 1 1 0 65 0.788 0.541
## 13 1 1 0 0 0 115 0.765 0.483
## 9 1 0 0 0 0 59 0.754 0.358
## 1 0 0 0 0 0 39 0.604 0.132
##
##
## $SA11_high
##
## OUT: output value
## n: number of cases in configuration
## incl: sufficiency inclusion score
## PRI: proportional reduction in inconsistency
##
## Demandm Laborm Materialm RelCommm OUT n incl PRI
## 15 1 1 1 0 0 45 0.608 0.118
## 14 1 1 0 1 0 56 0.599 0.153
## 16 1 1 1 1 0 65 0.578 0.168
## 9 1 0 0 0 0 59 0.496 0.041
## 13 1 1 0 0 0 115 0.462 0.069
## 1 0 0 0 0 0 39 0.457 0.033
##
## It seems that all output values have been coded to zero.
## Suggestion: lower the inclusion score for the presence of the outcome,
## the relevant argument is "incl.cut" which now has a value of 0.8.
##
##

## $SA12_low
##
## OUT: output value
## n: number of cases in configuration
## incl: sufficiency inclusion score
## PRI: proportional reduction in inconsistency
##
## Demandm Laborm Materialm RelCommm OUT n incl PRI
## 14 1 1 0 1 1 56 0.874 0.731
## 15 1 1 1 0 1 45 0.866 0.697
## 13 1 1 0 0 1 115 0.832 0.667
## 16 1 1 1 1 1 65 0.830 0.682
## 9 1 0 0 0 1 59 0.817 0.554
## 1 0 0 0 0 0 39 0.642 0.240
##
##
## $SA12_high
##
## OUT: output value
## n: number of cases in configuration
## incl: sufficiency inclusion score
## PRI: proportional reduction in inconsistency
##
## Demandm Laborm Materialm RelCommm OUT n incl PRI
## 15 1 1 1 0 0 45 0.728 0.386
## 14 1 1 0 1 0 56 0.724 0.395
## 16 1 1 1 1 0 65 0.695 0.405
## 13 1 1 0 0 0 115 0.596 0.266
## 9 1 0 0 0 0 59 0.594 0.174
## 1 0 0 0 0 0 39 0.514 0.104
##
## It seems that all output values have been coded to zero.
## Suggestion: lower the inclusion score for the presence of the outcome,
## the relevant argument is "incl.cut" which now has a value of 0.8.
##
##

## $SA13_low
##
## OUT: output value
## n: number of cases in configuration
## incl: sufficiency inclusion score
## PRI: proportional reduction in inconsistency
##
## Demandm Laborm Materialm RelCommm OUT n incl PRI
## 16 1 1 1 1 0 65 0.675 0.402
## 14 1 1 0 1 0 56 0.653 0.366
## 15 1 1 1 0 0 45 0.645 0.280
## 13 1 1 0 0 0 115 0.535 0.244
## 9 1 0 0 0 0 59 0.534 0.167
## 1 0 0 0 0 0 39 0.490 0.112
##
## It seems that all output values have been coded to zero.
## Suggestion: lower the inclusion score for the presence of the outcome,
## the relevant argument is "incl.cut" which now has a value of 0.8.
##
##
## $SA13_high
##
## OUT: output value
## n: number of cases in configuration
## incl: sufficiency inclusion score
## PRI: proportional reduction in inconsistency
##
## Demandm Laborm Materialm RelCommm OUT n incl PRI
## 14 1 1 0 1 0 56 0.522 0.181
## 16 1 1 1 1 0 65 0.504 0.171
## 15 1 1 1 0 0 45 0.490 0.079
## 1 0 0 0 0 0 39 0.399 0.032
## 9 1 0 0 0 0 59 0.398 0.049
## 13 1 1 0 0 0 115 0.372 0.078
##
## It seems that all output values have been coded to zero.
## Suggestion: lower the inclusion score for the presence of the outcome,
## the relevant argument is "incl.cut" which now has a value of 0.8.
##
##

## $SA14a_low
##
## OUT: output value
## n: number of cases in configuration
## incl: sufficiency inclusion score
## PRI: proportional reduction in inconsistency
##
## Demandm Laborm Materialm RelCommm OUT n incl PRI
## 15 1 1 1 0 1 45 0.887 0.708
## 16 1 1 1 1 1 65 0.883 0.753
## 14 1 1 0 1 1 56 0.872 0.712
## 13 1 1 0 0 1 115 0.827 0.650
## 9 1 0 0 0 0 59 0.795 0.488
## 1 0 0 0 0 0 39 0.670 0.272
##
##
## $SA14a_high
##
## OUT: output value
## n: number of cases in configuration
## incl: sufficiency inclusion score
## PRI: proportional reduction in inconsistency
##
## Demandm Laborm Materialm RelCommm OUT n incl PRI
## 14 1 1 0 1 0 56 0.726 0.355
## 16 1 1 1 1 0 65 0.706 0.376
## 15 1 1 1 0 0 45 0.703 0.277
## 13 1 1 0 0 0 115 0.590 0.248
## 9 1 0 0 0 0 59 0.567 0.124
## 1 0 0 0 0 0 39 0.524 0.090
##
## It seems that all output values have been coded to zero.
## Suggestion: lower the inclusion score for the presence of the outcome,
## the relevant argument is "incl.cut" which now has a value of 0.8.
##
##

## $SA14b_low
##
## OUT: output value
## n: number of cases in configuration
## incl: sufficiency inclusion score
## PRI: proportional reduction in inconsistency
##
## Demandm Laborm Materialm RelCommm OUT n incl PRI
## 14 1 1 0 1 1 56 0.963 0.927
## 15 1 1 1 0 1 45 0.953 0.898
## 16 1 1 1 1 1 65 0.952 0.913
## 13 1 1 0 0 1 115 0.926 0.863
## 9 1 0 0 0 1 59 0.895 0.758
## 1 0 0 0 0 0 39 0.717 0.380
##
##
## $SA14b_high
##
## OUT: output value
## n: number of cases in configuration
## incl: sufficiency inclusion score
## PRI: proportional reduction in inconsistency
##
## Demandm Laborm Materialm RelCommm OUT n incl PRI
## 14 1 1 0 1 1 56 0.863 0.699
## 15 1 1 1 0 1 45 0.843 0.614
## 16 1 1 1 1 1 65 0.840 0.677
## 13 1 1 0 0 0 115 0.736 0.509
## 9 1 0 0 0 0 59 0.702 0.346
## 1 0 0 0 0 0 39 0.593 0.138
##
##

## $SA14c_low
##
## OUT: output value
## n: number of cases in configuration
## incl: sufficiency inclusion score
## PRI: proportional reduction in inconsistency
##
## Demandm Laborm Materialm RelCommm OUT n incl PRI
## 14 1 1 0 1 1 56 0.968 0.945
## 15 1 1 1 0 1 45 0.959 0.920
## 16 1 1 1 1 1 65 0.959 0.931
## 13 1 1 0 0 1 115 0.940 0.901
## 9 1 0 0 0 1 59 0.903 0.808
## 1 0 0 0 0 0 39 0.735 0.461
##
##
## $SA14c_high
##
## OUT: output value
## n: number of cases in configuration
## incl: sufficiency inclusion score
## PRI: proportional reduction in inconsistency
##
## Demandm Laborm Materialm RelCommm OUT n incl PRI
## 14 1 1 0 1 1 56 0.907 0.817
## 16 1 1 1 1 1 65 0.882 0.781
## 15 1 1 1 0 1 45 0.859 0.686
## 13 1 1 0 0 1 115 0.815 0.674
## 9 1 0 0 0 0 59 0.760 0.501
## 1 0 0 0 0 0 39 0.614 0.214
##
##

## $SA15_low
##
## OUT: output value
## n: number of cases in configuration
## incl: sufficiency inclusion score
## PRI: proportional reduction in inconsistency
##
## Demandm Laborm Materialm RelCommm OUT n incl PRI
## 15 1 1 1 0 0 45 0.630 0.241
## 16 1 1 1 1 0 65 0.618 0.307
## 14 1 1 0 1 0 56 0.610 0.263
## 13 1 1 0 0 0 115 0.539 0.223
## 9 1 0 0 0 0 59 0.518 0.136
## 1 0 0 0 0 0 39 0.451 0.064
##
## It seems that all output values have been coded to zero.
## Suggestion: lower the inclusion score for the presence of the outcome,
## the relevant argument is "incl.cut" which now has a value of 0.8.
##
##
## $SA15_high
##
## OUT: output value
## n: number of cases in configuration
## incl: sufficiency inclusion score
## PRI: proportional reduction in inconsistency
##
## Demandm Laborm Materialm RelCommm OUT n incl PRI
## 15 1 1 1 0 0 45 0.470 0.065
## 16 1 1 1 1 0 65 0.454 0.097
## 14 1 1 0 1 0 56 0.453 0.080
## 1 0 0 0 0 0 39 0.380 0.015
## 9 1 0 0 0 0 59 0.372 0.023
## 13 1 1 0 0 0 115 0.338 0.043
##
## It seems that all output values have been coded to zero.
## Suggestion: lower the inclusion score for the presence of the outcome,
## the relevant argument is "incl.cut" which now has a value of 0.8.
##
##

## $SA16_low
##
## OUT: output value
## n: number of cases in configuration
## incl: sufficiency inclusion score
## PRI: proportional reduction in inconsistency
##
## Demandm Laborm Materialm RelCommm OUT n incl PRI
## 14 1 1 0 1 1 56 0.913 0.811
## 16 1 1 1 1 1 65 0.912 0.823
## 15 1 1 1 0 1 45 0.912 0.776
## 13 1 1 0 0 1 115 0.871 0.748
## 9 1 0 0 0 1 59 0.853 0.655
## 1 0 0 0 0 0 39 0.703 0.314
##
##
## $SA16_high
##
## OUT: output value
## n: number of cases in configuration
## incl: sufficiency inclusion score
## PRI: proportional reduction in inconsistency
##
## Demandm Laborm Materialm RelCommm OUT n incl PRI
## 14 1 1 0 1 0 56 0.786 0.477
## 16 1 1 1 1 0 65 0.754 0.465
## 15 1 1 1 0 0 45 0.752 0.336
## 13 1 1 0 0 0 115 0.676 0.353
## 9 1 0 0 0 0 59 0.654 0.220
## 1 0 0 0 0 0 39 0.546 0.086
##
## It seems that all output values have been coded to zero.
## Suggestion: lower the inclusion score for the presence of the outcome,
## the relevant argument is "incl.cut" which now has a value of 0.8.
##
##

## $SA17_low
##
## OUT: output value
## n: number of cases in configuration
## incl: sufficiency inclusion score
## PRI: proportional reduction in inconsistency
##
## Demandm Laborm Materialm RelCommm OUT n incl PRI
## 15 1 1 1 0 1 45 0.937 0.877
## 14 1 1 0 1 1 56 0.934 0.878
## 16 1 1 1 1 1 65 0.924 0.871
## 13 1 1 0 0 1 115 0.896 0.823
## 9 1 0 0 0 1 59 0.878 0.750
## 1 0 0 0 0 0 39 0.701 0.409
##
##
## $SA17_high
##
## OUT: output value
## n: number of cases in configuration
## incl: sufficiency inclusion score
## PRI: proportional reduction in inconsistency
##
## Demandm Laborm Materialm RelCommm OUT n incl PRI
## 15 1 1 1 0 1 45 0.850 0.668
## 14 1 1 0 1 1 56 0.845 0.677
## 16 1 1 1 1 1 65 0.830 0.671
## 13 1 1 0 0 0 115 0.756 0.566
## 9 1 0 0 0 0 59 0.710 0.405
## 1 0 0 0 0 0 39 0.589 0.217
##
##

## $SA18a_low
##
## OUT: output value
## n: number of cases in configuration
## incl: sufficiency inclusion score
## PRI: proportional reduction in inconsistency
##
## Demandm Laborm Materialm RelCommm OUT n incl PRI
## 16 1 1 1 1 0 65 0.785 0.564
## 14 1 1 0 1 0 56 0.773 0.515
## 15 1 1 1 0 0 45 0.769 0.460
## 13 1 1 0 0 0 115 0.721 0.484
## 9 1 0 0 0 0 59 0.718 0.397
## 1 0 0 0 0 0 39 0.591 0.207
##
## It seems that all output values have been coded to zero.
## Suggestion: lower the inclusion score for the presence of the outcome,
## the relevant argument is "incl.cut" which now has a value of 0.8.
##
##
## $SA18a_high
##
## OUT: output value
## n: number of cases in configuration
## incl: sufficiency inclusion score
## PRI: proportional reduction in inconsistency
##
## Demandm Laborm Materialm RelCommm OUT n incl PRI
## 14 1 1 0 1 0 56 0.610 0.229
## 15 1 1 1 0 0 45 0.587 0.143
## 16 1 1 1 1 0 65 0.582 0.214
## 9 1 0 0 0 0 59 0.539 0.129
## 13 1 1 0 0 0 115 0.507 0.179
## 1 0 0 0 0 0 39 0.498 0.095
##
## It seems that all output values have been coded to zero.
## Suggestion: lower the inclusion score for the presence of the outcome,
## the relevant argument is "incl.cut" which now has a value of 0.8.
##
##

## $SA18b_low
##
## OUT: output value
## n: number of cases in configuration
## incl: sufficiency inclusion score
## PRI: proportional reduction in inconsistency
##
## Demandm Laborm Materialm RelCommm OUT n incl PRI
## 16 1 1 1 1 0 65 0.696 0.407
## 14 1 1 0 1 0 56 0.687 0.370
## 15 1 1 1 0 0 45 0.662 0.262
## 9 1 0 0 0 0 59 0.643 0.265
## 13 1 1 0 0 0 115 0.629 0.336
## 1 0 0 0 0 0 39 0.533 0.131
##
## It seems that all output values have been coded to zero.
## Suggestion: lower the inclusion score for the presence of the outcome,
## the relevant argument is "incl.cut" which now has a value of 0.8.
##
##
## $SA18b_high
##
## OUT: output value
## n: number of cases in configuration
## incl: sufficiency inclusion score
## PRI: proportional reduction in inconsistency
##
## Demandm Laborm Materialm RelCommm OUT n incl PRI
## 14 1 1 0 1 0 56 0.522 0.137
## 15 1 1 1 0 0 45 0.494 0.075
## 16 1 1 1 1 0 65 0.492 0.133
## 9 1 0 0 0 0 59 0.462 0.050
## 1 0 0 0 0 0 39 0.442 0.038
## 13 1 1 0 0 0 115 0.410 0.080
##
## It seems that all output values have been coded to zero.
## Suggestion: lower the inclusion score for the presence of the outcome,
## the relevant argument is "incl.cut" which now has a value of 0.8.
##
##

## $SA19_low
##
## OUT: output value
## n: number of cases in configuration
## incl: sufficiency inclusion score
## PRI: proportional reduction in inconsistency
##
## Demandm Laborm Materialm RelCommm OUT n incl PRI
## 15 1 1 1 0 1 45 0.941 0.863
## 14 1 1 0 1 1 56 0.935 0.861
## 13 1 1 0 0 1 115 0.911 0.832
## 16 1 1 1 1 1 65 0.910 0.827
## 9 1 0 0 0 1 59 0.871 0.698
## 1 0 0 0 0 0 39 0.743 0.414
##
##
## $SA19_high
##
## OUT: output value
## n: number of cases in configuration
## incl: sufficiency inclusion score
## PRI: proportional reduction in inconsistency
##
## Demandm Laborm Materialm RelCommm OUT n incl PRI
## 15 1 1 1 0 1 45 0.800 0.494
## 14 1 1 0 1 0 56 0.798 0.521
## 16 1 1 1 1 0 65 0.775 0.544
## 13 1 1 0 0 0 115 0.721 0.444
## 9 1 0 0 0 0 59 0.700 0.293
## 1 0 0 0 0 0 39 0.584 0.122
##
##

## $SA20_low
##
## OUT: output value
## n: number of cases in configuration
## incl: sufficiency inclusion score
## PRI: proportional reduction in inconsistency
##
## Demandm Laborm Materialm RelCommm OUT n incl PRI
## 16 1 1 1 1 1 65 0.830 0.650
## 15 1 1 1 0 1 45 0.827 0.576
## 14 1 1 0 1 1 56 0.825 0.586
## 13 1 1 0 0 0 115 0.767 0.522
## 9 1 0 0 0 0 59 0.714 0.326
## 1 0 0 0 0 0 39 0.613 0.170
##
##
## $SA20_high
##
## OUT: output value
## n: number of cases in configuration
## incl: sufficiency inclusion score
## PRI: proportional reduction in inconsistency
##
## Demandm Laborm Materialm RelCommm OUT n incl PRI
## 15 1 1 1 0 0 45 0.667 0.181
## 16 1 1 1 1 0 65 0.661 0.291
## 14 1 1 0 1 0 56 0.641 0.192
## 9 1 0 0 0 0 59 0.514 0.048
## 13 1 1 0 0 0 115 0.512 0.116
## 1 0 0 0 0 0 39 0.472 0.022
##
## It seems that all output values have been coded to zero.
## Suggestion: lower the inclusion score for the presence of the outcome,
## the relevant argument is "incl.cut" which now has a value of 0.8.
##
##

## $SA21_low
##
## OUT: output value
## n: number of cases in configuration
## incl: sufficiency inclusion score
## PRI: proportional reduction in inconsistency
##
## Demandm Laborm Materialm RelCommm OUT n incl PRI
## 15 1 1 1 0 1 45 0.944 0.878
## 14 1 1 0 1 1 56 0.933 0.867
## 16 1 1 1 1 1 65 0.929 0.872
## 13 1 1 0 0 1 115 0.908 0.835
## 9 1 0 0 0 1 59 0.884 0.744
## 1 0 0 0 0 0 39 0.729 0.412
##
##
## $SA21_high
##
## OUT: output value
## n: number of cases in configuration
## incl: sufficiency inclusion score
## PRI: proportional reduction in inconsistency
##
## Demandm Laborm Materialm RelCommm OUT n incl PRI
## 14 1 1 0 1 1 56 0.839 0.618
## 15 1 1 1 0 1 45 0.825 0.586
## 16 1 1 1 1 1 65 0.823 0.641
## 13 1 1 0 0 0 115 0.753 0.518
## 9 1 0 0 0 0 59 0.722 0.368
## 1 0 0 0 0 0 39 0.604 0.163
##
##

## $SA22_low
##
## OUT: output value
## n: number of cases in configuration
## incl: sufficiency inclusion score
## PRI: proportional reduction in inconsistency
##
## Demandm Laborm Materialm RelCommm OUT n incl PRI
## 14 1 1 0 1 1 56 0.958 0.928
## 15 1 1 1 0 1 45 0.948 0.910
## 13 1 1 0 0 1 115 0.944 0.911
## 16 1 1 1 1 1 65 0.938 0.901
## 9 1 0 0 0 1 59 0.909 0.831
## 1 0 0 0 0 0 39 0.742 0.494
##
##
## $SA22_high
##
## OUT: output value
## n: number of cases in configuration
## incl: sufficiency inclusion score
## PRI: proportional reduction in inconsistency
##
## Demandm Laborm Materialm RelCommm OUT n incl PRI
## 15 1 1 1 0 1 45 0.908 0.824
## 16 1 1 1 1 1 65 0.870 0.781
## 14 1 1 0 1 1 56 0.866 0.757
## 13 1 1 0 0 1 115 0.811 0.689
## 9 1 0 0 0 0 59 0.770 0.564
## 1 0 0 0 0 0 39 0.620 0.277
##
##

## $SA23_low
##
## OUT: output value
## n: number of cases in configuration
## incl: sufficiency inclusion score
## PRI: proportional reduction in inconsistency
##
## Demandm Laborm Materialm RelCommm OUT n incl PRI
## 16 1 1 1 1 1 65 0.845 0.674
## 15 1 1 1 0 1 45 0.839 0.575
## 14 1 1 0 1 1 56 0.836 0.601
## 13 1 1 0 0 0 115 0.796 0.557
## 9 1 0 0 0 0 59 0.775 0.406
## 1 0 0 0 0 0 39 0.599 0.123
##
##
## $SA23_high
##
## OUT: output value
## n: number of cases in configuration
## incl: sufficiency inclusion score
## PRI: proportional reduction in inconsistency
##
## Demandm Laborm Materialm RelCommm OUT n incl PRI
## 16 1 1 1 1 0 65 0.658 0.264
## 14 1 1 0 1 0 56 0.647 0.195
## 15 1 1 1 0 0 45 0.642 0.137
## 9 1 0 0 0 0 59 0.529 0.053
## 13 1 1 0 0 0 115 0.519 0.118
## 1 0 0 0 0 0 39 0.475 0.012
##
## It seems that all output values have been coded to zero.
## Suggestion: lower the inclusion score for the presence of the outcome,
## the relevant argument is "incl.cut" which now has a value of 0.8.
##
##

## $SA24_low
##
## OUT: output value
## n: number of cases in configuration
## incl: sufficiency inclusion score
## PRI: proportional reduction in inconsistency
##
## Demandm Laborm Materialm RelCommm OUT n incl PRI
## 15 1 1 1 0 0 45 0.773 0.442
## 16 1 1 1 1 0 65 0.773 0.534
## 14 1 1 0 1 0 56 0.755 0.437
## 9 1 0 0 0 0 59 0.706 0.331
## 13 1 1 0 0 0 115 0.700 0.399
## 1 0 0 0 0 0 39 0.596 0.125
##
## It seems that all output values have been coded to zero.
## Suggestion: lower the inclusion score for the presence of the outcome,
## the relevant argument is "incl.cut" which now has a value of 0.8.
##
##
## $SA24_high
##
## OUT: output value
## n: number of cases in configuration
## incl: sufficiency inclusion score
## PRI: proportional reduction in inconsistency
##
## Demandm Laborm Materialm RelCommm OUT n incl PRI
## 16 1 1 1 1 0 65 0.592 0.184
## 15 1 1 1 0 0 45 0.590 0.080
## 14 1 1 0 1 0 56 0.557 0.100
## 9 1 0 0 0 0 59 0.504 0.063
## 1 0 0 0 0 0 39 0.465 0.014
## 13 1 1 0 0 0 115 0.456 0.073
##
## It seems that all output values have been coded to zero.
## Suggestion: lower the inclusion score for the presence of the outcome,
## the relevant argument is "incl.cut" which now has a value of 0.8.
##
##

## $SA25_low
##
## OUT: output value
## n: number of cases in configuration
## incl: sufficiency inclusion score
## PRI: proportional reduction in inconsistency
##
## Demandm Laborm Materialm RelCommm OUT n incl PRI
## 14 1 1 0 1 1 56 0.958 0.920
## 15 1 1 1 0 1 45 0.948 0.889
## 13 1 1 0 0 1 115 0.942 0.901
## 16 1 1 1 1 1 65 0.940 0.893
## 9 1 0 0 0 1 59 0.923 0.834
## 1 0 0 0 0 0 39 0.754 0.465
##
##
## $SA25_high
##
## OUT: output value
## n: number of cases in configuration
## incl: sufficiency inclusion score
## PRI: proportional reduction in inconsistency
##
## Demandm Laborm Materialm RelCommm OUT n incl PRI
## 14 1 1 0 1 1 56 0.885 0.736
## 15 1 1 1 0 1 45 0.853 0.627
## 16 1 1 1 1 1 65 0.852 0.700
## 13 1 1 0 0 1 115 0.805 0.608
## 9 1 0 0 0 0 59 0.775 0.448
## 1 0 0 0 0 0 39 0.627 0.190

#### Performing QCA: Necessity analysis: Reasons MNC -> MNC

# Result: Necessity Analysis: Single Reasons for MNC necessary for single Outcomes of MNC

## $SA1_low
##
## inclN RoN covN
## ---------------------------------
## 1 Demandm 0.892 0.655 0.834
## 2 Laborm 0.820 0.821 0.887
## 3 Materialm 0.405 0.952 0.895
## 4 RelCommm 0.492 0.956 0.925
## ---------------------------------
##
##
## $SA1_high
##
## inclN RoN covN
## ---------------------------------
## 1 Demandm 0.922 0.457 0.625
## 2 Laborm 0.889 0.633 0.698
## 3 Materialm 0.458 0.888 0.734
## 4 RelCommm 0.583 0.889 0.795
## ---------------------------------
##
##
## $SA2_low
##
## inclN RoN covN
## ---------------------------------
## 1 Demandm 0.911 0.451 0.616
## 2 Laborm 0.867 0.619 0.679
## 3 Materialm 0.445 0.880 0.711
## 4 RelCommm 0.554 0.869 0.753
## ---------------------------------
##
##
## $SA2_high
##
## inclN RoN covN
## ---------------------------------
## 1 Demandm 0.942 0.330 0.361
## 2 Laborm 0.932 0.470 0.413
## 3 Materialm 0.541 0.805 0.490
## 4 RelCommm 0.707 0.783 0.544
## ---------------------------------
##
##

## $SA3_low
##
## inclN RoN covN
## ---------------------------------
## 1 Demandm 0.929 0.461 0.632
## 2 Laborm 0.867 0.621 0.682
## 3 Materialm 0.478 0.901 0.769
## 4 RelCommm 0.549 0.868 0.750
## ---------------------------------
##
##
## $SA3_high
##
## inclN RoN covN
## ---------------------------------
## 1 Demandm 0.947 0.344 0.400
## 2 Laborm 0.930 0.489 0.455
## 3 Materialm 0.557 0.826 0.556
## 4 RelCommm 0.683 0.796 0.580
## ---------------------------------
##
##
## $SA4_low
##
## inclN RoN covN
## ---------------------------------
## 1 Demandm 0.927 0.423 0.571
## 2 Laborm 0.888 0.587 0.633
## 3 Materialm 0.485 0.878 0.707
## 4 RelCommm 0.585 0.856 0.725
## ---------------------------------
##
##
## $SA4_high
##
## inclN RoN covN
## ---------------------------------
## 1 Demandm 0.941 0.315 0.315
## 2 Laborm 0.947 0.452 0.367
## 3 Materialm 0.617 0.805 0.488
## 4 RelCommm 0.746 0.767 0.501
## ---------------------------------
##
##
## $SA5_low
##
## inclN RoN covN
## ---------------------------------
## 1 Demandm 0.917 0.515 0.703
## 2 Laborm 0.862 0.689 0.765
## 3 Materialm 0.456 0.924 0.826
## 4 RelCommm 0.541 0.907 0.833
## ---------------------------------
##
##

## $SA5_high
##
## inclN RoN covN
## ---------------------------------
## 1 Demandm 0.944 0.356 0.430
## 2 Laborm 0.938 0.508 0.495
## 3 Materialm 0.561 0.842 0.604
## 4 RelCommm 0.677 0.812 0.619
## ---------------------------------
##
##
## $SA6_low
##
## inclN RoN covN
## ---------------------------------
## 1 Demandm 0.918 0.488 0.669
## 2 Laborm 0.867 0.660 0.732
## 3 Materialm 0.450 0.904 0.775
## 4 RelCommm 0.555 0.898 0.814
## ---------------------------------
##
##
## $SA6_high
##
## inclN RoN covN
## ---------------------------------
## 1 Demandm 0.944 0.351 0.418
## 2 Laborm 0.940 0.501 0.481
## 3 Materialm 0.564 0.837 0.590
## 4 RelCommm 0.678 0.805 0.603
## ---------------------------------
##
##
## $SA7_low
##
## inclN RoN covN
## ---------------------------------
## 1 Demandm 0.912 0.495 0.679
## 2 Laborm 0.859 0.667 0.740
## 3 Materialm 0.451 0.910 0.792
## 4 RelCommm 0.558 0.908 0.833
## ---------------------------------
##
##
## $SA7_high
##
## inclN RoN covN
## ---------------------------------
## 1 Demandm 0.946 0.356 0.431
## 2 Laborm 0.923 0.504 0.486
## 3 Materialm 0.538 0.834 0.579
## 4 RelCommm 0.692 0.817 0.633
## ---------------------------------
##
##

## $SA8_low
##
## inclN RoN covN
## ---------------------------------
## 1 Demandm 0.884 0.606 0.795
## 2 Laborm 0.813 0.772 0.846
## 3 Materialm 0.410 0.942 0.870
## 4 RelCommm 0.492 0.937 0.889
## ---------------------------------
##
##
## $SA8_high
##
## inclN RoN covN
## ---------------------------------
## 1 Demandm 0.912 0.430 0.583
## 2 Laborm 0.889 0.604 0.658
## 3 Materialm 0.482 0.886 0.728
## 4 RelCommm 0.596 0.875 0.765
## ---------------------------------
##
##
## $SA9a_low
##
## inclN RoN covN
## ---------------------------------
## 1 Demandm 0.868 0.740 0.890
## 2 Laborm 0.781 0.876 0.926
## 3 Materialm 0.387 0.971 0.937
## 4 RelCommm 0.464 0.974 0.956
## ---------------------------------
##
##
## $SA9a_high
##
## inclN RoN covN
## ---------------------------------
## 1 Demandm 0.904 0.558 0.751
## 2 Laborm 0.845 0.735 0.812
## 3 Materialm 0.434 0.934 0.851
## 4 RelCommm 0.532 0.936 0.887
## ---------------------------------
##
##
## $SA9b_low
##
## inclN RoN covN
## ---------------------------------
## 1 Demandm 0.869 0.742 0.891
## 2 Laborm 0.780 0.874 0.925
## 3 Materialm 0.383 0.967 0.927
## 4 RelCommm 0.462 0.971 0.951
## ---------------------------------
##
##

## $SA9b_high
##
## inclN RoN covN
## ---------------------------------
## 1 Demandm 0.900 0.556 0.749
## 2 Laborm 0.839 0.731 0.808
## 3 Materialm 0.428 0.930 0.842
## 4 RelCommm 0.525 0.931 0.878
## ---------------------------------
##
##
## $SA10a_low
##
## inclN RoN covN
## ---------------------------------
## 1 Demandm 0.868 0.736 0.887
## 2 Laborm 0.780 0.871 0.923
## 3 Materialm 0.387 0.970 0.935
## 4 RelCommm 0.462 0.970 0.949
## ---------------------------------
##
##
## $SA10a_high
##
## inclN RoN covN
## ---------------------------------
## 1 Demandm 0.900 0.546 0.738
## 2 Laborm 0.841 0.720 0.798
## 3 Materialm 0.427 0.925 0.828
## 4 RelCommm 0.525 0.924 0.864
## ---------------------------------
##
##
## $SA10b_low
##
## inclN RoN covN
## ---------------------------------
## 1 Demandm 0.860 0.809 0.926
## 2 Laborm 0.766 0.920 0.954
## 3 Materialm 0.379 0.983 0.965
## 4 RelCommm 0.451 0.985 0.975
## ---------------------------------
##
##
## $SA10b_high
##
## inclN RoN covN
## ---------------------------------
## 1 Demandm 0.888 0.635 0.819
## 2 Laborm 0.822 0.809 0.877
## 3 Materialm 0.412 0.954 0.898
## 4 RelCommm 0.501 0.958 0.928
## ---------------------------------
##
##

## $SA11_low
##
## inclN RoN covN
## ---------------------------------
## 1 Demandm 0.932 0.445 0.607
## 2 Laborm 0.883 0.609 0.665
## 3 Materialm 0.468 0.883 0.721
## 4 RelCommm 0.573 0.868 0.750
## ---------------------------------
##
##
## $SA11_high
##
## inclN RoN covN
## ---------------------------------
## 1 Demandm 0.947 0.311 0.303
## 2 Laborm 0.960 0.447 0.356
## 3 Materialm 0.618 0.798 0.468
## 4 RelCommm 0.777 0.766 0.500
## ---------------------------------
##
##
## $SA12_low
##
## inclN RoN covN
## ---------------------------------
## 1 Demandm 0.914 0.512 0.701
## 2 Laborm 0.858 0.686 0.761
## 3 Materialm 0.430 0.905 0.779
## 4 RelCommm 0.533 0.902 0.821
## ---------------------------------
##
##
## $SA12_high
##
## inclN RoN covN
## ---------------------------------
## 1 Demandm 0.926 0.362 0.445
## 2 Laborm 0.919 0.516 0.511
## 3 Materialm 0.528 0.841 0.601
## 4 RelCommm 0.666 0.822 0.644
## ---------------------------------
##
##
## $SA13_low
##
## inclN RoN covN
## ---------------------------------
## 1 Demandm 0.921 0.362 0.445
## 2 Laborm 0.894 0.510 0.499
## 3 Materialm 0.505 0.832 0.576
## 4 RelCommm 0.638 0.812 0.619
## ---------------------------------
##
##

## $SA13_high
##
## inclN RoN covN
## ---------------------------------
## 1 Demandm 0.945 0.300 0.264
## 2 Laborm 0.944 0.429 0.305
## 3 Materialm 0.600 0.777 0.396
## 4 RelCommm 0.787 0.746 0.442
## ---------------------------------
##
##
## $SA14a_low
##
## inclN RoN covN
## ---------------------------------
## 1 Demandm 0.913 0.512 0.700
## 2 Laborm 0.861 0.688 0.764
## 3 Materialm 0.449 0.919 0.814
## 4 RelCommm 0.544 0.910 0.837
## ---------------------------------
##
##
## $SA14a_high
##
## inclN RoN covN
## ---------------------------------
## 1 Demandm 0.933 0.355 0.428
## 2 Laborm 0.926 0.506 0.491
## 3 Materialm 0.537 0.835 0.582
## 4 RelCommm 0.693 0.820 0.639
## ---------------------------------
##
##
## $SA14b_low
##
## inclN RoN covN
## ---------------------------------
## 1 Demandm 0.902 0.650 0.831
## 2 Laborm 0.827 0.814 0.881
## 3 Materialm 0.417 0.958 0.907
## 4 RelCommm 0.509 0.965 0.941
## ---------------------------------
##
##
## $SA14b_high
##
## inclN RoN covN
## ---------------------------------
## 1 Demandm 0.930 0.450 0.614
## 2 Laborm 0.883 0.616 0.675
## 3 Materialm 0.481 0.894 0.751
## 4 RelCommm 0.604 0.892 0.802
## ---------------------------------
##
##

## $SA14c_low
##
## inclN RoN covN
## ---------------------------------
## 1 Demandm 0.885 0.693 0.860
## 2 Laborm 0.807 0.849 0.908
## 3 Materialm 0.400 0.963 0.919
## 4 RelCommm 0.487 0.971 0.951
## ---------------------------------
##
##
## $SA14c_high
##
## inclN RoN covN
## ---------------------------------
## 1 Demandm 0.914 0.507 0.694
## 2 Laborm 0.861 0.681 0.756
## 3 Materialm 0.441 0.910 0.791
## 4 RelCommm 0.562 0.920 0.857
## ---------------------------------
##
##
## $SA15_low
##
## inclN RoN covN
## ---------------------------------
## 1 Demandm 0.942 0.348 0.411
## 2 Laborm 0.909 0.490 0.458
## 3 Materialm 0.517 0.819 0.533
## 4 RelCommm 0.628 0.785 0.550
## ---------------------------------
##
##
## $SA15_high
##
## inclN RoN covN
## ---------------------------------
## 1 Demandm 0.949 0.286 0.216
## 2 Laborm 0.963 0.411 0.253
## 3 Materialm 0.664 0.766 0.357
## 4 RelCommm 0.811 0.723 0.370
## ---------------------------------
##
##
## $SA16_low
##
## inclN RoN covN
## ---------------------------------
## 1 Demandm 0.901 0.562 0.754
## 2 Laborm 0.836 0.732 0.809
## 3 Materialm 0.427 0.931 0.844
## 4 RelCommm 0.523 0.931 0.879
## ---------------------------------
##
##

## $SA16_high
##
## inclN RoN covN
## ---------------------------------
## 1 Demandm 0.927 0.384 0.496
## 2 Laborm 0.914 0.546 0.566
## 3 Materialm 0.501 0.852 0.634
## 4 RelCommm 0.656 0.848 0.705
## ---------------------------------
##
##
## $SA17_low
##
## inclN RoN covN
## ---------------------------------
## 1 Demandm 0.890 0.622 0.809
## 2 Laborm 0.811 0.780 0.853
## 3 Materialm 0.411 0.947 0.882
## 4 RelCommm 0.494 0.943 0.901
## ---------------------------------
##
##
## $SA17_high
##
## inclN RoN covN
## ---------------------------------
## 1 Demandm 0.906 0.451 0.617
## 2 Laborm 0.869 0.623 0.685
## 3 Materialm 0.464 0.893 0.748
## 4 RelCommm 0.571 0.882 0.781
## ---------------------------------
##
##
## $SA18a_low
##
## inclN RoN covN
## ---------------------------------
## 1 Demandm 0.909 0.434 0.590
## 2 Laborm 0.865 0.598 0.650
## 3 Materialm 0.459 0.877 0.705
## 4 RelCommm 0.560 0.859 0.730
## ---------------------------------
##
##
## $SA18a_high
##
## inclN RoN covN
## ---------------------------------
## 1 Demandm 0.915 0.324 0.343
## 2 Laborm 0.911 0.463 0.395
## 3 Materialm 0.526 0.798 0.466
## 4 RelCommm 0.692 0.774 0.522
## ---------------------------------
##
##

## $SA18b_low
##
## inclN RoN covN
## ---------------------------------
## 1 Demandm 0.921 0.384 0.495
## 2 Laborm 0.880 0.535 0.547
## 3 Materialm 0.475 0.841 0.603
## 4 RelCommm 0.594 0.820 0.640
## ---------------------------------
##
##
## $SA18b_high
##
## inclN RoN covN
## ---------------------------------
## 1 Demandm 0.935 0.300 0.266
## 2 Laborm 0.949 0.431 0.312
## 3 Materialm 0.575 0.774 0.386
## 4 RelCommm 0.760 0.744 0.434
## ---------------------------------
##
##
## $SA19_low
##
## inclN RoN covN
## ---------------------------------
## 1 Demandm 0.891 0.601 0.791
## 2 Laborm 0.828 0.777 0.850
## 3 Materialm 0.415 0.942 0.870
## 4 RelCommm 0.502 0.940 0.894
## ---------------------------------
##
##
## $SA19_high
##
## inclN RoN covN
## ---------------------------------
## 1 Demandm 0.929 0.413 0.553
## 2 Laborm 0.899 0.578 0.619
## 3 Materialm 0.487 0.870 0.686
## 4 RelCommm 0.607 0.857 0.727
## ---------------------------------
##
##
## $SA20_low
##
## inclN RoN covN
## ---------------------------------
## 1 Demandm 0.920 0.458 0.628
## 2 Laborm 0.888 0.636 0.702
## 3 Materialm 0.466 0.895 0.752
## 4 RelCommm 0.572 0.884 0.784
## ---------------------------------
##
##

## $SA20_high
##
## inclN RoN covN
## ---------------------------------
## 1 Demandm 0.952 0.328 0.355
## 2 Laborm 0.963 0.471 0.415
## 3 Materialm 0.621 0.823 0.547
## 4 RelCommm 0.761 0.792 0.570
## ---------------------------------
##
##
## $SA21_low
##
## inclN RoN covN
## ---------------------------------
## 1 Demandm 0.893 0.619 0.807
## 2 Laborm 0.819 0.784 0.856
## 3 Materialm 0.414 0.948 0.884
## 4 RelCommm 0.496 0.943 0.901
## ---------------------------------
##
##
## $SA21_high
##
## inclN RoN covN
## ---------------------------------
## 1 Demandm 0.922 0.434 0.589
## 2 Laborm 0.887 0.602 0.656
## 3 Materialm 0.480 0.885 0.725
## 4 RelCommm 0.599 0.877 0.769
## ---------------------------------
##
##
## $SA22_low
##
## inclN RoN covN
## ---------------------------------
## 1 Demandm 0.877 0.695 0.862
## 2 Laborm 0.793 0.841 0.901
## 3 Materialm 0.390 0.957 0.906
## 4 RelCommm 0.471 0.958 0.928
## ---------------------------------
##
##
## $SA22_high
##
## inclN RoN covN
## ---------------------------------
## 1 Demandm 0.899 0.517 0.706
## 2 Laborm 0.838 0.686 0.761
## 3 Materialm 0.444 0.923 0.825
## 4 RelCommm 0.523 0.904 0.825
## ---------------------------------
##
##

## $SA23_low
##
## inclN RoN covN
## ---------------------------------
## 1 Demandm 0.937 0.478 0.656
## 2 Laborm 0.884 0.647 0.715
## 3 Materialm 0.468 0.903 0.773
## 4 RelCommm 0.571 0.893 0.802
## ---------------------------------
##
##
## $SA23_high
##
## inclN RoN covN
## ---------------------------------
## 1 Demandm 0.957 0.329 0.359
## 2 Laborm 0.965 0.473 0.419
## 3 Materialm 0.601 0.819 0.533
## 4 RelCommm 0.758 0.793 0.571
## ---------------------------------
##
##
## $SA24_low
##
## inclN RoN covN
## ---------------------------------
## 1 Demandm 0.927 0.424 0.573
## 2 Laborm 0.876 0.583 0.627
## 3 Materialm 0.485 0.879 0.709
## 4 RelCommm 0.575 0.852 0.715
## ---------------------------------
##
##
## $SA24_high
##
## inclN RoN covN
## ---------------------------------
## 1 Demandm 0.963 0.312 0.306
## 2 Laborm 0.953 0.445 0.350
## 3 Materialm 0.629 0.800 0.472
## 4 RelCommm 0.764 0.762 0.487
## ---------------------------------
##
##
## $SA25_low
##
## inclN RoN covN
## ---------------------------------
## 1 Demandm 0.889 0.677 0.850
## 2 Laborm 0.805 0.825 0.890
## 3 Materialm 0.399 0.955 0.901
## 4 RelCommm 0.484 0.959 0.930
## ---------------------------------
##
##

## $SA25_high
##
## inclN RoN covN
## ---------------------------------
## 1 Demandm 0.919 0.476 0.653
## 2 Laborm 0.878 0.652 0.722
## 3 Materialm 0.456 0.900 0.765
## 4 RelCommm 0.577 0.903 0.823
## ---------------------------------

# Result: Necessity Analysis: Sets of Reasons for MNC necessary for single Outcomes of MNC

## $SA1_low
##
## inclN RoN covN
## -------------------------------------------
## 1 Demandm + Laborm 0.937 0.581 0.826
## 2 Demandm + Materialm 0.903 0.635 0.831
## 3 Demandm + RelCommm 0.907 0.632 0.831
## -------------------------------------------
##
##
## $SA1_high
## [1] "Error: There are no configurations, using these cutoff values."
##
## $SA2_low
## [1] "Error: There are no configurations, using these cutoff values."
##
## $SA2_high
## [1] "Error: There are no configurations, using these cutoff values."
##
## $SA3_low
## [1] "Error: There are no configurations, using these cutoff values."
##
## $SA3_high
## [1] "Error: There are no configurations, using these cutoff values."
##
## $SA4_low
## [1] "Error: There are no configurations, using these cutoff values."
##
## $SA4_high
## [1] "Error: There are no configurations, using these cutoff values."
##
## $SA5_low
## [1] "Error: There are no configurations, using these cutoff values."
##
## $SA5_high
## [1] "Error: There are no configurations, using these cutoff values."
##
## $SA6_low
## [1] "Error: There are no configurations, using these cutoff values."
##
## $SA6_high
## [1] "Error: There are no configurations, using these cutoff values."
##
## $SA7_low
## [1] "Error: There are no configurations, using these cutoff values."
##
## $SA7_high
## [1] "Error: There are no configurations, using these cutoff values."
##

## $SA8_low
##
## inclN RoN covN
## ------------------------------------------
## 1 Demandm + Laborm 0.931 0.535 0.791
## 2 Demandm + RelCommm 0.901 0.585 0.795
## ------------------------------------------
##
##
## $SA8_high
## [1] "Error: There are no configurations, using these cutoff values."
##
## $SA9a_low
##
## inclN RoN covN
## ------------------------------------------
## 1 Demandm + ~Laborm 0.915 0.582 0.856
## 2 Demandm + Laborm 0.916 0.679 0.886
## 3 Laborm + ~RelCommm 0.932 0.563 0.859
## ------------------------------------------
##
##
## $SA9a_high
##
## inclN RoN covN
## -------------------------------
## 1 Demandm 0.904 0.558 0.751
## -------------------------------
##
##
## $SA9b_low
##
## inclN RoN covN
## ------------------------------------------
## 1 Demandm + ~Laborm 0.913 0.580 0.855
## 2 Demandm + Laborm 0.918 0.682 0.888
## 3 Laborm + ~RelCommm 0.932 0.563 0.859
## ------------------------------------------
##
##
## $SA9b_high
## [1] "Error: There are no configurations, using these cutoff values."
##
## $SA10a_low
##
## inclN RoN covN
## ------------------------------------------
## 1 Demandm + ~Laborm 0.915 0.578 0.854
## 2 Demandm + Laborm 0.913 0.669 0.881
## 3 Laborm + ~RelCommm 0.934 0.563 0.858
## ------------------------------------------
##
##

## $SA10a_high
## [1] "Error: There are no configurations, using these cutoff values."
##
## $SA10b_low
##
## inclN RoN covN
## -------------------------------------------
## 1 Demandm + ~Laborm 0.908 0.651 0.893
## 2 Demandm + Laborm 0.907 0.756 0.922
## 3 Demandm + ~RelCommm 0.941 0.535 0.879
## 4 Laborm + ~Materialm 0.940 0.558 0.883
## 5 Laborm + ~RelCommm 0.922 0.628 0.892
## -------------------------------------------
##
##
## $SA10b_high
##
## inclN RoN covN
## ------------------------------------------
## 1 Demandm + Laborm 0.937 0.567 0.816
## 2 Demandm + RelCommm 0.904 0.614 0.818
## ------------------------------------------
##
##
## $SA11_low
## [1] "Error: There are no configurations, using these cutoff values."
##
## $SA11_high
## [1] "Error: There are no configurations, using these cutoff values."
##
## $SA12_low
## [1] "Error: There are no configurations, using these cutoff values."
##
## $SA12_high
## [1] "Error: There are no configurations, using these cutoff values."
##
## $SA13_low
## [1] "Error: There are no configurations, using these cutoff values."
##
## $SA13_high
## [1] "Error: There are no configurations, using these cutoff values."
##
## $SA14a_low
## [1] "Error: There are no configurations, using these cutoff values."
##
## $SA14a_high
## [1] "Error: There are no configurations, using these cutoff values."
##

## $SA14b_low
##
## inclN RoN covN
## -------------------------------
## 1 Demandm 0.902 0.650 0.831
## -------------------------------
##
##
## $SA14b_high
## [1] "Error: There are no configurations, using these cutoff values."
##
## $SA14c_low
##
## inclN RoN covN
## ------------------------------------------
## 1 Demandm + ~Laborm 0.920 0.522 0.816
## 2 Demandm + Laborm 0.933 0.627 0.856
## 3 Demandm + RelCommm 0.903 0.675 0.861
## ------------------------------------------
##
##
## $SA14c_high
## [1] "Error: There are no configurations, using these cutoff values."
##
## $SA15_low
## [1] "Error: There are no configurations, using these cutoff values."
##
## $SA15_high
## [1] "Error: There are no configurations, using these cutoff values."
##
## $SA16_low
##
## inclN RoN covN
## -------------------------------
## 1 Demandm 0.901 0.562 0.754
## -------------------------------
##
##
## $SA16_high
## [1] "Error: There are no configurations, using these cutoff values."
##

## $SA17_low
##
## inclN RoN covN
## -------------------------------------------
## 1 Demandm + Laborm 0.933 0.547 0.801
## 2 Demandm + Materialm 0.900 0.602 0.806
## 3 Demandm + RelCommm 0.907 0.602 0.809
## -------------------------------------------
##
##
## $SA17_high
## [1] "Error: There are no configurations, using these cutoff values."
##
## $SA18a_low
## [1] "Error: There are no configurations, using these cutoff values."
##
## $SA18a_high
## [1] "Error: There are no configurations, using these cutoff values."
##
## $SA18b_low
## [1] "Error: There are no configurations, using these cutoff values."
##
## $SA18b_high
## [1] "Error: There are no configurations, using these cutoff values."
##
## $SA19_low
##
## inclN RoN covN
## -------------------------------------------
## 1 Demandm + Laborm 0.935 0.527 0.784
## 2 Demandm + Materialm 0.902 0.581 0.788
## 3 Demandm + RelCommm 0.906 0.578 0.789
## -------------------------------------------
##
##
## $SA19_high
## [1] "Error: There are no configurations, using these cutoff values."
##
## $SA20_low
## [1] "Error: There are no configurations, using these cutoff values."
##
## $SA20_high
## [1] "Error: There are no configurations, using these cutoff values."
##
## $SA21_low
##
## inclN RoN covN
## -------------------------------------------
## 1 Demandm + Laborm 0.937 0.546 0.800
## 2 Demandm + Materialm 0.904 0.600 0.804
## 3 Demandm + RelCommm 0.910 0.600 0.807
## -------------------------------------------
##
##
## $SA21_high
## [1] "Error: There are no configurations, using these cutoff values."
##
## $SA22_low
##
## inclN RoN covN
## ------------------------------------------------------
## 1 Demandm + ~Laborm 0.914 0.526 0.820
## 2 Demandm + Laborm 0.926 0.630 0.858
## 3 Laborm + ~RelCommm 0.930 0.504 0.821
## 4 Demandm + Materialm + RelCommm 0.901 0.663 0.859
## ------------------------------------------------------
##
##
## $SA22_high
## [1] "Error: There are no configurations, using these cutoff values."
##
## $SA23_low
## [1] "Error: There are no configurations, using these cutoff values."
##
## $SA23_high
## [1] "Error: There are no configurations, using these cutoff values."
##
## $SA24_low
## [1] "Error: There are no configurations, using these cutoff values."
##
## $SA24_high
## [1] "Error: There are no configurations, using these cutoff values."
##
## $SA25_low
##
## inclN RoN covN
## -------------------------------------------
## 1 Demandm + ~Laborm 0.928 0.513 0.810
## 2 Demandm + Laborm 0.934 0.604 0.842
## 3 Demandm + Materialm 0.901 0.659 0.848
## 4 Demandm + RelCommm 0.906 0.658 0.849
## -------------------------------------------
##
##
## $SA25_high
## [1] "Error: There are no configurations, using these cutoff values."

#### Performing QCA: Sufficiency analysis: Reasons MNC -> MNC
# create processed lists based on QCA::pof function, which applies the following

# Result: Sufficiency Analysis: Single Reasons for MNC sufficient for single Outcomes of MNC

## $SA1_low
##
## inclS PRI covS covU
## ----------------------------------------
## 1 Demandm 0.834 0.784 0.892 0.102
## 2 Laborm 0.887 0.846 0.820 0.026
## 3 Materialm 0.895 0.835 0.405 0.003
## 4 RelCommm 0.925 0.880 0.492 0.000
## ----------------------------------------
##
##
## $SA1_high
##
## inclS PRI covS covU
## ----------------------------------------
## 1 Demandm 0.625 0.480 0.922 0.068
## 2 Laborm 0.698 0.553 0.889 0.026
## 3 Materialm 0.734 0.535 0.458 0.001
## 4 RelCommm 0.795 0.624 0.583 0.000
## ----------------------------------------
##
##
## $SA2_low
##
## inclS PRI covS covU
## ----------------------------------------
## 1 Demandm 0.616 0.473 0.911 0.079
## 2 Laborm 0.679 0.534 0.867 0.028
## 3 Materialm 0.711 0.512 0.445 0.002
## 4 RelCommm 0.753 0.571 0.554 0.000
## ----------------------------------------
##
##
## $SA2_high
##
## inclS PRI covS covU
## ----------------------------------------
## 1 Demandm 0.361 0.159 0.942 0.040
## 2 Laborm 0.413 0.188 0.932 0.019
## 3 Materialm 0.490 0.191 0.541 0.002
## 4 RelCommm 0.544 0.240 0.707 0.000
## ----------------------------------------
##
##

## $SA3_low
##
## inclS PRI covS covU
## ----------------------------------------
## 1 Demandm 0.632 0.511 0.929 0.084
## 2 Laborm 0.682 0.559 0.867 0.019
## 3 Materialm 0.769 0.614 0.478 0.001
## 4 RelCommm 0.750 0.593 0.549 0.000
## ----------------------------------------
##
##
## $SA3_high
##
## inclS PRI covS covU
## ----------------------------------------
## 1 Demandm 0.400 0.219 0.947 0.040
## 2 Laborm 0.455 0.254 0.930 0.014
## 3 Materialm 0.556 0.281 0.557 0.000
## 4 RelCommm 0.580 0.315 0.683 0.000
## ----------------------------------------
##
##
## $SA4_low
##
## inclS PRI covS covU
## ----------------------------------------
## 1 Demandm 0.571 0.413 0.927 0.068
## 2 Laborm 0.633 0.471 0.888 0.020
## 3 Materialm 0.707 0.504 0.485 0.002
## 4 RelCommm 0.725 0.520 0.585 0.000
## ----------------------------------------
##
##
## $SA4_high
##
## inclS PRI covS covU
## ----------------------------------------
## 1 Demandm 0.315 0.124 0.941 0.029
## 2 Laborm 0.367 0.152 0.947 0.015
## 3 Materialm 0.488 0.193 0.617 0.001
## 4 RelCommm 0.501 0.196 0.746 0.000
## ----------------------------------------
##
##

## $SA5_low
##
## inclS PRI covS covU
## ----------------------------------------
## 1 Demandm 0.703 0.584 0.917 0.080
## 2 Laborm 0.765 0.651 0.862 0.023
## 3 Materialm 0.826 0.700 0.456 0.002
## 4 RelCommm 0.833 0.699 0.541 0.001
## ----------------------------------------
##
##
## $SA5_high
##
## inclS PRI covS covU
## ----------------------------------------
## 1 Demandm 0.430 0.231 0.944 0.036
## 2 Laborm 0.495 0.277 0.938 0.017
## 3 Materialm 0.604 0.323 0.561 0.001
## 4 RelCommm 0.619 0.325 0.677 0.001
## ----------------------------------------
##
##
## $SA6_low
##
## inclS PRI covS covU
## ----------------------------------------
## 1 Demandm 0.669 0.547 0.918 0.082
## 2 Laborm 0.732 0.615 0.867 0.022
## 3 Materialm 0.775 0.636 0.450 0.003
## 4 RelCommm 0.814 0.676 0.555 0.001
## ----------------------------------------
##
##
## $SA6_high
##
## inclS PRI covS covU
## ----------------------------------------
## 1 Demandm 0.418 0.229 0.944 0.036
## 2 Laborm 0.481 0.277 0.940 0.019
## 3 Materialm 0.590 0.334 0.564 0.001
## 4 RelCommm 0.603 0.322 0.678 0.001
## ----------------------------------------
##
##

## $SA7_low
##
## inclS PRI covS covU
## ----------------------------------------
## 1 Demandm 0.679 0.563 0.912 0.082
## 2 Laborm 0.740 0.628 0.859 0.025
## 3 Materialm 0.792 0.648 0.451 0.001
## 4 RelCommm 0.833 0.713 0.558 0.000
## ----------------------------------------
##
##
## $SA7_high
##
## inclS PRI covS covU
## ----------------------------------------
## 1 Demandm 0.431 0.246 0.946 0.051
## 2 Laborm 0.486 0.284 0.923 0.019
## 3 Materialm 0.579 0.314 0.538 0.000
## 4 RelCommm 0.633 0.366 0.692 0.000
## ----------------------------------------
##
##
## $SA8_low
##
## inclS PRI covS covU
## ----------------------------------------
## 1 Demandm 0.795 0.733 0.884 0.104
## 2 Laborm 0.846 0.791 0.813 0.026
## 3 Materialm 0.870 0.799 0.410 0.002
## 4 RelCommm 0.889 0.824 0.492 0.001
## ----------------------------------------
##
##
## $SA8_high
##
## inclS PRI covS covU
## ----------------------------------------
## 1 Demandm 0.583 0.431 0.912 0.060
## 2 Laborm 0.658 0.505 0.889 0.026
## 3 Materialm 0.728 0.540 0.482 0.002
## 4 RelCommm 0.765 0.580 0.596 0.001
## ----------------------------------------
##
##

## $SA9a_low
##
## inclS PRI covS covU
## ----------------------------------------
## 1 Demandm 0.890 0.864 0.868 0.118
## 2 Laborm 0.926 0.906 0.781 0.029
## 3 Materialm 0.937 0.910 0.387 0.003
## 4 RelCommm 0.956 0.938 0.464 0.001
## ----------------------------------------
##
##
## $SA9a_high
##
## inclS PRI covS covU
## ----------------------------------------
## 1 Demandm 0.751 0.673 0.904 0.090
## 2 Laborm 0.812 0.742 0.845 0.028
## 3 Materialm 0.851 0.764 0.434 0.001
## 4 RelCommm 0.887 0.818 0.532 0.001
## ----------------------------------------
##
##
## $SA9b_low
##
## inclS PRI covS covU
## ----------------------------------------
## 1 Demandm 0.891 0.865 0.869 0.122
## 2 Laborm 0.925 0.905 0.780 0.030
## 3 Materialm 0.927 0.896 0.383 0.003
## 4 RelCommm 0.951 0.930 0.462 0.001
## ----------------------------------------
##
##
## $SA9b_high
##
## inclS PRI covS covU
## ----------------------------------------
## 1 Demandm 0.749 0.667 0.900 0.092
## 2 Laborm 0.808 0.732 0.839 0.027
## 3 Materialm 0.842 0.747 0.428 0.003
## 4 RelCommm 0.878 0.798 0.525 0.001
## ----------------------------------------
##
##

## $SA10a_low
##
## inclS PRI covS covU
## ----------------------------------------
## 1 Demandm 0.887 0.861 0.868 0.118
## 2 Laborm 0.923 0.901 0.780 0.027
## 3 Materialm 0.935 0.906 0.387 0.002
## 4 RelCommm 0.949 0.926 0.462 0.001
## ----------------------------------------
##
##
## $SA10a_high
##
## inclS PRI covS covU
## ----------------------------------------
## 1 Demandm 0.738 0.655 0.900 0.090
## 2 Laborm 0.798 0.721 0.841 0.027
## 3 Materialm 0.828 0.729 0.427 0.001
## 4 RelCommm 0.864 0.778 0.525 0.001
## ----------------------------------------
##
##
## $SA10b_low
##
## inclS PRI covS covU
## ----------------------------------------
## 1 Demandm 0.926 0.912 0.860 0.125
## 2 Laborm 0.954 0.944 0.766 0.029
## 3 Materialm 0.965 0.951 0.379 0.003
## 4 RelCommm 0.975 0.967 0.451 0.001
## ----------------------------------------
##
##
## $SA10b_high
##
## inclS PRI covS covU
## ----------------------------------------
## 1 Demandm 0.819 0.769 0.888 0.100
## 2 Laborm 0.877 0.837 0.822 0.031
## 3 Materialm 0.898 0.847 0.412 0.003
## 4 RelCommm 0.928 0.891 0.501 0.001
## ----------------------------------------
##
##

## $SA11_low
##
## inclS PRI covS covU
## ----------------------------------------
## 1 Demandm 0.607 0.433 0.932 0.076
## 2 Laborm 0.665 0.487 0.883 0.016
## 3 Materialm 0.721 0.501 0.468 0.002
## 4 RelCommm 0.750 0.537 0.573 0.000
## ----------------------------------------
##
##
## $SA11_high
##
## inclS PRI covS covU
## ----------------------------------------
## 1 Demandm 0.303 0.093 0.947 0.019
## 2 Laborm 0.356 0.120 0.960 0.016
## 3 Materialm 0.468 0.143 0.618 0.000
## 4 RelCommm 0.500 0.168 0.777 0.000
## ----------------------------------------
##
##
## $SA12_low
##
## inclS PRI covS covU
## ----------------------------------------
## 1 Demandm 0.701 0.591 0.914 0.087
## 2 Laborm 0.761 0.656 0.858 0.021
## 3 Materialm 0.779 0.644 0.430 0.003
## 4 RelCommm 0.821 0.700 0.533 0.001
## ----------------------------------------
##
##
## $SA12_high
##
## inclS PRI covS covU
## ----------------------------------------
## 1 Demandm 0.445 0.268 0.926 0.041
## 2 Laborm 0.511 0.321 0.919 0.018
## 3 Materialm 0.601 0.361 0.528 0.003
## 4 RelCommm 0.644 0.395 0.666 0.000
## ----------------------------------------
##
##

## $SA13_low
##
## inclS PRI covS covU
## ----------------------------------------
## 1 Demandm 0.445 0.293 0.921 0.057
## 2 Laborm 0.499 0.335 0.894 0.013
## 3 Materialm 0.576 0.348 0.505 0.002
## 4 RelCommm 0.619 0.411 0.638 0.001
## ----------------------------------------
##
##
## $SA13_high
##
## inclS PRI covS covU
## ----------------------------------------
## 1 Demandm 0.264 0.115 0.945 0.028
## 2 Laborm 0.305 0.138 0.944 0.010
## 3 Materialm 0.396 0.138 0.600 0.003
## 4 RelCommm 0.442 0.198 0.787 0.001
## ----------------------------------------
##
##
## $SA14a_low
##
## inclS PRI covS covU
## ----------------------------------------
## 1 Demandm 0.700 0.581 0.913 0.080
## 2 Laborm 0.764 0.651 0.861 0.020
## 3 Materialm 0.814 0.674 0.449 0.002
## 4 RelCommm 0.837 0.716 0.544 0.000
## ----------------------------------------
##
##
## $SA14a_high
##
## inclS PRI covS covU
## ----------------------------------------
## 1 Demandm 0.428 0.240 0.933 0.039
## 2 Laborm 0.491 0.287 0.926 0.015
## 3 Materialm 0.582 0.303 0.537 0.001
## 4 RelCommm 0.639 0.367 0.693 0.000
## ----------------------------------------
##
##

## $SA14b_low
##
## inclS PRI covS covU
## ----------------------------------------
## 1 Demandm 0.831 0.776 0.902 0.101
## 2 Laborm 0.881 0.835 0.827 0.024
## 3 Materialm 0.907 0.854 0.417 0.002
## 4 RelCommm 0.941 0.905 0.509 0.001
## ----------------------------------------
##
##
## $SA14b_high
##
## inclS PRI covS covU
## ----------------------------------------
## 1 Demandm 0.614 0.483 0.930 0.071
## 2 Laborm 0.675 0.542 0.883 0.020
## 3 Materialm 0.751 0.582 0.481 0.001
## 4 RelCommm 0.802 0.661 0.604 0.001
## ----------------------------------------
##
##
## $SA14c_low
##
## inclS PRI covS covU
## ----------------------------------------
## 1 Demandm 0.860 0.824 0.885 0.111
## 2 Laborm 0.908 0.879 0.807 0.028
## 3 Materialm 0.919 0.880 0.400 0.003
## 4 RelCommm 0.951 0.927 0.487 0.001
## ----------------------------------------
##
##
## $SA14c_high
##
## inclS PRI covS covU
## ----------------------------------------
## 1 Demandm 0.694 0.602 0.914 0.084
## 2 Laborm 0.756 0.668 0.861 0.026
## 3 Materialm 0.791 0.670 0.441 0.002
## 4 RelCommm 0.857 0.774 0.562 0.001
## ----------------------------------------
##
##

## $SA15_low
##
## inclS PRI covS covU
## ----------------------------------------
## 1 Demandm 0.411 0.242 0.942 0.057
## 2 Laborm 0.458 0.273 0.909 0.013
## 3 Materialm 0.533 0.283 0.517 0.001
## 4 RelCommm 0.550 0.302 0.628 0.000
## ----------------------------------------
##
##
## $SA15_high
##
## inclS PRI covS covU
## ----------------------------------------
## 1 Demandm 0.216 0.063 0.949 0.015
## 2 Laborm 0.253 0.079 0.963 0.016
## 3 Materialm 0.357 0.090 0.664 0.000
## 4 RelCommm 0.370 0.101 0.811 0.000
## ----------------------------------------
##
##
## $SA16_low
##
## inclS PRI covS covU
## ----------------------------------------
## 1 Demandm 0.754 0.663 0.901 0.099
## 2 Laborm 0.809 0.725 0.836 0.025
## 3 Materialm 0.844 0.735 0.427 0.002
## 4 RelCommm 0.879 0.794 0.523 0.001
## ----------------------------------------
##
##
## $SA16_high
##
## inclS PRI covS covU
## ----------------------------------------
## 1 Demandm 0.496 0.308 0.927 0.052
## 2 Laborm 0.566 0.369 0.914 0.023
## 3 Materialm 0.634 0.368 0.501 0.002
## 4 RelCommm 0.705 0.465 0.656 0.002
## ----------------------------------------
##
##

## $SA17_low
##
## inclS PRI covS covU
## ----------------------------------------
## 1 Demandm 0.809 0.756 0.890 0.107
## 2 Laborm 0.853 0.805 0.811 0.024
## 3 Materialm 0.882 0.825 0.411 0.003
## 4 RelCommm 0.901 0.851 0.494 0.001
## ----------------------------------------
##
##
## $SA17_high
##
## inclS PRI covS covU
## ----------------------------------------
## 1 Demandm 0.617 0.497 0.906 0.070
## 2 Laborm 0.685 0.566 0.869 0.023
## 3 Materialm 0.748 0.598 0.464 0.003
## 4 RelCommm 0.781 0.641 0.571 0.001
## ----------------------------------------
##
##
## $SA18a_low
##
## inclS PRI covS covU
## ----------------------------------------
## 1 Demandm 0.590 0.443 0.909 0.081
## 2 Laborm 0.650 0.497 0.865 0.023
## 3 Materialm 0.705 0.500 0.459 0.001
## 4 RelCommm 0.730 0.540 0.560 0.000
## ----------------------------------------
##
##
## $SA18a_high
##
## inclS PRI covS covU
## ----------------------------------------
## 1 Demandm 0.343 0.164 0.915 0.045
## 2 Laborm 0.395 0.196 0.911 0.023
## 3 Materialm 0.466 0.173 0.526 0.000
## 4 RelCommm 0.522 0.237 0.692 0.000
## ----------------------------------------
##
##

## $SA18b_low
##
## inclS PRI covS covU
## ----------------------------------------
## 1 Demandm 0.495 0.324 0.921 0.077
## 2 Laborm 0.547 0.362 0.880 0.020
## 3 Materialm 0.603 0.346 0.475 0.001
## 4 RelCommm 0.640 0.407 0.594 0.000
## ----------------------------------------
##
##
## $SA18b_high
##
## inclS PRI covS covU
## ----------------------------------------
## 1 Demandm 0.266 0.094 0.935 0.025
## 2 Laborm 0.312 0.117 0.949 0.019
## 3 Materialm 0.386 0.110 0.575 0.000
## 4 RelCommm 0.434 0.152 0.760 0.000
## ----------------------------------------
##
##
## $SA19_low
##
## inclS PRI covS covU
## ----------------------------------------
## 1 Demandm 0.791 0.718 0.891 0.094
## 2 Laborm 0.850 0.787 0.828 0.025
## 3 Materialm 0.870 0.788 0.415 0.003
## 4 RelCommm 0.894 0.824 0.502 0.000
## ----------------------------------------
##
##
## $SA19_high
##
## inclS PRI covS covU
## ----------------------------------------
## 1 Demandm 0.553 0.389 0.929 0.060
## 2 Laborm 0.619 0.449 0.899 0.022
## 3 Materialm 0.686 0.476 0.487 0.001
## 4 RelCommm 0.727 0.518 0.607 0.000
## ----------------------------------------
##
##

## $SA20_low
##
## inclS PRI covS covU
## ----------------------------------------
## 1 Demandm 0.628 0.482 0.920 0.065
## 2 Laborm 0.702 0.557 0.888 0.023
## 3 Materialm 0.752 0.586 0.466 0.002
## 4 RelCommm 0.784 0.614 0.572 0.000
## ----------------------------------------
##
##
## $SA20_high
##
## inclS PRI covS covU
## ----------------------------------------
## 1 Demandm 0.355 0.144 0.952 0.019
## 2 Laborm 0.415 0.181 0.963 0.012
## 3 Materialm 0.547 0.235 0.621 0.001
## 4 RelCommm 0.570 0.248 0.761 0.000
## ----------------------------------------
##
##
## $SA21_low
##
## inclS PRI covS covU
## ----------------------------------------
## 1 Demandm 0.807 0.745 0.893 0.105
## 2 Laborm 0.856 0.802 0.819 0.025
## 3 Materialm 0.884 0.817 0.414 0.002
## 4 RelCommm 0.901 0.843 0.496 0.001
## ----------------------------------------
##
##
## $SA21_high
##
## inclS PRI covS covU
## ----------------------------------------
## 1 Demandm 0.589 0.443 0.922 0.067
## 2 Laborm 0.656 0.504 0.887 0.022
## 3 Materialm 0.725 0.547 0.480 0.002
## 4 RelCommm 0.769 0.593 0.599 0.001
## ----------------------------------------
##
##

## $SA22_low
##
## inclS PRI covS covU
## ----------------------------------------
## 1 Demandm 0.862 0.828 0.877 0.118
## 2 Laborm 0.901 0.873 0.793 0.028
## 3 Materialm 0.906 0.869 0.390 0.003
## 4 RelCommm 0.928 0.896 0.471 0.001
## ----------------------------------------
##
##
## $SA22_high
##
## inclS PRI covS covU
## ----------------------------------------
## 1 Demandm 0.706 0.631 0.899 0.095
## 2 Laborm 0.761 0.688 0.838 0.027
## 3 Materialm 0.825 0.744 0.444 0.002
## 4 RelCommm 0.825 0.739 0.523 0.001
## ----------------------------------------
##
##
## $SA23_low
##
## inclS PRI covS covU
## ----------------------------------------
## 1 Demandm 0.656 0.509 0.937 0.079
## 2 Laborm 0.715 0.571 0.884 0.016
## 3 Materialm 0.773 0.604 0.468 0.002
## 4 RelCommm 0.802 0.640 0.571 0.000
## ----------------------------------------
##
##
## $SA23_high
##
## inclS PRI covS covU
## ----------------------------------------
## 1 Demandm 0.359 0.138 0.957 0.023
## 2 Laborm 0.419 0.173 0.965 0.012
## 3 Materialm 0.533 0.205 0.601 0.002
## 4 RelCommm 0.571 0.239 0.758 0.000
## ----------------------------------------
##
##

## $SA24_low
##
## inclS PRI covS covU
## ----------------------------------------
## 1 Demandm 0.573 0.408 0.927 0.082
## 2 Laborm 0.627 0.452 0.876 0.021
## 3 Materialm 0.709 0.501 0.485 0.001
## 4 RelCommm 0.715 0.500 0.575 0.000
## ----------------------------------------
##
##
## $SA24_high
##
## inclS PRI covS covU
## ----------------------------------------
## 1 Demandm 0.306 0.099 0.963 0.036
## 2 Laborm 0.350 0.114 0.953 0.009
## 3 Materialm 0.472 0.141 0.629 0.000
## 4 RelCommm 0.487 0.155 0.764 0.000
## ----------------------------------------
##
##
## $SA25_low
##
## inclS PRI covS covU
## ----------------------------------------
## 1 Demandm 0.850 0.806 0.889 0.114
## 2 Laborm 0.890 0.852 0.805 0.026
## 3 Materialm 0.901 0.846 0.399 0.002
## 4 RelCommm 0.930 0.891 0.484 0.001
## ----------------------------------------
##
##
## $SA25_high
##
## inclS PRI covS covU
## ----------------------------------------
## 1 Demandm 0.653 0.524 0.919 0.071
## 2 Laborm 0.722 0.597 0.878 0.024
## 3 Materialm 0.765 0.602 0.456 0.001
## 4 RelCommm 0.823 0.693 0.577 0.001
## ----------------------------------------

#### Performing QCA: Analysis of Sufficiency and Minimization

#### FOR n.cut (minimum number of cases with membership > 0.5 for an outcome) -> n3:n.cut=3

# Complex solutions: Positive output
# Result: Final QCA COMPLEX Solutions for Configurations of Reasons for MNC triggering single Outcomes of MNC (n.cut=3)

## $SA1_low
##
## M1: Demandm*Laborm + Demandm*~RelCommm + Laborm*~Materialm*~RelCommm -> SA1_low
##
## inclS PRI covS covU
## ----------------------------------------------------------
## 1 Demandm*Laborm 0.900 0.862 0.775 0.236
## 2 Demandm*~RelCommm 0.881 0.822 0.625 0.086
## 3 Laborm*~Materialm*~RelCommm 0.928 0.875 0.484 0.020
## ----------------------------------------------------------
## M1 0.863 0.818 0.882
##
##
## $SA1_high
##
## M1: Demandm*Laborm*RelCommm + Demandm*Materialm*~RelCommm +
## ~Demandm*Laborm*~Materialm*~RelCommm -> SA1_high
##
## inclS PRI covS covU
## -------------------------------------------------------------------
## 1 Demandm*Laborm*RelCommm 0.803 0.626 0.560 0.249
## 2 Demandm*Materialm*~RelCommm 0.828 0.582 0.332 0.060
## 3 ~Demandm*Laborm*~Materialm*~RelCommm 0.826 0.448 0.240 0.045
## -------------------------------------------------------------------
## M1 0.759 0.579 0.674
##
##
## $SA2_low
##
## M1: Demandm*Materialm*~RelCommm + Demandm*Laborm*~Materialm*RelCommm -> SA2_low
##
## inclS PRI covS covU
## -----------------------------------------------------------------
## 1 Demandm*Materialm*~RelCommm 0.788 0.516 0.317 0.110
## 2 Demandm*Laborm*~Materialm*RelCommm 0.813 0.590 0.407 0.200
## -----------------------------------------------------------------
## M1 0.780 0.578 0.517
##
##
## $SA2_high
## [1] "Error: None of the values in OUT is explained. Please check the truth table."
##

## $SA3_low
##
## M1: Demandm*Laborm*Materialm + Demandm*Materialm*~RelCommm -> SA3_low
##
## inclS PRI covS covU
## ----------------------------------------------------------
## 1 Demandm*Laborm*Materialm 0.797 0.644 0.456 0.133
## 2 Demandm*Materialm*~RelCommm 0.834 0.624 0.334 0.010
## ----------------------------------------------------------
## M1 0.791 0.640 0.466
##
##
## $SA3_high
## [1] "Error: None of the values in OUT is explained. Please check the truth table."
##
## $SA4_low
## [1] "Error: None of the values in OUT is explained. Please check the truth table."
##
## $SA4_high
## [1] "Error: None of the values in OUT is explained. Please check the truth table."
##
## $SA5_low
##
## M1: Demandm*Laborm + Demandm*~RelCommm + Laborm*~Materialm*~RelCommm -> SA5_low
##
## inclS PRI covS covU
## ----------------------------------------------------------
## 1 Demandm*Laborm 0.781 0.666 0.819 0.229
## 2 Demandm*~RelCommm 0.771 0.620 0.667 0.077
## 3 Laborm*~Materialm*~RelCommm 0.822 0.648 0.522 0.020
## ----------------------------------------------------------
## M1 0.735 0.619 0.916
##
##
## $SA5_high
##
## M1: Demandm*~Laborm*Materialm*~RelCommm -> SA5_high
##
## inclS PRI covS covU
## ------------------------------------------------------------------
## 1 Demandm*~Laborm*Materialm*~RelCommm 0.810 0.181 0.286 -
## ------------------------------------------------------------------
## M1 0.810 0.181 0.286
##
##

## $SA6_low
##
## M1: Demandm*Laborm + Demandm*Materialm*~RelCommm -> SA6_low
##
## inclS PRI covS covU
## ----------------------------------------------------------
## 1 Demandm*Laborm 0.748 0.629 0.826 0.523
## 2 Demandm*Materialm*~RelCommm 0.822 0.610 0.307 0.004
## ----------------------------------------------------------
## M1 0.741 0.620 0.830
##
##
## $SA6_high
## [1] "Error: None of the values in OUT is explained. Please check the truth table."
##
## $SA7_low
##
## M1: Demandm*Laborm*RelCommm + Demandm*Materialm*~RelCommm -> SA7_low
##
## inclS PRI covS covU
## ----------------------------------------------------------
## 1 Demandm*Laborm*RelCommm 0.845 0.727 0.537 0.304
## 2 Demandm*Materialm*~RelCommm 0.850 0.647 0.311 0.078
## ----------------------------------------------------------
## M1 0.828 0.709 0.615
##
##
## $SA7_high
## [1] "Error: None of the values in OUT is explained. Please check the truth table."
##
## $SA8_low
##
## M1: Demandm*Laborm + Demandm*~RelCommm + Laborm*~Materialm*~RelCommm -> SA8_low
##
## inclS PRI covS covU
## ----------------------------------------------------------
## 1 Demandm*Laborm 0.856 0.800 0.766 0.231
## 2 Demandm*~RelCommm 0.847 0.771 0.625 0.090
## 3 Laborm*~Materialm*~RelCommm 0.885 0.800 0.479 0.022
## ----------------------------------------------------------
## M1 0.826 0.768 0.878
##
##

## $SA8_high
##
## M1: Demandm*Laborm*RelCommm + Demandm*Materialm*~RelCommm +
## ~Demandm*Laborm*~Materialm*~RelCommm -> SA8_high
##
## inclS PRI covS covU
## -------------------------------------------------------------------
## 1 Demandm*Laborm*RelCommm 0.772 0.581 0.571 0.252
## 2 Demandm*Materialm*~RelCommm 0.817 0.572 0.347 0.063
## 3 ~Demandm*Laborm*~Materialm*~RelCommm 0.804 0.420 0.248 0.045
## -------------------------------------------------------------------
## M1 0.731 0.549 0.690
##
##
## $SA9a_low
##
## M1: Demandm*Laborm + Demandm*~RelCommm + ~Materialm*~RelCommm -> SA9a_low
##
## inclS PRI covS covU
## ---------------------------------------------------
## 1 Demandm*Laborm 0.934 0.914 0.733 0.229
## 2 Demandm*~RelCommm 0.926 0.897 0.599 0.014
## 3 ~Materialm*~RelCommm 0.848 0.792 0.581 0.070
## ---------------------------------------------------
## M1 0.864 0.833 0.898
##
##
## $SA9a_high
##
## M1: Demandm*Laborm + Demandm*~RelCommm + Laborm*~Materialm*~RelCommm
## -> SA9a_high
##
## inclS PRI covS covU
## ----------------------------------------------------------
## 1 Demandm*Laborm 0.822 0.751 0.797 0.239
## 2 Demandm*~RelCommm 0.793 0.684 0.633 0.076
## 3 Laborm*~Materialm*~RelCommm 0.850 0.732 0.499 0.020
## ----------------------------------------------------------
## M1 0.776 0.699 0.892
##
##
## $SA9b_low
##
## M1: Demandm*Laborm + Demandm*~RelCommm + ~Materialm*~RelCommm -> SA9b_low
##
## inclS PRI covS covU
## ---------------------------------------------------
## 1 Demandm*Laborm 0.931 0.912 0.732 0.229
## 2 Demandm*~RelCommm 0.930 0.904 0.602 0.015
## 3 ~Materialm*~RelCommm 0.851 0.799 0.583 0.068
## ---------------------------------------------------
## M1 0.865 0.834 0.899
##
##

## $SA9b_high
##
## M1: Demandm*Laborm + Demandm*~RelCommm + Laborm*~Materialm*~RelCommm
## -> SA9b_high
##
## inclS PRI covS covU
## ----------------------------------------------------------
## 1 Demandm*Laborm 0.819 0.743 0.793 0.229
## 2 Demandm*~RelCommm 0.805 0.700 0.642 0.078
## 3 Laborm*~Materialm*~RelCommm 0.866 0.757 0.507 0.020
## ----------------------------------------------------------
## M1 0.775 0.695 0.891
##
##
## $SA10a_low
##
## M1: Demandm*Laborm + Demandm*~RelCommm + ~Materialm*~RelCommm -> SA10a_low
##
## inclS PRI covS covU
## ---------------------------------------------------
## 1 Demandm*Laborm 0.934 0.914 0.735 0.230
## 2 Demandm*~RelCommm 0.927 0.899 0.602 0.013
## 3 ~Materialm*~RelCommm 0.852 0.798 0.585 0.072
## ---------------------------------------------------
## M1 0.867 0.835 0.903
##
##
## $SA10a_high
##
## M1: Demandm*Laborm + Demandm*~RelCommm + Laborm*~Materialm*~RelCommm
## -> SA10a_high
##
## inclS PRI covS covU
## ----------------------------------------------------------
## 1 Demandm*Laborm 0.811 0.734 0.796 0.230
## 2 Demandm*~RelCommm 0.792 0.682 0.641 0.074
## 3 Laborm*~Materialm*~RelCommm 0.852 0.737 0.506 0.019
## ----------------------------------------------------------
## M1 0.763 0.681 0.889
##
##
## $SA10b_low
##
## M1: Demandm*Laborm + Demandm*~RelCommm + ~Materialm*~RelCommm -> SA10b_low
##
## inclS PRI covS covU
## ---------------------------------------------------
## 1 Demandm*Laborm 0.961 0.952 0.719 0.232
## 2 Demandm*~RelCommm 0.948 0.932 0.584 0.015
## 3 ~Materialm*~RelCommm 0.877 0.841 0.572 0.074
## ---------------------------------------------------
## M1 0.900 0.881 0.890
##
##

## $SA10b_high
##
## M1: Demandm*Laborm + Demandm*~RelCommm + Laborm*~Materialm*~RelCommm
## -> SA10b_high
##
## inclS PRI covS covU
## ----------------------------------------------------------
## 1 Demandm*Laborm 0.885 0.845 0.772 0.239
## 2 Demandm*~RelCommm 0.850 0.783 0.612 0.078
## 3 Laborm*~Materialm*~RelCommm 0.913 0.856 0.482 0.023
## ----------------------------------------------------------
## M1 0.843 0.796 0.873
##
##
## $SA11_low
##
## M1: Demandm*Materialm*~RelCommm + Demandm*Laborm*~Materialm*RelCommm
## -> SA11_low
##
## inclS PRI covS covU
## -----------------------------------------------------------------
## 1 Demandm*Materialm*~RelCommm 0.797 0.482 0.333 0.117
## 2 Demandm*Laborm*~Materialm*RelCommm 0.806 0.524 0.418 0.203
## -----------------------------------------------------------------
## M1 0.778 0.528 0.536
##
##
## $SA11_high
## [1] "Error: None of the values in OUT is explained. Please check the truth table."
##
## $SA12_low
##
## M1: Demandm*Laborm + Demandm*~RelCommm + Laborm*~Materialm*~RelCommm
## -> SA12_low
##
## inclS PRI covS covU
## ----------------------------------------------------------
## 1 Demandm*Laborm 0.778 0.673 0.817 0.232
## 2 Demandm*~RelCommm 0.761 0.614 0.659 0.074
## 3 Laborm*~Materialm*~RelCommm 0.817 0.650 0.519 0.019
## ----------------------------------------------------------
## M1 0.730 0.623 0.910
##
##
## $SA12_high
## [1] "Error: None of the values in OUT is explained. Please check the truth table."
##
## $SA13_low
## [1] "Error: None of the values in OUT is explained. Please check the truth table."
##
## $SA13_high
## [1] "Error: None of the values in OUT is explained. Please check the truth table."
##
## $SA14a_low
##
## M1: Demandm*Laborm + Demandm*Materialm*~RelCommm + Laborm*~Materialm*~RelCommm
## -> SA14a_low
##
## inclS PRI covS covU
## ----------------------------------------------------------
## 1 Demandm*Laborm 0.783 0.671 0.822 0.245
## 2 Demandm*Materialm*~RelCommm 0.864 0.665 0.306 0.005
## 3 Laborm*~Materialm*~RelCommm 0.810 0.629 0.515 0.018
## ----------------------------------------------------------
## M1 0.769 0.653 0.845
##
##
## $SA14a_high
## [1] "Error: None of the values in OUT is explained. Please check the truth table."
##
## $SA14b_low
##
## M1: Demandm*Laborm + Demandm*~RelCommm + Laborm*~Materialm*~RelCommm
## -> SA14b_low
##
## inclS PRI covS covU
## ----------------------------------------------------------
## 1 Demandm*Laborm 0.896 0.853 0.784 0.241
## 2 Demandm*~RelCommm 0.870 0.798 0.627 0.085
## 3 Laborm*~Materialm*~RelCommm 0.911 0.840 0.482 0.019
## ----------------------------------------------------------
## M1 0.854 0.803 0.887
##
##
## $SA14b_high
##
## M1: Demandm*Laborm*RelCommm + Demandm*Materialm*~RelCommm -> SA14b_high
##
## inclS PRI covS covU
## ----------------------------------------------------------
## 1 Demandm*Laborm*RelCommm 0.811 0.667 0.580 0.315
## 2 Demandm*Materialm*~RelCommm 0.828 0.598 0.341 0.075
## ----------------------------------------------------------
## M1 0.784 0.638 0.656
##
##
## $SA14c_low
##
## M1: Demandm*Laborm + Demandm*~RelCommm + Laborm*~Materialm*~RelCommm
## -> SA14c_low
##
## inclS PRI covS covU
## ----------------------------------------------------------
## 1 Demandm*Laborm 0.916 0.889 0.759 0.238
## 2 Demandm*~RelCommm 0.890 0.843 0.607 0.087
## 3 Laborm*~Materialm*~RelCommm 0.930 0.887 0.466 0.020
## ----------------------------------------------------------
## M1 0.881 0.848 0.866
##
##
## $SA14c_high
##
## M1: Demandm*Laborm + Demandm*Materialm*~RelCommm + Laborm*~Materialm*~RelCommm
## -> SA14c_high
##
## inclS PRI covS covU
## ----------------------------------------------------------
## 1 Demandm*Laborm 0.770 0.680 0.816 0.258
## 2 Demandm*Materialm*~RelCommm 0.846 0.670 0.303 0.008
## 3 Laborm*~Materialm*~RelCommm 0.798 0.654 0.512 0.017
## ----------------------------------------------------------
## M1 0.758 0.668 0.841
##
##
## $SA15_low
## [1] "Error: None of the values in OUT is explained. Please check the truth table."
##
## $SA15_high
## [1] "Error: None of the values in OUT is explained. Please check the truth table."
##
## $SA16_low
##
## M1: Demandm*Laborm + Demandm*~RelCommm + Laborm*~Materialm*~RelCommm
## -> SA16_low
##
## inclS PRI covS covU
## ----------------------------------------------------------
## 1 Demandm*Laborm 0.822 0.737 0.791 0.232
## 2 Demandm*~RelCommm 0.815 0.700 0.646 0.087
## 3 Laborm*~Materialm*~RelCommm 0.854 0.725 0.497 0.018
## ----------------------------------------------------------
## M1 0.784 0.697 0.895
##
##
## $SA16_high
##
## M1: Demandm*~Laborm*Materialm*~RelCommm -> SA16_high
##
## inclS PRI covS covU
## ------------------------------------------------------------------
## 1 Demandm*~Laborm*Materialm*~RelCommm 0.816 0.238 0.245 -
## ------------------------------------------------------------------
## M1 0.816 0.238 0.245
##
##

## $SA17_low
##
## M1: Demandm*Laborm + Demandm*~RelCommm + Laborm*~Materialm*~RelCommm
## -> SA17_low
##
## inclS PRI covS covU
## ----------------------------------------------------------
## 1 Demandm*Laborm 0.867 0.821 0.768 0.237
## 2 Demandm*~RelCommm 0.850 0.782 0.621 0.090
## 3 Laborm*~Materialm*~RelCommm 0.881 0.803 0.472 0.018
## ----------------------------------------------------------
## M1 0.833 0.783 0.875
##
##
## $SA17_high
##
## M1: Demandm*Laborm*RelCommm + Demandm*Materialm*~RelCommm -> SA17_high
##
## inclS PRI covS covU
## ----------------------------------------------------------
## 1 Demandm*Laborm*RelCommm 0.788 0.643 0.547 0.288
## 2 Demandm*Materialm*~RelCommm 0.830 0.641 0.332 0.072
## ----------------------------------------------------------
## M1 0.764 0.626 0.620
##
##
## $SA18a_low
##
## M1: Demandm*~Laborm*Materialm*~RelCommm -> SA18a_low
##
## inclS PRI covS covU
## ------------------------------------------------------------------
## 1 Demandm*~Laborm*Materialm*~RelCommm 0.809 0.300 0.201 -
## ------------------------------------------------------------------
## M1 0.809 0.300 0.201
##
##
## $SA18a_high
## [1] "Error: None of the values in OUT is explained. Please check the truth table."
##
## $SA18b_low
## [1] "Error: None of the values in OUT is explained. Please check the truth table."
##
## $SA18b_high
## [1] "Error: None of the values in OUT is explained. Please check the truth table."
##

## $SA19_low
##
## M1: Demandm*Laborm + Demandm*~RelCommm + Laborm*~Materialm*~RelCommm
## -> SA19_low
##
## inclS PRI covS covU
## ----------------------------------------------------------
## 1 Demandm*Laborm 0.864 0.803 0.784 0.229
## 2 Demandm*~RelCommm 0.852 0.767 0.637 0.082
## 3 Laborm*~Materialm*~RelCommm 0.898 0.813 0.493 0.020
## ----------------------------------------------------------
## M1 0.823 0.755 0.886
##
##
## $SA19_high
##
## M1: Demandm*Materialm*~RelCommm -> SA19_high
##
## inclS PRI covS covU
## ----------------------------------------------------------
## 1 Demandm*Materialm*~RelCommm 0.779 0.474 0.356 -
## ----------------------------------------------------------
## M1 0.779 0.474 0.356
##
##
## $SA20_low
##
## M1: Demandm*Laborm*Materialm + Demandm*Laborm*RelCommm -> SA20_low
##
## inclS PRI covS covU
## -------------------------------------------------------
## 1 Demandm*Laborm*Materialm 0.781 0.617 0.445 0.080
## 2 Demandm*Laborm*RelCommm 0.797 0.627 0.552 0.187
## -------------------------------------------------------
## M1 0.769 0.610 0.633
##
##
## $SA20_high
## [1] "Error: None of the values in OUT is explained. Please check the truth table."
##
## $SA21_low
##
## M1: Demandm*Laborm + Demandm*~RelCommm + Laborm*~Materialm*~RelCommm
## -> SA21_low
##
## inclS PRI covS covU
## ----------------------------------------------------------
## 1 Demandm*Laborm 0.869 0.817 0.774 0.232
## 2 Demandm*~RelCommm 0.860 0.787 0.631 0.089
## 3 Laborm*~Materialm*~RelCommm 0.892 0.812 0.481 0.018
## ----------------------------------------------------------
## M1 0.833 0.775 0.881
##
##

## $SA21_high
##
## M1: Demandm*Laborm*RelCommm + Demandm*Materialm*~RelCommm +
## ~Demandm*Laborm*~Materialm*~RelCommm -> SA21_high
##
## inclS PRI covS covU
## -------------------------------------------------------------------
## 1 Demandm*Laborm*RelCommm 0.778 0.597 0.575 0.253
## 2 Demandm*Materialm*~RelCommm 0.801 0.553 0.341 0.056
## 3 ~Demandm*Laborm*~Materialm*~RelCommm 0.803 0.387 0.247 0.039
## -------------------------------------------------------------------
## M1 0.722 0.535 0.681
##
##
## $SA22_low
##
## M1: Demandm*Laborm + Demandm*~RelCommm + Laborm*~Materialm*~RelCommm
## -> SA22_low
##
## inclS PRI covS covU
## ----------------------------------------------------------
## 1 Demandm*Laborm 0.909 0.881 0.745 0.228
## 2 Demandm*~RelCommm 0.902 0.863 0.609 0.092
## 3 Laborm*~Materialm*~RelCommm 0.931 0.892 0.462 0.019
## ----------------------------------------------------------
## M1 0.880 0.848 0.856
##
##
## $SA22_high
##
## M1: Demandm*Laborm + Demandm*Materialm*~RelCommm + Laborm*~Materialm*~RelCommm
## -> SA22_high
##
## inclS PRI covS covU
## ----------------------------------------------------------
## 1 Demandm*Laborm 0.770 0.695 0.790 0.239
## 2 Demandm*Materialm*~RelCommm 0.893 0.802 0.309 0.008
## 3 Laborm*~Materialm*~RelCommm 0.794 0.669 0.493 0.016
## ----------------------------------------------------------
## M1 0.759 0.682 0.814
##
##
## $SA23_low
##
## M1: Demandm*Laborm*RelCommm + Demandm*Materialm*~RelCommm -> SA23_low
##
## inclS PRI covS covU
## ----------------------------------------------------------
## 1 Demandm*Laborm*RelCommm 0.811 0.646 0.548 0.305
## 2 Demandm*Materialm*~RelCommm 0.819 0.550 0.318 0.075
## ----------------------------------------------------------
## M1 0.789 0.627 0.623
##
##
## $SA23_high
## [1] "Error: None of the values in OUT is explained. Please check the truth table."
##
## $SA24_low
##
## M1: Demandm*~Laborm*Materialm*~RelCommm -> SA24_low
##
## inclS PRI covS covU
## ------------------------------------------------------------------
## 1 Demandm*~Laborm*Materialm*~RelCommm 0.802 0.212 0.209 -
## ------------------------------------------------------------------
## M1 0.802 0.212 0.209
##
##
## $SA24_high
## [1] "Error: None of the values in OUT is explained. Please check the truth table."
##
## $SA25_low
##
## M1: Demandm*Laborm + Demandm*~RelCommm + Laborm*~Materialm*~RelCommm
## -> SA25_low
##
## inclS PRI covS covU
## ----------------------------------------------------------
## 1 Demandm*Laborm 0.903 0.867 0.760 0.233
## 2 Demandm*~RelCommm 0.899 0.851 0.624 0.097
## 3 Laborm*~Materialm*~RelCommm 0.931 0.883 0.474 0.020
## ----------------------------------------------------------
## M1 0.878 0.839 0.877
##
##
## $SA25_high
##
## M1: Demandm*Laborm + Demandm*Materialm*~RelCommm + Laborm*~Materialm*~RelCommm
## -> SA25_high
##
## inclS PRI covS covU
## ----------------------------------------------------------
## 1 Demandm*Laborm 0.737 0.610 0.835 0.245
## 2 Demandm*Materialm*~RelCommm 0.835 0.603 0.320 0.007
## 3 Laborm*~Materialm*~RelCommm 0.789 0.592 0.541 0.019
## ----------------------------------------------------------
## M1 0.726 0.598 0.862

# Parsimonious solutions: Positive output including the remainders
# Main Results: Calling results
results_TTCondsReasons_p3 # Result: Final QCA PARSIMONIOUS Solutions for Configurations of Reasons for MNC triggering single Outcomes of MNC (n.cut=3)

## $SA1_low
##
## M1: Demandm + Laborm -> SA1_low
##
## inclS PRI covS covU
## --------------------------------------
## 1 Demandm 0.834 0.784 0.892 0.117
## 2 Laborm 0.887 0.846 0.820 0.045
## --------------------------------------
## M1 0.826 0.777 0.937
##
##
## $SA1_high
##
## M1: Materialm + RelCommm + ~Demandm*Laborm -> SA1_high
##
## inclS PRI covS covU
## ----------------------------------------------
## 1 Materialm 0.734 0.535 0.458 0.075
## 2 RelCommm 0.795 0.624 0.583 0.153
## 3 ~Demandm*Laborm 0.823 0.509 0.266 0.042
## ----------------------------------------------
## M1 0.734 0.563 0.712
##
##
## $SA2_low
##
## M1: Materialm*~RelCommm + ~Materialm*RelCommm -> SA2_low
##
## inclS PRI covS covU
## --------------------------------------------------
## 1 Materialm*~RelCommm 0.778 0.502 0.320 0.111
## 2 ~Materialm*RelCommm 0.806 0.589 0.421 0.212
## --------------------------------------------------
## M1 0.770 0.569 0.533
##
##
## $SA2_high
## [1] "Error: None of the values in OUT is explained. Please check the truth table."
##
## $SA3_low
##
## M1: Materialm -> SA3_low
##
## inclS PRI covS covU
## ----------------------------------------
## 1 Materialm 0.769 0.614 0.478 -
## ----------------------------------------
## M1 0.769 0.614 0.478
##
##
## $SA3_high
## [1] "Error: None of the values in OUT is explained. Please check the truth table."
##
## $SA4_low
## [1] "Error: None of the values in OUT is explained. Please check the truth table."
##
## $SA4_high
## [1] "Error: None of the values in OUT is explained. Please check the truth table."
##
## $SA5_low
##
## M1: Demandm + Laborm -> SA5_low
##
## inclS PRI covS covU
## --------------------------------------
## 1 Demandm 0.703 0.584 0.917 0.097
## 2 Laborm 0.765 0.651 0.862 0.043
## --------------------------------------
## M1 0.694 0.579 0.959
##
##
## $SA5_high
##
## M1: ~Laborm*Materialm -> SA5_high
##
## inclS PRI covS covU
## ------------------------------------------------
## 1 ~Laborm*Materialm 0.786 0.170 0.291 -
## ------------------------------------------------
## M1 0.786 0.170 0.291
##
##
## $SA6_low
##
## M1: Materialm + Demandm*Laborm -> SA6_low
##
## inclS PRI covS covU
## ---------------------------------------------
## 1 Materialm 0.775 0.636 0.450 0.018
## 2 Demandm*Laborm 0.748 0.629 0.826 0.394
## ---------------------------------------------
## M1 0.734 0.614 0.844
##
##
## $SA6_high
## [1] "Error: None of the values in OUT is explained. Please check the truth table."
##

## $SA7_low
##
## M1: Materialm + RelCommm -> SA7_low
##
## inclS PRI covS covU
## ----------------------------------------
## 1 Materialm 0.792 0.648 0.451 0.097
## 2 RelCommm 0.833 0.713 0.558 0.204
## ----------------------------------------
## M1 0.795 0.673 0.655
##
##
## $SA7_high
## [1] "Error: None of the values in OUT is explained. Please check the truth table."
##
## $SA8_low
##
## M1: Demandm + Laborm -> SA8_low
##
## inclS PRI covS covU
## --------------------------------------
## 1 Demandm 0.795 0.733 0.884 0.118
## 2 Laborm 0.846 0.791 0.813 0.047
## --------------------------------------
## M1 0.791 0.730 0.931
##
##
## $SA8_high
##
## M1: Materialm + RelCommm + ~Demandm*Laborm -> SA8_high
##
## inclS PRI covS covU
## ----------------------------------------------
## 1 Materialm 0.728 0.540 0.482 0.080
## 2 RelCommm 0.765 0.580 0.596 0.148
## 3 ~Demandm*Laborm 0.799 0.485 0.274 0.041
## ----------------------------------------------
## M1 0.708 0.534 0.728
##
##
## $SA9a_low
## [1] "Error: All truth table configurations are used, all conditions are minimized. Please check the truth table."
##
## $SA9a_high
##
## M1: Demandm + Laborm -> SA9a_high
##
## inclS PRI covS covU
## --------------------------------------
## 1 Demandm 0.751 0.673 0.904 0.108
## 2 Laborm 0.812 0.742 0.845 0.049
## --------------------------------------
## M1 0.747 0.672 0.953
##
##
## $SA9b_low
## [1] "Error: All truth table configurations are used, all conditions are minimized. Please check the truth table."
##
## $SA9b_high
##
## M1: Demandm + Laborm -> SA9b_high
##
## inclS PRI covS covU
## --------------------------------------
## 1 Demandm 0.749 0.667 0.900 0.108
## 2 Laborm 0.808 0.732 0.839 0.047
## --------------------------------------
## M1 0.744 0.664 0.947
##
##
## $SA10a_low
## [1] "Error: All truth table configurations are used, all conditions are minimized. Please check the truth table."
##
## $SA10a_high
##
## M1: Demandm + Laborm -> SA10a_high
##
## inclS PRI covS covU
## --------------------------------------
## 1 Demandm 0.738 0.655 0.900 0.104
## 2 Laborm 0.798 0.721 0.841 0.045
## --------------------------------------
## M1 0.731 0.650 0.945
##
##
## $SA10b_low
## [1] "Error: All truth table configurations are used, all conditions are minimized. Please check the truth table."
##
## $SA10b_high
##
## M1: Demandm + Laborm -> SA10b_high
##
## inclS PRI covS covU
## --------------------------------------
## 1 Demandm 0.819 0.769 0.888 0.115
## 2 Laborm 0.877 0.837 0.822 0.049
## --------------------------------------
## M1 0.816 0.768 0.937
##
##

## $SA11_low
##
## M1: Materialm*~RelCommm + ~Materialm*RelCommm -> SA11_low
##
## inclS PRI covS covU
## --------------------------------------------------
## 1 Materialm*~RelCommm 0.796 0.482 0.341 0.121
## 2 ~Materialm*RelCommm 0.797 0.523 0.433 0.213
## --------------------------------------------------
## M1 0.771 0.525 0.554
##
##
## $SA11_high
## [1] "Error: None of the values in OUT is explained. Please check the truth table."
##
## $SA12_low
##
## M1: Demandm + Laborm -> SA12_low
##
## inclS PRI covS covU
## --------------------------------------
## 1 Demandm 0.701 0.591 0.914 0.097
## 2 Laborm 0.761 0.656 0.858 0.041
## --------------------------------------
## M1 0.691 0.584 0.955
##
##
## $SA12_high
## [1] "Error: None of the values in OUT is explained. Please check the truth table."
##
## $SA13_low
## [1] "Error: None of the values in OUT is explained. Please check the truth table."
##
## $SA13_high
## [1] "Error: None of the values in OUT is explained. Please check the truth table."
##
## $SA14a_low
##
## M1: Laborm + Materialm -> SA14a_low
##
## inclS PRI covS covU
## ----------------------------------------
## 1 Laborm 0.764 0.651 0.861 0.423
## 2 Materialm 0.814 0.674 0.449 0.011
## ----------------------------------------
## M1 0.753 0.638 0.873
##
##
## $SA14a_high
## [1] "Error: None of the values in OUT is explained. Please check the truth table."
##

## $SA14b_low
##
## M1: Demandm + Laborm -> SA14b_low
##
## inclS PRI covS covU
## --------------------------------------
## 1 Demandm 0.831 0.776 0.902 0.119
## 2 Laborm 0.881 0.835 0.827 0.044
## --------------------------------------
## M1 0.822 0.768 0.946
##
##
## $SA14b_high
##
## M1: Materialm + RelCommm -> SA14b_high
##
## inclS PRI covS covU
## ----------------------------------------
## 1 Materialm 0.751 0.582 0.481 0.093
## 2 RelCommm 0.802 0.661 0.604 0.216
## ----------------------------------------
## M1 0.752 0.608 0.697
##
##
## $SA14c_low
##
## M1: Demandm + Laborm -> SA14c_low
##
## inclS PRI covS covU
## --------------------------------------
## 1 Demandm 0.860 0.824 0.885 0.126
## 2 Laborm 0.908 0.879 0.807 0.048
## --------------------------------------
## M1 0.856 0.821 0.933
##
##
## $SA14c_high
##
## M1: Laborm + Materialm -> SA14c_high
##
## inclS PRI covS covU
## ----------------------------------------
## 1 Laborm 0.756 0.668 0.861 0.435
## 2 Materialm 0.791 0.670 0.441 0.015
## ----------------------------------------
## M1 0.748 0.661 0.876
##
##
## $SA15_low
## [1] "Error: None of the values in OUT is explained. Please check the truth table."
##
## $SA15_high
## [1] "Error: None of the values in OUT is explained. Please check the truth table."
##

## $SA16_low
##
## M1: Demandm + Laborm -> SA16_low
##
## inclS PRI covS covU
## --------------------------------------
## 1 Demandm 0.754 0.663 0.901 0.110
## 2 Laborm 0.809 0.725 0.836 0.045
## --------------------------------------
## M1 0.747 0.659 0.946
##
##
## $SA16_high
##
## M1: ~Laborm*Materialm -> SA16_high
##
## inclS PRI covS covU
## ------------------------------------------------
## 1 ~Laborm*Materialm 0.796 0.239 0.251 -
## ------------------------------------------------
## M1 0.796 0.239 0.251
##
##
## $SA17_low
##
## M1: Demandm + Laborm -> SA17_low
##
## inclS PRI covS covU
## --------------------------------------
## 1 Demandm 0.809 0.756 0.890 0.122
## 2 Laborm 0.853 0.805 0.811 0.043
## --------------------------------------
## M1 0.801 0.748 0.933
##
##
## $SA17_high
##
## M1: Materialm + RelCommm -> SA17_high
##
## inclS PRI covS covU
## ----------------------------------------
## 1 Materialm 0.748 0.598 0.464 0.093
## 2 RelCommm 0.781 0.641 0.571 0.199
## ----------------------------------------
## M1 0.738 0.606 0.664
##
##
## $SA18a_low
##
## M1: ~Laborm*Materialm -> SA18a_low
##
## inclS PRI covS covU
## ------------------------------------------------
## 1 ~Laborm*Materialm 0.800 0.299 0.208 -
## ------------------------------------------------
## M1 0.800 0.299 0.208
##
##
## $SA18a_high
## [1] "Error: None of the values in OUT is explained. Please check the truth table."
##
## $SA18b_low
## [1] "Error: None of the values in OUT is explained. Please check the truth table."
##
## $SA18b_high
## [1] "Error: None of the values in OUT is explained. Please check the truth table."
##
## $SA19_low
##
## M1: Demandm + Laborm -> SA19_low
##
## inclS PRI covS covU
## --------------------------------------
## 1 Demandm 0.791 0.718 0.891 0.107
## 2 Laborm 0.850 0.787 0.828 0.044
## --------------------------------------
## M1 0.784 0.711 0.935
##
##
## $SA19_high
##
## M1: Materialm*~RelCommm -> SA19_high
##
## inclS PRI covS covU
## --------------------------------------------------
## 1 Materialm*~RelCommm 0.767 0.457 0.359 -
## --------------------------------------------------
## M1 0.767 0.457 0.359
##
##
## $SA20_low
##
## M1: RelCommm + Laborm*Materialm -> SA20_low
##
## inclS PRI covS covU
## -----------------------------------------------
## 1 RelCommm 0.784 0.614 0.572 0.202
## 2 Laborm*Materialm 0.778 0.615 0.455 0.085
## -----------------------------------------------
## M1 0.757 0.600 0.657
##
##
## $SA20_high
## [1] "Error: None of the values in OUT is explained. Please check the truth table."
##

## $SA21_low
##
## M1: Demandm + Laborm -> SA21_low
##
## inclS PRI covS covU
## --------------------------------------
## 1 Demandm 0.807 0.745 0.893 0.118
## 2 Laborm 0.856 0.802 0.819 0.045
## --------------------------------------
## M1 0.800 0.739 0.937
##
##
## $SA21_high
##
## M1: Materialm + RelCommm + ~Demandm*Laborm -> SA21_high
##
## inclS PRI covS covU
## ----------------------------------------------
## 1 Materialm 0.725 0.547 0.480 0.074
## 2 RelCommm 0.769 0.593 0.599 0.144
## 3 ~Demandm*Laborm 0.798 0.456 0.274 0.035
## ----------------------------------------------
## M1 0.699 0.523 0.719
##
##
## $SA22_low
##
## M1: Demandm + Laborm -> SA22_low
##
## inclS PRI covS covU
## --------------------------------------
## 1 Demandm 0.862 0.828 0.877 0.133
## 2 Laborm 0.901 0.873 0.793 0.048
## --------------------------------------
## M1 0.858 0.825 0.926
##
##
## $SA22_high
##
## M1: Laborm + Materialm -> SA22_high
##
## inclS PRI covS covU
## ----------------------------------------
## 1 Laborm 0.761 0.688 0.838 0.408
## 2 Materialm 0.825 0.744 0.444 0.015
## ----------------------------------------
## M1 0.753 0.680 0.853
##
##

## $SA23_low
##
## M1: Materialm + RelCommm -> SA23_low
##
## inclS PRI covS covU
## ----------------------------------------
## 1 Materialm 0.773 0.604 0.468 0.097
## 2 RelCommm 0.802 0.640 0.571 0.200
## ----------------------------------------
## M1 0.763 0.604 0.668
##
##
## $SA23_high
## [1] "Error: None of the values in OUT is explained. Please check the truth table."
##
## $SA24_low
##
## M1: ~Laborm*Materialm -> SA24_low
##
## inclS PRI covS covU
## ------------------------------------------------
## 1 ~Laborm*Materialm 0.793 0.228 0.217 -
## ------------------------------------------------
## M1 0.793 0.228 0.217
##
##
## $SA24_high
## [1] "Error: None of the values in OUT is explained. Please check the truth table."
##
## $SA25_low
##
## M1: Demandm + Laborm -> SA25_low
##
## inclS PRI covS covU
## --------------------------------------
## 1 Demandm 0.850 0.806 0.889 0.129
## 2 Laborm 0.890 0.852 0.805 0.044
## --------------------------------------
## M1 0.842 0.799 0.934
##
##
## $SA25_high
##
## M1: Laborm + Materialm -> SA25_high
##
## inclS PRI covS covU
## ----------------------------------------
## 1 Laborm 0.722 0.597 0.878 0.435
## 2 Materialm 0.765 0.602 0.456 0.012
## ----------------------------------------
## M1 0.712 0.587 0.891

# Intermediate solutions: Positive output including the remainders and with directional expectations
# Result: Final QCA INTERMEDIATE Solutions for Configurations of Reasons for MNC triggering single Outcomes of MNC (n.cut=3)

## $SA1_low
##
## From C1P1:
##
## M1: Demandm*Laborm + Demandm*~RelCommm + Laborm*~Materialm*~RelCommm
## -> SA1_low
##
## inclS PRI covS covU
## ----------------------------------------------------------
## 1 Demandm*Laborm 0.900 0.862 0.775 0.236
## 2 Demandm*~RelCommm 0.881 0.822 0.625 0.086
## 3 Laborm*~Materialm*~RelCommm 0.928 0.875 0.484 0.020
## ----------------------------------------------------------
## M1 0.863 0.818 0.882
##
##
## $SA1_high
##
## From C1P1:
##
## M1: Demandm*Laborm*RelCommm + Demandm*Materialm*~RelCommm +
## ~Demandm*Laborm*~Materialm*~RelCommm -> SA1_high
##
## inclS PRI covS covU
## -------------------------------------------------------------------
## 1 Demandm*Laborm*RelCommm 0.803 0.626 0.560 0.249
## 2 Demandm*Materialm*~RelCommm 0.828 0.582 0.332 0.060
## 3 ~Demandm*Laborm*~Materialm*~RelCommm 0.826 0.448 0.240 0.045
## -------------------------------------------------------------------
## M1 0.759 0.579 0.674
##
##
## $SA2_low
##
## From C1P1:
##
## M1: Demandm*Materialm*~RelCommm + Demandm*Laborm*~Materialm*RelCommm
## -> SA2_low
##
## inclS PRI covS covU
## -----------------------------------------------------------------
## 1 Demandm*Materialm*~RelCommm 0.788 0.516 0.317 0.110
## 2 Demandm*Laborm*~Materialm*RelCommm 0.813 0.590 0.407 0.200
## -----------------------------------------------------------------
## M1 0.780 0.578 0.517
##
##
## $SA2_high
## [1] "Error: None of the values in OUT is explained. Please check the truth table."
##

## $SA3_low
##
## From C1P1:
##
## M1: Demandm*Laborm*Materialm + Demandm*Materialm*~RelCommm -> SA3_low
##
## inclS PRI covS covU
## ----------------------------------------------------------
## 1 Demandm*Laborm*Materialm 0.797 0.644 0.456 0.133
## 2 Demandm*Materialm*~RelCommm 0.834 0.624 0.334 0.010
## ----------------------------------------------------------
## M1 0.791 0.640 0.466
##
##
## $SA3_high
## [1] "Error: None of the values in OUT is explained. Please check the truth table."
##
## $SA4_low
## [1] "Error: None of the values in OUT is explained. Please check the truth table."
##
## $SA4_high
## [1] "Error: None of the values in OUT is explained. Please check the truth table."
##
## $SA5_low
##
## From C1P1:
##
## M1: Demandm*Laborm + Demandm*~RelCommm + Laborm*~Materialm*~RelCommm
## -> SA5_low
##
## inclS PRI covS covU
## ----------------------------------------------------------
## 1 Demandm*Laborm 0.781 0.666 0.819 0.229
## 2 Demandm*~RelCommm 0.771 0.620 0.667 0.077
## 3 Laborm*~Materialm*~RelCommm 0.822 0.648 0.522 0.020
## ----------------------------------------------------------
## M1 0.735 0.619 0.916
##
##
## $SA5_high
##
## From C1P1:
##
## M1: Demandm*~Laborm*Materialm*~RelCommm -> SA5_high
##
## inclS PRI covS covU
## ------------------------------------------------------------------
## 1 Demandm*~Laborm*Materialm*~RelCommm 0.810 0.181 0.286 -
## ------------------------------------------------------------------
## M1 0.810 0.181 0.286
##
##

## $SA6_low
##
## From C1P1:
##
## M1: Demandm*Laborm + Demandm*Materialm*~RelCommm -> SA6_low
##
## inclS PRI covS covU
## ----------------------------------------------------------
## 1 Demandm*Laborm 0.748 0.629 0.826 0.523
## 2 Demandm*Materialm*~RelCommm 0.822 0.610 0.307 0.004
## ----------------------------------------------------------
## M1 0.741 0.620 0.830
##
##
## $SA6_high
## [1] "Error: None of the values in OUT is explained. Please check the truth table."
##
## $SA7_low
##
## From C1P1:
##
## M1: Demandm*Laborm*RelCommm + Demandm*Materialm*~RelCommm -> SA7_low
##
## inclS PRI covS covU
## ----------------------------------------------------------
## 1 Demandm*Laborm*RelCommm 0.845 0.727 0.537 0.304
## 2 Demandm*Materialm*~RelCommm 0.850 0.647 0.311 0.078
## ----------------------------------------------------------
## M1 0.828 0.709 0.615
##
##
## $SA7_high
## [1] "Error: None of the values in OUT is explained. Please check the truth table."
##
## $SA8_low
##
## From C1P1:
##
## M1: Demandm*Laborm + Demandm*~RelCommm + Laborm*~Materialm*~RelCommm
## -> SA8_low
##
## inclS PRI covS covU
## ----------------------------------------------------------
## 1 Demandm*Laborm 0.856 0.800 0.766 0.231
## 2 Demandm*~RelCommm 0.847 0.771 0.625 0.090
## 3 Laborm*~Materialm*~RelCommm 0.885 0.800 0.479 0.022
## ----------------------------------------------------------
## M1 0.826 0.768 0.878
##
##

## $SA8_high
##
## From C1P1:
##
## M1: Demandm*Laborm*RelCommm + Demandm*Materialm*~RelCommm +
## ~Demandm*Laborm*~Materialm*~RelCommm -> SA8_high
##
## inclS PRI covS covU
## -------------------------------------------------------------------
## 1 Demandm*Laborm*RelCommm 0.772 0.581 0.571 0.252
## 2 Demandm*Materialm*~RelCommm 0.817 0.572 0.347 0.063
## 3 ~Demandm*Laborm*~Materialm*~RelCommm 0.804 0.420 0.248 0.045
## -------------------------------------------------------------------
## M1 0.731 0.549 0.690
##
##
## $SA9a_low
## [1] "Error: All truth table configurations are used, all conditions are minimized. Please check the truth table."
##
## $SA9a_high
##
## From C1P1:
##
## M1: Demandm*Laborm + Demandm*~RelCommm + Laborm*~Materialm*~RelCommm
## -> SA9a_high
##
## inclS PRI covS covU
## ----------------------------------------------------------
## 1 Demandm*Laborm 0.822 0.751 0.797 0.239
## 2 Demandm*~RelCommm 0.793 0.684 0.633 0.076
## 3 Laborm*~Materialm*~RelCommm 0.850 0.732 0.499 0.020
## ----------------------------------------------------------
## M1 0.776 0.699 0.892
##
##
## $SA9b_low
## [1] "Error: All truth table configurations are used, all conditions are minimized. Please check the truth table."
##
## $SA9b_high
##
## From C1P1:
##
## M1: Demandm*Laborm + Demandm*~RelCommm + Laborm*~Materialm*~RelCommm
## -> SA9b_high
##
## inclS PRI covS covU
## ----------------------------------------------------------
## 1 Demandm*Laborm 0.819 0.743 0.793 0.229
## 2 Demandm*~RelCommm 0.805 0.700 0.642 0.078
## 3 Laborm*~Materialm*~RelCommm 0.866 0.757 0.507 0.020
## ----------------------------------------------------------
## M1 0.775 0.695 0.891
##
##

## $SA10a_low
## [1] "Error: All truth table configurations are used, all conditions are minimized. Please check the truth table."
##
## $SA10a_high
##
## From C1P1:
##
## M1: Demandm*Laborm + Demandm*~RelCommm + Laborm*~Materialm*~RelCommm
## -> SA10a_high
##
## inclS PRI covS covU
## ----------------------------------------------------------
## 1 Demandm*Laborm 0.811 0.734 0.796 0.230
## 2 Demandm*~RelCommm 0.792 0.682 0.641 0.074
## 3 Laborm*~Materialm*~RelCommm 0.852 0.737 0.506 0.019
## ----------------------------------------------------------
## M1 0.763 0.681 0.889
##
##
## $SA10b_low
## [1] "Error: All truth table configurations are used, all conditions are minimized. Please check the truth table."
##
## $SA10b_high
##
## From C1P1:
##
## M1: Demandm*Laborm + Demandm*~RelCommm + Laborm*~Materialm*~RelCommm
## -> SA10b_high
##
## inclS PRI covS covU
## ----------------------------------------------------------
## 1 Demandm*Laborm 0.885 0.845 0.772 0.239
## 2 Demandm*~RelCommm 0.850 0.783 0.612 0.078
## 3 Laborm*~Materialm*~RelCommm 0.913 0.856 0.482 0.023
## ----------------------------------------------------------
## M1 0.843 0.796 0.873
##
##
## $SA11_low
##
## From C1P1:
##
## M1: Demandm*Materialm*~RelCommm + Demandm*Laborm*~Materialm*RelCommm
## -> SA11_low
##
## inclS PRI covS covU
## -----------------------------------------------------------------
## 1 Demandm*Materialm*~RelCommm 0.797 0.482 0.333 0.117
## 2 Demandm*Laborm*~Materialm*RelCommm 0.806 0.524 0.418 0.203
## -----------------------------------------------------------------
## M1 0.778 0.528 0.536
##
##

## $SA11_high
## [1] "Error: None of the values in OUT is explained. Please check the truth table."
##
## $SA12_low
##
## From C1P1:
##
## M1: Demandm*Laborm + Demandm*~RelCommm + Laborm*~Materialm*~RelCommm
## -> SA12_low
##
## inclS PRI covS covU
## ----------------------------------------------------------
## 1 Demandm*Laborm 0.778 0.673 0.817 0.232
## 2 Demandm*~RelCommm 0.761 0.614 0.659 0.074
## 3 Laborm*~Materialm*~RelCommm 0.817 0.650 0.519 0.019
## ----------------------------------------------------------
## M1 0.730 0.623 0.910
##
##
## $SA12_high
## [1] "Error: None of the values in OUT is explained. Please check the truth table."
##
## $SA13_low
## [1] "Error: None of the values in OUT is explained. Please check the truth table."
##
## $SA13_high
## [1] "Error: None of the values in OUT is explained. Please check the truth table."
##
## $SA14a_low
##
## From C1P1:
##
## M1: Demandm*Laborm + Demandm*Materialm*~RelCommm +
## Laborm*~Materialm*~RelCommm -> SA14a_low
##
## inclS PRI covS covU
## ----------------------------------------------------------
## 1 Demandm*Laborm 0.783 0.671 0.822 0.245
## 2 Demandm*Materialm*~RelCommm 0.864 0.665 0.306 0.005
## 3 Laborm*~Materialm*~RelCommm 0.810 0.629 0.515 0.018
## ----------------------------------------------------------
## M1 0.769 0.653 0.845
##
##
## $SA14a_high
## [1] "Error: None of the values in OUT is explained. Please check the truth table."
##

## $SA14b_low
##
## From C1P1:
##
## M1: Demandm*Laborm + Demandm*~RelCommm + Laborm*~Materialm*~RelCommm
## -> SA14b_low
##
## inclS PRI covS covU
## ----------------------------------------------------------
## 1 Demandm*Laborm 0.896 0.853 0.784 0.241
## 2 Demandm*~RelCommm 0.870 0.798 0.627 0.085
## 3 Laborm*~Materialm*~RelCommm 0.911 0.840 0.482 0.019
## ----------------------------------------------------------
## M1 0.854 0.803 0.887
##
##
## $SA14b_high
##
## From C1P1:
##
## M1: Demandm*Laborm*RelCommm + Demandm*Materialm*~RelCommm -> SA14b_high
##
## inclS PRI covS covU
## ----------------------------------------------------------
## 1 Demandm*Laborm*RelCommm 0.811 0.667 0.580 0.315
## 2 Demandm*Materialm*~RelCommm 0.828 0.598 0.341 0.075
## ----------------------------------------------------------
## M1 0.784 0.638 0.656
##
##
## $SA14c_low
##
## From C1P1:
##
## M1: Demandm*Laborm + Demandm*~RelCommm + Laborm*~Materialm*~RelCommm
## -> SA14c_low
##
## inclS PRI covS covU
## ----------------------------------------------------------
## 1 Demandm*Laborm 0.916 0.889 0.759 0.238
## 2 Demandm*~RelCommm 0.890 0.843 0.607 0.087
## 3 Laborm*~Materialm*~RelCommm 0.930 0.887 0.466 0.020
## ----------------------------------------------------------
## M1 0.881 0.848 0.866
##
##

## $SA14c_high
##
## From C1P1:
##
## M1: Demandm*Laborm + Demandm*Materialm*~RelCommm +
## Laborm*~Materialm*~RelCommm -> SA14c_high
##
## inclS PRI covS covU
## ----------------------------------------------------------
## 1 Demandm*Laborm 0.770 0.680 0.816 0.258
## 2 Demandm*Materialm*~RelCommm 0.846 0.670 0.303 0.008
## 3 Laborm*~Materialm*~RelCommm 0.798 0.654 0.512 0.017
## ----------------------------------------------------------
## M1 0.758 0.668 0.841
##
##
## $SA15_low
## [1] "Error: None of the values in OUT is explained. Please check the truth table."
##
## $SA15_high
## [1] "Error: None of the values in OUT is explained. Please check the truth table."
##
## $SA16_low
##
## From C1P1:
##
## M1: Demandm*Laborm + Demandm*~RelCommm + Laborm*~Materialm*~RelCommm
## -> SA16_low
##
## inclS PRI covS covU
## ----------------------------------------------------------
## 1 Demandm*Laborm 0.822 0.737 0.791 0.232
## 2 Demandm*~RelCommm 0.815 0.700 0.646 0.087
## 3 Laborm*~Materialm*~RelCommm 0.854 0.725 0.497 0.018
## ----------------------------------------------------------
## M1 0.784 0.697 0.895
##
##
## $SA16_high
##
## From C1P1:
##
## M1: Demandm*~Laborm*Materialm*~RelCommm -> SA16_high
##
## inclS PRI covS covU
## ------------------------------------------------------------------
## 1 Demandm*~Laborm*Materialm*~RelCommm 0.816 0.238 0.245 -
## ------------------------------------------------------------------
## M1 0.816 0.238 0.245
##
##

## $SA17_low
##
## From C1P1:
##
## M1: Demandm*Laborm + Demandm*~RelCommm + Laborm*~Materialm*~RelCommm
## -> SA17_low
##
## inclS PRI covS covU
## ----------------------------------------------------------
## 1 Demandm*Laborm 0.867 0.821 0.768 0.237
## 2 Demandm*~RelCommm 0.850 0.782 0.621 0.090
## 3 Laborm*~Materialm*~RelCommm 0.881 0.803 0.472 0.018
## ----------------------------------------------------------
## M1 0.833 0.783 0.875
##
##
## $SA17_high
##
## From C1P1:
##
## M1: Demandm*Laborm*RelCommm + Demandm*Materialm*~RelCommm -> SA17_high
##
## inclS PRI covS covU
## ----------------------------------------------------------
## 1 Demandm*Laborm*RelCommm 0.788 0.643 0.547 0.288
## 2 Demandm*Materialm*~RelCommm 0.830 0.641 0.332 0.072
## ----------------------------------------------------------
## M1 0.764 0.626 0.620
##
##
## $SA18a_low
##
## From C1P1:
##
## M1: Demandm*~Laborm*Materialm*~RelCommm -> SA18a_low
##
## inclS PRI covS covU
## ------------------------------------------------------------------
## 1 Demandm*~Laborm*Materialm*~RelCommm 0.809 0.300 0.201 -
## ------------------------------------------------------------------
## M1 0.809 0.300 0.201
##
##
## $SA18a_high
## [1] "Error: None of the values in OUT is explained. Please check the truth table."
##
## $SA18b_low
## [1] "Error: None of the values in OUT is explained. Please check the truth table."
##
## $SA18b_high
## [1] "Error: None of the values in OUT is explained. Please check the truth table."
##

## $SA19_low
##
## From C1P1:
##
## M1: Demandm*Laborm + Demandm*~RelCommm + Laborm*~Materialm*~RelCommm
## -> SA19_low
##
## inclS PRI covS covU
## ----------------------------------------------------------
## 1 Demandm*Laborm 0.864 0.803 0.784 0.229
## 2 Demandm*~RelCommm 0.852 0.767 0.637 0.082
## 3 Laborm*~Materialm*~RelCommm 0.898 0.813 0.493 0.020
## ----------------------------------------------------------
## M1 0.823 0.755 0.886
##
##
## $SA19_high
##
## From C1P1:
##
## M1: Demandm*Materialm*~RelCommm -> SA19_high
##
## inclS PRI covS covU
## ----------------------------------------------------------
## 1 Demandm*Materialm*~RelCommm 0.779 0.474 0.356 -
## ----------------------------------------------------------
## M1 0.779 0.474 0.356
##
##
## $SA20_low
##
## From C1P1:
##
## M1: Demandm*Laborm*Materialm + Demandm*Laborm*RelCommm -> SA20_low
##
## inclS PRI covS covU
## -------------------------------------------------------
## 1 Demandm*Laborm*Materialm 0.781 0.617 0.445 0.080
## 2 Demandm*Laborm*RelCommm 0.797 0.627 0.552 0.187
## -------------------------------------------------------
## M1 0.769 0.610 0.633
##
##
## $SA20_high
## [1] "Error: None of the values in OUT is explained. Please check the truth table."
##

## $SA21_low
##
## From C1P1:
##
## M1: Demandm*Laborm + Demandm*~RelCommm + Laborm*~Materialm*~RelCommm
## -> SA21_low
##
## inclS PRI covS covU
## ----------------------------------------------------------
## 1 Demandm*Laborm 0.869 0.817 0.774 0.232
## 2 Demandm*~RelCommm 0.860 0.787 0.631 0.089
## 3 Laborm*~Materialm*~RelCommm 0.892 0.812 0.481 0.018
## ----------------------------------------------------------
## M1 0.833 0.775 0.881
##
##
## $SA21_high
##
## From C1P1:
##
## M1: Demandm*Laborm*RelCommm + Demandm*Materialm*~RelCommm +
## ~Demandm*Laborm*~Materialm*~RelCommm -> SA21_high
##
## inclS PRI covS covU
## -------------------------------------------------------------------
## 1 Demandm*Laborm*RelCommm 0.778 0.597 0.575 0.253
## 2 Demandm*Materialm*~RelCommm 0.801 0.553 0.341 0.056
## 3 ~Demandm*Laborm*~Materialm*~RelCommm 0.803 0.387 0.247 0.039
## -------------------------------------------------------------------
## M1 0.722 0.535 0.681
##
##
## $SA22_low
##
## From C1P1:
##
## M1: Demandm*Laborm + Demandm*~RelCommm + Laborm*~Materialm*~RelCommm
## -> SA22_low
##
## inclS PRI covS covU
## ----------------------------------------------------------
## 1 Demandm*Laborm 0.909 0.881 0.745 0.228
## 2 Demandm*~RelCommm 0.902 0.863 0.609 0.092
## 3 Laborm*~Materialm*~RelCommm 0.931 0.892 0.462 0.019
## ----------------------------------------------------------
## M1 0.880 0.848 0.856
##
##

## $SA22_high
##
## From C1P1:
##
## M1: Demandm*Laborm + Demandm*Materialm*~RelCommm +
## Laborm*~Materialm*~RelCommm -> SA22_high
##
## inclS PRI covS covU
## ----------------------------------------------------------
## 1 Demandm*Laborm 0.770 0.695 0.790 0.239
## 2 Demandm*Materialm*~RelCommm 0.893 0.802 0.309 0.008
## 3 Laborm*~Materialm*~RelCommm 0.794 0.669 0.493 0.016
## ----------------------------------------------------------
## M1 0.759 0.682 0.814
##
##
## $SA23_low
##
## From C1P1:
##
## M1: Demandm*Laborm*RelCommm + Demandm*Materialm*~RelCommm -> SA23_low
##
## inclS PRI covS covU
## ----------------------------------------------------------
## 1 Demandm*Laborm*RelCommm 0.811 0.646 0.548 0.305
## 2 Demandm*Materialm*~RelCommm 0.819 0.550 0.318 0.075
## ----------------------------------------------------------
## M1 0.789 0.627 0.623
##
##
## $SA23_high
## [1] "Error: None of the values in OUT is explained. Please check the truth table."
##
## $SA24_low
##
## From C1P1:
##
## M1: Demandm*~Laborm*Materialm*~RelCommm -> SA24_low
##
## inclS PRI covS covU
## ------------------------------------------------------------------
## 1 Demandm*~Laborm*Materialm*~RelCommm 0.802 0.212 0.209 -
## ------------------------------------------------------------------
## M1 0.802 0.212 0.209
##
##
## $SA24_high
## [1] "Error: None of the values in OUT is explained. Please check the truth table."
##

## $SA25_low
##
## From C1P1:
##
## M1: Demandm*Laborm + Demandm*~RelCommm + Laborm*~Materialm*~RelCommm
## -> SA25_low
##
## inclS PRI covS covU
## ----------------------------------------------------------
## 1 Demandm*Laborm 0.903 0.867 0.760 0.233
## 2 Demandm*~RelCommm 0.899 0.851 0.624 0.097
## 3 Laborm*~Materialm*~RelCommm 0.931 0.883 0.474 0.020
## ----------------------------------------------------------
## M1 0.878 0.839 0.877
##
##
## $SA25_high
##
## From C1P1:
##
## M1: Demandm*Laborm + Demandm*Materialm*~RelCommm +
## Laborm*~Materialm*~RelCommm -> SA25_high
##
## inclS PRI covS covU
## ----------------------------------------------------------
## 1 Demandm*Laborm 0.737 0.610 0.835 0.245
## 2 Demandm*Materialm*~RelCommm 0.835 0.603 0.320 0.007
## 3 Laborm*~Materialm*~RelCommm 0.789 0.592 0.541 0.019
## ----------------------------------------------------------
## M1 0.726 0.598 0.862

#### FOR n.cut (minimum number of cases with membership > 0.5 for an outcome) -> n9:n.cut=9

# Complex solutions: Positive output
# Apply minimize to each truth table in TTCondsReasons

# Result: Final QCA COMPLEX Solutions for Configurations of Reasons for MNC triggering single Outcomes of MNC (n.cut=9)

## $SA1_low
##
## M1: Demandm*Laborm + Demandm*~Materialm*~RelCommm + Laborm*~Materialm*~RelCommm
## -> SA1_low
##
## inclS PRI covS covU
## -----------------------------------------------------------
## 1 Demandm*Laborm 0.900 0.862 0.775 0.312
## 2 Demandm*~Materialm*~RelCommm 0.890 0.825 0.539 0.076
## 3 Laborm*~Materialm*~RelCommm 0.928 0.875 0.484 0.020
## -----------------------------------------------------------
## M1 0.870 0.826 0.871
##
##
## $SA1_high
##
## M1: Demandm*Laborm*Materialm + Demandm*Laborm*RelCommm +
## ~Demandm*Laborm*~Materialm*~RelCommm -> SA1_high
##
## inclS PRI covS covU
## -------------------------------------------------------------------
## 1 Demandm*Laborm*Materialm 0.765 0.565 0.439 0.067
## 2 Demandm*Laborm*RelCommm 0.803 0.626 0.560 0.149
## 3 ~Demandm*Laborm*~Materialm*~RelCommm 0.826 0.448 0.240 0.045
## -------------------------------------------------------------------
## M1 0.755 0.578 0.681
##
##
## $SA2_low
##
## M1: Demandm*Laborm*Materialm*~RelCommm + Demandm*Laborm*~Materialm*RelCommm
## -> SA2_low
##
## inclS PRI covS covU
## -----------------------------------------------------------------
## 1 Demandm*Laborm*Materialm*~RelCommm 0.803 0.529 0.309 0.102
## 2 Demandm*Laborm*~Materialm*RelCommm 0.813 0.590 0.407 0.200
## -----------------------------------------------------------------
## M1 0.788 0.587 0.509
##
##
## $SA2_high
## [1] "Error: None of the values in OUT is explained. Please check the truth table."
##

## $SA3_low
##
## M1: Demandm*Laborm*Materialm -> SA3_low
##
## inclS PRI covS covU
## -------------------------------------------------------
## 1 Demandm*Laborm*Materialm 0.797 0.644 0.456 -
## -------------------------------------------------------
## M1 0.797 0.644 0.456
##
##
## $SA3_high
## [1] "Error: None of the values in OUT is explained. Please check the truth table."
##
## $SA4_low
## [1] "Error: None of the values in OUT is explained. Please check the truth table."
##
## $SA4_high
## [1] "Error: None of the values in OUT is explained. Please check the truth table."
##
## $SA5_low
##
## M1: Demandm*Laborm + Demandm*~Materialm*~RelCommm + Laborm*~Materialm*~RelCommm
## -> SA5_low
##
## inclS PRI covS covU
## -----------------------------------------------------------
## 1 Demandm*Laborm 0.781 0.666 0.819 0.317
## 2 Demandm*~Materialm*~RelCommm 0.770 0.593 0.569 0.066
## 3 Laborm*~Materialm*~RelCommm 0.822 0.648 0.522 0.020
## -----------------------------------------------------------
## M1 0.741 0.625 0.905
##
##
## $SA5_high
## [1] "Error: None of the values in OUT is explained. Please check the truth table."
##
## $SA6_low
##
## M1: Demandm*Laborm -> SA6_low
##
## inclS PRI covS covU
## ---------------------------------------------
## 1 Demandm*Laborm 0.748 0.629 0.826 -
## ---------------------------------------------
## M1 0.748 0.629 0.826
##
##
## $SA6_high
## [1] "Error: None of the values in OUT is explained. Please check the truth table."
##

## $SA7_low
##
## M1: Demandm*Laborm*Materialm + Demandm*Laborm*RelCommm -> SA7_low
##
## inclS PRI covS covU
## -------------------------------------------------------
## 1 Demandm*Laborm*Materialm 0.821 0.683 0.430 0.082
## 2 Demandm*Laborm*RelCommm 0.845 0.727 0.537 0.189
## -------------------------------------------------------
## M1 0.820 0.701 0.619
##
##
## $SA7_high
## [1] "Error: None of the values in OUT is explained. Please check the truth table."
##
## $SA8_low
##
## M1: Demandm*Laborm + Demandm*~Materialm*~RelCommm + Laborm*~Materialm*~RelCommm
## -> SA8_low
##
## inclS PRI covS covU
## -----------------------------------------------------------
## 1 Demandm*Laborm 0.856 0.800 0.766 0.308
## 2 Demandm*~Materialm*~RelCommm 0.854 0.767 0.538 0.080
## 3 Laborm*~Materialm*~RelCommm 0.885 0.800 0.479 0.022
## -----------------------------------------------------------
## M1 0.833 0.776 0.868
##
##
## $SA8_high
##
## M1: Demandm*Laborm*Materialm + Demandm*Laborm*RelCommm +
## ~Demandm*Laborm*~Materialm*~RelCommm -> SA8_high
##
## inclS PRI covS covU
## -------------------------------------------------------------------
## 1 Demandm*Laborm*Materialm 0.758 0.569 0.461 0.067
## 2 Demandm*Laborm*RelCommm 0.772 0.581 0.571 0.143
## 3 ~Demandm*Laborm*~Materialm*~RelCommm 0.804 0.420 0.248 0.045
## -------------------------------------------------------------------
## M1 0.725 0.543 0.694
##
##
## $SA9a_low
##
## M1: Demandm*Laborm + ~Materialm*~RelCommm -> SA9a_low
##
## inclS PRI covS covU
## ---------------------------------------------------
## 1 Demandm*Laborm 0.934 0.914 0.733 0.303
## 2 ~Materialm*~RelCommm 0.848 0.792 0.581 0.151
## ---------------------------------------------------
## M1 0.866 0.834 0.884
##
##

## $SA9a_high
##
## M1: Demandm*Laborm + Demandm*~Materialm*~RelCommm + Laborm*~Materialm*~RelCommm
## -> SA9a_high
##
## inclS PRI covS covU
## -----------------------------------------------------------
## 1 Demandm*Laborm 0.822 0.751 0.797 0.318
## 2 Demandm*~Materialm*~RelCommm 0.795 0.669 0.542 0.063
## 3 Laborm*~Materialm*~RelCommm 0.850 0.732 0.499 0.020
## -----------------------------------------------------------
## M1 0.780 0.703 0.880
##
##
## $SA9b_low
##
## M1: Demandm*Laborm + ~Materialm*~RelCommm -> SA9b_low
##
## inclS PRI covS covU
## ---------------------------------------------------
## 1 Demandm*Laborm 0.931 0.912 0.732 0.301
## 2 ~Materialm*~RelCommm 0.851 0.799 0.583 0.153
## ---------------------------------------------------
## M1 0.866 0.835 0.884
##
##
## $SA9b_high
##
## M1: Demandm*Laborm + Demandm*~Materialm*~RelCommm + Laborm*~Materialm*~RelCommm
## -> SA9b_high
##
## inclS PRI covS covU
## -----------------------------------------------------------
## 1 Demandm*Laborm 0.819 0.743 0.793 0.306
## 2 Demandm*~Materialm*~RelCommm 0.811 0.692 0.552 0.066
## 3 Laborm*~Materialm*~RelCommm 0.866 0.757 0.507 0.020
## -----------------------------------------------------------
## M1 0.780 0.700 0.878
##
##
## $SA10a_low
##
## M1: Demandm*Laborm + ~Materialm*~RelCommm -> SA10a_low
##
## inclS PRI covS covU
## ---------------------------------------------------
## 1 Demandm*Laborm 0.934 0.914 0.735 0.305
## 2 ~Materialm*~RelCommm 0.852 0.798 0.585 0.155
## ---------------------------------------------------
## M1 0.870 0.838 0.890
##
##

## $SA10a_high
##
## M1: Demandm*Laborm + Demandm*~Materialm*~RelCommm + Laborm*~Materialm*~RelCommm
## -> SA10a_high
##
## inclS PRI covS covU
## -----------------------------------------------------------
## 1 Demandm*Laborm 0.811 0.734 0.796 0.309
## 2 Demandm*~Materialm*~RelCommm 0.799 0.675 0.552 0.064
## 3 Laborm*~Materialm*~RelCommm 0.852 0.737 0.506 0.019
## -----------------------------------------------------------
## M1 0.770 0.688 0.879
##
##
## $SA10b_low
##
## M1: Demandm*Laborm + ~Materialm*~RelCommm -> SA10b_low
##
## inclS PRI covS covU
## ---------------------------------------------------
## 1 Demandm*Laborm 0.961 0.952 0.719 0.303
## 2 ~Materialm*~RelCommm 0.877 0.841 0.572 0.157
## ---------------------------------------------------
## M1 0.901 0.881 0.875
##
##
## $SA10b_high
##
## M1: Demandm*Laborm + Demandm*~Materialm*~RelCommm + Laborm*~Materialm*~RelCommm
## -> SA10b_high
##
## inclS PRI covS covU
## -----------------------------------------------------------
## 1 Demandm*Laborm 0.885 0.845 0.772 0.313
## 2 Demandm*~Materialm*~RelCommm 0.856 0.780 0.526 0.066
## 3 Laborm*~Materialm*~RelCommm 0.913 0.856 0.482 0.023
## -----------------------------------------------------------
## M1 0.848 0.801 0.861
##
##
## $SA11_low
##
## M1: Demandm*Laborm*Materialm*~RelCommm + Demandm*Laborm*~Materialm*RelCommm
## -> SA11_low
##
## inclS PRI covS covU
## -----------------------------------------------------------------
## 1 Demandm*Laborm*Materialm*~RelCommm 0.820 0.508 0.328 0.112
## 2 Demandm*Laborm*~Materialm*RelCommm 0.806 0.524 0.418 0.203
## -----------------------------------------------------------------
## M1 0.791 0.543 0.531
##
##
## $SA11_high
## [1] "Error: None of the values in OUT is explained. Please check the truth table."
##
## $SA12_low
##
## M1: Demandm*Laborm + Demandm*~Materialm*~RelCommm + Laborm*~Materialm*~RelCommm
## -> SA12_low
##
## inclS PRI covS covU
## -----------------------------------------------------------
## 1 Demandm*Laborm 0.778 0.673 0.817 0.317
## 2 Demandm*~Materialm*~RelCommm 0.769 0.604 0.568 0.068
## 3 Laborm*~Materialm*~RelCommm 0.817 0.650 0.519 0.019
## -----------------------------------------------------------
## M1 0.740 0.634 0.904
##
##
## $SA12_high
## [1] "Error: None of the values in OUT is explained. Please check the truth table."
##
## $SA13_low
## [1] "Error: None of the values in OUT is explained. Please check the truth table."
##
## $SA13_high
## [1] "Error: None of the values in OUT is explained. Please check the truth table."
##
## $SA14a_low
##
## M1: Demandm*Laborm + Laborm*~Materialm*~RelCommm -> SA14a_low
##
## inclS PRI covS covU
## ----------------------------------------------------------
## 1 Demandm*Laborm 0.783 0.671 0.822 0.325
## 2 Laborm*~Materialm*~RelCommm 0.810 0.629 0.515 0.018
## ----------------------------------------------------------
## M1 0.775 0.661 0.840
##
##
## $SA14a_high
## [1] "Error: None of the values in OUT is explained. Please check the truth table."
##
## $SA14b_low
##
## M1: Demandm*Laborm + Demandm*~Materialm*~RelCommm + Laborm*~Materialm*~RelCommm
## -> SA14b_low
##
## inclS PRI covS covU
## -----------------------------------------------------------
## 1 Demandm*Laborm 0.896 0.853 0.784 0.320
## 2 Demandm*~Materialm*~RelCommm 0.869 0.786 0.535 0.071
## 3 Laborm*~Materialm*~RelCommm 0.911 0.840 0.482 0.019
## -----------------------------------------------------------
## M1 0.859 0.807 0.873
##
##

## $SA14b_high
##
## M1: Demandm*Laborm*Materialm + Demandm*Laborm*RelCommm -> SA14b_high
##
## inclS PRI covS covU
## -------------------------------------------------------
## 1 Demandm*Laborm*Materialm 0.781 0.614 0.461 0.078
## 2 Demandm*Laborm*RelCommm 0.811 0.667 0.580 0.198
## -------------------------------------------------------
## M1 0.775 0.629 0.659
##
##
## $SA14c_low
##
## M1: Demandm*Laborm + Demandm*~Materialm*~RelCommm + Laborm*~Materialm*~RelCommm
## -> SA14c_low
##
## inclS PRI covS covU
## -----------------------------------------------------------
## 1 Demandm*Laborm 0.916 0.889 0.759 0.313
## 2 Demandm*~Materialm*~RelCommm 0.893 0.840 0.520 0.075
## 3 Laborm*~Materialm*~RelCommm 0.930 0.887 0.466 0.020
## -----------------------------------------------------------
## M1 0.886 0.853 0.854
##
##
## $SA14c_high
##
## M1: Demandm*Laborm + Laborm*~Materialm*~RelCommm -> SA14c_high
##
## inclS PRI covS covU
## ----------------------------------------------------------
## 1 Demandm*Laborm 0.770 0.680 0.816 0.321
## 2 Laborm*~Materialm*~RelCommm 0.798 0.654 0.512 0.017
## ----------------------------------------------------------
## M1 0.761 0.671 0.833
##
##
## $SA15_low
## [1] "Error: None of the values in OUT is explained. Please check the truth table."
##
## $SA15_high
## [1] "Error: None of the values in OUT is explained. Please check the truth table."
##

## $SA16_low
##
## M1: Demandm*Laborm + Demandm*~Materialm*~RelCommm + Laborm*~Materialm*~RelCommm
## -> SA16_low
##
## inclS PRI covS covU
## -----------------------------------------------------------
## 1 Demandm*Laborm 0.822 0.737 0.791 0.311
## 2 Demandm*~Materialm*~RelCommm 0.824 0.700 0.558 0.078
## 3 Laborm*~Materialm*~RelCommm 0.854 0.725 0.497 0.018
## -----------------------------------------------------------
## M1 0.792 0.706 0.887
##
##
## $SA16_high
## [1] "Error: None of the values in OUT is explained. Please check the truth table."
##
## $SA17_low
##
## M1: Demandm*Laborm + Demandm*~Materialm*~RelCommm + Laborm*~Materialm*~RelCommm
## -> SA17_low
##
## inclS PRI covS covU
## -----------------------------------------------------------
## 1 Demandm*Laborm 0.867 0.821 0.768 0.314
## 2 Demandm*~Materialm*~RelCommm 0.852 0.772 0.531 0.076
## 3 Laborm*~Materialm*~RelCommm 0.881 0.803 0.472 0.018
## -----------------------------------------------------------
## M1 0.837 0.787 0.862
##
##
## $SA17_high
##
## M1: Demandm*Laborm*Materialm + Demandm*Laborm*RelCommm -> SA17_high
##
## inclS PRI covS covU
## -------------------------------------------------------
## 1 Demandm*Laborm*Materialm 0.779 0.632 0.446 0.079
## 2 Demandm*Laborm*RelCommm 0.788 0.643 0.547 0.181
## -------------------------------------------------------
## M1 0.760 0.624 0.627
##
##
## $SA18a_low
## [1] "Error: None of the values in OUT is explained. Please check the truth table."
##
## $SA18a_high
## [1] "Error: None of the values in OUT is explained. Please check the truth table."
##
## $SA18b_low
## [1] "Error: None of the values in OUT is explained. Please check the truth table."
##

## $SA18b_high
## [1] "Error: None of the values in OUT is explained. Please check the truth table."
##
## $SA19_low
##
## M1: Demandm*Laborm + Demandm*~Materialm*~RelCommm + Laborm*~Materialm*~RelCommm
## -> SA19_low
##
## inclS PRI covS covU
## -----------------------------------------------------------
## 1 Demandm*Laborm 0.864 0.803 0.784 0.310
## 2 Demandm*~Materialm*~RelCommm 0.852 0.754 0.544 0.071
## 3 Laborm*~Materialm*~RelCommm 0.898 0.813 0.493 0.020
## -----------------------------------------------------------
## M1 0.828 0.761 0.874
##
##
## $SA19_high
##
## M1: Demandm*Laborm*Materialm*~RelCommm -> SA19_high
##
## inclS PRI covS covU
## -----------------------------------------------------------------
## 1 Demandm*Laborm*Materialm*~RelCommm 0.800 0.494 0.350 -
## -----------------------------------------------------------------
## M1 0.800 0.494 0.350
##
##
## $SA20_low
##
## M1: Demandm*Laborm*Materialm + Demandm*Laborm*RelCommm -> SA20_low
##
## inclS PRI covS covU
## -------------------------------------------------------
## 1 Demandm*Laborm*Materialm 0.781 0.617 0.445 0.080
## 2 Demandm*Laborm*RelCommm 0.797 0.627 0.552 0.187
## -------------------------------------------------------
## M1 0.769 0.610 0.633
##
##
## $SA20_high
## [1] "Error: None of the values in OUT is explained. Please check the truth table."
##
## $SA21_low
##
## M1: Demandm*Laborm + Demandm*~Materialm*~RelCommm + Laborm*~Materialm*~RelCommm
## -> SA21_low
##
## inclS PRI covS covU
## -----------------------------------------------------------
## 1 Demandm*Laborm 0.869 0.817 0.774 0.311
## 2 Demandm*~Materialm*~RelCommm 0.863 0.780 0.541 0.078
## 3 Laborm*~Materialm*~RelCommm 0.892 0.812 0.481 0.018
## -----------------------------------------------------------
## M1 0.839 0.782 0.870
##
##
## $SA21_high
##
## M1: Demandm*Laborm*Materialm + Demandm*Laborm*RelCommm +
## ~Demandm*Laborm*~Materialm*~RelCommm -> SA21_high
##
## inclS PRI covS covU
## -------------------------------------------------------------------
## 1 Demandm*Laborm*Materialm 0.759 0.582 0.462 0.065
## 2 Demandm*Laborm*RelCommm 0.778 0.597 0.575 0.140
## 3 ~Demandm*Laborm*~Materialm*~RelCommm 0.803 0.387 0.247 0.039
## -------------------------------------------------------------------
## M1 0.720 0.537 0.689
##
##
## $SA22_low
##
## M1: Demandm*Laborm + Demandm*~Materialm*~RelCommm + Laborm*~Materialm*~RelCommm
## -> SA22_low
##
## inclS PRI covS covU
## -----------------------------------------------------------
## 1 Demandm*Laborm 0.909 0.881 0.745 0.302
## 2 Demandm*~Materialm*~RelCommm 0.903 0.858 0.521 0.078
## 3 Laborm*~Materialm*~RelCommm 0.931 0.892 0.462 0.019
## -----------------------------------------------------------
## M1 0.882 0.850 0.841
##
##
## $SA22_high
##
## M1: Demandm*Laborm + Laborm*~Materialm*~RelCommm -> SA22_high
##
## inclS PRI covS covU
## ----------------------------------------------------------
## 1 Demandm*Laborm 0.770 0.695 0.790 0.314
## 2 Laborm*~Materialm*~RelCommm 0.794 0.669 0.493 0.016
## ----------------------------------------------------------
## M1 0.761 0.685 0.806
##
##
## $SA23_low
##
## M1: Demandm*Laborm*Materialm + Demandm*Laborm*RelCommm -> SA23_low
##
## inclS PRI covS covU
## -------------------------------------------------------
## 1 Demandm*Laborm*Materialm 0.801 0.635 0.446 0.083
## 2 Demandm*Laborm*RelCommm 0.811 0.646 0.548 0.185
## -------------------------------------------------------
## M1 0.786 0.627 0.631
##
##
## $SA23_high
## [1] "Error: None of the values in OUT is explained. Please check the truth table."
##
## $SA24_low
## [1] "Error: None of the values in OUT is explained. Please check the truth table."
##
## $SA24_high
## [1] "Error: None of the values in OUT is explained. Please check the truth table."
##
## $SA25_low
##
## M1: Demandm*Laborm + Demandm*~Materialm*~RelCommm + Laborm*~Materialm*~RelCommm
## -> SA25_low
##
## inclS PRI covS covU
## -----------------------------------------------------------
## 1 Demandm*Laborm 0.903 0.867 0.760 0.306
## 2 Demandm*~Materialm*~RelCommm 0.908 0.856 0.538 0.084
## 3 Laborm*~Materialm*~RelCommm 0.931 0.883 0.474 0.020
## -----------------------------------------------------------
## M1 0.881 0.843 0.864
##
##
## $SA25_high
##
## M1: Demandm*Laborm + Laborm*~Materialm*~RelCommm -> SA25_high
##
## inclS PRI covS covU
## ----------------------------------------------------------
## 1 Demandm*Laborm 0.737 0.610 0.835 0.313
## 2 Laborm*~Materialm*~RelCommm 0.789 0.592 0.541 0.019
## ----------------------------------------------------------
## M1 0.730 0.602 0.855

# Parsimonious solutions: Positive output including the remainders

# Result: Final QCA PARSIMONIOUS Solutions for Configurations of Reasons for MNC triggering single Outcomes of MNC (n.cut=9)

## $SA1_low
##
## M1: Demandm + Laborm -> SA1_low
##
## inclS PRI covS covU
## --------------------------------------
## 1 Demandm 0.834 0.784 0.892 0.117
## 2 Laborm 0.887 0.846 0.820 0.045
## --------------------------------------
## M1 0.826 0.777 0.937
##
##
## $SA1_high
##
## M1: Materialm + RelCommm + ~Demandm*Laborm -> SA1_high
##
## inclS PRI covS covU
## ----------------------------------------------
## 1 Materialm 0.734 0.535 0.458 0.075
## 2 RelCommm 0.795 0.624 0.583 0.153
## 3 ~Demandm*Laborm 0.823 0.509 0.266 0.042
## ----------------------------------------------
## M1 0.734 0.563 0.712
##
##
## $SA2_low
##
## M1: Materialm*~RelCommm + ~Materialm*RelCommm -> SA2_low
##
## inclS PRI covS covU
## --------------------------------------------------
## 1 Materialm*~RelCommm 0.778 0.502 0.320 0.111
## 2 ~Materialm*RelCommm 0.806 0.589 0.421 0.212
## --------------------------------------------------
## M1 0.770 0.569 0.533
##
##
## $SA2_high
## [1] "Error: None of the values in OUT is explained. Please check the truth table."
##
## $SA3_low
##
## M1: Materialm -> SA3_low
##
## inclS PRI covS covU
## ----------------------------------------
## 1 Materialm 0.769 0.614 0.478 -
## ----------------------------------------
## M1 0.769 0.614 0.478
##
##
## $SA3_high
## [1] "Error: None of the values in OUT is explained. Please check the truth table."
##
## $SA4_low
## [1] "Error: None of the values in OUT is explained. Please check the truth table."
##
## $SA4_high
## [1] "Error: None of the values in OUT is explained. Please check the truth table."
##
## $SA5_low
##
## M1: Demandm + Laborm -> SA5_low
##
## inclS PRI covS covU
## --------------------------------------
## 1 Demandm 0.703 0.584 0.917 0.097
## 2 Laborm 0.765 0.651 0.862 0.043
## --------------------------------------
## M1 0.694 0.579 0.959
##
##
## $SA5_high
## [1] "Error: None of the values in OUT is explained. Please check the truth table."
##
## $SA6_low
##
## M1: Demandm*Laborm -> SA6_low
##
## inclS PRI covS covU
## ---------------------------------------------
## 1 Demandm*Laborm 0.748 0.629 0.826 -
## ---------------------------------------------
## M1 0.748 0.629 0.826
##
##
## $SA6_high
## [1] "Error: None of the values in OUT is explained. Please check the truth table."
##
## $SA7_low
##
## M1: Materialm + RelCommm -> SA7_low
##
## inclS PRI covS covU
## ----------------------------------------
## 1 Materialm 0.792 0.648 0.451 0.097
## 2 RelCommm 0.833 0.713 0.558 0.204
## ----------------------------------------
## M1 0.795 0.673 0.655
##
##

## $SA7_high
## [1] "Error: None of the values in OUT is explained. Please check the truth table."
##
## $SA8_low
##
## M1: Demandm + Laborm -> SA8_low
##
## inclS PRI covS covU
## --------------------------------------
## 1 Demandm 0.795 0.733 0.884 0.118
## 2 Laborm 0.846 0.791 0.813 0.047
## --------------------------------------
## M1 0.791 0.730 0.931
##
##
## $SA8_high
##
## M1: Materialm + RelCommm + ~Demandm*Laborm -> SA8_high
##
## inclS PRI covS covU
## ----------------------------------------------
## 1 Materialm 0.728 0.540 0.482 0.080
## 2 RelCommm 0.765 0.580 0.596 0.148
## 3 ~Demandm*Laborm 0.799 0.485 0.274 0.041
## ----------------------------------------------
## M1 0.708 0.534 0.728
##
##
## $SA9a_low
## [1] "Error: All truth table configurations are used, all conditions are minimized. Please check the truth table."
##
## $SA9a_high
##
## M1: Demandm + Laborm -> SA9a_high
##
## inclS PRI covS covU
## --------------------------------------
## 1 Demandm 0.751 0.673 0.904 0.108
## 2 Laborm 0.812 0.742 0.845 0.049
## --------------------------------------
## M1 0.747 0.672 0.953
##
##

## $SA9b_low
## [1] "Error: All truth table configurations are used, all conditions are minimized. Please check the truth table."
##
## $SA9b_high
##
## M1: Demandm + Laborm -> SA9b_high
##
## inclS PRI covS covU
## --------------------------------------
## 1 Demandm 0.749 0.667 0.900 0.108
## 2 Laborm 0.808 0.732 0.839 0.047
## --------------------------------------
## M1 0.744 0.664 0.947
##
##
## $SA10a_low
## [1] "Error: All truth table configurations are used, all conditions are minimized. Please check the truth table."
##
## $SA10a_high
##
## M1: Demandm + Laborm -> SA10a_high
##
## inclS PRI covS covU
## --------------------------------------
## 1 Demandm 0.738 0.655 0.900 0.104
## 2 Laborm 0.798 0.721 0.841 0.045
## --------------------------------------
## M1 0.731 0.650 0.945
##
##
## $SA10b_low
## [1] "Error: All truth table configurations are used, all conditions are minimized. Please check the truth table."
##
## $SA10b_high
##
## M1: Demandm + Laborm -> SA10b_high
##
## inclS PRI covS covU
## --------------------------------------
## 1 Demandm 0.819 0.769 0.888 0.115
## 2 Laborm 0.877 0.837 0.822 0.049
## --------------------------------------
## M1 0.816 0.768 0.937
##
##

## $SA11_low
##
## M1: Materialm*~RelCommm + ~Materialm*RelCommm -> SA11_low
##
## inclS PRI covS covU
## --------------------------------------------------
## 1 Materialm*~RelCommm 0.796 0.482 0.341 0.121
## 2 ~Materialm*RelCommm 0.797 0.523 0.433 0.213
## --------------------------------------------------
## M1 0.771 0.525 0.554
##
##
## $SA11_high
## [1] "Error: None of the values in OUT is explained. Please check the truth table."
##
## $SA12_low
##
## M1: Demandm + Laborm -> SA12_low
##
## inclS PRI covS covU
## --------------------------------------
## 1 Demandm 0.701 0.591 0.914 0.097
## 2 Laborm 0.761 0.656 0.858 0.041
## --------------------------------------
## M1 0.691 0.584 0.955
##
##
## $SA12_high
## [1] "Error: None of the values in OUT is explained. Please check the truth table."
##
## $SA13_low
## [1] "Error: None of the values in OUT is explained. Please check the truth table."
##
## $SA13_high
## [1] "Error: None of the values in OUT is explained. Please check the truth table."
##
## $SA14a_low
##
## M1: Laborm -> SA14a_low
##
## inclS PRI covS covU
## -------------------------------------
## 1 Laborm 0.764 0.651 0.861 -
## -------------------------------------
## M1 0.764 0.651 0.861
##
##
## $SA14a_high
## [1] "Error: None of the values in OUT is explained. Please check the truth table."
##

## $SA14b_low
##
## M1: Demandm + Laborm -> SA14b_low
##
## inclS PRI covS covU
## --------------------------------------
## 1 Demandm 0.831 0.776 0.902 0.119
## 2 Laborm 0.881 0.835 0.827 0.044
## --------------------------------------
## M1 0.822 0.768 0.946
##
##
## $SA14b_high
##
## M1: Materialm + RelCommm -> SA14b_high
##
## inclS PRI covS covU
## ----------------------------------------
## 1 Materialm 0.751 0.582 0.481 0.093
## 2 RelCommm 0.802 0.661 0.604 0.216
## ----------------------------------------
## M1 0.752 0.608 0.697
##
##
## $SA14c_low
##
## M1: Demandm + Laborm -> SA14c_low
##
## inclS PRI covS covU
## --------------------------------------
## 1 Demandm 0.860 0.824 0.885 0.126
## 2 Laborm 0.908 0.879 0.807 0.048
## --------------------------------------
## M1 0.856 0.821 0.933
##
##
## $SA14c_high
##
## M1: Laborm -> SA14c_high
##
## inclS PRI covS covU
## -------------------------------------
## 1 Laborm 0.756 0.668 0.861 -
## -------------------------------------
## M1 0.756 0.668 0.861
##
##
## $SA15_low
## [1] "Error: None of the values in OUT is explained. Please check the truth table."
##
## $SA15_high
## [1] "Error: None of the values in OUT is explained. Please check the truth table."
##
## $SA16_low
##
## M1: Demandm + Laborm -> SA16_low
##
## inclS PRI covS covU
## --------------------------------------
## 1 Demandm 0.754 0.663 0.901 0.110
## 2 Laborm 0.809 0.725 0.836 0.045
## --------------------------------------
## M1 0.747 0.659 0.946
##
##
## $SA16_high
## [1] "Error: None of the values in OUT is explained. Please check the truth table."
##
## $SA17_low
##
## M1: Demandm + Laborm -> SA17_low
##
## inclS PRI covS covU
## --------------------------------------
## 1 Demandm 0.809 0.756 0.890 0.122
## 2 Laborm 0.853 0.805 0.811 0.043
## --------------------------------------
## M1 0.801 0.748 0.933
##
##
## $SA17_high
##
## M1: Materialm + RelCommm -> SA17_high
##
## inclS PRI covS covU
## ----------------------------------------
## 1 Materialm 0.748 0.598 0.464 0.093
## 2 RelCommm 0.781 0.641 0.571 0.199
## ----------------------------------------
## M1 0.738 0.606 0.664
##
##
## $SA18a_low
## [1] "Error: None of the values in OUT is explained. Please check the truth table."
##
## $SA18a_high
## [1] "Error: None of the values in OUT is explained. Please check the truth table."
##
## $SA18b_low
## [1] "Error: None of the values in OUT is explained. Please check the truth table."
##
## $SA18b_high
## [1] "Error: None of the values in OUT is explained. Please check the truth table."
##

## $SA19_low
##
## M1: Demandm + Laborm -> SA19_low
##
## inclS PRI covS covU
## --------------------------------------
## 1 Demandm 0.791 0.718 0.891 0.107
## 2 Laborm 0.850 0.787 0.828 0.044
## --------------------------------------
## M1 0.784 0.711 0.935
##
##
## $SA19_high
##
## M1: Materialm*~RelCommm -> SA19_high
##
## inclS PRI covS covU
## --------------------------------------------------
## 1 Materialm*~RelCommm 0.767 0.457 0.359 -
## --------------------------------------------------
## M1 0.767 0.457 0.359
##
##
## $SA20_low
##
## M1: Materialm + RelCommm -> SA20_low
##
## inclS PRI covS covU
## ----------------------------------------
## 1 Materialm 0.752 0.586 0.466 0.095
## 2 RelCommm 0.784 0.614 0.572 0.200
## ----------------------------------------
## M1 0.743 0.585 0.666
##
##
## $SA20_high
## [1] "Error: None of the values in OUT is explained. Please check the truth table."
##
## $SA21_low
##
## M1: Demandm + Laborm -> SA21_low
##
## inclS PRI covS covU
## --------------------------------------
## 1 Demandm 0.807 0.745 0.893 0.118
## 2 Laborm 0.856 0.802 0.819 0.045
## --------------------------------------
## M1 0.800 0.739 0.937
##
##

## $SA21_high
##
## M1: Materialm + RelCommm + ~Demandm*Laborm -> SA21_high
##
## inclS PRI covS covU
## ----------------------------------------------
## 1 Materialm 0.725 0.547 0.480 0.074
## 2 RelCommm 0.769 0.593 0.599 0.144
## 3 ~Demandm*Laborm 0.798 0.456 0.274 0.035
## ----------------------------------------------
## M1 0.699 0.523 0.719
##
##
## $SA22_low
##
## M1: Demandm + Laborm -> SA22_low
##
## inclS PRI covS covU
## --------------------------------------
## 1 Demandm 0.862 0.828 0.877 0.133
## 2 Laborm 0.901 0.873 0.793 0.048
## --------------------------------------
## M1 0.858 0.825 0.926
##
##
## $SA22_high
##
## M1: Laborm -> SA22_high
##
## inclS PRI covS covU
## -------------------------------------
## 1 Laborm 0.761 0.688 0.838 -
## -------------------------------------
## M1 0.761 0.688 0.838
##
##
## $SA23_low
##
## M1: Materialm + RelCommm -> SA23_low
##
## inclS PRI covS covU
## ----------------------------------------
## 1 Materialm 0.773 0.604 0.468 0.097
## 2 RelCommm 0.802 0.640 0.571 0.200
## ----------------------------------------
## M1 0.763 0.604 0.668
##
##
## $SA23_high
## [1] "Error: None of the values in OUT is explained. Please check the truth table."
##

## $SA24_low
## [1] "Error: None of the values in OUT is explained. Please check the truth table."
##
## $SA24_high
## [1] "Error: None of the values in OUT is explained. Please check the truth table."
##
## $SA25_low
##
## M1: Demandm + Laborm -> SA25_low
##
## inclS PRI covS covU
## --------------------------------------
## 1 Demandm 0.850 0.806 0.889 0.129
## 2 Laborm 0.890 0.852 0.805 0.044
## --------------------------------------
## M1 0.842 0.799 0.934
##
##
## $SA25_high
##
## M1: Laborm -> SA25_high
##
## inclS PRI covS covU
## -------------------------------------
## 1 Laborm 0.722 0.597 0.878 -
## -------------------------------------
## M1 0.722 0.597 0.878

# Intermediate solutions: Positive output including the remainders and with directional expectations

# Result: Final QCA INTERMEDIATE Solutions for Configurations of Reasons for MNC triggering single Outcomes of MNC (n.cut=9)

## $SA1_low
##
## From C1P1:
##
## M1: Demandm*Laborm + Demandm*~Materialm*~RelCommm +
## Laborm*~Materialm*~RelCommm -> SA1_low
##
## inclS PRI covS covU
## -----------------------------------------------------------
## 1 Demandm*Laborm 0.900 0.862 0.775 0.312
## 2 Demandm*~Materialm*~RelCommm 0.890 0.825 0.539 0.076
## 3 Laborm*~Materialm*~RelCommm 0.928 0.875 0.484 0.020
## -----------------------------------------------------------
## M1 0.870 0.826 0.871
##
##
## $SA1_high
##
## From C1P1:
##
## M1: Demandm*Laborm*Materialm + Demandm*Laborm*RelCommm +
## ~Demandm*Laborm*~Materialm*~RelCommm -> SA1_high
##
## inclS PRI covS covU
## -------------------------------------------------------------------
## 1 Demandm*Laborm*Materialm 0.765 0.565 0.439 0.067
## 2 Demandm*Laborm*RelCommm 0.803 0.626 0.560 0.149
## 3 ~Demandm*Laborm*~Materialm*~RelCommm 0.826 0.448 0.240 0.045
## -------------------------------------------------------------------
## M1 0.755 0.578 0.681
##
##
## $SA2_low
##
## From C1P1:
##
## M1: Demandm*Laborm*Materialm*~RelCommm + Demandm*Laborm*~Materialm*RelCommm
## -> SA2_low
##
## inclS PRI covS covU
## -----------------------------------------------------------------
## 1 Demandm*Laborm*Materialm*~RelCommm 0.803 0.529 0.309 0.102
## 2 Demandm*Laborm*~Materialm*RelCommm 0.813 0.590 0.407 0.200
## -----------------------------------------------------------------
## M1 0.788 0.587 0.509
##
##
## $SA2_high
## [1] "Error: None of the values in OUT is explained. Please check the truth table."
##
## $SA3_low
##
## From C1P1:
##
## M1: Demandm*Laborm*Materialm -> SA3_low
##
## inclS PRI covS covU
## -------------------------------------------------------
## 1 Demandm*Laborm*Materialm 0.797 0.644 0.456 -
## -------------------------------------------------------
## M1 0.797 0.644 0.456
##
##
## $SA3_high
## [1] "Error: None of the values in OUT is explained. Please check the truth table."
##
## $SA4_low
## [1] "Error: None of the values in OUT is explained. Please check the truth table."
##
## $SA4_high
## [1] "Error: None of the values in OUT is explained. Please check the truth table."
##
## $SA5_low
##
## From C1P1:
##
## M1: Demandm*Laborm + Demandm*~Materialm*~RelCommm +
## Laborm*~Materialm*~RelCommm -> SA5_low
##
## inclS PRI covS covU
## -----------------------------------------------------------
## 1 Demandm*Laborm 0.781 0.666 0.819 0.317
## 2 Demandm*~Materialm*~RelCommm 0.770 0.593 0.569 0.066
## 3 Laborm*~Materialm*~RelCommm 0.822 0.648 0.522 0.020
## -----------------------------------------------------------
## M1 0.741 0.625 0.905
##
##
## $SA5_high
## [1] "Error: None of the values in OUT is explained. Please check the truth table."
##
## $SA6_low
##
## From C1P1:
##
## M1: Demandm*Laborm -> SA6_low
##
## inclS PRI covS covU
## ---------------------------------------------
## 1 Demandm*Laborm 0.748 0.629 0.826 -
## ---------------------------------------------
## M1 0.748 0.629 0.826
##
##
## $SA6_high
## [1] "Error: None of the values in OUT is explained. Please check the truth table."
##
## $SA7_low
##
## From C1P1:
##
## M1: Demandm*Laborm*Materialm + Demandm*Laborm*RelCommm -> SA7_low
##
## inclS PRI covS covU
## -------------------------------------------------------
## 1 Demandm*Laborm*Materialm 0.821 0.683 0.430 0.082
## 2 Demandm*Laborm*RelCommm 0.845 0.727 0.537 0.189
## -------------------------------------------------------
## M1 0.820 0.701 0.619
##
##
## $SA7_high
## [1] "Error: None of the values in OUT is explained. Please check the truth table."
##
## $SA8_low
##
## From C1P1:
##
## M1: Demandm*Laborm + Demandm*~Materialm*~RelCommm +
## Laborm*~Materialm*~RelCommm -> SA8_low
##
## inclS PRI covS covU
## -----------------------------------------------------------
## 1 Demandm*Laborm 0.856 0.800 0.766 0.308
## 2 Demandm*~Materialm*~RelCommm 0.854 0.767 0.538 0.080
## 3 Laborm*~Materialm*~RelCommm 0.885 0.800 0.479 0.022
## -----------------------------------------------------------
## M1 0.833 0.776 0.868
##
##
## $SA8_high
##
## From C1P1:
##
## M1: Demandm*Laborm*Materialm + Demandm*Laborm*RelCommm +
## ~Demandm*Laborm*~Materialm*~RelCommm -> SA8_high
##
## inclS PRI covS covU
## -------------------------------------------------------------------
## 1 Demandm*Laborm*Materialm 0.758 0.569 0.461 0.067
## 2 Demandm*Laborm*RelCommm 0.772 0.581 0.571 0.143
## 3 ~Demandm*Laborm*~Materialm*~RelCommm 0.804 0.420 0.248 0.045
## -------------------------------------------------------------------
## M1 0.725 0.543 0.694
##
##

## $SA9a_low
## [1] "Error: All truth table configurations are used, all conditions are minimized. Please check the truth table."
##
## $SA9a_high
##
## From C1P1:
##
## M1: Demandm*Laborm + Demandm*~Materialm*~RelCommm +
## Laborm*~Materialm*~RelCommm -> SA9a_high
##
## inclS PRI covS covU
## -----------------------------------------------------------
## 1 Demandm*Laborm 0.822 0.751 0.797 0.318
## 2 Demandm*~Materialm*~RelCommm 0.795 0.669 0.542 0.063
## 3 Laborm*~Materialm*~RelCommm 0.850 0.732 0.499 0.020
## -----------------------------------------------------------
## M1 0.780 0.703 0.880
##
##
## $SA9b_low
## [1] "Error: All truth table configurations are used, all conditions are minimized. Please check the truth table."
##
## $SA9b_high
##
## From C1P1:
##
## M1: Demandm*Laborm + Demandm*~Materialm*~RelCommm +
## Laborm*~Materialm*~RelCommm -> SA9b_high
##
## inclS PRI covS covU
## -----------------------------------------------------------
## 1 Demandm*Laborm 0.819 0.743 0.793 0.306
## 2 Demandm*~Materialm*~RelCommm 0.811 0.692 0.552 0.066
## 3 Laborm*~Materialm*~RelCommm 0.866 0.757 0.507 0.020
## -----------------------------------------------------------
## M1 0.780 0.700 0.878
##
##
## $SA10a_low
## [1] "Error: All truth table configurations are used, all conditions are minimized. Please check the truth table."
##

## $SA10a_high
##
## From C1P1:
##
## M1: Demandm*Laborm + Demandm*~Materialm*~RelCommm +
## Laborm*~Materialm*~RelCommm -> SA10a_high
##
## inclS PRI covS covU
## -----------------------------------------------------------
## 1 Demandm*Laborm 0.811 0.734 0.796 0.309
## 2 Demandm*~Materialm*~RelCommm 0.799 0.675 0.552 0.064
## 3 Laborm*~Materialm*~RelCommm 0.852 0.737 0.506 0.019
## -----------------------------------------------------------
## M1 0.770 0.688 0.879
##
##
## $SA10b_low
## [1] "Error: All truth table configurations are used, all conditions are minimized. Please check the truth table."
##
## $SA10b_high
##
## From C1P1:
##
## M1: Demandm*Laborm + Demandm*~Materialm*~RelCommm +
## Laborm*~Materialm*~RelCommm -> SA10b_high
##
## inclS PRI covS covU
## -----------------------------------------------------------
## 1 Demandm*Laborm 0.885 0.845 0.772 0.313
## 2 Demandm*~Materialm*~RelCommm 0.856 0.780 0.526 0.066
## 3 Laborm*~Materialm*~RelCommm 0.913 0.856 0.482 0.023
## -----------------------------------------------------------
## M1 0.848 0.801 0.861
##
##
## $SA11_low
##
## From C1P1:
##
## M1: Demandm*Laborm*Materialm*~RelCommm + Demandm*Laborm*~Materialm*RelCommm
## -> SA11_low
##
## inclS PRI covS covU
## -----------------------------------------------------------------
## 1 Demandm*Laborm*Materialm*~RelCommm 0.820 0.508 0.328 0.112
## 2 Demandm*Laborm*~Materialm*RelCommm 0.806 0.524 0.418 0.203
## -----------------------------------------------------------------
## M1 0.791 0.543 0.531
##
##
## $SA11_high
## [1] "Error: None of the values in OUT is explained. Please check the truth table."
##

## $SA12_low
##
## From C1P1:
##
## M1: Demandm*Laborm + Demandm*~Materialm*~RelCommm +
## Laborm*~Materialm*~RelCommm -> SA12_low
##
## inclS PRI covS covU
## -----------------------------------------------------------
## 1 Demandm*Laborm 0.778 0.673 0.817 0.317
## 2 Demandm*~Materialm*~RelCommm 0.769 0.604 0.568 0.068
## 3 Laborm*~Materialm*~RelCommm 0.817 0.650 0.519 0.019
## -----------------------------------------------------------
## M1 0.740 0.634 0.904
##
##
## $SA12_high
## [1] "Error: None of the values in OUT is explained. Please check the truth table."
##
## $SA13_low
## [1] "Error: None of the values in OUT is explained. Please check the truth table."
##
## $SA13_high
## [1] "Error: None of the values in OUT is explained. Please check the truth table."
##
## $SA14a_low
##
## From C1P1:
##
## M1: Demandm*Laborm + Laborm*~Materialm*~RelCommm -> SA14a_low
##
## inclS PRI covS covU
## ----------------------------------------------------------
## 1 Demandm*Laborm 0.783 0.671 0.822 0.325
## 2 Laborm*~Materialm*~RelCommm 0.810 0.629 0.515 0.018
## ----------------------------------------------------------
## M1 0.775 0.661 0.840
##
##
## $SA14a_high
## [1] "Error: None of the values in OUT is explained. Please check the truth table."
##

## $SA14b_low
##
## From C1P1:
##
## M1: Demandm*Laborm + Demandm*~Materialm*~RelCommm +
## Laborm*~Materialm*~RelCommm -> SA14b_low
##
## inclS PRI covS covU
## -----------------------------------------------------------
## 1 Demandm*Laborm 0.896 0.853 0.784 0.320
## 2 Demandm*~Materialm*~RelCommm 0.869 0.786 0.535 0.071
## 3 Laborm*~Materialm*~RelCommm 0.911 0.840 0.482 0.019
## -----------------------------------------------------------
## M1 0.859 0.807 0.873
##
##
## $SA14b_high
##
## From C1P1:
##
## M1: Demandm*Laborm*Materialm + Demandm*Laborm*RelCommm -> SA14b_high
##
## inclS PRI covS covU
## -------------------------------------------------------
## 1 Demandm*Laborm*Materialm 0.781 0.614 0.461 0.078
## 2 Demandm*Laborm*RelCommm 0.811 0.667 0.580 0.198
## -------------------------------------------------------
## M1 0.775 0.629 0.659
##
##
## $SA14c_low
##
## From C1P1:
##
## M1: Demandm*Laborm + Demandm*~Materialm*~RelCommm +
## Laborm*~Materialm*~RelCommm -> SA14c_low
##
## inclS PRI covS covU
## -----------------------------------------------------------
## 1 Demandm*Laborm 0.916 0.889 0.759 0.313
## 2 Demandm*~Materialm*~RelCommm 0.893 0.840 0.520 0.075
## 3 Laborm*~Materialm*~RelCommm 0.930 0.887 0.466 0.020
## -----------------------------------------------------------
## M1 0.886 0.853 0.854
##
##

## $SA14c_high
##
## From C1P1:
##
## M1: Demandm*Laborm + Laborm*~Materialm*~RelCommm -> SA14c_high
##
## inclS PRI covS covU
## ----------------------------------------------------------
## 1 Demandm*Laborm 0.770 0.680 0.816 0.321
## 2 Laborm*~Materialm*~RelCommm 0.798 0.654 0.512 0.017
## ----------------------------------------------------------
## M1 0.761 0.671 0.833
##
##
## $SA15_low
## [1] "Error: None of the values in OUT is explained. Please check the truth table."
##
## $SA15_high
## [1] "Error: None of the values in OUT is explained. Please check the truth table."
##
## $SA16_low
##
## From C1P1:
##
## M1: Demandm*Laborm + Demandm*~Materialm*~RelCommm +
## Laborm*~Materialm*~RelCommm -> SA16_low
##
## inclS PRI covS covU
## -----------------------------------------------------------
## 1 Demandm*Laborm 0.822 0.737 0.791 0.311
## 2 Demandm*~Materialm*~RelCommm 0.824 0.700 0.558 0.078
## 3 Laborm*~Materialm*~RelCommm 0.854 0.725 0.497 0.018
## -----------------------------------------------------------
## M1 0.792 0.706 0.887
##
##
## $SA16_high
## [1] "Error: None of the values in OUT is explained. Please check the truth table."
##
## $SA17_low
##
## From C1P1:
##
## M1: Demandm*Laborm + Demandm*~Materialm*~RelCommm +
## Laborm*~Materialm*~RelCommm -> SA17_low
##
## inclS PRI covS covU
## -----------------------------------------------------------
## 1 Demandm*Laborm 0.867 0.821 0.768 0.314
## 2 Demandm*~Materialm*~RelCommm 0.852 0.772 0.531 0.076
## 3 Laborm*~Materialm*~RelCommm 0.881 0.803 0.472 0.018
## -----------------------------------------------------------
## M1 0.837 0.787 0.862
##
##
## $SA17_high
##
## From C1P1:
##
## M1: Demandm*Laborm*Materialm + Demandm*Laborm*RelCommm -> SA17_high
##
## inclS PRI covS covU
## -------------------------------------------------------
## 1 Demandm*Laborm*Materialm 0.779 0.632 0.446 0.079
## 2 Demandm*Laborm*RelCommm 0.788 0.643 0.547 0.181
## -------------------------------------------------------
## M1 0.760 0.624 0.627
##
##
## $SA18a_low
## [1] "Error: None of the values in OUT is explained. Please check the truth table."
##
## $SA18a_high
## [1] "Error: None of the values in OUT is explained. Please check the truth table."
##
## $SA18b_low
## [1] "Error: None of the values in OUT is explained. Please check the truth table."
##
## $SA18b_high
## [1] "Error: None of the values in OUT is explained. Please check the truth table."
##
## $SA19_low
##
## From C1P1:
##
## M1: Demandm*Laborm + Demandm*~Materialm*~RelCommm +
## Laborm*~Materialm*~RelCommm -> SA19_low
##
## inclS PRI covS covU
## -----------------------------------------------------------
## 1 Demandm*Laborm 0.864 0.803 0.784 0.310
## 2 Demandm*~Materialm*~RelCommm 0.852 0.754 0.544 0.071
## 3 Laborm*~Materialm*~RelCommm 0.898 0.813 0.493 0.020
## -----------------------------------------------------------
## M1 0.828 0.761 0.874
##
##

## $SA19_high
##
## From C1P1:
##
## M1: Demandm*Laborm*Materialm*~RelCommm -> SA19_high
##
## inclS PRI covS covU
## -----------------------------------------------------------------
## 1 Demandm*Laborm*Materialm*~RelCommm 0.800 0.494 0.350 -
## -----------------------------------------------------------------
## M1 0.800 0.494 0.350
##
##
## $SA20_low
##
## From C1P1:
##
## M1: Demandm*Laborm*Materialm + Demandm*Laborm*RelCommm -> SA20_low
##
## inclS PRI covS covU
## -------------------------------------------------------
## 1 Demandm*Laborm*Materialm 0.781 0.617 0.445 0.080
## 2 Demandm*Laborm*RelCommm 0.797 0.627 0.552 0.187
## -------------------------------------------------------
## M1 0.769 0.610 0.633
##
##
## $SA20_high
## [1] "Error: None of the values in OUT is explained. Please check the truth table."
##
## $SA21_low
##
## From C1P1:
##
## M1: Demandm*Laborm + Demandm*~Materialm*~RelCommm +
## Laborm*~Materialm*~RelCommm -> SA21_low
##
## inclS PRI covS covU
## -----------------------------------------------------------
## 1 Demandm*Laborm 0.869 0.817 0.774 0.311
## 2 Demandm*~Materialm*~RelCommm 0.863 0.780 0.541 0.078
## 3 Laborm*~Materialm*~RelCommm 0.892 0.812 0.481 0.018
## -----------------------------------------------------------
## M1 0.839 0.782 0.870
##
##

## $SA21_high
##
## From C1P1:
##
## M1: Demandm*Laborm*Materialm + Demandm*Laborm*RelCommm +
## ~Demandm*Laborm*~Materialm*~RelCommm -> SA21_high
##
## inclS PRI covS covU
## -------------------------------------------------------------------
## 1 Demandm*Laborm*Materialm 0.759 0.582 0.462 0.065
## 2 Demandm*Laborm*RelCommm 0.778 0.597 0.575 0.140
## 3 ~Demandm*Laborm*~Materialm*~RelCommm 0.803 0.387 0.247 0.039
## -------------------------------------------------------------------
## M1 0.720 0.537 0.689
##
##
## $SA22_low
##
## From C1P1:
##
## M1: Demandm*Laborm + Demandm*~Materialm*~RelCommm +
## Laborm*~Materialm*~RelCommm -> SA22_low
##
## inclS PRI covS covU
## -----------------------------------------------------------
## 1 Demandm*Laborm 0.909 0.881 0.745 0.302
## 2 Demandm*~Materialm*~RelCommm 0.903 0.858 0.521 0.078
## 3 Laborm*~Materialm*~RelCommm 0.931 0.892 0.462 0.019
## -----------------------------------------------------------
## M1 0.882 0.850 0.841
##
##
## $SA22_high
##
## From C1P1:
##
## M1: Demandm*Laborm + Laborm*~Materialm*~RelCommm -> SA22_high
##
## inclS PRI covS covU
## ----------------------------------------------------------
## 1 Demandm*Laborm 0.770 0.695 0.790 0.314
## 2 Laborm*~Materialm*~RelCommm 0.794 0.669 0.493 0.016
## ----------------------------------------------------------
## M1 0.761 0.685 0.806
##
##

## $SA23_low
##
## From C1P1:
##
## M1: Demandm*Laborm*Materialm + Demandm*Laborm*RelCommm -> SA23_low
##
## inclS PRI covS covU
## -------------------------------------------------------
## 1 Demandm*Laborm*Materialm 0.801 0.635 0.446 0.083
## 2 Demandm*Laborm*RelCommm 0.811 0.646 0.548 0.185
## -------------------------------------------------------
## M1 0.786 0.627 0.631
##
##
## $SA23_high
## [1] "Error: None of the values in OUT is explained. Please check the truth table."
##
## $SA24_low
## [1] "Error: None of the values in OUT is explained. Please check the truth table."
##
## $SA24_high
## [1] "Error: None of the values in OUT is explained. Please check the truth table."
##
## $SA25_low
##
## From C1P1:
##
## M1: Demandm*Laborm + Demandm*~Materialm*~RelCommm +
## Laborm*~Materialm*~RelCommm -> SA25_low
##
## inclS PRI covS covU
## -----------------------------------------------------------
## 1 Demandm*Laborm 0.903 0.867 0.760 0.306
## 2 Demandm*~Materialm*~RelCommm 0.908 0.856 0.538 0.084
## 3 Laborm*~Materialm*~RelCommm 0.931 0.883 0.474 0.020
## -----------------------------------------------------------
## M1 0.881 0.843 0.864
##
##
## $SA25_high
##
## From C1P1:
##
## M1: Demandm*Laborm + Laborm*~Materialm*~RelCommm -> SA25_high
##
## inclS PRI covS covU
## ----------------------------------------------------------
## 1 Demandm*Laborm 0.737 0.610 0.835 0.313
## 2 Laborm*~Materialm*~RelCommm 0.789 0.592 0.541 0.019
## ----------------------------------------------------------
## M1 0.730 0.602 0.855

#### FOR n.cut (minimum number of cases with membership > 0.5 for an outcome) -> n15:n.cut=15
# Complex solutions: Positive output

# Result: Final QCA COMPLEX Solutions for Configurations of Reasons for MNC triggering single Outcomes of MNC (n.cut=15)

## $SA1_low
##
## M1: Demandm*Laborm + Demandm*~Materialm*~RelCommm -> SA1_low
##
## inclS PRI covS covU
## -----------------------------------------------------------
## 1 Demandm*Laborm 0.900 0.862 0.775 0.312
## 2 Demandm*~Materialm*~RelCommm 0.890 0.825 0.539 0.076
## -----------------------------------------------------------
## M1 0.874 0.831 0.851
##
##
## $SA1_high
##
## M1: Demandm*Laborm*Materialm + Demandm*Laborm*RelCommm -> SA1_high
##
## inclS PRI covS covU
## -------------------------------------------------------
## 1 Demandm*Laborm*Materialm 0.765 0.565 0.439 0.076
## 2 Demandm*Laborm*RelCommm 0.803 0.626 0.560 0.196
## -------------------------------------------------------
## M1 0.768 0.597 0.636
##
##
## $SA2_low
##
## M1: Demandm*Laborm*Materialm*~RelCommm + Demandm*Laborm*~Materialm*RelCommm
## -> SA2_low
##
## inclS PRI covS covU
## -----------------------------------------------------------------
## 1 Demandm*Laborm*Materialm*~RelCommm 0.803 0.529 0.309 0.102
## 2 Demandm*Laborm*~Materialm*RelCommm 0.813 0.590 0.407 0.200
## -----------------------------------------------------------------
## M1 0.788 0.587 0.509
##
##
## $SA2_high
## [1] "Error: None of the values in OUT is explained. Please check the truth table."
##

## $SA3_low
##
## M1: Demandm*Laborm*Materialm -> SA3_low
##
## inclS PRI covS covU
## -------------------------------------------------------
## 1 Demandm*Laborm*Materialm 0.797 0.644 0.456 -
## -------------------------------------------------------
## M1 0.797 0.644 0.456
##
##
## $SA3_high
## [1] "Error: None of the values in OUT is explained. Please check the truth table."
##
## $SA4_low
## [1] "Error: None of the values in OUT is explained. Please check the truth table."
##
## $SA4_high
## [1] "Error: None of the values in OUT is explained. Please check the truth table."
##
## $SA5_low
##
## M1: Demandm*Laborm + Demandm*~Materialm*~RelCommm -> SA5_low
##
## inclS PRI covS covU
## -----------------------------------------------------------
## 1 Demandm*Laborm 0.781 0.666 0.819 0.317
## 2 Demandm*~Materialm*~RelCommm 0.770 0.593 0.569 0.066
## -----------------------------------------------------------
## M1 0.746 0.630 0.885
##
##
## $SA5_high
## [1] "Error: None of the values in OUT is explained. Please check the truth table."
##
## $SA6_low
##
## M1: Demandm*Laborm -> SA6_low
##
## inclS PRI covS covU
## ---------------------------------------------
## 1 Demandm*Laborm 0.748 0.629 0.826 -
## ---------------------------------------------
## M1 0.748 0.629 0.826
##
##
## $SA6_high
## [1] "Error: None of the values in OUT is explained. Please check the truth table."
##

## $SA7_low
##
## M1: Demandm*Laborm*Materialm + Demandm*Laborm*RelCommm -> SA7_low
##
## inclS PRI covS covU
## -------------------------------------------------------
## 1 Demandm*Laborm*Materialm 0.821 0.683 0.430 0.082
## 2 Demandm*Laborm*RelCommm 0.845 0.727 0.537 0.189
## -------------------------------------------------------
## M1 0.820 0.701 0.619
##
##
## $SA7_high
## [1] "Error: None of the values in OUT is explained. Please check the truth table."
##
## $SA8_low
##
## M1: Demandm*Laborm + Demandm*~Materialm*~RelCommm -> SA8_low
##
## inclS PRI covS covU
## -----------------------------------------------------------
## 1 Demandm*Laborm 0.856 0.800 0.766 0.308
## 2 Demandm*~Materialm*~RelCommm 0.854 0.767 0.538 0.080
## -----------------------------------------------------------
## M1 0.836 0.779 0.846
##
##
## $SA8_high
##
## M1: Demandm*Laborm*Materialm + Demandm*Laborm*RelCommm -> SA8_high
##
## inclS PRI covS covU
## -------------------------------------------------------
## 1 Demandm*Laborm*Materialm 0.758 0.569 0.461 0.078
## 2 Demandm*Laborm*RelCommm 0.772 0.581 0.571 0.188
## -------------------------------------------------------
## M1 0.739 0.561 0.649
##
##

## $SA9a_low
##
## M1: Demandm*Laborm + ~Laborm*~Materialm*~RelCommm -> SA9a_low
##
## inclS PRI covS covU
## -----------------------------------------------------------
## 1 Demandm*Laborm 0.934 0.914 0.733 0.476
## 2 ~Laborm*~Materialm*~RelCommm 0.845 0.742 0.348 0.091
## -----------------------------------------------------------
## M1 0.885 0.854 0.824
##
##
## $SA9a_high
##
## M1: Demandm*Laborm + Demandm*~Materialm*~RelCommm -> SA9a_high
##
## inclS PRI covS covU
## -----------------------------------------------------------
## 1 Demandm*Laborm 0.822 0.751 0.797 0.318
## 2 Demandm*~Materialm*~RelCommm 0.795 0.669 0.542 0.063
## -----------------------------------------------------------
## M1 0.785 0.708 0.860
##
##
## $SA9b_low
##
## M1: Demandm*Laborm + ~Laborm*~Materialm*~RelCommm -> SA9b_low
##
## inclS PRI covS covU
## -----------------------------------------------------------
## 1 Demandm*Laborm 0.931 0.912 0.732 0.474
## 2 ~Laborm*~Materialm*~RelCommm 0.848 0.748 0.349 0.092
## -----------------------------------------------------------
## M1 0.884 0.854 0.824
##
##
## $SA9b_high
##
## M1: Demandm*Laborm + Demandm*~Materialm*~RelCommm -> SA9b_high
##
## inclS PRI covS covU
## -----------------------------------------------------------
## 1 Demandm*Laborm 0.819 0.743 0.793 0.306
## 2 Demandm*~Materialm*~RelCommm 0.811 0.692 0.552 0.066
## -----------------------------------------------------------
## M1 0.784 0.705 0.858
##
##

## $SA10a_low
##
## M1: Demandm*Laborm + ~Laborm*~Materialm*~RelCommm -> SA10a_low
##
## inclS PRI covS covU
## -----------------------------------------------------------
## 1 Demandm*Laborm 0.934 0.914 0.735 0.479
## 2 ~Laborm*~Materialm*~RelCommm 0.850 0.752 0.351 0.095
## -----------------------------------------------------------
## M1 0.889 0.859 0.830
##
##
## $SA10a_high
##
## M1: Demandm*Laborm + Demandm*~Materialm*~RelCommm -> SA10a_high
##
## inclS PRI covS covU
## -----------------------------------------------------------
## 1 Demandm*Laborm 0.811 0.734 0.796 0.309
## 2 Demandm*~Materialm*~RelCommm 0.799 0.675 0.552 0.064
## -----------------------------------------------------------
## M1 0.775 0.694 0.861
##
##
## $SA10b_low
##
## M1: Demandm*Laborm + ~Laborm*~Materialm*~RelCommm -> SA10b_low
##
## inclS PRI covS covU
## -----------------------------------------------------------
## 1 Demandm*Laborm 0.961 0.952 0.719 0.471
## 2 ~Laborm*~Materialm*~RelCommm 0.869 0.797 0.341 0.093
## -----------------------------------------------------------
## M1 0.916 0.898 0.812
##
##
## $SA10b_high
##
## M1: Demandm*Laborm + Demandm*~Materialm*~RelCommm -> SA10b_high
##
## inclS PRI covS covU
## -----------------------------------------------------------
## 1 Demandm*Laborm 0.885 0.845 0.772 0.313
## 2 Demandm*~Materialm*~RelCommm 0.856 0.780 0.526 0.066
## -----------------------------------------------------------
## M1 0.849 0.803 0.838
##
##

## $SA11_low
##
## M1: Demandm*Laborm*Materialm*~RelCommm + Demandm*Laborm*~Materialm*RelCommm
## -> SA11_low
##
## inclS PRI covS covU
## -----------------------------------------------------------------
## 1 Demandm*Laborm*Materialm*~RelCommm 0.820 0.508 0.328 0.112
## 2 Demandm*Laborm*~Materialm*RelCommm 0.806 0.524 0.418 0.203
## -----------------------------------------------------------------
## M1 0.791 0.543 0.531
##
##
## $SA11_high
## [1] "Error: None of the values in OUT is explained. Please check the truth table."
##
## $SA12_low
##
## M1: Demandm*Laborm + Demandm*~Materialm*~RelCommm -> SA12_low
##
## inclS PRI covS covU
## -----------------------------------------------------------
## 1 Demandm*Laborm 0.778 0.673 0.817 0.317
## 2 Demandm*~Materialm*~RelCommm 0.769 0.604 0.568 0.068
## -----------------------------------------------------------
## M1 0.746 0.639 0.885
##
##
## $SA12_high
## [1] "Error: None of the values in OUT is explained. Please check the truth table."
##
## $SA13_low
## [1] "Error: None of the values in OUT is explained. Please check the truth table."
##
## $SA13_high
## [1] "Error: None of the values in OUT is explained. Please check the truth table."
##

## $SA14a_low
##
## M1: Demandm*Laborm -> SA14a_low
##
## inclS PRI covS covU
## ---------------------------------------------
## 1 Demandm*Laborm 0.783 0.671 0.822 -
## ---------------------------------------------
## M1 0.783 0.671 0.822
##
##
## $SA14a_high
## [1] "Error: None of the values in OUT is explained. Please check the truth table."
##
## $SA14b_low
##
## M1: Demandm*Laborm + Demandm*~Materialm*~RelCommm -> SA14b_low
##
## inclS PRI covS covU
## -----------------------------------------------------------
## 1 Demandm*Laborm 0.896 0.853 0.784 0.320
## 2 Demandm*~Materialm*~RelCommm 0.869 0.786 0.535 0.071
## -----------------------------------------------------------
## M1 0.865 0.815 0.855
##
##
## $SA14b_high
##
## M1: Demandm*Laborm*Materialm + Demandm*Laborm*RelCommm -> SA14b_high
##
## inclS PRI covS covU
## -------------------------------------------------------
## 1 Demandm*Laborm*Materialm 0.781 0.614 0.461 0.078
## 2 Demandm*Laborm*RelCommm 0.811 0.667 0.580 0.198
## -------------------------------------------------------
## M1 0.775 0.629 0.659
##
##

## $SA14c_low
##
## M1: Demandm*Laborm + Demandm*~Materialm*~RelCommm -> SA14c_low
##
## inclS PRI covS covU
## -----------------------------------------------------------
## 1 Demandm*Laborm 0.916 0.889 0.759 0.313
## 2 Demandm*~Materialm*~RelCommm 0.893 0.840 0.520 0.075
## -----------------------------------------------------------
## M1 0.890 0.858 0.833
##
##
## $SA14c_high
##
## M1: Demandm*Laborm -> SA14c_high
##
## inclS PRI covS covU
## ---------------------------------------------
## 1 Demandm*Laborm 0.770 0.680 0.816 -
## ---------------------------------------------
## M1 0.770 0.680 0.816
##
##
## $SA15_low
## [1] "Error: None of the values in OUT is explained. Please check the truth table."
##
## $SA15_high
## [1] "Error: None of the values in OUT is explained. Please check the truth table."
##
## $SA16_low
##
## M1: Demandm*Laborm + Demandm*~Materialm*~RelCommm -> SA16_low
##
## inclS PRI covS covU
## -----------------------------------------------------------
## 1 Demandm*Laborm 0.822 0.737 0.791 0.311
## 2 Demandm*~Materialm*~RelCommm 0.824 0.700 0.558 0.078
## -----------------------------------------------------------
## M1 0.799 0.714 0.869
##
##
## $SA16_high
## [1] "Error: None of the values in OUT is explained. Please check the truth table."
##

## $SA17_low
##
## M1: Demandm*Laborm + Demandm*~Materialm*~RelCommm -> SA17_low
##
## inclS PRI covS covU
## -----------------------------------------------------------
## 1 Demandm*Laborm 0.867 0.821 0.768 0.314
## 2 Demandm*~Materialm*~RelCommm 0.852 0.772 0.531 0.076
## -----------------------------------------------------------
## M1 0.843 0.794 0.844
##
##
## $SA17_high
##
## M1: Demandm*Laborm*Materialm + Demandm*Laborm*RelCommm -> SA17_high
##
## inclS PRI covS covU
## -------------------------------------------------------
## 1 Demandm*Laborm*Materialm 0.779 0.632 0.446 0.079
## 2 Demandm*Laborm*RelCommm 0.788 0.643 0.547 0.181
## -------------------------------------------------------
## M1 0.760 0.624 0.627
##
##
## $SA18a_low
## [1] "Error: None of the values in OUT is explained. Please check the truth table."
##
## $SA18a_high
## [1] "Error: None of the values in OUT is explained. Please check the truth table."
##
## $SA18b_low
## [1] "Error: None of the values in OUT is explained. Please check the truth table."
##
## $SA18b_high
## [1] "Error: None of the values in OUT is explained. Please check the truth table."
##

## $SA19_low
##
## M1: Demandm*Laborm + Demandm*~Materialm*~RelCommm -> SA19_low
##
## inclS PRI covS covU
## -----------------------------------------------------------
## 1 Demandm*Laborm 0.864 0.803 0.784 0.310
## 2 Demandm*~Materialm*~RelCommm 0.852 0.754 0.544 0.071
## -----------------------------------------------------------
## M1 0.833 0.766 0.854
##
##
## $SA19_high
##
## M1: Demandm*Laborm*Materialm*~RelCommm -> SA19_high
##
## inclS PRI covS covU
## -----------------------------------------------------------------
## 1 Demandm*Laborm*Materialm*~RelCommm 0.800 0.494 0.350 -
## -----------------------------------------------------------------
## M1 0.800 0.494 0.350
##
##
## $SA20_low
##
## M1: Demandm*Laborm*Materialm + Demandm*Laborm*RelCommm -> SA20_low
##
## inclS PRI covS covU
## -------------------------------------------------------
## 1 Demandm*Laborm*Materialm 0.781 0.617 0.445 0.080
## 2 Demandm*Laborm*RelCommm 0.797 0.627 0.552 0.187
## -------------------------------------------------------
## M1 0.769 0.610 0.633
##
##
## $SA20_high
## [1] "Error: None of the values in OUT is explained. Please check the truth table."
##

## $SA21_low
##
## M1: Demandm*Laborm + Demandm*~Materialm*~RelCommm -> SA21_low
##
## inclS PRI covS covU
## -----------------------------------------------------------
## 1 Demandm*Laborm 0.869 0.817 0.774 0.311
## 2 Demandm*~Materialm*~RelCommm 0.863 0.780 0.541 0.078
## -----------------------------------------------------------
## M1 0.846 0.790 0.852
##
##
## $SA21_high
##
## M1: Demandm*Laborm*Materialm + Demandm*Laborm*RelCommm -> SA21_high
##
## inclS PRI covS covU
## -------------------------------------------------------
## 1 Demandm*Laborm*Materialm 0.759 0.582 0.462 0.075
## 2 Demandm*Laborm*RelCommm 0.778 0.597 0.575 0.188
## -------------------------------------------------------
## M1 0.740 0.568 0.650
##
##
## $SA22_low
##
## M1: Demandm*Laborm + Demandm*~Materialm*~RelCommm -> SA22_low
##
## inclS PRI covS covU
## -----------------------------------------------------------
## 1 Demandm*Laborm 0.909 0.881 0.745 0.302
## 2 Demandm*~Materialm*~RelCommm 0.903 0.858 0.521 0.078
## -----------------------------------------------------------
## M1 0.887 0.856 0.822
##
##
## $SA22_high
##
## M1: Demandm*Laborm -> SA22_high
##
## inclS PRI covS covU
## ---------------------------------------------
## 1 Demandm*Laborm 0.770 0.695 0.790 -
## ---------------------------------------------
## M1 0.770 0.695 0.790
##
##

## $SA23_low
##
## M1: Demandm*Laborm*Materialm + Demandm*Laborm*RelCommm -> SA23_low
##
## inclS PRI covS covU
## -------------------------------------------------------
## 1 Demandm*Laborm*Materialm 0.801 0.635 0.446 0.083
## 2 Demandm*Laborm*RelCommm 0.811 0.646 0.548 0.185
## -------------------------------------------------------
## M1 0.786 0.627 0.631
##
##
## $SA23_high
## [1] "Error: None of the values in OUT is explained. Please check the truth table."
##
## $SA24_low
## [1] "Error: None of the values in OUT is explained. Please check the truth table."
##
## $SA24_high
## [1] "Error: None of the values in OUT is explained. Please check the truth table."
##
## $SA25_low
##
## M1: Demandm*Laborm + Demandm*~Materialm*~RelCommm -> SA25_low
##
## inclS PRI covS covU
## -----------------------------------------------------------
## 1 Demandm*Laborm 0.903 0.867 0.760 0.306
## 2 Demandm*~Materialm*~RelCommm 0.908 0.856 0.538 0.084
## -----------------------------------------------------------
## M1 0.886 0.849 0.844
##
##
## $SA25_high
##
## M1: Demandm*Laborm -> SA25_high
##
## inclS PRI covS covU
## ---------------------------------------------
## 1 Demandm*Laborm 0.737 0.610 0.835 -
## ---------------------------------------------
## M1 0.737 0.610 0.835

# Parsimonious solutions: Positive output including the remainders

# Result: Final QCA PARSIMONIOUS Solutions for Configurations of Reasons for MNC triggering single Outcomes of MNC (n.cut=15)

## $SA1_low
##
## M1: Demandm -> SA1_low
##
## inclS PRI covS covU
## --------------------------------------
## 1 Demandm 0.834 0.784 0.892 -
## --------------------------------------
## M1 0.834 0.784 0.892
##
##
## $SA1_high
##
## M1: Materialm + RelCommm -> SA1_high
##
## inclS PRI covS covU
## ----------------------------------------
## 1 Materialm 0.734 0.535 0.458 0.087
## 2 RelCommm 0.795 0.624 0.583 0.212
## ----------------------------------------
## M1 0.743 0.575 0.670
##
##
## $SA2_low
##
## M1: Materialm*~RelCommm + ~Materialm*RelCommm -> SA2_low
##
## inclS PRI covS covU
## --------------------------------------------------
## 1 Materialm*~RelCommm 0.778 0.502 0.320 0.111
## 2 ~Materialm*RelCommm 0.806 0.589 0.421 0.212
## --------------------------------------------------
## M1 0.770 0.569 0.533
##
##
## $SA2_high
## [1] "Error: None of the values in OUT is explained. Please check the truth table."
##

## $SA3_low
##
## M1: Materialm -> SA3_low
##
## inclS PRI covS covU
## ----------------------------------------
## 1 Materialm 0.769 0.614 0.478 -
## ----------------------------------------
## M1 0.769 0.614 0.478
##
##
## $SA3_high
## [1] "Error: None of the values in OUT is explained. Please check the truth table."
##
## $SA4_low
## [1] "Error: None of the values in OUT is explained. Please check the truth table."
##
## $SA4_high
## [1] "Error: None of the values in OUT is explained. Please check the truth table."
##
## $SA5_low
##
## M1: Demandm -> SA5_low
##
## inclS PRI covS covU
## --------------------------------------
## 1 Demandm 0.703 0.584 0.917 -
## --------------------------------------
## M1 0.703 0.584 0.917
##
##
## $SA5_high
## [1] "Error: None of the values in OUT is explained. Please check the truth table."
##
## $SA6_low
##
## M1: Laborm -> SA6_low
##
## inclS PRI covS covU
## -------------------------------------
## 1 Laborm 0.732 0.615 0.867 -
## -------------------------------------
## M1 0.732 0.615 0.867
##
##
## $SA6_high
## [1] "Error: None of the values in OUT is explained. Please check the truth table."
##

## $SA7_low
##
## M1: Materialm + RelCommm -> SA7_low
##
## inclS PRI covS covU
## ----------------------------------------
## 1 Materialm 0.792 0.648 0.451 0.097
## 2 RelCommm 0.833 0.713 0.558 0.204
## ----------------------------------------
## M1 0.795 0.673 0.655
##
##
## $SA7_high
## [1] "Error: None of the values in OUT is explained. Please check the truth table."
##
## $SA8_low
##
## M1: Demandm -> SA8_low
##
## inclS PRI covS covU
## --------------------------------------
## 1 Demandm 0.795 0.733 0.884 -
## --------------------------------------
## M1 0.795 0.733 0.884
##
##
## $SA8_high
##
## M1: Materialm + RelCommm -> SA8_high
##
## inclS PRI covS covU
## ----------------------------------------
## 1 Materialm 0.728 0.540 0.482 0.092
## 2 RelCommm 0.765 0.580 0.596 0.206
## ----------------------------------------
## M1 0.718 0.546 0.688
##
##
## $SA9a_low
## [1] "Error: All truth table configurations are used, all conditions are minimized. Please check the truth table."
##
## $SA9a_high
##
## M1: Demandm -> SA9a_high
##
## inclS PRI covS covU
## --------------------------------------
## 1 Demandm 0.751 0.673 0.904 -
## --------------------------------------
## M1 0.751 0.673 0.904
##
##

## $SA9b_low
## [1] "Error: All truth table configurations are used, all conditions are minimized. Please check the truth table."
##
## $SA9b_high
##
## M1: Demandm -> SA9b_high
##
## inclS PRI covS covU
## --------------------------------------
## 1 Demandm 0.749 0.667 0.900 -
## --------------------------------------
## M1 0.749 0.667 0.900
##
##
## $SA10a_low
## [1] "Error: All truth table configurations are used, all conditions are minimized. Please check the truth table."
##
## $SA10a_high
##
## M1: Demandm -> SA10a_high
##
## inclS PRI covS covU
## --------------------------------------
## 1 Demandm 0.738 0.655 0.900 -
## --------------------------------------
## M1 0.738 0.655 0.900
##
##
## $SA10b_low
## [1] "Error: All truth table configurations are used, all conditions are minimized. Please check the truth table."
##
## $SA10b_high
##
## M1: Demandm -> SA10b_high
##
## inclS PRI covS covU
## --------------------------------------
## 1 Demandm 0.819 0.769 0.888 -
## --------------------------------------
## M1 0.819 0.769 0.888
##
##

## $SA11_low
##
## M1: Materialm*~RelCommm + ~Materialm*RelCommm -> SA11_low
##
## inclS PRI covS covU
## --------------------------------------------------
## 1 Materialm*~RelCommm 0.796 0.482 0.341 0.121
## 2 ~Materialm*RelCommm 0.797 0.523 0.433 0.213
## --------------------------------------------------
## M1 0.771 0.525 0.554
##
##
## $SA11_high
## [1] "Error: None of the values in OUT is explained. Please check the truth table."
##
## $SA12_low
##
## M1: Demandm -> SA12_low
##
## inclS PRI covS covU
## --------------------------------------
## 1 Demandm 0.701 0.591 0.914 -
## --------------------------------------
## M1 0.701 0.591 0.914
##
##
## $SA12_high
## [1] "Error: None of the values in OUT is explained. Please check the truth table."
##
## $SA13_low
## [1] "Error: None of the values in OUT is explained. Please check the truth table."
##
## $SA13_high
## [1] "Error: None of the values in OUT is explained. Please check the truth table."
##
## $SA14a_low
##
## M1: Laborm -> SA14a_low
##
## inclS PRI covS covU
## -------------------------------------
## 1 Laborm 0.764 0.651 0.861 -
## -------------------------------------
## M1 0.764 0.651 0.861
##
##
## $SA14a_high
## [1] "Error: None of the values in OUT is explained. Please check the truth table."
##

## $SA14b_low
##
## M1: Demandm -> SA14b_low
##
## inclS PRI covS covU
## --------------------------------------
## 1 Demandm 0.831 0.776 0.902 -
## --------------------------------------
## M1 0.831 0.776 0.902
##
##
## $SA14b_high
##
## M1: Materialm + RelCommm -> SA14b_high
##
## inclS PRI covS covU
## ----------------------------------------
## 1 Materialm 0.751 0.582 0.481 0.093
## 2 RelCommm 0.802 0.661 0.604 0.216
## ----------------------------------------
## M1 0.752 0.608 0.697
##
##
## $SA14c_low
##
## M1: Demandm -> SA14c_low
##
## inclS PRI covS covU
## --------------------------------------
## 1 Demandm 0.860 0.824 0.885 -
## --------------------------------------
## M1 0.860 0.824 0.885
##
##
## $SA14c_high
##
## M1: Laborm -> SA14c_high
##
## inclS PRI covS covU
## -------------------------------------
## 1 Laborm 0.756 0.668 0.861 -
## -------------------------------------
## M1 0.756 0.668 0.861
##
##
## $SA15_low
## [1] "Error: None of the values in OUT is explained. Please check the truth table."
##
## $SA15_high
## [1] "Error: None of the values in OUT is explained. Please check the truth table."
##

## $SA16_low
##
## M1: Demandm -> SA16_low
##
## inclS PRI covS covU
## --------------------------------------
## 1 Demandm 0.754 0.663 0.901 -
## --------------------------------------
## M1 0.754 0.663 0.901
##
##
## $SA16_high
## [1] "Error: None of the values in OUT is explained. Please check the truth table."
##
## $SA17_low
##
## M1: Demandm -> SA17_low
##
## inclS PRI covS covU
## --------------------------------------
## 1 Demandm 0.809 0.756 0.890 -
## --------------------------------------
## M1 0.809 0.756 0.890
##
##
## $SA17_high
##
## M1: Materialm + RelCommm -> SA17_high
##
## inclS PRI covS covU
## ----------------------------------------
## 1 Materialm 0.748 0.598 0.464 0.093
## 2 RelCommm 0.781 0.641 0.571 0.199
## ----------------------------------------
## M1 0.738 0.606 0.664
##
##
## $SA18a_low
## [1] "Error: None of the values in OUT is explained. Please check the truth table."
##
## $SA18a_high
## [1] "Error: None of the values in OUT is explained. Please check the truth table."
##
## $SA18b_low
## [1] "Error: None of the values in OUT is explained. Please check the truth table."
##
## $SA18b_high
## [1] "Error: None of the values in OUT is explained. Please check the truth table."
##

## $SA19_low
##
## M1: Demandm -> SA19_low
##
## inclS PRI covS covU
## --------------------------------------
## 1 Demandm 0.791 0.718 0.891 -
## --------------------------------------
## M1 0.791 0.718 0.891
##
##
## $SA19_high
##
## M1: Materialm*~RelCommm -> SA19_high
##
## inclS PRI covS covU
## --------------------------------------------------
## 1 Materialm*~RelCommm 0.767 0.457 0.359 -
## --------------------------------------------------
## M1 0.767 0.457 0.359
##
##
## $SA20_low
##
## M1: Materialm + RelCommm -> SA20_low
##
## inclS PRI covS covU
## ----------------------------------------
## 1 Materialm 0.752 0.586 0.466 0.095
## 2 RelCommm 0.784 0.614 0.572 0.200
## ----------------------------------------
## M1 0.743 0.585 0.666
##
##
## $SA20_high
## [1] "Error: None of the values in OUT is explained. Please check the truth table."
##

## $SA21_low
##
## M1: Demandm -> SA21_low
##
## inclS PRI covS covU
## --------------------------------------
## 1 Demandm 0.807 0.745 0.893 -
## --------------------------------------
## M1 0.807 0.745 0.893
##
##
## $SA21_high
##
## M1: Materialm + RelCommm -> SA21_high
##
## inclS PRI covS covU
## ----------------------------------------
## 1 Materialm 0.725 0.547 0.480 0.085
## 2 RelCommm 0.769 0.593 0.599 0.204
## ----------------------------------------
## M1 0.714 0.545 0.684
##
##
## $SA22_low
##
## M1: Demandm -> SA22_low
##
## inclS PRI covS covU
## --------------------------------------
## 1 Demandm 0.862 0.828 0.877 -
## --------------------------------------
## M1 0.862 0.828 0.877
##
##
## $SA22_high
##
## M1: Laborm -> SA22_high
##
## inclS PRI covS covU
## -------------------------------------
## 1 Laborm 0.761 0.688 0.838 -
## -------------------------------------
## M1 0.761 0.688 0.838
##
##

## $SA23_low
##
## M1: Materialm + RelCommm -> SA23_low
##
## inclS PRI covS covU
## ----------------------------------------
## 1 Materialm 0.773 0.604 0.468 0.097
## 2 RelCommm 0.802 0.640 0.571 0.200
## ----------------------------------------
## M1 0.763 0.604 0.668
##
##
## $SA23_high
## [1] "Error: None of the values in OUT is explained. Please check the truth table."
##
## $SA24_low
## [1] "Error: None of the values in OUT is explained. Please check the truth table."
##
## $SA24_high
## [1] "Error: None of the values in OUT is explained. Please check the truth table."
##
## $SA25_low
##
## M1: Demandm -> SA25_low
##
## inclS PRI covS covU
## --------------------------------------
## 1 Demandm 0.850 0.806 0.889 -
## --------------------------------------
## M1 0.850 0.806 0.889
##
##
## $SA25_high
##
## M1: Laborm -> SA25_high
##
## inclS PRI covS covU
## -------------------------------------
## 1 Laborm 0.722 0.597 0.878 -
## -------------------------------------
## M1 0.722 0.597 0.878

# Intermediate solutions: Positive output including the remainders and with directional expectations

# Result: Final QCA INTERMEDIATE Solutions for Configurations of Reasons for MNC triggering single Outcomes of MNC (n.cut=15)

## $SA1_low
##
## From C1P1:
##
## M1: Demandm*Laborm + Demandm*~Materialm*~RelCommm -> SA1_low
##
## inclS PRI covS covU
## -----------------------------------------------------------
## 1 Demandm*Laborm 0.900 0.862 0.775 0.312
## 2 Demandm*~Materialm*~RelCommm 0.890 0.825 0.539 0.076
## -----------------------------------------------------------
## M1 0.874 0.831 0.851
##
##
## $SA1_high
##
## From C1P1:
##
## M1: Demandm*Laborm*Materialm + Demandm*Laborm*RelCommm -> SA1_high
##
## inclS PRI covS covU
## -------------------------------------------------------
## 1 Demandm*Laborm*Materialm 0.765 0.565 0.439 0.076
## 2 Demandm*Laborm*RelCommm 0.803 0.626 0.560 0.196
## -------------------------------------------------------
## M1 0.768 0.597 0.636
##
##
## $SA2_low
##
## From C1P1:
##
## M1: Demandm*Laborm*Materialm*~RelCommm + Demandm*Laborm*~Materialm*RelCommm
## -> SA2_low
##
## inclS PRI covS covU
## -----------------------------------------------------------------
## 1 Demandm*Laborm*Materialm*~RelCommm 0.803 0.529 0.309 0.102
## 2 Demandm*Laborm*~Materialm*RelCommm 0.813 0.590 0.407 0.200
## -----------------------------------------------------------------
## M1 0.788 0.587 0.509
##
##
## $SA2_high
## [1] "Error: None of the values in OUT is explained. Please check the truth table."
##

## $SA3_low
##
## From C1P1:
##
## M1: Demandm*Laborm*Materialm -> SA3_low
##
## inclS PRI covS covU
## -------------------------------------------------------
## 1 Demandm*Laborm*Materialm 0.797 0.644 0.456 -
## -------------------------------------------------------
## M1 0.797 0.644 0.456
##
##
## $SA3_high
## [1] "Error: None of the values in OUT is explained. Please check the truth table."
##
## $SA4_low
## [1] "Error: None of the values in OUT is explained. Please check the truth table."
##
## $SA4_high
## [1] "Error: None of the values in OUT is explained. Please check the truth table."
##
## $SA5_low
##
## From C1P1:
##
## M1: Demandm*Laborm + Demandm*~Materialm*~RelCommm -> SA5_low
##
## inclS PRI covS covU
## -----------------------------------------------------------
## 1 Demandm*Laborm 0.781 0.666 0.819 0.317
## 2 Demandm*~Materialm*~RelCommm 0.770 0.593 0.569 0.066
## -----------------------------------------------------------
## M1 0.746 0.630 0.885
##
##
## $SA5_high
## [1] "Error: None of the values in OUT is explained. Please check the truth table."
##

## $SA6_low
##
## From C1P1:
##
## M1: Demandm*Laborm -> SA6_low
##
## inclS PRI covS covU
## ---------------------------------------------
## 1 Demandm*Laborm 0.748 0.629 0.826 -
## ---------------------------------------------
## M1 0.748 0.629 0.826
##
##
## $SA6_high
## [1] "Error: None of the values in OUT is explained. Please check the truth table."
##
## $SA7_low
##
## From C1P1:
##
## M1: Demandm*Laborm*Materialm + Demandm*Laborm*RelCommm -> SA7_low
##
## inclS PRI covS covU
## -------------------------------------------------------
## 1 Demandm*Laborm*Materialm 0.821 0.683 0.430 0.082
## 2 Demandm*Laborm*RelCommm 0.845 0.727 0.537 0.189
## -------------------------------------------------------
## M1 0.820 0.701 0.619
##
##
## $SA7_high
## [1] "Error: None of the values in OUT is explained. Please check the truth table."
##

## $SA8_low
##
## From C1P1:
##
## M1: Demandm*Laborm + Demandm*~Materialm*~RelCommm -> SA8_low
##
## inclS PRI covS covU
## -----------------------------------------------------------
## 1 Demandm*Laborm 0.856 0.800 0.766 0.308
## 2 Demandm*~Materialm*~RelCommm 0.854 0.767 0.538 0.080
## -----------------------------------------------------------
## M1 0.836 0.779 0.846
##
##
## $SA8_high
##
## From C1P1:
##
## M1: Demandm*Laborm*Materialm + Demandm*Laborm*RelCommm -> SA8_high
##
## inclS PRI covS covU
## -------------------------------------------------------
## 1 Demandm*Laborm*Materialm 0.758 0.569 0.461 0.078
## 2 Demandm*Laborm*RelCommm 0.772 0.581 0.571 0.188
## -------------------------------------------------------
## M1 0.739 0.561 0.649
##
##
## $SA9a_low
## [1] "Error: All truth table configurations are used, all conditions are minimized. Please check the truth table."
##
## $SA9a_high
##
## From C1P1:
##
## M1: Demandm*Laborm + Demandm*~Materialm*~RelCommm -> SA9a_high
##
## inclS PRI covS covU
## -----------------------------------------------------------
## 1 Demandm*Laborm 0.822 0.751 0.797 0.318
## 2 Demandm*~Materialm*~RelCommm 0.795 0.669 0.542 0.063
## -----------------------------------------------------------
## M1 0.785 0.708 0.860
##
##

## $SA9b_low
## [1] "Error: All truth table configurations are used, all conditions are minimized. Please check the truth table."
##
## $SA9b_high
##
## From C1P1:
##
## M1: Demandm*Laborm + Demandm*~Materialm*~RelCommm -> SA9b_high
##
## inclS PRI covS covU
## -----------------------------------------------------------
## 1 Demandm*Laborm 0.819 0.743 0.793 0.306
## 2 Demandm*~Materialm*~RelCommm 0.811 0.692 0.552 0.066
## -----------------------------------------------------------
## M1 0.784 0.705 0.858
##
##
## $SA10a_low
## [1] "Error: All truth table configurations are used, all conditions are minimized. Please check the truth table."
##
## $SA10a_high
##
## From C1P1:
##
## M1: Demandm*Laborm + Demandm*~Materialm*~RelCommm -> SA10a_high
##
## inclS PRI covS covU
## -----------------------------------------------------------
## 1 Demandm*Laborm 0.811 0.734 0.796 0.309
## 2 Demandm*~Materialm*~RelCommm 0.799 0.675 0.552 0.064
## -----------------------------------------------------------
## M1 0.775 0.694 0.861
##
##
## $SA10b_low
## [1] "Error: All truth table configurations are used, all conditions are minimized. Please check the truth table."
##
## $SA10b_high
##
## From C1P1:
##
## M1: Demandm*Laborm + Demandm*~Materialm*~RelCommm -> SA10b_high
##
## inclS PRI covS covU
## -----------------------------------------------------------
## 1 Demandm*Laborm 0.885 0.845 0.772 0.313
## 2 Demandm*~Materialm*~RelCommm 0.856 0.780 0.526 0.066
## -----------------------------------------------------------
## M1 0.849 0.803 0.838
##
##

## $SA11_low
##
## From C1P1:
##
## M1: Demandm*Laborm*Materialm*~RelCommm + Demandm*Laborm*~Materialm*RelCommm
## -> SA11_low
##
## inclS PRI covS covU
## -----------------------------------------------------------------
## 1 Demandm*Laborm*Materialm*~RelCommm 0.820 0.508 0.328 0.112
## 2 Demandm*Laborm*~Materialm*RelCommm 0.806 0.524 0.418 0.203
## -----------------------------------------------------------------
## M1 0.791 0.543 0.531
##
##
## $SA11_high
## [1] "Error: None of the values in OUT is explained. Please check the truth table."
##
## $SA12_low
##
## From C1P1:
##
## M1: Demandm*Laborm + Demandm*~Materialm*~RelCommm -> SA12_low
##
## inclS PRI covS covU
## -----------------------------------------------------------
## 1 Demandm*Laborm 0.778 0.673 0.817 0.317
## 2 Demandm*~Materialm*~RelCommm 0.769 0.604 0.568 0.068
## -----------------------------------------------------------
## M1 0.746 0.639 0.885
##
##
## $SA12_high
## [1] "Error: None of the values in OUT is explained. Please check the truth table."
##
## $SA13_low
## [1] "Error: None of the values in OUT is explained. Please check the truth table."
##
## $SA13_high
## [1] "Error: None of the values in OUT is explained. Please check the truth table."
##

## $SA14a_low
##
## From C1P1:
##
## M1: Demandm*Laborm -> SA14a_low
##
## inclS PRI covS covU
## ---------------------------------------------
## 1 Demandm*Laborm 0.783 0.671 0.822 -
## ---------------------------------------------
## M1 0.783 0.671 0.822
##
##
## $SA14a_high
## [1] "Error: None of the values in OUT is explained. Please check the truth table."
##
## $SA14b_low
##
## From C1P1:
##
## M1: Demandm*Laborm + Demandm*~Materialm*~RelCommm -> SA14b_low
##
## inclS PRI covS covU
## -----------------------------------------------------------
## 1 Demandm*Laborm 0.896 0.853 0.784 0.320
## 2 Demandm*~Materialm*~RelCommm 0.869 0.786 0.535 0.071
## -----------------------------------------------------------
## M1 0.865 0.815 0.855
##
##
## $SA14b_high
##
## From C1P1:
##
## M1: Demandm*Laborm*Materialm + Demandm*Laborm*RelCommm -> SA14b_high
##
## inclS PRI covS covU
## -------------------------------------------------------
## 1 Demandm*Laborm*Materialm 0.781 0.614 0.461 0.078
## 2 Demandm*Laborm*RelCommm 0.811 0.667 0.580 0.198
## -------------------------------------------------------
## M1 0.775 0.629 0.659
##
##

## $SA14c_low
##
## From C1P1:
##
## M1: Demandm*Laborm + Demandm*~Materialm*~RelCommm -> SA14c_low
##
## inclS PRI covS covU
## -----------------------------------------------------------
## 1 Demandm*Laborm 0.916 0.889 0.759 0.313
## 2 Demandm*~Materialm*~RelCommm 0.893 0.840 0.520 0.075
## -----------------------------------------------------------
## M1 0.890 0.858 0.833
##
##
## $SA14c_high
##
## From C1P1:
##
## M1: Demandm*Laborm -> SA14c_high
##
## inclS PRI covS covU
## ---------------------------------------------
## 1 Demandm*Laborm 0.770 0.680 0.816 -
## ---------------------------------------------
## M1 0.770 0.680 0.816
##
##
## $SA15_low
## [1] "Error: None of the values in OUT is explained. Please check the truth table."
##
## $SA15_high
## [1] "Error: None of the values in OUT is explained. Please check the truth table."
##
## $SA16_low
##
## From C1P1:
##
## M1: Demandm*Laborm + Demandm*~Materialm*~RelCommm -> SA16_low
##
## inclS PRI covS covU
## -----------------------------------------------------------
## 1 Demandm*Laborm 0.822 0.737 0.791 0.311
## 2 Demandm*~Materialm*~RelCommm 0.824 0.700 0.558 0.078
## -----------------------------------------------------------
## M1 0.799 0.714 0.869
##
##
## $SA16_high
## [1] "Error: None of the values in OUT is explained. Please check the truth table."
##

## $SA17_low
##
## From C1P1:
##
## M1: Demandm*Laborm + Demandm*~Materialm*~RelCommm -> SA17_low
##
## inclS PRI covS covU
## -----------------------------------------------------------
## 1 Demandm*Laborm 0.867 0.821 0.768 0.314
## 2 Demandm*~Materialm*~RelCommm 0.852 0.772 0.531 0.076
## -----------------------------------------------------------
## M1 0.843 0.794 0.844
##
##
## $SA17_high
##
## From C1P1:
##
## M1: Demandm*Laborm*Materialm + Demandm*Laborm*RelCommm -> SA17_high
##
## inclS PRI covS covU
## -------------------------------------------------------
## 1 Demandm*Laborm*Materialm 0.779 0.632 0.446 0.079
## 2 Demandm*Laborm*RelCommm 0.788 0.643 0.547 0.181
## -------------------------------------------------------
## M1 0.760 0.624 0.627
##
##
## $SA18a_low
## [1] "Error: None of the values in OUT is explained. Please check the truth table."
##
## $SA18a_high
## [1] "Error: None of the values in OUT is explained. Please check the truth table."
##
## $SA18b_low
## [1] "Error: None of the values in OUT is explained. Please check the truth table."
##
## $SA18b_high
## [1] "Error: None of the values in OUT is explained. Please check the truth table."
##

## $SA19_low
##
## From C1P1:
##
## M1: Demandm*Laborm + Demandm*~Materialm*~RelCommm -> SA19_low
##
## inclS PRI covS covU
## -----------------------------------------------------------
## 1 Demandm*Laborm 0.864 0.803 0.784 0.310
## 2 Demandm*~Materialm*~RelCommm 0.852 0.754 0.544 0.071
## -----------------------------------------------------------
## M1 0.833 0.766 0.854
##
##
## $SA19_high
##
## From C1P1:
##
## M1: Demandm*Laborm*Materialm*~RelCommm -> SA19_high
##
## inclS PRI covS covU
## -----------------------------------------------------------------
## 1 Demandm*Laborm*Materialm*~RelCommm 0.800 0.494 0.350 -
## -----------------------------------------------------------------
## M1 0.800 0.494 0.350
##
##
## $SA20_low
##
## From C1P1:
##
## M1: Demandm*Laborm*Materialm + Demandm*Laborm*RelCommm -> SA20_low
##
## inclS PRI covS covU
## -------------------------------------------------------
## 1 Demandm*Laborm*Materialm 0.781 0.617 0.445 0.080
## 2 Demandm*Laborm*RelCommm 0.797 0.627 0.552 0.187
## -------------------------------------------------------
## M1 0.769 0.610 0.633
##
##
## $SA20_high
## [1] "Error: None of the values in OUT is explained. Please check the truth table."
##

## $SA21_low
##
## From C1P1:
##
## M1: Demandm*Laborm + Demandm*~Materialm*~RelCommm -> SA21_low
##
## inclS PRI covS covU
## -----------------------------------------------------------
## 1 Demandm*Laborm 0.869 0.817 0.774 0.311
## 2 Demandm*~Materialm*~RelCommm 0.863 0.780 0.541 0.078
## -----------------------------------------------------------
## M1 0.846 0.790 0.852
##
##
## $SA21_high
##
## From C1P1:
##
## M1: Demandm*Laborm*Materialm + Demandm*Laborm*RelCommm -> SA21_high
##
## inclS PRI covS covU
## -------------------------------------------------------
## 1 Demandm*Laborm*Materialm 0.759 0.582 0.462 0.075
## 2 Demandm*Laborm*RelCommm 0.778 0.597 0.575 0.188
## -------------------------------------------------------
## M1 0.740 0.568 0.650
##
##

## $SA22_low
##
## From C1P1:
##
## M1: Demandm*Laborm + Demandm*~Materialm*~RelCommm -> SA22_low
##
## inclS PRI covS covU
## -----------------------------------------------------------
## 1 Demandm*Laborm 0.909 0.881 0.745 0.302
## 2 Demandm*~Materialm*~RelCommm 0.903 0.858 0.521 0.078
## -----------------------------------------------------------
## M1 0.887 0.856 0.822
##
##
## $SA22_high
##
## From C1P1:
##
## M1: Demandm*Laborm -> SA22_high
##
## inclS PRI covS covU
## ---------------------------------------------
## 1 Demandm*Laborm 0.770 0.695 0.790 -
## ---------------------------------------------
## M1 0.770 0.695 0.790
##
##
## $SA23_low
##
## From C1P1:
##
## M1: Demandm*Laborm*Materialm + Demandm*Laborm*RelCommm -> SA23_low
##
## inclS PRI covS covU
## -------------------------------------------------------
## 1 Demandm*Laborm*Materialm 0.801 0.635 0.446 0.083
## 2 Demandm*Laborm*RelCommm 0.811 0.646 0.548 0.185
## -------------------------------------------------------
## M1 0.786 0.627 0.631
##
##
## $SA23_high
## [1] "Error: None of the values in OUT is explained. Please check the truth table."
##

## $SA24_low
## [1] "Error: None of the values in OUT is explained. Please check the truth table."
##
## $SA24_high
## [1] "Error: None of the values in OUT is explained. Please check the truth table."
##
## $SA25_low
##
## From C1P1:
##
## M1: Demandm*Laborm + Demandm*~Materialm*~RelCommm -> SA25_low
##
## inclS PRI covS covU
## -----------------------------------------------------------
## 1 Demandm*Laborm 0.903 0.867 0.760 0.306
## 2 Demandm*~Materialm*~RelCommm 0.908 0.856 0.538 0.084
## -----------------------------------------------------------
## M1 0.886 0.849 0.844
##
##
## $SA25_high
##
## From C1P1:
##
## M1: Demandm*Laborm -> SA25_high
##
## inclS PRI covS covU
## ---------------------------------------------
## 1 Demandm*Laborm 0.737 0.610 0.835 -
## ---------------------------------------------
## M1 0.737 0.610 0.835

# Overview of Results -> See Article, "Table 3 - Configuration chart for intermediate solutions for 1st level conditions and missed care"

####################################################
### Data Processing 3/3 ###
####################################################

#### De Morgan Laws ---> Testing for Causal Asymmetry
# Crating a list for the Negation of each of the Outcomes of MNC (neg_outcome_MNC) (~outcome_MNC)
neg_outcome_MNC <- paste("~", outcome_MNC, sep="")
# Print neg_outcome_MNC list
neg_outcome_MNC

## [1] "~SA1_low" "~SA1_high" "~SA2_low" "~SA2_high" "~SA3_low"
## [6] "~SA3_high" "~SA4_low" "~SA4_high" "~SA5_low" "~SA5_high"
## [11] "~SA6_low" "~SA6_high" "~SA7_low" "~SA7_high" "~SA8_low"
## [16] "~SA8_high" "~SA9a_low" "~SA9a_high" "~SA9b_low" "~SA9b_high"
## [21] "~SA10a_low" "~SA10a_high" "~SA10b_low" "~SA10b_high" "~SA11_low"
## [26] "~SA11_high" "~SA12_low" "~SA12_high" "~SA13_low" "~SA13_high"
## [31] "~SA14a_low" "~SA14a_high" "~SA14b_low" "~SA14b_high" "~SA14c_low"
## [36] "~SA14c_high" "~SA15_low" "~SA15_high" "~SA16_low" "~SA16_high"
## [41] "~SA17_low" "~SA17_high" "~SA18a_low" "~SA18a_high" "~SA18b_low"
## [46] "~SA18b_high" "~SA19_low" "~SA19_high" "~SA20_low" "~SA20_high"
## [51] "~SA21_low" "~SA21_high" "~SA22_low" "~SA22_high" "~SA23_low"
## [56] "~SA23_high" "~SA24_low" "~SA24_high" "~SA25_low" "~SA25_high"

#### Performing QCA: Truth Tables: 1st Level conditions -> MNC
# Result: Truth Table: Reasons for MNC triggering the negated single Outcomes of MNC (for n.cut = 15)

## $SA1_low
##
## OUT: output value
## n: number of cases in configuration
## incl: sufficiency inclusion score
## PRI: proportional reduction in inconsistency
##
## Demandm Laborm Materialm RelCommm OUT n incl PRI
## 1 0 0 0 0 0 39 0.776 0.518
## 9 1 0 0 0 0 59 0.643 0.226
## 15 1 1 1 0 0 45 0.586 0.107
## 14 1 1 0 1 0 56 0.531 0.054
## 16 1 1 1 1 0 65 0.506 0.103
## 13 1 1 0 0 0 115 0.493 0.094
##
## It seems that all output values have been coded to zero.
## Suggestion: lower the inclusion score for the presence of the outcome,
## the relevant argument is "incl.cut" which now has a value of 0.8.
##
##

## $SA1_high
##
## OUT: output value
## n: number of cases in configuration
## incl: sufficiency inclusion score
## PRI: proportional reduction in inconsistency
##
## Demandm Laborm Materialm RelCommm OUT n incl PRI
## 1 0 0 0 0 1 39 0.919 0.824
## 9 1 0 0 0 1 59 0.831 0.595
## 15 1 1 1 0 0 45 0.756 0.371
## 14 1 1 0 1 0 56 0.703 0.278
## 13 1 1 0 0 0 115 0.701 0.401
## 16 1 1 1 1 0 65 0.696 0.328
##
##
## $SA2_low
##
## OUT: output value
## n: number of cases in configuration
## incl: sufficiency inclusion score
## PRI: proportional reduction in inconsistency
##
## Demandm Laborm Materialm RelCommm OUT n incl PRI
## 1 0 0 0 0 1 39 0.895 0.776
## 9 1 0 0 0 1 59 0.820 0.581
## 15 1 1 1 0 0 45 0.773 0.457
## 14 1 1 0 1 0 56 0.726 0.397
## 13 1 1 0 0 0 115 0.719 0.452
## 16 1 1 1 1 0 65 0.702 0.381
##
##
## $SA2_high
##
## OUT: output value
## n: number of cases in configuration
## incl: sufficiency inclusion score
## PRI: proportional reduction in inconsistency
##
## Demandm Laborm Materialm RelCommm OUT n incl PRI
## 1 0 0 0 0 1 39 0.978 0.959
## 9 1 0 0 0 1 59 0.952 0.905
## 15 1 1 1 0 1 45 0.910 0.804
## 13 1 1 0 0 1 115 0.902 0.828
## 16 1 1 1 1 1 65 0.872 0.752
## 14 1 1 0 1 1 56 0.867 0.720
##
##

## $SA3_low
##
## OUT: output value
## n: number of cases in configuration
## incl: sufficiency inclusion score
## PRI: proportional reduction in inconsistency
##
## Demandm Laborm Materialm RelCommm OUT n incl PRI
## 1 0 0 0 0 1 39 0.916 0.840
## 9 1 0 0 0 1 59 0.804 0.592
## 15 1 1 1 0 0 45 0.723 0.345
## 14 1 1 0 1 0 56 0.717 0.434
## 13 1 1 0 0 0 115 0.690 0.447
## 16 1 1 1 1 0 65 0.647 0.307
##
##
## $SA3_high
##
## OUT: output value
## n: number of cases in configuration
## incl: sufficiency inclusion score
## PRI: proportional reduction in inconsistency
##
## Demandm Laborm Materialm RelCommm OUT n incl PRI
## 1 0 0 0 0 1 39 0.972 0.950
## 9 1 0 0 0 1 59 0.937 0.879
## 15 1 1 1 0 1 45 0.881 0.721
## 13 1 1 0 0 1 115 0.870 0.772
## 14 1 1 0 1 1 56 0.847 0.687
## 16 1 1 1 1 1 65 0.816 0.640
##
##

## $SA4_low
##
## OUT: output value
## n: number of cases in configuration
## incl: sufficiency inclusion score
## PRI: proportional reduction in inconsistency
##
## Demandm Laborm Materialm RelCommm OUT n incl PRI
## 1 0 0 0 0 1 39 0.925 0.855
## 9 1 0 0 0 1 59 0.867 0.713
## 15 1 1 1 0 0 45 0.782 0.472
## 13 1 1 0 0 0 115 0.766 0.547
## 14 1 1 0 1 0 56 0.763 0.453
## 16 1 1 1 1 0 65 0.719 0.429
##
##
## $SA4_high
##
## OUT: output value
## n: number of cases in configuration
## incl: sufficiency inclusion score
## PRI: proportional reduction in inconsistency
##
## Demandm Laborm Materialm RelCommm OUT n incl PRI
## 1 0 0 0 0 1 39 0.981 0.967
## 9 1 0 0 0 1 59 0.967 0.940
## 13 1 1 0 0 1 115 0.934 0.890
## 15 1 1 1 0 1 45 0.920 0.828
## 14 1 1 0 1 1 56 0.916 0.828
## 16 1 1 1 1 1 65 0.866 0.745
##
##

## $SA5_low
##
## OUT: output value
## n: number of cases in configuration
## incl: sufficiency inclusion score
## PRI: proportional reduction in inconsistency
##
## Demandm Laborm Materialm RelCommm OUT n incl PRI
## 1 0 0 0 0 1 39 0.884 0.747
## 9 1 0 0 0 0 59 0.789 0.472
## 15 1 1 1 0 0 45 0.693 0.231
## 14 1 1 0 1 0 56 0.691 0.263
## 13 1 1 0 0 0 115 0.661 0.302
## 16 1 1 1 1 0 65 0.618 0.217
##
##
## $SA5_high
##
## OUT: output value
## n: number of cases in configuration
## incl: sufficiency inclusion score
## PRI: proportional reduction in inconsistency
##
## Demandm Laborm Materialm RelCommm OUT n incl PRI
## 1 0 0 0 0 1 39 0.975 0.951
## 9 1 0 0 0 1 59 0.940 0.871
## 15 1 1 1 0 1 45 0.872 0.665
## 13 1 1 0 0 1 115 0.872 0.754
## 14 1 1 0 1 1 56 0.870 0.697
## 16 1 1 1 1 1 65 0.808 0.592
##
##

## $SA6_low
##
## OUT: output value
## n: number of cases in configuration
## incl: sufficiency inclusion score
## PRI: proportional reduction in inconsistency
##
## Demandm Laborm Materialm RelCommm OUT n incl PRI
## 1 0 0 0 0 1 39 0.889 0.766
## 9 1 0 0 0 0 59 0.798 0.532
## 15 1 1 1 0 0 45 0.711 0.337
## 14 1 1 0 1 0 56 0.701 0.309
## 13 1 1 0 0 0 115 0.668 0.350
## 16 1 1 1 1 0 65 0.616 0.253
##
##
## $SA6_high
##
## OUT: output value
## n: number of cases in configuration
## incl: sufficiency inclusion score
## PRI: proportional reduction in inconsistency
##
## Demandm Laborm Materialm RelCommm OUT n incl PRI
## 1 0 0 0 0 1 39 0.974 0.950
## 9 1 0 0 0 1 59 0.939 0.878
## 14 1 1 0 1 1 56 0.867 0.709
## 13 1 1 0 0 1 115 0.865 0.757
## 15 1 1 1 0 1 45 0.851 0.654
## 16 1 1 1 1 0 65 0.786 0.571
##
##

## $SA7_low
##
## OUT: output value
## n: number of cases in configuration
## incl: sufficiency inclusion score
## PRI: proportional reduction in inconsistency
##
## Demandm Laborm Materialm RelCommm OUT n incl PRI
## 1 0 0 0 0 1 39 0.874 0.733
## 9 1 0 0 0 0 59 0.792 0.551
## 15 1 1 1 0 0 45 0.721 0.311
## 13 1 1 0 0 0 115 0.668 0.375
## 14 1 1 0 1 0 56 0.659 0.256
## 16 1 1 1 1 0 65 0.616 0.228
##
##
## $SA7_high
##
## OUT: output value
## n: number of cases in configuration
## incl: sufficiency inclusion score
## PRI: proportional reduction in inconsistency
##
## Demandm Laborm Materialm RelCommm OUT n incl PRI
## 1 0 0 0 0 1 39 0.973 0.950
## 9 1 0 0 0 1 59 0.918 0.838
## 15 1 1 1 0 1 45 0.874 0.707
## 13 1 1 0 0 1 115 0.867 0.758
## 14 1 1 0 1 1 56 0.833 0.619
## 16 1 1 1 1 0 65 0.786 0.573
##
##

## $SA8_low
##
## OUT: output value
## n: number of cases in configuration
## incl: sufficiency inclusion score
## PRI: proportional reduction in inconsistency
##
## Demandm Laborm Materialm RelCommm OUT n incl PRI
## 1 0 0 0 0 0 39 0.765 0.471
## 9 1 0 0 0 0 59 0.663 0.257
## 15 1 1 1 0 0 45 0.601 0.158
## 14 1 1 0 1 0 56 0.566 0.131
## 13 1 1 0 0 0 115 0.536 0.174
## 16 1 1 1 1 0 65 0.524 0.156
##
## It seems that all output values have been coded to zero.
## Suggestion: lower the inclusion score for the presence of the outcome,
## the relevant argument is "incl.cut" which now has a value of 0.8.
##
##
## $SA8_high
##
## OUT: output value
## n: number of cases in configuration
## incl: sufficiency inclusion score
## PRI: proportional reduction in inconsistency
##
## Demandm Laborm Materialm RelCommm OUT n incl PRI
## 1 0 0 0 0 1 39 0.908 0.809
## 9 1 0 0 0 1 59 0.853 0.663
## 15 1 1 1 0 0 45 0.759 0.405
## 13 1 1 0 0 0 115 0.741 0.492
## 14 1 1 0 1 0 56 0.735 0.379
## 16 1 1 1 1 0 65 0.690 0.342
##
##

## $SA9a_low
##
## OUT: output value
## n: number of cases in configuration
## incl: sufficiency inclusion score
## PRI: proportional reduction in inconsistency
##
## Demandm Laborm Materialm RelCommm OUT n incl PRI
## 1 0 0 0 0 0 39 0.674 0.330
## 9 1 0 0 0 0 59 0.526 0.101
## 15 1 1 1 0 0 45 0.485 0.035
## 14 1 1 0 1 0 56 0.434 0.034
## 16 1 1 1 1 0 65 0.419 0.063
## 13 1 1 0 0 0 115 0.403 0.056
##
## It seems that all output values have been coded to zero.
## Suggestion: lower the inclusion score for the presence of the outcome,
## the relevant argument is "incl.cut" which now has a value of 0.8.
##
##
## $SA9a_high
##
## OUT: output value
## n: number of cases in configuration
## incl: sufficiency inclusion score
## PRI: proportional reduction in inconsistency
##
## Demandm Laborm Materialm RelCommm OUT n incl PRI
## 1 0 0 0 0 1 39 0.851 0.664
## 9 1 0 0 0 0 59 0.721 0.361
## 15 1 1 1 0 0 45 0.633 0.185
## 13 1 1 0 0 0 115 0.583 0.236
## 14 1 1 0 1 0 56 0.580 0.147
## 16 1 1 1 1 0 65 0.543 0.148
##
##

## $SA9b_low
##
## OUT: output value
## n: number of cases in configuration
## incl: sufficiency inclusion score
## PRI: proportional reduction in inconsistency
##
## Demandm Laborm Materialm RelCommm OUT n incl PRI
## 1 0 0 0 0 0 39 0.682 0.348
## 9 1 0 0 0 0 59 0.510 0.076
## 15 1 1 1 0 0 45 0.486 0.053
## 14 1 1 0 1 0 56 0.433 0.041
## 16 1 1 1 1 0 65 0.419 0.062
## 13 1 1 0 0 0 115 0.393 0.059
##
## It seems that all output values have been coded to zero.
## Suggestion: lower the inclusion score for the presence of the outcome,
## the relevant argument is "incl.cut" which now has a value of 0.8.
##
##
## $SA9b_high
##
## OUT: output value
## n: number of cases in configuration
## incl: sufficiency inclusion score
## PRI: proportional reduction in inconsistency
##
## Demandm Laborm Materialm RelCommm OUT n incl PRI
## 1 0 0 0 0 1 39 0.841 0.650
## 9 1 0 0 0 0 59 0.713 0.340
## 15 1 1 1 0 0 45 0.634 0.186
## 14 1 1 0 1 0 56 0.595 0.153
## 13 1 1 0 0 0 115 0.574 0.210
## 16 1 1 1 1 0 65 0.557 0.160
##
##

## $SA10a_low
##
## OUT: output value
## n: number of cases in configuration
## incl: sufficiency inclusion score
## PRI: proportional reduction in inconsistency
##
## Demandm Laborm Materialm RelCommm OUT n incl PRI
## 1 0 0 0 0 0 39 0.656 0.294
## 9 1 0 0 0 0 59 0.521 0.102
## 15 1 1 1 0 0 45 0.483 0.030
## 14 1 1 0 1 0 56 0.442 0.040
## 16 1 1 1 1 0 65 0.417 0.048
## 13 1 1 0 0 0 115 0.403 0.066
##
## It seems that all output values have been coded to zero.
## Suggestion: lower the inclusion score for the presence of the outcome,
## the relevant argument is "incl.cut" which now has a value of 0.8.
##
##
## $SA10a_high
##
## OUT: output value
## n: number of cases in configuration
## incl: sufficiency inclusion score
## PRI: proportional reduction in inconsistency
##
## Demandm Laborm Materialm RelCommm OUT n incl PRI
## 1 0 0 0 0 1 39 0.824 0.621
## 9 1 0 0 0 0 59 0.711 0.348
## 15 1 1 1 0 0 45 0.616 0.166
## 14 1 1 0 1 0 56 0.591 0.157
## 13 1 1 0 0 0 115 0.575 0.225
## 16 1 1 1 1 0 65 0.560 0.183
##
##

## $SA10b_low
##
## OUT: output value
## n: number of cases in configuration
## incl: sufficiency inclusion score
## PRI: proportional reduction in inconsistency
##
## Demandm Laborm Materialm RelCommm OUT n incl PRI
## 1 0 0 0 0 0 39 0.620 0.273
## 9 1 0 0 0 0 59 0.457 0.067
## 15 1 1 1 0 0 45 0.428 0.020
## 14 1 1 0 1 0 56 0.372 0.018
## 16 1 1 1 1 0 65 0.353 0.022
## 13 1 1 0 0 0 115 0.338 0.039
##
## It seems that all output values have been coded to zero.
## Suggestion: lower the inclusion score for the presence of the outcome,
## the relevant argument is "incl.cut" which now has a value of 0.8.
##
##
## $SA10b_high
##
## OUT: output value
## n: number of cases in configuration
## incl: sufficiency inclusion score
## PRI: proportional reduction in inconsistency
##
## Demandm Laborm Materialm RelCommm OUT n incl PRI
## 1 0 0 0 0 0 39 0.784 0.540
## 9 1 0 0 0 0 59 0.637 0.247
## 15 1 1 1 0 0 45 0.547 0.101
## 14 1 1 0 1 0 56 0.494 0.073
## 13 1 1 0 0 0 115 0.481 0.124
## 16 1 1 1 1 0 65 0.458 0.070
##
## It seems that all output values have been coded to zero.
## Suggestion: lower the inclusion score for the presence of the outcome,
## the relevant argument is "incl.cut" which now has a value of 0.8.
##
##

## $SA11_low
##
## OUT: output value
## n: number of cases in configuration
## incl: sufficiency inclusion score
## PRI: proportional reduction in inconsistency
##
## Demandm Laborm Materialm RelCommm OUT n incl PRI
## 1 0 0 0 0 1 39 0.927 0.839
## 9 1 0 0 0 1 59 0.848 0.604
## 15 1 1 1 0 1 45 0.806 0.469
## 14 1 1 0 1 0 56 0.773 0.441
## 13 1 1 0 0 0 115 0.758 0.467
## 16 1 1 1 1 0 65 0.720 0.393
##
##
## $SA11_high
##
## OUT: output value
## n: number of cases in configuration
## incl: sufficiency inclusion score
## PRI: proportional reduction in inconsistency
##
## Demandm Laborm Materialm RelCommm OUT n incl PRI
## 1 0 0 0 0 1 39 0.980 0.965
## 9 1 0 0 0 1 59 0.974 0.951
## 13 1 1 0 0 1 115 0.950 0.914
## 15 1 1 1 0 1 45 0.941 0.867
## 14 1 1 0 1 1 56 0.917 0.824
## 16 1 1 1 1 1 65 0.896 0.794
##
##

## $SA12_low
##
## OUT: output value
## n: number of cases in configuration
## incl: sufficiency inclusion score
## PRI: proportional reduction in inconsistency
##
## Demandm Laborm Materialm RelCommm OUT n incl PRI
## 1 0 0 0 0 1 39 0.874 0.732
## 9 1 0 0 0 0 59 0.764 0.425
## 15 1 1 1 0 0 45 0.686 0.289
## 14 1 1 0 1 0 56 0.652 0.256
## 13 1 1 0 0 0 115 0.645 0.298
## 16 1 1 1 1 0 65 0.616 0.281
##
##
## $SA12_high
##
## OUT: output value
## n: number of cases in configuration
## incl: sufficiency inclusion score
## PRI: proportional reduction in inconsistency
##
## Demandm Laborm Materialm RelCommm OUT n incl PRI
## 1 0 0 0 0 1 39 0.942 0.893
## 9 1 0 0 0 1 59 0.912 0.820
## 13 1 1 0 0 1 115 0.842 0.712
## 15 1 1 1 0 1 45 0.823 0.601
## 14 1 1 0 1 1 56 0.806 0.573
## 16 1 1 1 1 0 65 0.774 0.559
##
##

## $SA13_low
##
## OUT: output value
## n: number of cases in configuration
## incl: sufficiency inclusion score
## PRI: proportional reduction in inconsistency
##
## Demandm Laborm Materialm RelCommm OUT n incl PRI
## 1 0 0 0 0 1 39 0.922 0.865
## 9 1 0 0 0 1 59 0.903 0.826
## 15 1 1 1 0 1 45 0.854 0.704
## 13 1 1 0 0 1 115 0.837 0.735
## 14 1 1 0 1 0 56 0.796 0.628
## 16 1 1 1 1 0 65 0.755 0.549
##
##
## $SA13_high
##
## OUT: output value
## n: number of cases in configuration
## incl: sufficiency inclusion score
## PRI: proportional reduction in inconsistency
##
## Demandm Laborm Materialm RelCommm OUT n incl PRI
## 1 0 0 0 0 1 39 0.980 0.967
## 9 1 0 0 0 1 59 0.968 0.950
## 15 1 1 1 0 1 45 0.954 0.917
## 13 1 1 0 0 1 115 0.944 0.918
## 14 1 1 0 1 1 56 0.891 0.813
## 16 1 1 1 1 1 65 0.889 0.814
##
##

## $SA14a_low
##
## OUT: output value
## n: number of cases in configuration
## incl: sufficiency inclusion score
## PRI: proportional reduction in inconsistency
##
## Demandm Laborm Materialm RelCommm OUT n incl PRI
## 1 0 0 0 0 1 39 0.862 0.695
## 9 1 0 0 0 0 59 0.794 0.485
## 15 1 1 1 0 0 45 0.713 0.260
## 14 1 1 0 1 0 56 0.677 0.273
## 13 1 1 0 0 0 115 0.664 0.321
## 16 1 1 1 1 0 65 0.610 0.178
##
##
## $SA14a_high
##
## OUT: output value
## n: number of cases in configuration
## incl: sufficiency inclusion score
## PRI: proportional reduction in inconsistency
##
## Demandm Laborm Materialm RelCommm OUT n incl PRI
## 1 0 0 0 0 1 39 0.950 0.905
## 9 1 0 0 0 1 59 0.937 0.872
## 15 1 1 1 0 1 45 0.880 0.707
## 13 1 1 0 0 1 115 0.858 0.740
## 14 1 1 0 1 1 56 0.842 0.627
## 16 1 1 1 1 1 65 0.806 0.590
##
##

## $SA14b_low
##
## OUT: output value
## n: number of cases in configuration
## incl: sufficiency inclusion score
## PRI: proportional reduction in inconsistency
##
## Demandm Laborm Materialm RelCommm OUT n incl PRI
## 1 0 0 0 0 1 39 0.812 0.588
## 9 1 0 0 0 0 59 0.664 0.222
## 15 1 1 1 0 0 45 0.584 0.088
## 14 1 1 0 1 0 56 0.529 0.067
## 13 1 1 0 0 0 115 0.524 0.117
## 16 1 1 1 1 0 65 0.483 0.060
##
##
## $SA14b_high
##
## OUT: output value
## n: number of cases in configuration
## incl: sufficiency inclusion score
## PRI: proportional reduction in inconsistency
##
## Demandm Laborm Materialm RelCommm OUT n incl PRI
## 1 0 0 0 0 1 39 0.927 0.845
## 9 1 0 0 0 1 59 0.837 0.643
## 15 1 1 1 0 0 45 0.745 0.373
## 13 1 1 0 0 0 115 0.716 0.470
## 14 1 1 0 1 0 56 0.674 0.281
## 16 1 1 1 1 0 65 0.640 0.275
##
##

## $SA14c_low
##
## OUT: output value
## n: number of cases in configuration
## incl: sufficiency inclusion score
## PRI: proportional reduction in inconsistency
##
## Demandm Laborm Materialm RelCommm OUT n incl PRI
## 1 0 0 0 0 0 39 0.756 0.503
## 9 1 0 0 0 0 59 0.586 0.178
## 15 1 1 1 0 0 45 0.527 0.068
## 14 1 1 0 1 0 56 0.456 0.052
## 13 1 1 0 0 0 115 0.443 0.086
## 16 1 1 1 1 0 65 0.434 0.056
##
## It seems that all output values have been coded to zero.
## Suggestion: lower the inclusion score for the presence of the outcome,
## the relevant argument is "incl.cut" which now has a value of 0.8.
##
##
## $SA14c_high
##
## OUT: output value
## n: number of cases in configuration
## incl: sufficiency inclusion score
## PRI: proportional reduction in inconsistency
##
## Demandm Laborm Materialm RelCommm OUT n incl PRI
## 1 0 0 0 0 1 39 0.886 0.768
## 9 1 0 0 0 0 59 0.754 0.488
## 15 1 1 1 0 0 45 0.687 0.300
## 13 1 1 0 0 0 115 0.608 0.310
## 14 1 1 0 1 0 56 0.578 0.170
## 16 1 1 1 1 0 65 0.565 0.193
##
##

## $SA15_low
##
## OUT: output value
## n: number of cases in configuration
## incl: sufficiency inclusion score
## PRI: proportional reduction in inconsistency
##
## Demandm Laborm Materialm RelCommm OUT n incl PRI
## 1 0 0 0 0 1 39 0.952 0.918
## 9 1 0 0 0 1 59 0.920 0.857
## 15 1 1 1 0 1 45 0.878 0.749
## 14 1 1 0 1 1 56 0.850 0.717
## 13 1 1 0 0 1 115 0.850 0.746
## 16 1 1 1 1 1 65 0.815 0.665
##
##
## $SA15_high
##
## OUT: output value
## n: number of cases in configuration
## incl: sufficiency inclusion score
## PRI: proportional reduction in inconsistency
##
## Demandm Laborm Materialm RelCommm OUT n incl PRI
## 9 1 0 0 0 1 59 0.985 0.977
## 1 0 0 0 0 1 39 0.985 0.976
## 13 1 1 0 0 1 115 0.967 0.952
## 15 1 1 1 0 1 45 0.961 0.932
## 14 1 1 0 1 1 56 0.947 0.911
## 16 1 1 1 1 1 65 0.924 0.875
##
##

## $SA16_low
##
## OUT: output value
## n: number of cases in configuration
## incl: sufficiency inclusion score
## PRI: proportional reduction in inconsistency
##
## Demandm Laborm Materialm RelCommm OUT n incl PRI
## 1 0 0 0 0 1 39 0.832 0.613
## 9 1 0 0 0 0 59 0.709 0.319
## 15 1 1 1 0 0 45 0.687 0.203
## 14 1 1 0 1 0 56 0.622 0.176
## 13 1 1 0 0 0 115 0.598 0.216
## 16 1 1 1 1 0 65 0.575 0.140
##
##
## $SA16_high
##
## OUT: output value
## n: number of cases in configuration
## incl: sufficiency inclusion score
## PRI: proportional reduction in inconsistency
##
## Demandm Laborm Materialm RelCommm OUT n incl PRI
## 1 0 0 0 0 1 39 0.950 0.900
## 9 1 0 0 0 1 59 0.898 0.770
## 15 1 1 1 0 1 45 0.867 0.644
## 13 1 1 0 0 1 115 0.815 0.630
## 14 1 1 0 1 0 56 0.792 0.492
## 16 1 1 1 1 0 65 0.764 0.488
##
##

## $SA17_low
##
## OUT: output value
## n: number of cases in configuration
## incl: sufficiency inclusion score
## PRI: proportional reduction in inconsistency
##
## Demandm Laborm Materialm RelCommm OUT n incl PRI
## 1 0 0 0 0 0 39 0.772 0.549
## 9 1 0 0 0 0 59 0.624 0.231
## 15 1 1 1 0 0 45 0.552 0.121
## 14 1 1 0 1 0 56 0.519 0.114
## 13 1 1 0 0 0 115 0.506 0.160
## 16 1 1 1 1 0 65 0.477 0.108
##
## It seems that all output values have been coded to zero.
## Suggestion: lower the inclusion score for the presence of the outcome,
## the relevant argument is "incl.cut" which now has a value of 0.8.
##
##
## $SA17_high
##
## OUT: output value
## n: number of cases in configuration
## incl: sufficiency inclusion score
## PRI: proportional reduction in inconsistency
##
## Demandm Laborm Materialm RelCommm OUT n incl PRI
## 1 0 0 0 0 1 39 0.876 0.765
## 9 1 0 0 0 0 59 0.796 0.580
## 15 1 1 1 0 0 45 0.693 0.321
## 13 1 1 0 0 0 115 0.675 0.423
## 14 1 1 0 1 0 56 0.661 0.294
## 16 1 1 1 1 0 65 0.633 0.291
##
##

## $SA18a_low
##
## OUT: output value
## n: number of cases in configuration
## incl: sufficiency inclusion score
## PRI: proportional reduction in inconsistency
##
## Demandm Laborm Materialm RelCommm OUT n incl PRI
## 1 0 0 0 0 1 39 0.886 0.779
## 9 1 0 0 0 1 59 0.806 0.585
## 15 1 1 1 0 0 45 0.794 0.517
## 14 1 1 0 1 0 56 0.745 0.456
## 13 1 1 0 0 0 115 0.720 0.483
## 16 1 1 1 1 0 65 0.689 0.369
##
##
## $SA18a_high
##
## OUT: output value
## n: number of cases in configuration
## incl: sufficiency inclusion score
## PRI: proportional reduction in inconsistency
##
## Demandm Laborm Materialm RelCommm OUT n incl PRI
## 1 0 0 0 0 1 39 0.945 0.900
## 9 1 0 0 0 1 59 0.930 0.867
## 15 1 1 1 0 1 45 0.925 0.845
## 13 1 1 0 0 1 115 0.888 0.814
## 14 1 1 0 1 1 56 0.877 0.757
## 16 1 1 1 1 1 65 0.864 0.744
##
##

## $SA18b_low
##
## OUT: output value
## n: number of cases in configuration
## incl: sufficiency inclusion score
## PRI: proportional reduction in inconsistency
##
## Demandm Laborm Materialm RelCommm OUT n incl PRI
## 1 0 0 0 0 1 39 0.927 0.864
## 15 1 1 1 0 1 45 0.872 0.721
## 9 1 0 0 0 1 59 0.860 0.713
## 14 1 1 0 1 1 56 0.810 0.617
## 13 1 1 0 0 0 115 0.793 0.630
## 16 1 1 1 1 0 65 0.757 0.525
##
##
## $SA18b_high
##
## OUT: output value
## n: number of cases in configuration
## incl: sufficiency inclusion score
## PRI: proportional reduction in inconsistency
##
## Demandm Laborm Materialm RelCommm OUT n incl PRI
## 1 0 0 0 0 1 39 0.977 0.960
## 9 1 0 0 0 1 59 0.971 0.949
## 15 1 1 1 0 1 45 0.956 0.919
## 13 1 1 0 0 1 115 0.945 0.914
## 14 1 1 0 1 1 56 0.917 0.851
## 16 1 1 1 1 1 65 0.903 0.834
##
##

## $SA19_low
##
## OUT: output value
## n: number of cases in configuration
## incl: sufficiency inclusion score
## PRI: proportional reduction in inconsistency
##
## Demandm Laborm Materialm RelCommm OUT n incl PRI
## 1 0 0 0 0 0 39 0.780 0.499
## 9 1 0 0 0 0 59 0.696 0.288
## 15 1 1 1 0 0 45 0.622 0.114
## 14 1 1 0 1 0 56 0.587 0.120
## 13 1 1 0 0 0 115 0.552 0.153
## 16 1 1 1 1 0 65 0.552 0.139
##
## It seems that all output values have been coded to zero.
## Suggestion: lower the inclusion score for the presence of the outcome,
## the relevant argument is "incl.cut" which now has a value of 0.8.
##
##
## $SA19_high
##
## OUT: output value
## n: number of cases in configuration
## incl: sufficiency inclusion score
## PRI: proportional reduction in inconsistency
##
## Demandm Laborm Materialm RelCommm OUT n incl PRI
## 1 0 0 0 0 1 39 0.934 0.862
## 9 1 0 0 0 1 59 0.867 0.686
## 15 1 1 1 0 0 45 0.797 0.485
## 14 1 1 0 1 0 56 0.767 0.448
## 13 1 1 0 0 0 115 0.763 0.527
## 16 1 1 1 1 0 65 0.714 0.419
##
##

## $SA20_low
##
## OUT: output value
## n: number of cases in configuration
## incl: sufficiency inclusion score
## PRI: proportional reduction in inconsistency
##
## Demandm Laborm Materialm RelCommm OUT n incl PRI
## 1 0 0 0 0 1 39 0.906 0.798
## 9 1 0 0 0 1 59 0.856 0.661
## 15 1 1 1 0 0 45 0.753 0.397
## 14 1 1 0 1 0 56 0.737 0.375
## 13 1 1 0 0 0 115 0.724 0.434
## 16 1 1 1 1 0 65 0.662 0.307
##
##
## $SA20_high
##
## OUT: output value
## n: number of cases in configuration
## incl: sufficiency inclusion score
## PRI: proportional reduction in inconsistency
##
## Demandm Laborm Materialm RelCommm OUT n incl PRI
## 1 0 0 0 0 1 39 0.986 0.975
## 9 1 0 0 0 1 59 0.976 0.952
## 13 1 1 0 0 1 115 0.929 0.871
## 15 1 1 1 0 1 45 0.921 0.807
## 14 1 1 0 1 1 56 0.904 0.784
## 16 1 1 1 1 1 65 0.839 0.663
##
##

## $SA21_low
##
## OUT: output value
## n: number of cases in configuration
## incl: sufficiency inclusion score
## PRI: proportional reduction in inconsistency
##
## Demandm Laborm Materialm RelCommm OUT n incl PRI
## 1 0 0 0 0 0 39 0.787 0.539
## 9 1 0 0 0 0 59 0.651 0.232
## 15 1 1 1 0 0 45 0.587 0.096
## 14 1 1 0 1 0 56 0.552 0.118
## 13 1 1 0 0 0 115 0.521 0.147
## 16 1 1 1 1 0 65 0.507 0.106
##
## It seems that all output values have been coded to zero.
## Suggestion: lower the inclusion score for the presence of the outcome,
## the relevant argument is "incl.cut" which now has a value of 0.8.
##
##
## $SA21_high
##
## OUT: output value
## n: number of cases in configuration
## incl: sufficiency inclusion score
## PRI: proportional reduction in inconsistency
##
## Demandm Laborm Materialm RelCommm OUT n incl PRI
## 1 0 0 0 0 1 39 0.917 0.823
## 9 1 0 0 0 1 59 0.836 0.628
## 15 1 1 1 0 0 45 0.746 0.398
## 14 1 1 0 1 0 56 0.728 0.352
## 13 1 1 0 0 0 115 0.724 0.460
## 16 1 1 1 1 0 65 0.667 0.323
##
##

## $SA22_low
##
## OUT: output value
## n: number of cases in configuration
## incl: sufficiency inclusion score
## PRI: proportional reduction in inconsistency
##
## Demandm Laborm Materialm RelCommm OUT n incl PRI
## 1 0 0 0 0 0 39 0.720 0.450
## 9 1 0 0 0 0 59 0.548 0.155
## 15 1 1 1 0 0 45 0.474 0.087
## 14 1 1 0 1 0 56 0.456 0.058
## 13 1 1 0 0 0 115 0.424 0.078
## 16 1 1 1 1 0 65 0.418 0.077
##
## It seems that all output values have been coded to zero.
## Suggestion: lower the inclusion score for the presence of the outcome,
## the relevant argument is "incl.cut" which now has a value of 0.8.
##
##
## $SA22_high
##
## OUT: output value
## n: number of cases in configuration
## incl: sufficiency inclusion score
## PRI: proportional reduction in inconsistency
##
## Demandm Laborm Materialm RelCommm OUT n incl PRI
## 1 0 0 0 0 1 39 0.843 0.701
## 9 1 0 0 0 0 59 0.700 0.430
## 14 1 1 0 1 0 56 0.575 0.230
## 13 1 1 0 0 0 115 0.572 0.297
## 15 1 1 1 0 0 45 0.568 0.171
## 16 1 1 1 1 0 65 0.526 0.202
##
##

## $SA23_low
##
## OUT: output value
## n: number of cases in configuration
## incl: sufficiency inclusion score
## PRI: proportional reduction in inconsistency
##
## Demandm Laborm Materialm RelCommm OUT n incl PRI
## 1 0 0 0 0 1 39 0.937 0.863
## 9 1 0 0 0 1 59 0.831 0.552
## 15 1 1 1 0 0 45 0.772 0.398
## 14 1 1 0 1 0 56 0.747 0.384
## 13 1 1 0 0 0 115 0.717 0.387
## 16 1 1 1 1 0 65 0.654 0.270
##
##
## $SA23_high
##
## OUT: output value
## n: number of cases in configuration
## incl: sufficiency inclusion score
## PRI: proportional reduction in inconsistency
##
## Demandm Laborm Materialm RelCommm OUT n incl PRI
## 1 0 0 0 0 1 39 0.991 0.984
## 9 1 0 0 0 1 59 0.971 0.942
## 15 1 1 1 0 1 45 0.938 0.850
## 13 1 1 0 0 1 115 0.925 0.863
## 14 1 1 0 1 1 56 0.900 0.772
## 16 1 1 1 1 1 65 0.843 0.663
##
##

## $SA24_low
##
## OUT: output value
## n: number of cases in configuration
## incl: sufficiency inclusion score
## PRI: proportional reduction in inconsistency
##
## Demandm Laborm Materialm RelCommm OUT n incl PRI
## 1 0 0 0 0 1 39 0.929 0.847
## 9 1 0 0 0 1 59 0.845 0.646
## 15 1 1 1 0 1 45 0.811 0.536
## 14 1 1 0 1 1 56 0.803 0.548
## 13 1 1 0 0 0 115 0.780 0.561
## 16 1 1 1 1 0 65 0.709 0.403
##
##
## $SA24_high
##
## OUT: output value
## n: number of cases in configuration
## incl: sufficiency inclusion score
## PRI: proportional reduction in inconsistency
##
## Demandm Laborm Materialm RelCommm OUT n incl PRI
## 1 0 0 0 0 1 39 0.992 0.985
## 9 1 0 0 0 1 59 0.966 0.935
## 15 1 1 1 0 1 45 0.959 0.909
## 13 1 1 0 0 1 115 0.953 0.920
## 14 1 1 0 1 1 56 0.941 0.880
## 16 1 1 1 1 1 65 0.881 0.763
##
##

## $SA25_low
##
## OUT: output value
## n: number of cases in configuration
## incl: sufficiency inclusion score
## PRI: proportional reduction in inconsistency
##
## Demandm Laborm Materialm RelCommm OUT n incl PRI
## 1 0 0 0 0 0 39 0.762 0.482
## 9 1 0 0 0 0 59 0.606 0.151
## 15 1 1 1 0 0 45 0.585 0.107
## 14 1 1 0 1 0 56 0.514 0.074
## 16 1 1 1 1 0 65 0.488 0.082
## 13 1 1 0 0 0 115 0.472 0.087
##
## It seems that all output values have been coded to zero.
## Suggestion: lower the inclusion score for the presence of the outcome,
## the relevant argument is "incl.cut" which now has a value of 0.8.
##
##
## $SA25_high
##
## OUT: output value
## n: number of cases in configuration
## incl: sufficiency inclusion score
## PRI: proportional reduction in inconsistency
##
## Demandm Laborm Materialm RelCommm OUT n incl PRI
## 1 0 0 0 0 1 39 0.900 0.784
## 9 1 0 0 0 1 59 0.811 0.537
## 15 1 1 1 0 0 45 0.743 0.349
## 13 1 1 0 0 0 115 0.682 0.361
## 14 1 1 0 1 0 56 0.670 0.245
## 16 1 1 1 1 0 65 0.635 0.262

#### Performing QCA: Analysis of Sufficiency and Minimization
#### FOR n.cut (minimum number of cases with membership > 0.5 for an outcome) -> n15:n.cut=15

# Intermediate solutions: Positive output including the remainders and with directional expectations

# Result: Final QCA INTERMEDIATE Solutions for Configurations of Reasons for MNC triggering negated single Outcomes of MNC (n.cut=15)

## $SA1_low
## [1] "Error: None of the values in OUT is explained. Please check the truth table."
##
## $SA1_high
##
## From C1P1:
##
## M1: ~Laborm*~Materialm*~RelCommm -> ~SA1_high
##
## inclS PRI covS covU
## -----------------------------------------------------------
## 1 ~Laborm*~Materialm*~RelCommm 0.843 0.702 0.559 -
## -----------------------------------------------------------
## M1 0.843 0.702 0.559
##
##
## $SA2_low
##
## From C1P1:
##
## M1: ~Laborm*~Materialm*~RelCommm -> ~SA2_low
##
## inclS PRI covS covU
## -----------------------------------------------------------
## 1 ~Laborm*~Materialm*~RelCommm 0.811 0.646 0.537 -
## -----------------------------------------------------------
## M1 0.811 0.646 0.537
##
##
## $SA2_high
## [1] "Error: All truth table configurations are used, all conditions are minimized. Please check the truth table."
##
## $SA3_low
##
## From C1P1:
##
## M1: ~Laborm*~Materialm*~RelCommm -> ~SA3_low
##
## inclS PRI covS covU
## -----------------------------------------------------------
## 1 ~Laborm*~Materialm*~RelCommm 0.816 0.684 0.542 -
## -----------------------------------------------------------
## M1 0.816 0.684 0.542
##
##
## $SA3_high
## [1] "Error: All truth table configurations are used, all conditions are minimized. Please check the truth table."
##
## $SA4_low
##
## From C1P1:
##
## M1: ~Laborm*~Materialm*~RelCommm -> ~SA4_low
##
## inclS PRI covS covU
## -----------------------------------------------------------
## 1 ~Laborm*~Materialm*~RelCommm 0.857 0.746 0.518 -
## -----------------------------------------------------------
## M1 0.857 0.746 0.518
##
##
## $SA4_high
## [1] "Error: All truth table configurations are used, all conditions are minimized. Please check the truth table."
##
## $SA5_low
##
## From C1P1:
##
## M1: ~Demandm*~Laborm*~Materialm*~RelCommm -> ~SA5_low
##
## inclS PRI covS covU
## --------------------------------------------------------------------
## 1 ~Demandm*~Laborm*~Materialm*~RelCommm 0.884 0.747 0.399 -
## --------------------------------------------------------------------
## M1 0.884 0.747 0.399
##
##
## $SA5_high
## [1] "Error: All truth table configurations are used, all conditions are minimized. Please check the truth table."
##
## $SA6_low
##
## From C1P1:
##
## M1: ~Demandm*~Laborm*~Materialm*~RelCommm -> ~SA6_low
##
## inclS PRI covS covU
## --------------------------------------------------------------------
## 1 ~Demandm*~Laborm*~Materialm*~RelCommm 0.889 0.766 0.376 -
## --------------------------------------------------------------------
## M1 0.889 0.766 0.376
##
##

## $SA6_high
##
## From C1P1:
##
## M1: ~Materialm + ~RelCommm -> ~SA6_high
##
## inclS PRI covS covU
## -----------------------------------------
## 1 ~Materialm 0.784 0.711 0.801 0.114
## 2 ~RelCommm 0.826 0.759 0.773 0.086
## -----------------------------------------
## M1 0.769 0.697 0.887
##
##
## $SA7_low
##
## From C1P1:
##
## M1: ~Demandm*~Laborm*~Materialm*~RelCommm -> ~SA7_low
##
## inclS PRI covS covU
## --------------------------------------------------------------------
## 1 ~Demandm*~Laborm*~Materialm*~RelCommm 0.874 0.733 0.379 -
## --------------------------------------------------------------------
## M1 0.874 0.733 0.379
##
##
## $SA7_high
##
## From C1P1:
##
## M1: ~Materialm + ~RelCommm -> ~SA7_high
##
## inclS PRI covS covU
## -----------------------------------------
## 1 ~Materialm 0.764 0.684 0.793 0.097
## 2 ~RelCommm 0.829 0.763 0.788 0.091
## -----------------------------------------
## M1 0.755 0.679 0.884
##
##
## $SA8_low
## [1] "Error: None of the values in OUT is explained. Please check the truth table."
##
## $SA8_high
##
## From C1P1:
##
## M1: ~Laborm*~Materialm*~RelCommm -> ~SA8_high
##
## inclS PRI covS covU
## -----------------------------------------------------------
## 1 ~Laborm*~Materialm*~RelCommm 0.856 0.734 0.535 -
## -----------------------------------------------------------
## M1 0.856 0.734 0.535
##
##
## $SA9a_low
## [1] "Error: None of the values in OUT is explained. Please check the truth table."
##
## $SA9a_high
##
## From C1P1:
##
## M1: ~Demandm*~Laborm*~Materialm*~RelCommm -> ~SA9a_high
##
## inclS PRI covS covU
## --------------------------------------------------------------------
## 1 ~Demandm*~Laborm*~Materialm*~RelCommm 0.851 0.664 0.435 -
## --------------------------------------------------------------------
## M1 0.851 0.664 0.435
##
##
## $SA9b_low
## [1] "Error: None of the values in OUT is explained. Please check the truth table."
##
## $SA9b_high
##
## From C1P1:
##
## M1: ~Demandm*~Laborm*~Materialm*~RelCommm -> ~SA9b_high
##
## inclS PRI covS covU
## --------------------------------------------------------------------
## 1 ~Demandm*~Laborm*~Materialm*~RelCommm 0.841 0.650 0.431 -
## --------------------------------------------------------------------
## M1 0.841 0.650 0.431
##
##
## $SA10a_low
## [1] "Error: None of the values in OUT is explained. Please check the truth table."
##
## $SA10a_high
##
## From C1P1:
##
## M1: ~Demandm*~Laborm*~Materialm*~RelCommm -> ~SA10a_high
##
## inclS PRI covS covU
## --------------------------------------------------------------------
## 1 ~Demandm*~Laborm*~Materialm*~RelCommm 0.824 0.621 0.413 -
## --------------------------------------------------------------------
## M1 0.824 0.621 0.413
##
##
## $SA10b_low
## [1] "Error: None of the values in OUT is explained. Please check the truth table."
##

## $SA10b_high
## [1] "Error: None of the values in OUT is explained. Please check the truth table."
##
## $SA11_low
##
## From C1P1:
##
## M1: ~Laborm*~RelCommm + Materialm*~RelCommm -> ~SA11_low
##
## inclS PRI covS covU
## --------------------------------------------------
## 1 ~Laborm*~RelCommm 0.835 0.671 0.557 0.324
## 2 Materialm*~RelCommm 0.790 0.466 0.332 0.100
## --------------------------------------------------
## M1 0.783 0.589 0.656
##
##
## $SA11_high
## [1] "Error: All truth table configurations are used, all conditions are minimized. Please check the truth table."
##
## $SA12_low
##
## From C1P1:
##
## M1: ~Demandm*~Laborm*~Materialm*~RelCommm -> ~SA12_low
##
## inclS PRI covS covU
## --------------------------------------------------------------------
## 1 ~Demandm*~Laborm*~Materialm*~RelCommm 0.874 0.732 0.395 -
## --------------------------------------------------------------------
## M1 0.874 0.732 0.395
##
##
## $SA12_high
##
## From C1P1:
##
## M1: ~Materialm + ~RelCommm -> ~SA12_high
##
## inclS PRI covS covU
## -----------------------------------------
## 1 ~Materialm 0.745 0.660 0.797 0.098
## 2 ~RelCommm 0.803 0.727 0.787 0.088
## -----------------------------------------
## M1 0.734 0.650 0.885
##
##

## $SA13_low
##
## From C1P1:
##
## M1: ~RelCommm -> ~SA13_low
##
## inclS PRI covS covU
## ----------------------------------------
## 1 ~RelCommm 0.786 0.715 0.772 -
## ----------------------------------------
## M1 0.786 0.715 0.772
##
##
## $SA13_high
## [1] "Error: All truth table configurations are used, all conditions are minimized. Please check the truth table."
##
## $SA14a_low
##
## From C1P1:
##
## M1: ~Demandm*~Laborm*~Materialm*~RelCommm -> ~SA14a_low
##
## inclS PRI covS covU
## --------------------------------------------------------------------
## 1 ~Demandm*~Laborm*~Materialm*~RelCommm 0.862 0.695 0.389 -
## --------------------------------------------------------------------
## M1 0.862 0.695 0.389
##
##
## $SA14a_high
## [1] "Error: All truth table configurations are used, all conditions are minimized. Please check the truth table."
##
## $SA14b_low
##
## From C1P1:
##
## M1: ~Demandm*~Laborm*~Materialm*~RelCommm -> ~SA14b_low
##
## inclS PRI covS covU
## --------------------------------------------------------------------
## 1 ~Demandm*~Laborm*~Materialm*~RelCommm 0.812 0.588 0.510 -
## --------------------------------------------------------------------
## M1 0.812 0.588 0.510
##
##

## $SA14b_high
##
## From C1P1:
##
## M1: ~Laborm*~Materialm*~RelCommm -> ~SA14b_high
##
## inclS PRI covS covU
## -----------------------------------------------------------
## 1 ~Laborm*~Materialm*~RelCommm 0.847 0.727 0.546 -
## -----------------------------------------------------------
## M1 0.847 0.727 0.546
##
##
## $SA14c_low
## [1] "Error: None of the values in OUT is explained. Please check the truth table."
##
## $SA14c_high
##
## From C1P1:
##
## M1: ~Demandm*~Laborm*~Materialm*~RelCommm -> ~SA14c_high
##
## inclS PRI covS covU
## --------------------------------------------------------------------
## 1 ~Demandm*~Laborm*~Materialm*~RelCommm 0.886 0.768 0.395 -
## --------------------------------------------------------------------
## M1 0.886 0.768 0.395
##
##
## $SA15_low
## [1] "Error: All truth table configurations are used, all conditions are minimized. Please check the truth table."
##
## $SA15_high
## [1] "Error: All truth table configurations are used, all conditions are minimized. Please check the truth table."
##
## $SA16_low
##
## From C1P1:
##
## M1: ~Demandm*~Laborm*~Materialm*~RelCommm -> ~SA16_low
##
## inclS PRI covS covU
## --------------------------------------------------------------------
## 1 ~Demandm*~Laborm*~Materialm*~RelCommm 0.832 0.613 0.431 -
## --------------------------------------------------------------------
## M1 0.832 0.613 0.431
##
##

## $SA16_high
##
## From C1P1:
##
## M1: ~RelCommm -> ~SA16_high
##
## inclS PRI covS covU
## ----------------------------------------
## 1 ~RelCommm 0.774 0.672 0.811 -
## ----------------------------------------
## M1 0.774 0.672 0.811
##
##
## $SA17_low
## [1] "Error: None of the values in OUT is explained. Please check the truth table."
##
## $SA17_high
##
## From C1P1:
##
## M1: ~Demandm*~Laborm*~Materialm*~RelCommm -> ~SA17_high
##
## inclS PRI covS covU
## --------------------------------------------------------------------
## 1 ~Demandm*~Laborm*~Materialm*~RelCommm 0.876 0.765 0.342 -
## --------------------------------------------------------------------
## M1 0.876 0.765 0.342
##
##
## $SA18a_low
##
## From C1P1:
##
## M1: ~Laborm*~Materialm*~RelCommm -> ~SA18a_low
##
## inclS PRI covS covU
## -----------------------------------------------------------
## 1 ~Laborm*~Materialm*~RelCommm 0.808 0.662 0.512 -
## -----------------------------------------------------------
## M1 0.808 0.662 0.512
##
##

## $SA18a_high
## [1] "Error: All truth table configurations are used, all conditions are minimized. Please check the truth table."
##
## $SA18b_low
##
## From C1P1:
##
## M1: ~Materialm*RelCommm + Materialm*~RelCommm + (~Laborm*~Materialm)
## -> ~SA18b_low
## M2: ~Materialm*RelCommm + Materialm*~RelCommm + (~Laborm*~RelCommm)
## -> ~SA18b_low
## -------------------
## inclS PRI covS covU (M1) (M2)
## ----------------------------------------------------------------
## 1 ~Materialm*RelCommm 0.798 0.606 0.363 0.090 0.090 0.093
## 2 Materialm*~RelCommm 0.859 0.705 0.308 0.063 0.082 0.063
## ----------------------------------------------------------------
## 3 ~Laborm*~Materialm 0.859 0.759 0.471 0.001 0.212
## 4 ~Laborm*~RelCommm 0.859 0.760 0.488 0.003 0.213
## ----------------------------------------------------------------
## M1 0.786 0.670 0.686
## M2 0.785 0.670 0.687
##
##
## $SA18b_high
## [1] "Error: All truth table configurations are used, all conditions are minimized. Please check the truth table."
##
## $SA19_low
## [1] "Error: None of the values in OUT is explained. Please check the truth table."
##
## $SA19_high
##
## From C1P1:
##
## M1: ~Laborm*~Materialm*~RelCommm -> ~SA19_high
##
## inclS PRI covS covU
## -----------------------------------------------------------
## 1 ~Laborm*~Materialm*~RelCommm 0.877 0.769 0.515 -
## -----------------------------------------------------------
## M1 0.877 0.769 0.515
##
##
## $SA20_low
##
## From C1P1:
##
## M1: ~Laborm*~Materialm*~RelCommm -> ~SA20_low
##
## inclS PRI covS covU
## -----------------------------------------------------------
## 1 ~Laborm*~Materialm*~RelCommm 0.840 0.692 0.561 -
## -----------------------------------------------------------
## M1 0.840 0.692 0.561
##
##
## $SA20_high
## [1] "Error: All truth table configurations are used, all conditions are minimized. Please check the truth table."
##
## $SA21_low
## [1] "Error: None of the values in OUT is explained. Please check the truth table."
##
## $SA21_high
##
## From C1P1:
##
## M1: ~Laborm*~Materialm*~RelCommm -> ~SA21_high
##
## inclS PRI covS covU
## -----------------------------------------------------------
## 1 ~Laborm*~Materialm*~RelCommm 0.849 0.723 0.531 -
## -----------------------------------------------------------
## M1 0.849 0.723 0.531
##
##
## $SA22_low
## [1] "Error: None of the values in OUT is explained. Please check the truth table."
##
## $SA22_high
##
## From C1P1:
##
## M1: ~Demandm*~Laborm*~Materialm*~RelCommm -> ~SA22_high
##
## inclS PRI covS covU
## --------------------------------------------------------------------
## 1 ~Demandm*~Laborm*~Materialm*~RelCommm 0.843 0.701 0.394 -
## --------------------------------------------------------------------
## M1 0.843 0.701 0.394
##
##
## $SA23_low
##
## From C1P1:
##
## M1: ~Laborm*~Materialm*~RelCommm -> ~SA23_low
##
## inclS PRI covS covU
## -----------------------------------------------------------
## 1 ~Laborm*~Materialm*~RelCommm 0.832 0.663 0.571 -
## -----------------------------------------------------------
## M1 0.832 0.663 0.571
##
##
## $SA23_high
## [1] "Error: All truth table configurations are used, all conditions are minimized. Please check the truth table."
##
## $SA24_low
##
## From C1P1:
##
## M1: ~Materialm*RelCommm + Materialm*~RelCommm + (~Laborm*~Materialm)
## -> ~SA24_low
## M2: ~Materialm*RelCommm + Materialm*~RelCommm + (~Laborm*~RelCommm)
## -> ~SA24_low
## -------------------
## inclS PRI covS covU (M1) (M2)
## ----------------------------------------------------------------
## 1 ~Materialm*RelCommm 0.793 0.544 0.403 0.093 0.093 0.097
## 2 Materialm*~RelCommm 0.792 0.522 0.317 0.046 0.064 0.046
## ----------------------------------------------------------------
## 3 ~Laborm*~Materialm 0.837 0.699 0.512 0.001 0.219
## 4 ~Laborm*~RelCommm 0.833 0.690 0.529 0.003 0.221
## ----------------------------------------------------------------
## M1 0.736 0.562 0.717
## M2 0.736 0.563 0.719
##
##
## $SA24_high
## [1] "Error: All truth table configurations are used, all conditions are minimized. Please check the truth table."
##
## $SA25_low
## [1] "Error: None of the values in OUT is explained. Please check the truth table."
##
## $SA25_high
##
## From C1P1:
##
## M1: ~Laborm*~Materialm*~RelCommm -> ~SA25_high
##
## inclS PRI covS covU
## -----------------------------------------------------------
## 1 ~Laborm*~Materialm*~RelCommm 0.821 0.655 0.574 -
## -----------------------------------------------------------
## M1 0.821 0.655 0.574
